# Effects of Paraquat, Dextran Sulfate Sodium, and Irradiation on Behavioral and Cognitive Performance and the Gut Microbiome in A53T and A53T-L444P Mice

**DOI:** 10.3390/genes15030282

**Published:** 2024-02-23

**Authors:** Ariel Chaklai, Abigail O’Neil, Shrey Goel, Nick Margolies, Destine Krenik, Ruby Perez, Kat Kessler, Elizabeth Staltontall, Hong Ki (Eric) Yoon, Montzerrat Pantoja, Keaton Stagaman, Kristin Kasschau, Vivek Unni, Robert Duvoisin, Thomas Sharpton, Jacob Raber

**Affiliations:** 1 Department of Behavioral Neuroscience, Oregon Health & Science University, Portland, OR 97239, USA; achaklai@pdx.edu (A.C.); oniela@ohsu.edu (A.O.); goels@ohsu.edu (S.G.); margolie@ohsu.edu (N.M.); krenik@ohsu.edu (D.K.); rubyre.perez@gmail.com (R.P.); katkessler9@gmail.com (K.K.); saltonse@ohsu.edu (E.S.); pantoja@ohsu.edu (M.P.); 2 Department of Microbiology, Oregon State University, Corvallis, OR 97331, USA; kstagaman@gmail.com (K.S.); kristin.kasschau@oregonstate.edu (K.K.); thomas.sharpton@oregonstate.edu (T.S.); 3 Department of Neurology, Oregon Health & Science University, Portland, OR 97239, USA; unni@ohsu.edu; 4 Jungers Center, Oregon Health & Science University, Portland, OR 97239, USA; 5 Department of Chemical Physiology and Biochemistry, Oregon Health & Science University, Portland, OR 97239, USA; duvoisin@ohsu.edu; 6 Department of Statistics, Oregon State University, Corvallis, OR 97331, USA; 7 Department of Radiation Medicine, Oregon Health & Science University, Portland, OR 97239, USA; 8 Division of Neuroscience, Oregon National Primate Research Center, Oregon Health & Science University, Portland, OR 97239, USA

**Keywords:** paraquat, dextran sulfate sodium, irradiation, Gaucher mutation, alpha synuclein mutation, behavioral testing, cognitive testing, gut microbiome

## Abstract

Heterozygous carriers of the glucocerebrosidase 1 (GBA) L444P Gaucher mutation have an increased risk of developing Parkinson’s disease (PD). The GBA mutations result in elevated alpha synuclein (aSyn) levels. Heterozygous mice carrying one allele with the L444P mutation knocked-into the mouse gene show increased aSyn levels and are more sensitive to motor deficits following exposure to the neurotoxin (1-methyl-4-phenyl-1,2,3,6-tetrahydropyridine) MPTP than wild-type mice. Paraquat (PQ), a herbicide, increases PD risk in most studies. Its effects on the brain involve alterations in the gut microbiome. Exposure to dextran sulfate sodium (DSS), a mouse model of colitis, can be used to determine whether gut microbiome alterations are sufficient to induce PD-relevant phenotypes. We rederived the A53T-L444P and A53T mouse lines to assess whether PQ, PQ in combination with radiation exposure (IR), and DSS have differential effects in A53T and A53T-L444P mice and whether these effects are associated with alterations in the gut microbiome. PQ and PQ + IR have differential effects in A53T and A53T-L444P mice. In contrast, effects of DSS are only seen in A53T-L444P mice. Exposure and genotype modulate the relationship between the gut microbiome and behavioral performance. The gut microbiome may be an important mediator of how environmental exposures or genetic mutations yield behavioral and cognitive impacts.

## 1. Introduction

In Parkinson’s Disease (PD), cognitive symptoms often do not receive a lot of attention but they account for disability and reduced life expectancy [1,2]. PD patients also exhibit depression [3,4], a risk factor for developing PD [5]. PD patients with depression have greater problems with concentration than PD patients without depression [6,7]. An increased understanding of the mechanisms underlying behavioral alterations and cognitive impairments in PD are timely and critical.

Many PD patients show digestive symptoms before neurological symptoms and show an altered composition of the gut microbiome. The gut microbiome regulates impaired motor function and brain inflammation in alpha-synuclein (aSyn) transgenic mice [8]. aSyn is a major constituent of Lewy bodies, which are the pathological hallmark of PD [9]. The gut microbiome also plays a role in the effects of PD-related environmental neurotoxins. Parkin is important in PD pathogenesis and in regulating microbial load homeostasis [10]. Flies mutant for parkin are more sensitive to the neurotoxic herbicide paraquat (PQ) [11]. While PQ cannot be purchased in the UK, where it is produced, or in the EU, it is still widely used in the USA and elsewhere. In most studies, PQ increases the risk of developing PD (for reviews, see [12,13,14,15]), including association with PD in the Agricultural Health Study [16] and in particular following head injury [14]. The sensitivity of flies mutant for parkin to PQ is lower in germ-free flies [10]. In addition, in rats, vagotomy prevents PD symptoms and reduces aSyn pathology following combined administration of subthreshold doses of PQ and lectins [17].

aSyn is involved in the immune response. aSyn levels in the gut and brain are elevated following exposure to certain bacteria and correlate with inflammation severity in children with gut infections [18]. Elevated aSyn levels may induce aSyn pathology in the gut and spread to the brain via the gut–brain axis and, in this way, drive PD pathogenesis [19]. Chronic exposure of young mice expressing aSyn with the A53T mutation to colitis causing dextran sulfate sodium (DSS), results in more pronounced behavioral alterations, neuropathology, and gut and brain inflammation within 3 [20] to 21 months [21,22] after initiation of the DSS exposure than seen in control A53T littermates. The gut microbiome and gut–brain axis also play a role in anxiety and depression [23,24,25].

In humans, heterozygous carriers of the glucocerebrosidase 1 (GBA) L444P Gaucher mutation have an increased PD risk [26,27] and GBA mutations are associated with an earlier age at onset, a faster decline, and a higher risk of developing psychosis or dementia. The GBA mutations lower enzymatic activity of glucocerebrosidase (Gcase), causing a buildup of glycosphingolipid substrates, different ceramide species including glucosylceramide, and hexosylsphingosine, resulting in elevated aSyn levels [28]. Aggregated aSyn may further depress Gcase activity, further worsening the condition. Heterozygous mice carrying one allele with the L444P mutation knocked-into the mouse gene (GBA*^+/L444P^*; B6;129S4-*Gba^tm1RlpMmnc^*) have reduced GBA protein and enzymatic activity levels, show increased aSyn levels in the midbrain, and are more susceptible than wild-type mice to motor deficits, neuroinflammation, and dopamine (DA) neuronal pathology following exposure to the neurotoxin (1-methyl-4-phenyl-1,2,3,6-tetrahydropyridine) MPTP [29]. The combined aSyn A53T and GBA L444P genotype exacerbates the motor and gastrointestinal (GI) phenotypes at 14 to 15 months of age and increases measures of anxiety in the elevated zero maze at 20 months of age as compared to the A53T genotype alone [30].

Radiation exposure and associated gut dysbiosis can occur as part of a cancer treatment, a dirty bomb scenario, or a nuclear attack or incident [31]. Occupational chronic ionizing radiation exposure led to increased risk of PD in workers of a nuclear facility [32]. Resident GI microbiota are disrupted by radiation exposure to 10 Gy of ionizing radiation, a dose equivalent to a radionuclear event [33], and the administration of microbial metabolites prior to radiation exposure reduces dysbiosis of the gut microbiota and increases survival in animal models [34]. Our data show that radiation exposure affects behavioral and cognitive performance and the composition of the gut microbiome [35,36]. PQ was shown to interact with ionizing radiation to impair spatial learning and memory and enhance toxicity in mice [37].

While DSS primarily induces inflammation in the gut, PQ can affect the gut microbiome and gut–brain axis and also have other systemic effects. Therefore, assessing effects of both PQ and DSS can help to obtain a convergence of evidence for the role of the gut microbiome and gut–brain axis in PD (neuro)toxin models. In the current study, we rederived the A53T-L444P and A53T mouse lines originally generated by Dr. Nussbaum [30] to assess whether PQ (study 1) and DSS (study 4) have differential effects in A53T and A53T-L444P mice and whether these effects are associated with alterations in the gut microbiome. As PQ can interact with ionizing radiation to induce cognitive injury [37], and irradiation might increase PD risk [32], we also assessed the effects of radiation exposure by itself (study 2) and in combination with PQ (study 3) on behavioral and cognitive performance in A53T and A53T-L444P mice.

## 2. Materials and Methods

### 2.1. Animals

We used A53T-L444P and A53T mice. These lines were originally generated by Dr. Nussbaum [30]. As there were no live mice available of the A53T-L444P and A53T models, we pursued a cryo-recovery and in vitro fertilization effort to produce these two genotypes as littermates at the Jackson Laboratory, Bar Harbor, ME, in close consultation with Dr. Sarah Edie from the Jackson Laboratory. Pups were born on 21 January 2020. We set up breeding cages each containing 1 A53T-L444P female and 1 A53T male, or 1 A53T-L444P male and 1 A53T female mouse. The A53T-L444P and A53T mice we used were all littermates. Due to the complexity of the genetic model, including homozygosity for A53T, and the fact that these mice are not on a homozygous background strain, we did not use wild-type littermates. While in principle it would be possible to generate wild-type littermates, the percent of mice with the desired genotype out of all mice generated does not justify this approach, especially as the main comparisons are within a genotype. The genotype (L444P) of the mice was determined by PCR at Transnetyx, Cordova, TN, USA. The forward primer was CCAGAGAGTGGCGTTGGT, the reporter 1 was AGCACTGACTTGGAAAC, the reverse primer was CAGGGCGTAACAGTGCTACT, and the reporter 2 was CACTGACCCGGAAAC. Overall, 50.3 ± 3.5% (*n* = 8 sample sets) of the offspring was heterozygous for L444P. The average litter size was 7 pups. Twenty breeding pairs were set up to generate the experimental mice used in this study. No mortality or adverse effects based on genotype were noted.

The mice received PicoLab Rodent Diet 20, no. 5053 (PMI Nutrition International, St. Louis, MO, USA) provided ad libitum. The female and male mice were tested in one of four studies, at the age indicated and as described in detail below. All procedures were approved by the OHSU Institutional Animal Care and Use Committee and in compliance with all federal regulations.

### 2.2. Treatment Paradigms and Behavioral Tests

**Figure 1:**
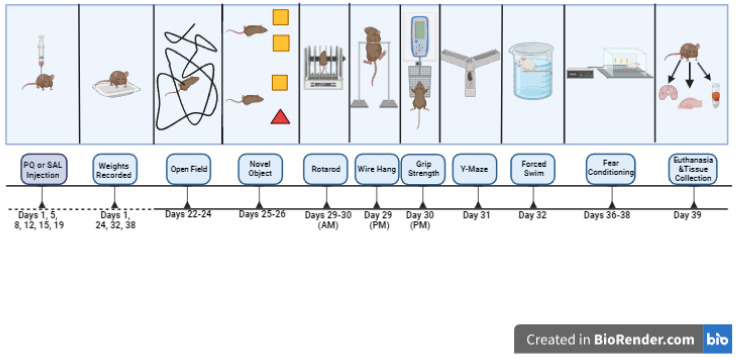
Schematic outline of study 1.

**Figure 2:**
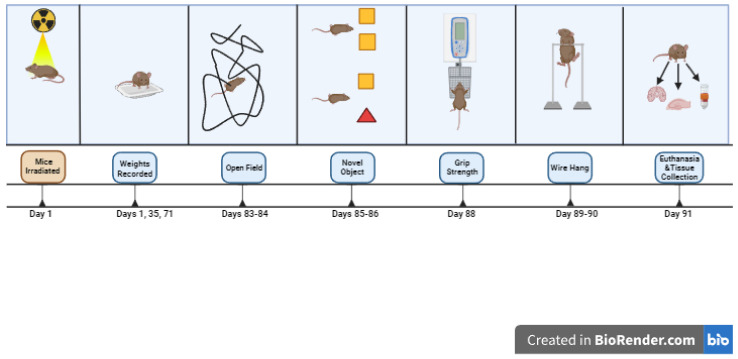
Schematic outline of study 2.

**Figure 3:**
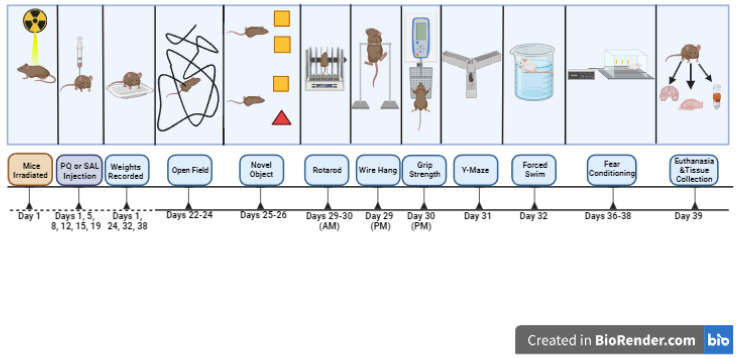
Schematic outline of study 3.

**Figure 4:**
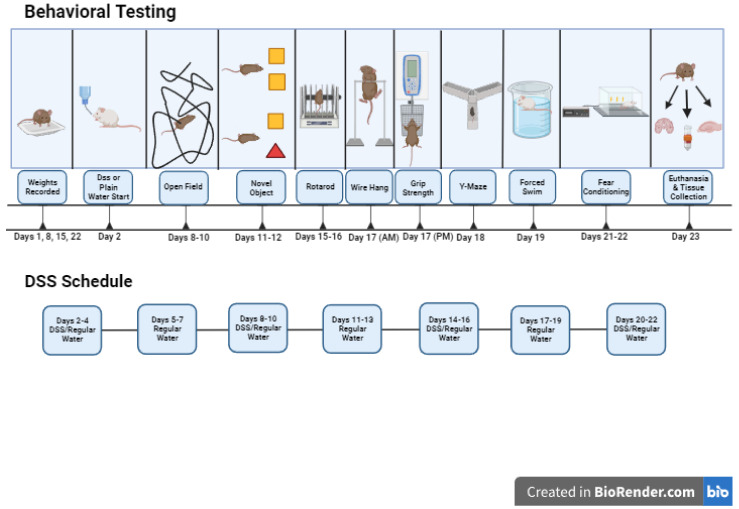
Schematic outline of study 4.

In studies 1 and 3, mice were treated with PQ at 10 mg/kg, twice per week (Mondays and Fridays) for 3 weeks, as described [38]. Behavioral testing started the week after the last PQ or saline injection. Study 1 involved 83 female and male mice (A53T: *n* = 37 mice, A53T-L444P: *n* = 46 mice, PQ: *n* = 41 mice, saline: *n* = 42 mice, *n* = 46 male mice, *n* = 37 female mice). The ages at the start of the treatments were 7.31 ± 0.32 months for the A53T mice and 8.06 ± 0.29 months for A53T-L444P mice. In study 2, mice were only irradiated (whole body X-ray irradiation at 4 Gy) or sham-irradiated. Due to logistical schedule-related limitations, we only included behavioral tests we anticipated to be most sensitive to effects of whole-body X-ray irradiation based on previous studies. Study 2 involved 73 female and male mice (A53T: *n* = 43 mice, A53T-L444P: *n* = 30 mice, Sham: *n* = 37 mice, radiation: *n* = 36 mice, *n* = 42 male mice, *n* = 31 female mice). The ages at the start of the treatments were 4.74 ± 0.09 months for the A53T mice and 4.77 ± 0.07 months for A53T-L444P mice. In study 3, mice were also irradiated (whole body X-ray irradiation (Rad Source RS2000 Biological Research Irradiator, Suwanee, GA, USA) at 4 Gy) or sham-irradiated on day 1 of week 1. Study 3 involved 66 female and male mice (A53T: *n* = 39 mice, A53T-L444P: *n* = 27 mice, control: *n* = 34 mice, PQ + IR: *n* = 32 mice, *n* = 38 male mice, *n* = 28 female mice). The ages at the start of the treatments were 8.62 ± 0.31 months for the A53T mice and 8.51 ± 0.40 months for A53T-L444P mice. There were no adverse effects of X-ray irradiation on the general health of the mice noted in studies 2 or 3. In study 4, mice were treated with 3% DSS (40–45 kDa, TdB Labs, Uppsala, Sweden) in the drinking water for 4 weeks, using a 3 days on/3 days off treatment paradigm, and tested during this 4-week period, as the effects of DSS are transient. The DSS solution was prepared every three days. Study 4 involved 91 female and male mice (A53T: *n* = 42 mice, A53T-L444P: *n* = 49 mice, H_2_O: *n* = 47 mice, DSS: *n* = 44 mice, *n* = 47 male mice, *n* = 44 female mice). The ages at the start of the treatments were 4.77 ± 0.17 months for the A53T mice and 4.55 ± 0.15 months for A53T-L444P mice. The experimental design of the four studies, including the days of weight recording, is outlined in Figure 1, Figure 2, Figure 3 and Figure 4. The detailed behavioral tests are described below.

### 2.3. Open Field and Novel Object Recognition 

The mice were tested in an open field enclosure (16 × 16 inches, Kinder Scientific, Poway, CA, USA) for 10 min on three subsequent days. In studies 1 and 4, fecal boli were collected in the open field for 16S analysis of the gut microbiome. On day 4, the open field contained two identical objects for a 15-min trial. The next day, one object was replaced with a novel object for a 15-min trial. Between trials, the arenas and objects were cleaned with 0.5% acetic acid. Interaction within a 2 cm proximity with the object was coded as object exploration by hand-scoring videos acquired with Noldus Ethovision software (version 17, Wageningen, The Netherlands). A discrimination index, defined as the time spent exploring the familiar object, was subtracted from the time exploring the novel object, and the resulting number was divided by the total time spent exploring both objects and was analyzed.

The outcome measures in the open field analyzed were: (1) distance moved in the open field in the absence and presence of objects, an activity measure; (2) the difference in the distance moved in the open field over days, habituation to the open field, a cognitive measure; (3) time spent in the center of the open field, an anxiety measure; and (4) the discrimination index, a cognitive measure.

### 2.4. Rotarod

The rotarod test (rod diameter: 3 cm, elevated: 45 cm, Rotamex-5, Columbus Instruments, Columbus, OH, USA) was used to assess sensorimotor function. The rotation speed started at 5 rpm and accelerated by 1.0 rpm every 3 s. Fall latency (s) was recorded. Mice received three subsequent trials on two subsequent days. The outcome measure used in the rotarod test was the mean fall latency of each mouse for each day.

### 2.5. Wire Hang

The wire hang task, as described by van Putten [39], was also used to assess motor function. The outcome measures in the wire hang test were fall and reach scores.

### 2.6. Grip Strength

A Harvard Apparatus (Holliston, MA, USA) grip strength meter for mice was positioned horizontally. The mice were allowed to grasp the metal grid and pulled backwards in the horizontal plane. The force applied to the grid was recorded as the peak tension. Three measurements were conducted at one-minute intervals [40]. The peak grip strength for each mouse was recorded. In addition, we calculated the relative grip strength (the ratio of grip strength to body weight) [41]. The outcome measures in the grip strength test were the peak grip strength and the ratio of grip strength to body weight.

### 2.7. Y-Maze

Activity levels and spontaneous alternations were assessed in the Y-maze (O’ Hara & Co., Ltd., Tokyo, Japan) in a 5 min trial. The maze was cleaned with 0.5% acetic acid between trials. Performance was recorded using Noldus Ethovision video tracking. Hand scoring was used to assess the number of arm entries and the percentage of spontaneous alternations. The outcome measures in the Y-maze were total arm entries, an activity measure, and percentage of spontaneous alternations, a cognitive measure.

### 2.8. Contextual and Cued Fear Conditioning 

Contextual and cued fear conditioning were assessed (Med Associates mouse fear conditioning system (PMED-VFC-NIR-M, Med Associates, St. Albans, Vermont and Med Associates VideoFreeze automated scoring system). When mice were placed inside the fear conditioning chamber, chamber lights were on at the beginning of the trial. There was a 90 s baseline habituation period, two 30 s tones (80 dB) co-terminating with a 2 s 0.5 mA shock, and separated by a 90 s inter-stimulus interval (ISI). The next day, the mice were placed into the same chamber for a 5 min trial with the chamber lights on to assess contextual fear memory. The chambers were cleaned with 0.5% acetic acid.

Two hours later, the mice were put in a novel environment (a novel floor texture, angled walls and vanilla-extract-soaked nestlet fixed to the outside of the wall) to assess cued fear memory. Following a 90-s baseline period, there was a 180-s period during which the same tone as present during training was played. Between cued trials, the chambers were cleaned with a 10% isopropanol solution. The outcome measures in the fear conditioning test were: (1) activity levels prior to the first tone; (2) freezing during the tone, a measure of fear learning; (3) response to the shock; (4) freezing during the intervals between tone-shocks, a measure of fear learning; (5) activity levels during the tones; (6) activity levels during the intervals between the tone-shocks; (7) percent freezing in the hippocampus-dependent contextual fear memory test; (8) percent freezing during the pre-tone and during the tone in the hippocamps-independent cued fear memory test; (9) activity levels in the contextual fear memory test; (10) activity levels in the cued fear memory test.

### 2.9. Forced Swim Test

To assess depressive-like behavior, mice were placed for 6 min in a container with water (water height: 15 cm, container diameter: 16–20 cm, 25 °C), not allowing the mouse’s tail to touch the bottom. Immobility, defined as cessation of limb movements except minor movements to stay afloat, was scored manually by a blinded observer using a sampling technique every 5 s during the trial. The outcome measure in the forced swim test was the percentage immobility (number of immobility observations divided by the total number of observations), a measure of depressive-like behavior, during the last 4 min (48 observations) of the test, as described [42].

### 2.10. 16S Gut Microbiome Analysis

16S rRNA gene sequence data was generated from fecal boli collected from mice in studies 1 and 4, following our prior work [43]. The fecal boli were carefully collected in individual tubes, cleaning the forceps used to collect the samples well between samples, and immediately stored at −80 °C to preserve the quality of the samples. They were subsequently shipped on dry ice to OSU for analysis. Cleaned amplicons were pooled at equimolar concentration and subject to DNA sequencing (Illumina MiSeq in the OSU Center for Quantitative Life Sciences). Sequence data was demultiplexed, adapter trimmed, and subject to amplicon sequence variant (ASV) clustering using the DADA2 workflow in the R programming environment. Prior to data analysis, ASV libraries were subject to rarefaction to normalize for variation in sequencing depth. For the DSS study, samples were rarefied to a uniform depth of 90,000 reads per sample, and for the PQ study, samples were rarefied to a uniform depth of 17,102 reads per sample.

We evaluated how the microbiome alpha-diversity and beta-diversity associate with genotype, treatment, sex, and behavioral physiological covariates. Alpha-diversity reflects how many different taxa there are in the community and summarizes ecological characteristics of the community. Beta-diversity reflects the composition of the microbiome (which specific taxa reside in a community) and how the taxonomic composition of the community differs from that of other communities. We assessed whether the types of taxa that comprise a microbiome vary as a function of different treatment (e.g., PQ or DSS). For both alpha- and beta-diversity, we evaluated a variety of metrics that summarize these microbiome properties but differ in mathematical form.

Our analysis of the gut microbiome data followed our prior work [43]. We used hypothesis tests or linear regression to associate alpha-diversity with study covariates and a step-wise model construction framework that zeroes-in on the set of covariates, their potential interaction, and significantly explains the variation in alpha-diversity across samples (*p* < 0.05). We used the Bray–Curtis dissimilarity (unless otherwise specified) to compare microbiome community composition across samples and applied PERMANOVA analyses to determine if study- or sample-specific covariates significantly explain the variation in community composition across samples. Negative binomial regression models were used to determine the relative abundance of specific taxa associated with covariates. Statistical tests were corrected for multiple tests by controlling the false discovery rate (fdr) at an alpha of 0.05.

For study 1, relationships between the gut microbiome and the following behavioral measures were assessed: grip strength (GS); distance moved in the open field on day 1 (OF1 TotDist), day 2 (OF2 TotDist), and day 3 (OF3 TotDist); percent spontaneous alternation in the Y-maze (YM.Pct.Spon.Alt); activity levels during the baseline period during fear learning (FC.train_base); freezing during the first tone during fear learning (FC.train_frz_tone1); freezing during the second tone during fear learning (FC.train_frz_tone2); and freezing during the contextual fear memory test (FC.context_totalfrzing) (see Table S1 PQ_sample_data).

For study 4, relationships between the gut microbiome and the following behavioral measures were assessed: distance moved in the open field on day 1 (OF1 TotDist); time spent in the center of the open field on day 1 (OF1.CentDur); difference in the distance moved on day 1 and 3 (OFdelta.dm.d3); distance on the first day of the open field test containing objects (NO1.SOTotDistMove.cm); distance 0 on the second day of the open field test containing objects (NO2.SOTotDistMove.cm); percentage of time spent exploring the novel object during the object recognition test (NO2.DO.PCT.Obj2); discrimination index in the object recognition test (NO2.DO.DI); total arm entries in the Y-maze (Y.Maze,Total.Arm.Entries); spontaneous alternation in the Y-maze (Y.Maze.Pct.Spont.Alt); improvement in rotorod performance (Delta.rotorod); fall score and reach score in the wire hang test (FallScore, Reachscore); grip strength (Grip.Strength.g); body weight during the grip strength test (Grip.Wk3.weight.g); grip strength divided by body weight (Grip.Ratio); percent immobility in the forced swim test (FST.scoring); percent freezing during the contextual fear memory test (ContextFC_ContTotPctFrz); and percent freezing during the tone in the cued fear memory test (Context.FC.CuedTnePrctFrz).

### 2.11. Statistical Analyses Behavioral Data

All behavioral data are reported as the mean ± standard error of the mean and were analyzed using SPSS v.22 (IBM, Armonk, NY, USA) or GraphPad v.8 (La Jolla, CA, USA) software. For each of the four studies, genotype (A53T-L444P or A53T), treatment (PQ or saline, radiation or sham-irradiation, and DSS or water), and sex were included as factors in analysis of variance (ANOVAs). In case of statistical interactions, genotypes and/or sexes were analyzed separately, as indicated. Unless sex or an interaction with sex was significant or showed a trend, we did not indicate that in the results. Repeated measures were used when appropriate. Statistical significance was considered as *p* < 0.05. When sphericity was violated (Mauchly’s test), Greenhouse–Geisser corrections were used. For each study, mice were tested in separate cohorts, each containing mice of all experimental groups. All researchers were blinded to genotype and treatment and the code was only broken after the data were analyzed.

## 3. Results

### 3.1. Study 1

#### 3.1.1. Body Weights

All groups lost body weight over the three weeks of treatment but there was no effect of genotype or treatment on the percent body weight change (Figure S1A). There was an effect of sex with females losing more percent body weights than males (*F* (1,75) = 4.311, *p* = 0.041).

#### 3.1.2. Open Field and Novel Object Recognition

**Figure 5:**
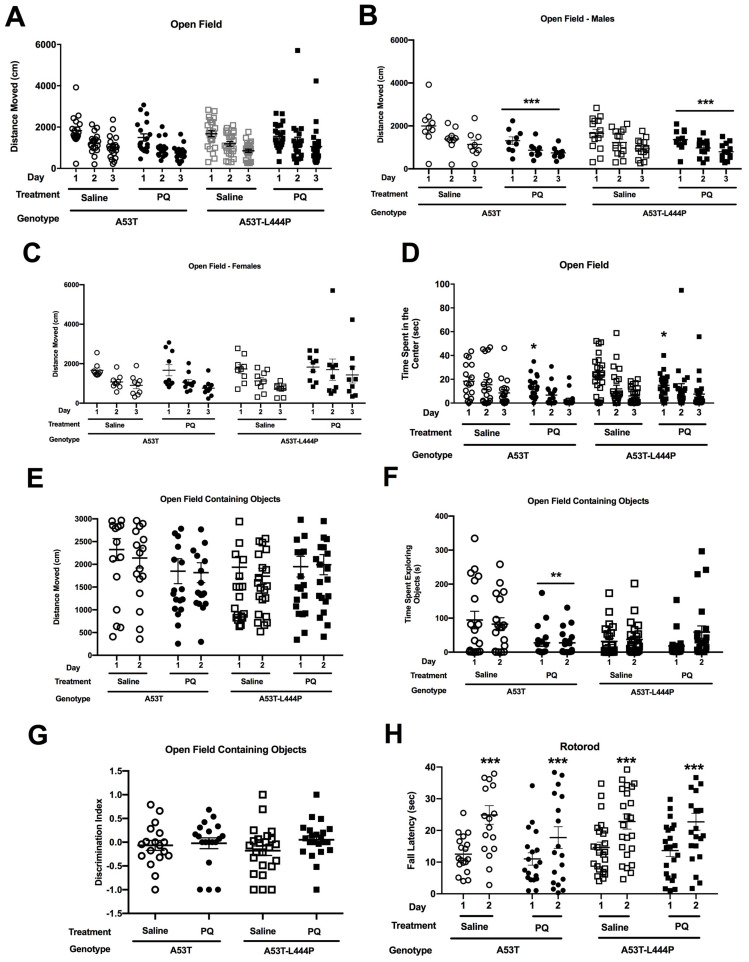
(**A**) For all figures, in cases that there was a main effect of sex or a statistical interaction with sex, female and male data were analyzed separately. All groups showed spatial habituation learning and moved less in the open field over the three subsequent days (*F* (1,75) = 84.686, *p* < 0.001). As there was a treatment × sex interaction (*F* (1,75) = 6.152, *p* = 0.015), we also analyzed the male and female data separately. (**B**) In males, there was an effect of day (*F* (1,42) = 93.550, *p* < 0.001) and an effect of treatment (*F* (1,42) = 93.550, *** *p* < 0.001). PQ-treated mice moved less than saline-treated mice. (**C**) In females, there was only an effect of day (*F* (2,66) = 15.320, *p* < 0.001). (**D**) For time spent in the more anxiety-provoking center of the open field, there was an effect of day (*F* (1,4) = 42.179, *p* < 0.001) and an effect of treatment (*F* (1,74) = 4.205, *p* = 0.044). When only the day 1 data were analyzed, there was an effect of treatment, with PQ-treated mice spending less time in the center of the open field on day 1 (*F* (1,74) = 6.091, * *p* = 0.016). (**E**) In the two days of the open field containing objects, there was no effect of genotype or treatment. There was no preferential exploration of the novel object in any of the groups. (**F**) For time spent exploring the objects, there was an effect of treatment (*F* (1,75) = 4.809, *p* = 0.031) and a treatment × genotype interaction (*F* (1,74) = 7.098, *p* = 0.009). When only A53T mice were analyzed, there was an effect of treatment (*F* (1,33) = 7.619, ** *p* = 0.009). PQ-treated A53T mice spent less time exploring the objects than saline-treated A53T mice. (**G**) For the discrimination index, there were no effects of genotype or treatment. None of the groups showed a discrimination index different from 0 and therefore no preferential exploration of the novel object. (**H**) When sensorimotor function was assessed in the rotorod test, there was only an effect of day (*F*(1,75) = 65.927, *** *p* < 0.001), with better performance on day 2 than day 1. Circles: A53T; squares: A53T-L444P. Open symbols: saline; filled symbols: PQ.

All groups showed spatial habituation learning and moved less in the open field over the three subsequent days (*F* (1,75) = 84.686, *p* < 0.001) (Figure 5A). There was a treatment × sex interaction (*F* (1,75) = 6.152, *p* = 0.015) and a trend towards a treatment × genotype interaction (*F* (1,75) = 2.986, *p* = 0.088). In males, there was an effect of day (*F* (1,42) = 93.550, *p* < 0.001) and an effect of treatment (*F* (1,42) = 93.550, *p* < 0.001) (Figure 5B). In females, there was only an effect of day (*F* (2,66) = 15.320, *p* < 0.001) (Figure 5C).

Next, we assessed the time spent in the more anxiety-provoking center of the open field. There was an effect of day (*F* (1,4) = 42.179, *p* < 0.001), with mice spending less time in the center on subsequent days, an effect of treatment (*F* (1,74) = 4.205, *p* = 0.044), a trend towards a treatment × day interaction (*F* (1,74) = 3.902, *p* = 0.052), and a trend towards a treatment × sex interaction (*F* (1,74) = 2.869, *p* = 0.095) (Figure 5D).

As measures of anxiety in the open field are easier to interpret on day 1, we also analyzed only the day 1 data. There was an effect of treatment, with PQ-treated mice spending less time in the center of the open field on day 1 (*F* (1,74) = 6.091, *p* = 0.016) (Figure 5D).

In the two days of the open field containing objects, there was a trend towards a treatment × sex interaction (*F* (1,75) = 3.791, *p* = 0.055) for activity levels (Figure 5E). For time spent exploring the objects, there was an effect of treatment (*F* (1,75) = 4.809, *p* = 0.031), a treatment × genotype interaction (*F* (1,74) = 7.098, *p* = 0.009), and a trend towards an effect of genotype (*F* (1,74) = 3.545, *p* = 0.064), day × treatment interaction (*F* (1,74) = 2.993, *p* = 0.088), and a day × genotype interaction (*F* (1,74) = 3.962, *p* = 0.050) (Figure 5F). When only A53T mice were analyzed, there was an effect of treatment (*F* (1,33) = 7.619, *p* = 0.009). When only the A53T-L444P mice were analyzed, there was an effect of day (*F* (1,42) = 5.176, *p* = 0.028) and a trend towards a day × treatment interaction (*F* (1,42) = 3.504, *p* = 0.068).

For the discrimination index, there were no effects of genotype, treatment, or sex (Figure 5G). In none of the groups was there a significant preferential exploration of the novel object.

#### 3.1.3. Rotorod

When sensorimotor function was assessed in the rotorod test, there was only an effect of day (*F*(1,75) = 65.927, *p* < 0.001), with better performance on day 2 than day 1 (Figure 5H). When the difference in rotorod performance for each mouse was calculated, there was a trend towards a genotype × treatment interaction (*F*(1,71) = 2.797, *p* = 0.099).

#### 3.1.4. Wire Hang

There was no effect of genotype or treatment on fall (Figure S1B) or reach (Figure S1C) scores in the wire hang test.

#### 3.1.5. Y-Maze

**Figure 6:**
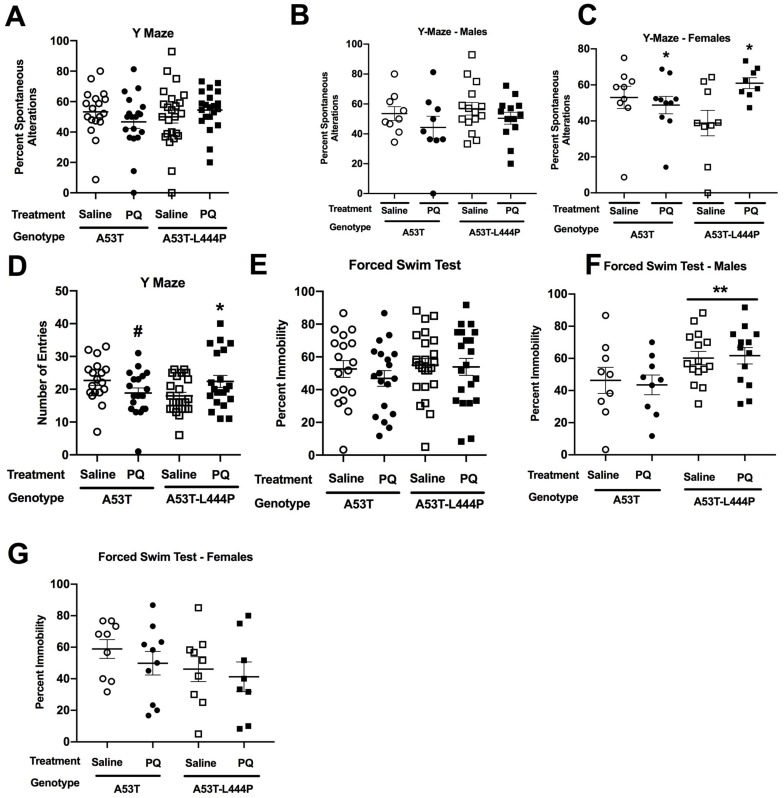
(**A**) For spontaneous alternation in the Y-maze, there a treatment × sex interaction (*F*(1,74) = 4.975, *p* = 0.029). (**B**) In males, there was a treatment × genotype interaction (*F*(1,32) = 5.578, *p* = 0.024). When each genotype was analyzed separately, there was no effect of treatment in either. (**C**) In females, there was an effect of treatment (*F*(1,15) = 7.602, * *p* = 0.015). This effect seemed driven by a higher percent spontaneous alternation in PQ- than saline-treated A53T-L444P mice. (**D**) For arm entries in the Y-maze, there was a treatment × genotype interaction (*F*(1,74) = 10.681, *p* = 0.002). In A53T mice, there was a trend towards an effect of treatment (*F*(1,33) = 3.842, ^#^ *p* = 0.058). In A53T-L444P mice, there was an effect of treatment (*F*(1,41) = 7.224, * *p* = 0.010). PQ-treated A53T-L444P mice were more active than saline-treated A53T-L444P mice. (**E**) There was no effect of genotype or treatment in depressive-like behavior in the forced swim test but there was a sex × genotype interaction (*F*(1,74) = 8.111, *p* = 0.006). (**F**) There was an effect of genotype in males (*F*(1,42) = 7.836, ** *p* = 0.0077). There was more depressive-like behavior in A53T-L444P than A53T mice. (**G**) There was no effect of genotype in females. Circles: A53T; squares: A53T-L444P. Open symbols: saline; filled symbols: PQ.

For spontaneous alternation in the Y-maze, there was a treatment × sex interaction (*F*(1,74) = 4.975, *p* = 0.029) and a trend towards a genotype × treatment interaction (*F*(1,74) = 3.778, *p* = 0.056) (Figure 6A). When only males were analyzed, there was a treatment × genotype interaction (*F*(1,32) = 5.578, *p* = 0.024) (Figure 6B), but when each male genotype group was analyzed separately, there was no effect of treatment in either. When only females were analyzed, there was an effect of treatment (*F*(1,15) = 7.602, *p* = 0.015) (Figure 6C). However, the directional effect of PQ on spontaneous alternation in the Y-maze in female mice was genotype dependent. While PQ decreased spontaneous alternation in A53T female mice, it increased spontaneous alternation in A53T-L444P female mice (Figure 6C). The genotype-dependent effect of PQ on spontaneous alternation in female mice might relate to the spontaneous alternation levels in saline-treated female mice, which were higher in A53T than A53T-L444P female mice.

Arm entries were analyzed as an activity measure in the Y-maze. There was an effect of sex (*F*(1,75) = 12.736, *p* = 0.001), a treatment × genotype interaction (*F*(1,74) = 10.681, *p* = 0.002), and a trend towards a treatment × sex interaction (*F*(1,75) = 3.869, *p* = 0.053) (Figure 6D). When only A53T mice were analyzed, there was an effect of sex (*F*(1,33) = 5.482, *p* = 0.025) and a trend towards an effect of treatment (*F*(1,33) = 3.842, *p* = 0.058). When only A53T-L444P mice were analyzed, there was an effect of treatment (*F*(1,41) = 7.224, *p* = 0.010), and a trend towards a treatment × sex interaction (*F*(1,75) = 3.537, *p* = 0.067).

#### 3.1.6. Grip Strength

In the grip strength test, there was an effect of sex (*F*(1,48) = 5.715, *p* = 0.021) and a trend towards a treatment × sex × genotype interaction (*F*(1,48) = 3.336, *p* = 0.074) for grip strength (Figure S1D). When grip strength per body weight was analyzed, there was no effect of genotype, treatment, or sex (Figure S1E).

#### 3.1.7. Forced Swim Test

When depressive-like behavior was assessed in the forced swim test, there was a sex × genotype interaction (*F*(1,74) = 8.111, *p* = 0.006) (Figure 6E). In A53T mice, there was no effect of treatment, genotype, or sex. In A53T-L444P mice, there was an effect of sex (*F*(1,41) = 7.562, *p* = 0.009), with higher percent immobility in males (Figure 6F) than females (Figure 6G). In addition, there was an effect of genotype in males (*F*(1,42) = 7.836, *p* = 0.0077). This was not seen in females.

#### 3.1.8. Fear Conditioning

**Figure 7:**
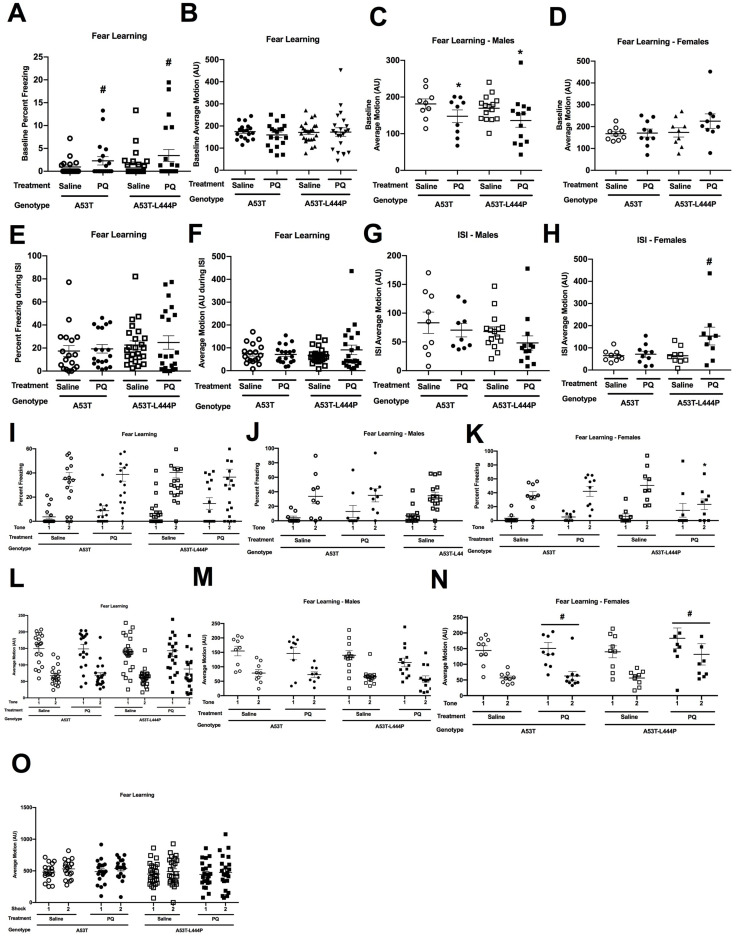
(**A**) During the baseline period on the training day, there was a trend towards higher freezing levels in PQ- than saline-treated mice (*F*(1,75) = 2.856, ^#^ *p* = 0.095) on the percent freezing. (**B**) When activity levels during the baseline period on the training day were analyzed, there was a treatment × sex interaction (*F*(1,75) = 5.475, *p* = 0.022). (**C**) In males, there was an effect of treatment (*F*(1,42) = 4.928, * *p* = 0.032). Activity levels were lower in PQ- than saline-treated mice. (**D**) In females, there was no effect of treatment. (**E**) There was no effect of genotype or treatment on the freezing levels during the ISI. (**F**) During the ISI, there was a treatment × sex interaction (*F*(1,75) = 6.853, *p* = 0.011) and a genotype × sex interaction (*F*(1,75) = 5.956, *p* = 0.017). (**G**) In males, there were no significant effects or trend towards treatment effects. (**H**) In A53T-L444P female mice, there was a trend towards an effect of treatment (*F*(1,16) = 4.408, ^#^ *p* = 0.052). (**I**) When freezing during the tones was analyzed, there was a tone × sex × genotype × treatment interaction (*F*(1,75) = 6.612, *p* = 0.012). In A53T mice, there was only an effect of tone (*F*(1,33) = 83.234, *p* < 0.001). In A53T-L444P mice, there was a tone × sex × treatment interaction (*F*(1,42) = 7.478, *p* = 0.009) and a tone × treatment interaction (*F*(1,75) = 5.093, *p* = 0.029). In A53T-L444P mice during tone 2, there was a treatment × sex interaction (*F*(1,42) = 6.160, *p* = 0.017). (**J**) In A53T-L444P male mice, there was no effect on percent freezing during tone 2. (**K**) In A53T-L444P female mice, there was an effect of treatment in females (*F*(1,16) = 5.674, * *p* = 0.030). Freezing levels were lower in PQ- than saline-treated mice. (**L**) When activity levels during the tones were analyzed, there was a treatment × sex interaction (*F*(1,75) = 4.199, *p* = 0.044). (**M**) There was no effect of treatment or trend towards an effect of treatment on activity levels during the tones. (**N**) In females, there was a trend towards an effect of treatment (*F*(1,33) = 3.067, ^#^ *p* = 0.089) on activity levels during the tones. (**O**) There was no effect of genotype or treatment on responsiveness to the shocks. Circles: A53T; squares: A53T-L444P. Open symbols: saline; filled symbols: PQ.

During the baseline period on the training day, prior to the first tone, there was a trend towards an effect of treatment (*F*(1,75) = 2.856, *p* = 0.095) on the percent freezing, with higher freezing levels in PQ- than saline-treated mice (Figure 7A). When activity levels during the baseline period on the training day were analyzed, there was a treatment × sex interaction (*F*(1,75) = 5.475, *p* = 0.022) and a trend towards an effect of sex (*F*(1,75) = 3.891, *p* = 0.052) (Figure 7B). When activity levels during the baseline period on the training day were analyzed in males, there was an effect of treatment (*F*(1,42) = 4.928, *p* = 0.032) (Figure 7C); in contrast, there was no effect of treatment in females (Figure 7D).

During the ISI, there was a trend towards a genotype × treatment × sex interaction for the percent freezing (*F*(1,75) = 3.112, *p* = 0.082) (Figure 7E). When activity levels during the ISI were analyzed, there was a treatment × sex interaction (*F*(1,75) = 6.853, *p* = 0.011), a genotype × sex interaction (*F*(1,75) = 5.956, *p* = 0.017), trends towards a treatment × genotype × sex interaction (*F*(1,75) = 3.341, *p* = 0.072), and an effect of sex (*F*(1,75) = 2.976, *p* = 0.089) (Figure 7F). When activity levels in only A53T-L444P mice were analyzed, there was an effect of sex (*F*(1,42) = 7.119, *p* = 0.011), a treatment × sex interaction (*F*(1,42) = 8.109, *p* = 0.007), and a trend towards an effect of treatment (*F*(1,42) = 3.265, *p* = 0.078). In contrast, no significance or trends were seen in A53T mice. When activity levels only in A53T-L444P female mice were analyzed, there was a trend towards an effect of treatment (*F*(1,16) = 4.408, *p* = 0.052) (Figure 7H). This was not seen in A53T-L444P male mice (Figure 7G).

When freezing during the tones was analyzed, there was a tone × sex × genotype × treatment interaction (*F*(1,75) = 6.612, *p* = 0.012) and a trend towards a tone × treatment interaction (*F*(1,75) = 3.128, *p* = 0.081). When only A53T mice were analyzed, there was only an effect of tone (*F*(1,33) = 83.234, *p* < 0.001). When only A53T-L444P mice were analyzed, there was a tone × sex × treatment interaction (*F*(1,42) = 7.478, *p* = 0.009) and a tone × treatment interaction (*F*(1,75) = 5.093, *p* = 0.029). When in A53T-L444P mice freezing only during tone 1 was analyzed, there were no significant effects. When in A53T-L444P mice freezing only during tone 2 was analyzed, there was a treatment × sex interaction (*F*(1,42) = 6.160, *p* = 0.017). When A53T-L444P mice freezing only during tone 2 was analyzed in either males or females, there was an effect of treatment in females (*F*(1,16) = 5.674, *p* = 0.030) (Figure 7K), but not males (Figure 7J). When A53T-L444P male mice freezing during both tones was analyzed, there was only an effect of tone (*F*(1,26) = 40.996, *p* < 0.001). When A53T-L444P female mice freezing during both tones was analyzed, there was a tone × treatment interaction (*F*(1,16) = 10.921, *p* = 0.004) and an effect of tone (*F*(1,16) = 23.958, *p* < 0.001).

When activity levels during the tones were analyzed, there was a treatment × sex interaction (*F*(1,75) = 4.199, *p* = 0.044) and a trend towards a sex × genotype interaction (*F*(1,75) = 3.839, *p* = 0.054) (Figure 7L). When activity levels during the tones in only females were analyzed, there was a trend towards an effect of treatment (*F*(1,33) = 3.067, *p* = 0.089) (Figure 7N). This was not seen in males (Figure 7M). When response to the shocks was analyzed, there was an effect of shock (*F*(1,75) = 4.979, *p* = 0.029) (Figure 7O). The following day, contextual fear memory was assessed.

**Figure 8:**
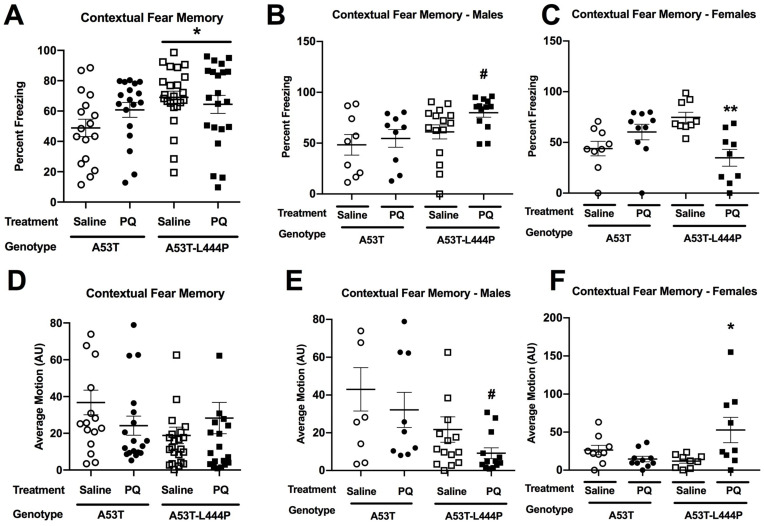

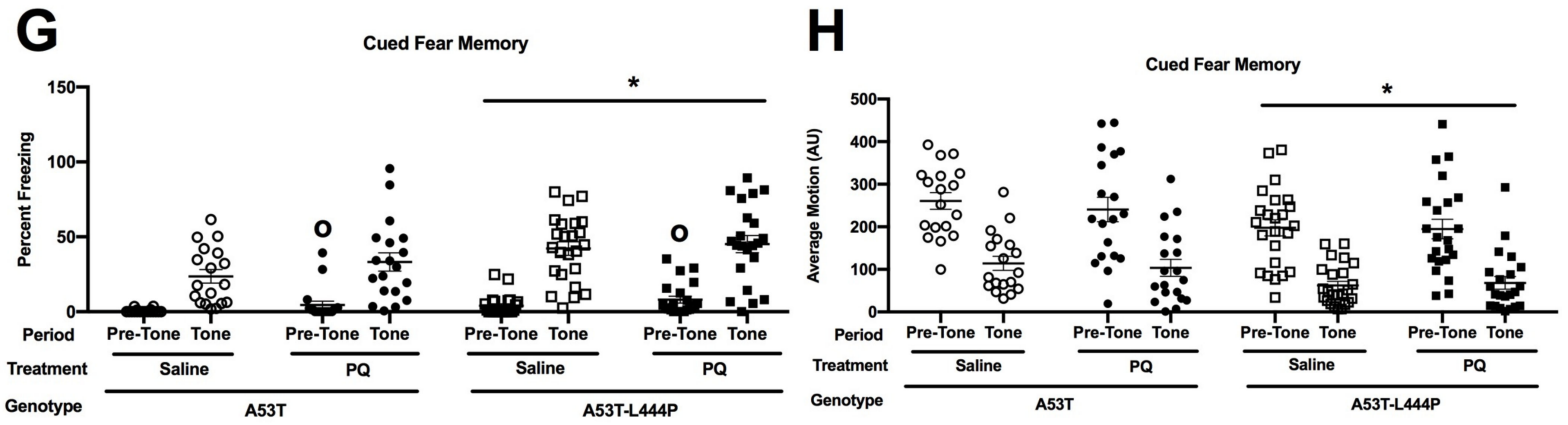
(**A**) There was an effect of genotype on percent freezing in the contextual fear memory test (*F*(1,71) = 4.448, * *p* = 0.038). Contextual fear memory was stronger in A53T-L444P than A53T mice. There was also a genotype × treatment interaction (*F*(1,71) = 5.573, *p* = 0.021), a genotype × sex interaction (*F*(1,71) = 5.597, *p* = 0.021), a treatment × sex interaction (*F*(1,71) = 4.178, *p* = 0.045), and a genotype × treatment × sex interaction (*F*(1,71) = 10.463, *p* = 0.002). (**B**) In A53T-L444P male mice, there was a trend towards an effect of treatment (*F*(1,25) = 3.866, ^#^ *p* = 0.060). (**C**) In A53T-L444P female mice, there was an effect of treatment (*F*(1,75) = 14.725, * *p* = 0.002), with reduced freezing in PQ-treated female mice. ** *p* < 0.01. (**D**) When activity levels were analyzed in the contextual fear memory test, there was a genotype × treatment interaction (*F*(1,71) = 6.067, *p* = 0.016), a genotype × sex interaction (*F*(1,75) = 8.423, *p* = 0.005), a treatment × sex interaction (*F*(1,71) = 6.307, *p* = 0.014), and a genotype × treatment × sex interaction (*F*(1,75) = 7.482, *p* = 0.008). (**E**) In A53T-L444P males, there was a trend towards an effect of treatment (*F*(1,25) = 3.263, ^#^ *p* = 0.083). (**F**) In A53T-L444P females, there was an effect of treatment (*F*(1,15) = 8.201, * *p* = 0.012). Activity levels were higher in PQ- than saline-treated mice. (**G**) In the cued fear memory test, there was an effect of genotype (*F*(1,75) = 9.391, * *p* = 0.003), with higher freezing levels in A53T-L444P than A53T mice. There was also a period × genotype interaction (*F*(1,75) = 6.537, *p* = 0.013). During the pre-tone period, there was an effect of genotype (*F*(1,75) = 4.381, *p* = 0.040), with higher freezing levels in A53T-L444P mice, and treatment (*F*(1,75) = 4.353, ^o^ *p* = 0.040), with higher freezing levels in PQ- than saline-treated mice. When percent freezing was analyzed during the tone, there was only an effect of genotype (*F*(1,75) = 8.885, *p* = 0.004), with higher freezing in A53T-L444P than A53T mice. (**H**) When activity levels during the pre-tone and tone periods were analyzed in the cued fear memory test, there was an effect of genotype (*F*(1,75) = 5.871, * *p* = 0.018), with lower activity levels in A53T-L444P than A53T mice. There was also an effect of period (*F*(1,75) = 272.918, *p* < 0.001), with lower activity during the tone than pre-tone period. When activity levels during the tone in the cued fear memory test were analyzed, there was an effect of genotype (*F*(1,75) = 8.667, *p* = 0.004), with lower activity levels in A53T-L444P than A53T mice. Circles: A53T; squares: A53T-L444P. Open symbols: saline; filled symbols: PQ.

When percent freezing was assessed in the contextual fear memory test, there was an effect of genotype (*F*(1,71) = 4.448, *p* = 0.038), a genotype × treatment interaction (*F*(1,71) = 5.573, *p* = 0.021), a genotype × sex interaction (*F*(1,71) = 5.597, *p* = 0.021), a treatment × sex interaction (*F*(1,71) = 4.178, *p* = 0.045), and a genotype × treatment × sex interaction (*F*(1,71) = 10.463, *p* = 0.002) (Figure 8A). When freezing was only analyzed in A53T-L444P mice, there was an effect of sex (*F*(1,71) = 7.071, *p* = 0.011) and a treatment × sex interaction (*F*(1,71) = 17.804, *p* < 0.001). Significant effects were not seen in A53T mice. When percent freezing was only analyzed in A53T-L444P male mice, there was a trend towards an effect of treatment (*F*(1,25) = 3.866, *p* = 0.060), with a trend towards increased freezing in PQ-treated male mice (Figure 8B). When percent freezing was only analyzed in A53T-L444P female mice, there was an effect of treatment (*F*(1,75) = 14.725, *p* = 0.002), with reduced freezing in PQ-treated female mice (Figure 8C).

When activity levels were analyzed in the contextual fear memory test, there was a genotype × treatment interaction (*F*(1,71) = 6.067, *p* = 0.016), a genotype × sex interaction (*F*(1,75) = 8.423, *p* = 0.005), a treatment × sex interaction (*F*(1,71) = 6.307, *p* = 0.014), and a genotype × treatment × sex interaction (*F*(1,75) = 7.482, *p* = 0.008) (Figure 8D). When activity levels were analyzed in A53T-L444P mice, there was an effect of treatment (*F*(1,40) = 4.179, *p* = 0.048), an effect of sex (*F*(1,40) = 5.690, *p* = 0.022), and a treatment × sex interaction (*F*(1,40) = 14.225, *p* = 0.001). When activity levels were analyzed in only A53T mice, there was only a trend towards an effect of sex (*F*(1,31) = 3.082, *p* = 0.089). When activity levels were analyzed in only A53T-L444P females, there was an effect of treatment (*F*(1,15) = 8.201, *p* = 0.012) (Figure 8F). When activity levels were analyzed in only A53T-L444P males, there was only a trend towards an effect of treatment (*F*(1,25) = 3.263, *p* = 0.083) (Figure 8E).

Finally, cued fear memory was assessed. When percent freezing was assessed in the cued fear memory test, there was an effect of genotype (*F*(1,75) = 9.391, *p* = 0.003), with higher freezing levels in A53T-L444P than A53T mice, and a period × genotype interaction (*F*(1,75) = 6.537, *p* = 0.013) (Figure 8G). When percent freezing was only analyzed in the pre-tone period, there were effects of genotype (*F*(1,75) = 4.381, *p* = 0.040), with higher freezing levels in A53T-L444P mice, and treatment (*F*(1,75) = 4.353, *p* = 0.040), with higher freezing levels in PQ- than saline-treated mice. When percent freezing was analyzed during the tone, there was only an effect of genotype (*F*(1,75) = 8.885, *p* = 0.004), with higher freezing in A53T-L444P than A53T mice.

When activity levels during the pre-tone and tone periods were analyzed in the cued fear memory test, there was an effect of genotype (*F*(1,75) = 5.871, *p* = 0.018), with lower activity levels in A53T-L444P than A53T mice, and an effect of period (*F*(1,75) = 272.918, *p* < 0.001), with lower activity during the tone than pre-tone period (Figure 8H). When activity levels during the tone in the cued fear memory test were analyzed, there was an effect of genotype (*F*(1,75) = 8.667, *p* = 0.004), with lower activity levels in A53T-L444P than A53T mice, and a trend towards a genotype × treatment × sex interaction (*F*(1,75) = 3.026, *p* = 0.086).

### 3.2. Study 2

#### 3.2.1. Body Weights

There was no effect of genotype or treatment on the percentage body weight change (Figure S2A).

#### 3.2.2. Open Field and Novel Object Recognition

There was a trend towards spatial habituation with mice moving less on day 2 than day 1 in the open field (*F*(1,29) = 3.502, *p* = 0.071) (Figure S2B), but no effect of genotype or treatment. When time spent in the center of the open field was analyzed, there was an effect of day (*F*(1,29) = 6.309, *p* = 0.018), with mice spending less time in the center of the open field on day 2 than day 1 (Figure S2C), but no effect of genotype or treatment.

When activity levels were analyzed in the two days of open field containing objects, there was a trend towards a genotype × treatment × day interaction (*F*(1,29) = 4.132, *p* = 0.051) (Figure S2D). While activity levels were comparable on days 1 and 2 in the open field containing objects in irradiated A53T and sham-irradiated A53T-L444P mice, activity levels were lower on day 2 than day 1 in sham-irradiated A53T and irradiated A53T-L444P mice. There was no effect of genotype or treatment on time spent exploring the objects (Figure S2E). There was no effect of genotype or treatment on the discrimination index, but the pattern indicated that while sham-irradiated A53T and irradiated A53T-L444P mice showed a preference for exploring the familiar object, irradiated A53T and sham-irradiated A53T-L444P mice did not show a preference (Figure S2F).

#### 3.2.3. Grip Strength

There were no effects of genotype or treatment on grip strength (Figure S2G) or grip strength per body weight (Figure S2H).

#### 3.2.4. Wire Hang

There was no effect of genotype or treatment on fall scores in the wire hang test (Figure S2H) but an effect of sex (*F*(1,29) = 6.759, *p* = 0.015) with lower fall scores in females than males. There was no effect of genotype or treatment on fall scores of females or males in the wire hang test.

There was a trend towards an effect of genotype for reach scores (*F*(1,29) = 3.271, *p* = 0.081), with a trend towards higher reach scores in A53T than A53T-L444P mice (Figure S2I). There was also a trend towards an effect of genotype for latency to first reach (*F*(1,29) = 4.108, *p* = 0.052), with lower reach scores in A53T than A53T-L444P mice (Figure S2J).

### 3.3. Study 3

#### 3.3.1. Body Weights

All groups lost body weight over the three weeks of treatment but there was no effect of genotype or treatment on the percent body weight change (Figure S3A).

#### 3.3.2. Open Field and Novel Object Recognition

**Figure 9:**
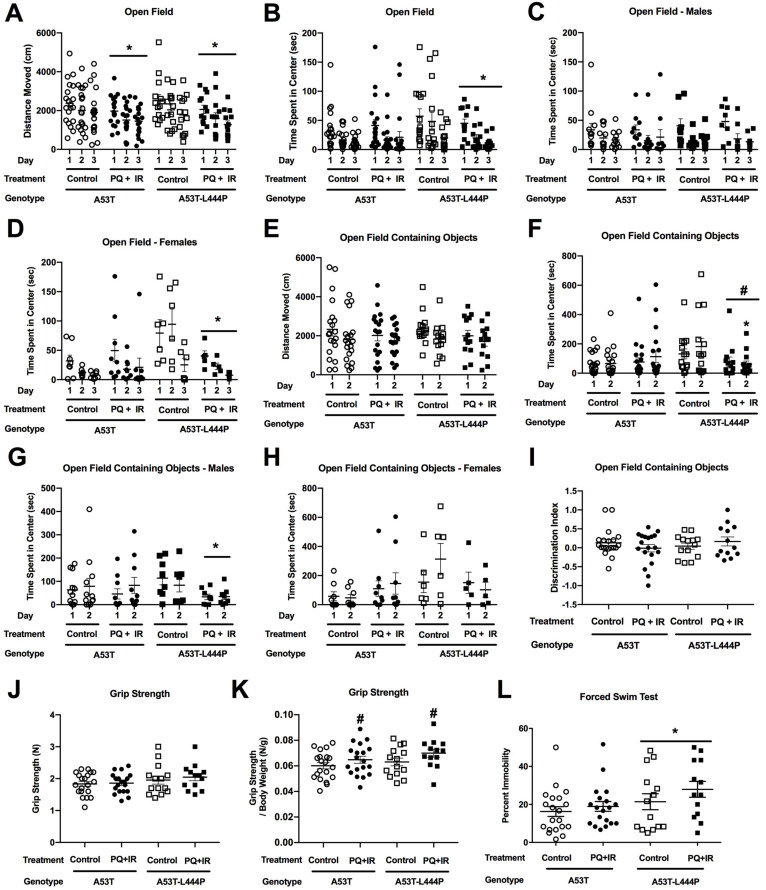
(**A**) PQ + IR-treated mice moved less than control mice in the open field (*F*(1,58) = 4.668, * *p* = 0.035). (**B**) There was also a genotype × treatment interaction (*F*(1,58) = 4.784, *p* = 0.033), and a sex × genotype × treatment interaction (*F*(1,58) = 5.826, *p* = 0.019) for time spent in the more anxiety-provoking center of the open field. In A53T-L444P mice, anxiety levels were higher in PQ + IR- than control mice. (**C**) There was no effect of genotype or treatment on measures of anxiety in the open field in male mice. (**D**) In A53T-L444P mice, PQ + IR-treated female mice spent less time in the center than control-treated female mice (*F*(1,23) = 4.825, * *p* = 0.038). (**E**) There was no effect of genotype or treatment on activity levels in the open field containing objects. (**F**) For time spent in the center of the open field where the objects were, there was an effect of sex (*F*(1,58) = 5.385, *p* = 0.024), a treatment × genotype interaction (*F*(1,58) = 4.221, *p* = 0.044), a day × treatment × sex interaction (*F*(1,58) = 4.692, *p* = 0.034), a day × treatment × genotype interaction (*F*(1,58) = 6.167, *p* = 0.016), and a day × treatment × sex × genotype interaction (*F*(1,58) = 7.233, *p* = 0.009). In A53T-L444P mice, there was a trend towards an effect of treatment over the two days of testing in the open field containing objects (*F*(1,23) = 3.241, ^#^ *p* = 0.085) and an effect of treatment on day 2 (*F*(1,23) = 5.498, * *p* = 0.028). (**G**) A53T-L444P male mice were analyzed, and there was an effect of treatment (*F*(1,14) = 5.297, ^#^ *p* = 0.037). (**H**) In A53T-L444P female mice, there was a day × treatment interaction (*F*(1,9) = 6.989, *p* = 0.027) but not an effect of treatment on either day. (**I**) There was no effect of genotype or treatment on the discrimination index. (**J**) There was no effect of genotype or treatment on grip strength. (**K**) There was a trend towards a higher grip strength per body weight in mice that received PQ + IR grip strength (*F*(1,58) = 3.329, ^#^ *p* = 0.073). (**L**) In the forced swim test, there was more depressive-like behavior in A53T-L444P than A53T mice (*F*(1,58) = 4.669, * *p* = 0.035). Circles: A53T; squares: A53T-L444P. Open symbols: control; filled symbols: PQ + IR.

There was spatial habituation, with mice moving less in the open field on subsequent days (*F*(1,58) = 50.774, *p* < 0.001) and an effect of treatment (*F*(1,58) = 4.668, *p* = 0.035) (Figure 9A), but no effect of genotype. There were also trends towards an effect of sex (*F*(1,58) = 3.090, *p* = 0.084), a day × sex interaction (*F*(1,58) = 3.833, *p* = 0.055), and a trend towards a day × genotype interaction (*F*(1,58) = 3.055, *p* = 0.086).

When time spent in the center of the open field was analyzed, there was an effect of day (*F*(1,58) = 29.831, *p* < 0.001), with mice spending less time in the center of the open field on day 2 than day 1 (Figure 9B). There was also a genotype × treatment interaction (*F*(1,58) = 4.784, *p* = 0.033), a sex × genotype × treatment interaction (*F*(1,58) = 5.826, *p* = 0.019), and a trend towards an effect of genotype (*F*(1,58) = 3.802, *p* = 0.056). When only A53T mice were analyzed, there was an effect of day (*F*(1,35) = 8.394, *p* = 0.006). When only A53T-L444P mice were analyzed, there were effects of day (*F*(1,23) = 26.810, *p* < 0.001), treatment (*F*(1,23) = 4.825, *p* = 0.038), sex (*F*(1,23) = 4.776, *p* = 0.039), and sex × treatment interaction (*F*(1,23) = 6.228, *p* = 0.020).

When only A53T-L444P male mice were analyzed, there was only an effect of day (*F*(1,14) = 22.746, *p* < 0.001) (Figure 9C). However, when only A53T-L444P female mice were analyzed, there was an effect of day (*F*(1,9) = 8.826, *p* = 0.016) and an effect of treatment (*F*(1,9) = 6.279, *p* = 0.034) (Figure 9D).

In the open field containing objects, there was an effect of day on activity levels (*F*(1,58) = 24.068, *p* < 0.001) and a trend towards a day × sex interaction (*F*(1,58) = 2.817, *p* = 0.099) (Figure 8E). For time spent in the center of the open field where the objects were, there was an effect of sex (*F*(1,58) = 5.385, *p* = 0.024), a treatment × genotype interaction (*F*(1,58) = 4.221, *p* = 0.044), a day × treatment × sex interaction (*F*(1,58) = 4.692, *p* = 0.034), a day × treatment × genotype interaction (*F*(1,58) = 6.167, *p* = 0.016), a day × treatment × sex × genotype interaction (*F*(1,58) = 7.233, *p* = 0.009), and a trend towards a day × sex × genotype interaction (*F*(1,58) = 2.863, *p* = 0.096) (Figure 9F). When only the A53T mice were analyzed, there were no significant effects. When only the A53T-L444P mice were analyzed, there was an effect of sex (*F*(1,23) = 5.678, *p* = 0.026), a day × treatment interaction (*F*(1,23) = 5.968, *p* = 0.023), a day × treatment × sex interaction (*F*(1,23) = 11.044, *p* = 0.003), a trend towards an effect of treatment (*F*(1,23) = 3.241, *p* = 0.085), and a trend towards a day × sex interaction (*F*(1,23) = 3.730, *p* = 0.066). When only A53T-L444P male mice were analyzed, there was an effect of treatment (*F*(1,14) = 5.297, *p* = 0.037) (Figure 9G). When only A53T-L444P female mice were analyzed, there was a day × treatment interaction (*F*(1,9) = 6.989, *p* = 0.027), but not an effect of treatment on either day (Figure 9H). No significant effects were seen on day 1. When only day 1 in the open field with the two objects was analyzed, there was a trend towards an effect of sex (*F*(1,58) = 3.922, *p* = 0.052). When only day 2 in the open field with the two objects was analyzed, there was an effect of sex (*F*(1,58) = 5.276, *p* = 0.025), a treatment × genotype interaction (*F*(1,58) = 6.469, *p* = 0.014), and trends towards a sex × genotype interaction (*F*(1,58) = 3.492, *p* = 0.067) and a treatment × sex × genotype interaction (*F*(1,58) = 3.238, *p* = 0.077). When day 2 in the open field with the objects in only the A53T mice were analyzed, there were no significant effects. When day 2 in the open field in only the A53T-L444P mice were analyzed, there were effects of treatment (*F*(1,23) = 5.498, *p* = 0.028) and sex (*F*(1,23) = 7.195, *p* = 0.013).

For the discrimination index, there was a trend towards a genotype × treatment interaction (*F*(1,51) = 3.997, *p* = 0.051) and a trend towards a sex × genotype × treatment interaction (*F*(1,51) = 3.126, *p* = 0.083) (Figure 9I).

#### 3.3.3. Wire Hang

There was an effect of sex for fall scores in the wire hang test (*F*(1,58) = 8.345, *p* = 0.005) (Figure S3B), with higher fall scores in females (Figure S3D) than males (Figure S3C). For reach scores, there was a trend towards a treatment × sex × genotype interaction (*F*(1,58) = 3.248, *p* = 0.077) (Figure S3E).

#### 3.3.4. Grip Strength

There was an effect of sex for grip strength (*F*(1,58) = 7.700, *p* = 0.007) (Figure 9J). There was a trend towards an effect of treatment for grip strength per body weight (*F*(1,58) = 3.329, *p* = 0.073), with a higher mean grip strength in mice that received PQ and irradiation (Figure 9K).

#### 3.3.5. Y-Maze

There was no effect of genotype or treatment for spontaneous alternation in the Y-maze (Figure S3F). There was a trend towards an effect of sex for the number of entries (*F*(1,58) = 3.949, *p* = 0.052) (Figure S3G).

#### 3.3.6. Forced Swim Test

In the forced swim test, there was an effect of genotype on percent immobility (*F*(1,58) = 4.669, *p* = 0.035), with higher depressive-like behavior in A53T-L444P than A53T mice (Figure 9L).

#### 3.3.7. Fear Conditioning

**Figure 10:**
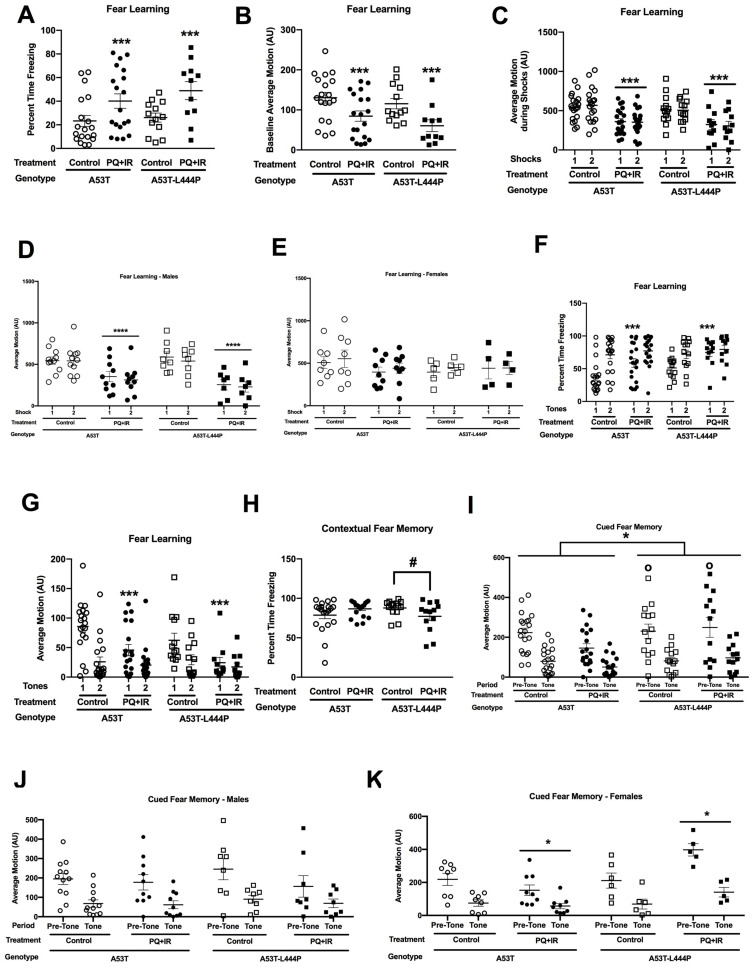
(**A**) During the baseline period on the training day, freezing levels were higher in the PQ + IR- than control-treated mice (*F*(1,54) = 9.216, *** *p* = 0.004). (**B**) During the baseline period on the training day, activity levels were lower in the PQ + IR- than control-treated mice (*F*(1,54) = 9.845, *** *p* = 0.003). (**C**) There was an effect of treatment (*F*(1,58) = 11.898, *** *p* = 0.001) and a treatment × genotype interaction (*F*(1,58) = 5.944, *p* = 0.018) for response to the shocks. The response to shock was lower in PQ + IR than control mice. (**D**) In males, PQ + IR-treated mice showed a lower response to the shocks than control-treated mice (*F*(1,32) = 23.416, **** *p* < 0.001). (**E**) In females, there was no effect of genotype or treatment on response to the shocks. (**F**) PQ + IR-treated mice froze more during the first tone (*F*(1,54) = 9.986, *** *p* = 0.003). (**G**) PQ + IR-treated mice moved less during the first tone (*F*(1,54) = 9.275, *** *p* = 0.004). (**H**) When freezing during the contextual fear memory test was analyzed, there was a treatment × genotype interaction (*F*(1,58) = 4.037, *p* = 0.049). In A53T-L444P mice, there was a trend towards lower freezing levels in IR + PQ -treated than control-treated mice (*F*(1,23) = 3.780, ^#^ *p* = 0.064). (**I**) When activity levels were analyzed in the cued fear memory test, there was an effect of genotype (*F*(1,58) = 4.604, * *p* = 0.036). This effect seemed driven by higher activity levels in PQ + IR-treated A53T-L444P than A53T mice. There were also a treatment × genotype × sex interaction (*F*(1,58) = 6.048, *p* = 0.017) and a period × treatment × genotype × sex interaction (*F*(1,58) = 6.874, *p* = 0.011). In A53T mice, there was only an effect of period (*F*(1,35) = 126.536, *p* < 0.001). In A53T-L444P mice, there was a treatment × sex interaction (*F*(1,23) = 6.233, *p* = 0.020), a period × treatment × sex interaction (*F*(1,23) = 5.265, *p* = 0.031), and trends towards an effect of sex (*F*(1,58) = 3.040, *p* = 0.095) and a period × sex interaction (*F*(1,58) = 3.914, *p* = 0.060). During the pre-tone period, activity levels were higher in A53T-L44P than A53T mice (*F*(1,58) = 4.816, ^o^ *p* = 0.032). (**J**) In males, there was only an effect of period. (**K**) When activity levels were only analyzed in A53T-L444P females, there was as effect of treatment (*F*(1,9) = 8.163, * *p* = 0.019), with lower activity levels in IR + PQ- than control-treated A53T mice and higher activity levels in IR + PQ- than control-treated A53T-L444P mice. Circles: A53T; squares: A53T-L444P. Open symbols: control; filled symbols: PQ + IR.

During the baseline period on the training day, there was an effect of treatment on the percent freezing (*F*(1,54) = 9.216, *p* = 0.004), with higher freezing levels in the combined PQ + radiation- than control-treated mice (Figure 10A). When activity levels during the baseline period were analyzed, there also was an effect of treatment (*F*(1,54) = 9.845, *p* = 0.003) (Figure 10B).

When response to the shocks was analyzed, there was an effect of treatment (*F*(1,58) = 11.898, *p* = 0.001) and a treatment × genotype interaction (*F*(1,58) = 5.944, *p* = 0.018) (Figure 10C). When response to the shocks in only female mice were analyzed, there were no significant effects (Figure 9E). When response to the shocks in only male mice were analyzed, there was an effect of treatment (*F*(1,32) = 23.416, *p* < 0.001) (Figure 10D).

For freezing during the tones, there was an effect of treatment (*F*(1,54) = 4.807, *p* = 0.033), an effect of tone (*F*(1,54) = 54.991, *p* < 0.001), a tone × treatment interaction (*F*(1,58) = 8.421, *p* = 0.005), and a tone × genotype interaction (*F*(1,54) = 6.484, *p* = 0.014) (Figure 10F). When only freezing during tone 1 was analyzed, there were effects of treatment (*F*(1,54) = 9.986, *p* = 0.003) and genotype (*F*(1,54) = 5.911, *p* = 0.018). When only freezing during tone 2 was analyzed, there were no significant effects.

When activity levels during the tones were analyzed, there was an effect of treatment (*F*(1,54) = 5.338, *p* = 0.025), a period × treatment interaction (*F*(1,54) = 10.230, *p* = 0.002), a period × genotype interaction (*F*(1,54) = 8.247, *p* = 0.006), and a trend towards a tone × treatment × genotype × sex interaction (*F*(1,54) = 3.479, *p* = 0.068) (Figure 10G). When activity levels during only tone 1 were analyzed, there were effects of treatment (*F*(1,54) = 9.275, *p* = 0.004) and genotype (*F*(1,54) = 4.719, *p* = 0.034), and a trend towards a treatment × sex × genotype interaction (*F*(1,54) = 3.589, *p* = 0.064). When activity levels during only tone 2 were analyzed, there were no significant effects.

For freezing levels during the ISI, there was an effect of period (*F*(1,54) = 67.698, *p* < 0.001) (Figure S4A). When activity levels during the ISI were analyzed, there was an effect of ISI (*F*(1,58) = 23.153, *p* < 0.001) (Figure S4B).

When freezing during the contextual fear memory test was analyzed, there was a treatment × genotype interaction (*F*(1,58) = 4.037, *p* = 0.049) (Figure 10H). There were no effects of treatment in A53T mice, and there was a trend towards an effect of treatment in A53T-L444P mice (*F*(1,23) = 3.780, *p* = 0.064). When activity levels during the contextual fear memory test were analyzed, there were no significant effects (Figure S4C).

In the cued fear memory test, there was an effect of period (*F*(1,58) = 225.302, *p* < 0.001) for percent freezing (Figure S4D). When activity levels were analyzed in the cued fear memory test, there was an effect of genotype (*F*(1,58) = 4.604, *p* = 0.036), a treatment × genotype × sex interaction (*F*(1,58) = 6.048, *p* = 0.017), a period × treatment × genotype × sex interaction (*F*(1,58) = 6.874, *p* = 0.011), and trends towards a treatment × sex interaction (*F*(1,58) = 3.114, *p* = 0.083), a period × genotype interaction (*F*(1,58) = 3.641, *p* = 0.061), a period × sex interaction (*F*(1,58) = 3.415, *p* = 0.070), a period × treatment × sex interaction (*F*(1,58) = 3.047, *p* = 0.086), and a period × genotype × sex interaction (*F*(1,58) = 3.678, *p* = 0.060) (Figure 10I). When activity levels were only analyzed in A53T mice, there was only an effect of period (*F*(1,35) = 126.536, *p* < 0.001). When activity levels were only analyzed in A53T-L444P mice, there was a treatment × sex interaction (*F*(1,23) = 6.233, *p* = 0.020), a period × treatment × sex interaction (*F*(1,23) = 5.265, *p* = 0.031), and trends towards an effect of sex (*F*(1,58) = 3.040, *p* = 0.095) and a period × sex interaction (*F*(1,58) = 3.914, *p* = 0.060).

When activity levels were only analyzed in A53T-L444P females, there was as effect of treatment (*F*(1,9) = 8.163, *p* = 0.019) and a trend towards a period × treatment interaction (*F*(1,9) = 4.621, *p* = 0.060) (Figure 10K). When activity levels were only analyzed in A53T-L444P males, there was only an effect of period (*F*(1,14) = 19.981, *p* < 0.001) (Figure 10J). When activity levels were analyzed only during the pre-tone period, there were effects of genotype (*F*(1,58) = 4.816, *p* = 0.032) and a treatment × sex × genotype interaction (*F*(1,58) = 7.097, *p* = 0.010), and trends towards an effect of sex (*F*(1,58) = 2.890, *p* = 0.094), a treatment × sex (*F*(1,58) = 3.479, *p* = 0.067), and a sex × genotype interaction (*F*(1,58) = 2.917, *p* = 0.093). When activity levels during the pre-tone period were only analyzed in A53T mice, there were no significant effects. In contrast, in A53T-L444P mice, there was a treatment × sex interaction (*F*(1,23) = 6.555, *p* = 0.017) and a trend towards an effect of sex (*F*(1,23) = 3.711, *p* = 0.066). When activity levels during the pre-tone period were only analyzed in A53T-L444P females, there was an effect of treatment (*F*(1,9) = 9.353, *p* = 0.014); in contrast, this was not seen in A53T-L444P males. When activity levels during the tone were analyzed, there were trends towards an effect of genotype (*F*(1,58) = 2.961, *p* = 0.091) and a treatment × sex × genotype interaction (*F*(1,58) = 2.836, *p* = 0.098).

### 3.4. Study 4

#### 3.4.1. Body Weights

All groups lost body weight over the three weeks of treatment but there was no effect of genotype or treatment on the percent body weight change (Figure S5A).

#### 3.4.2. Open Field and Novel Object Recognition

**Figure 11:**
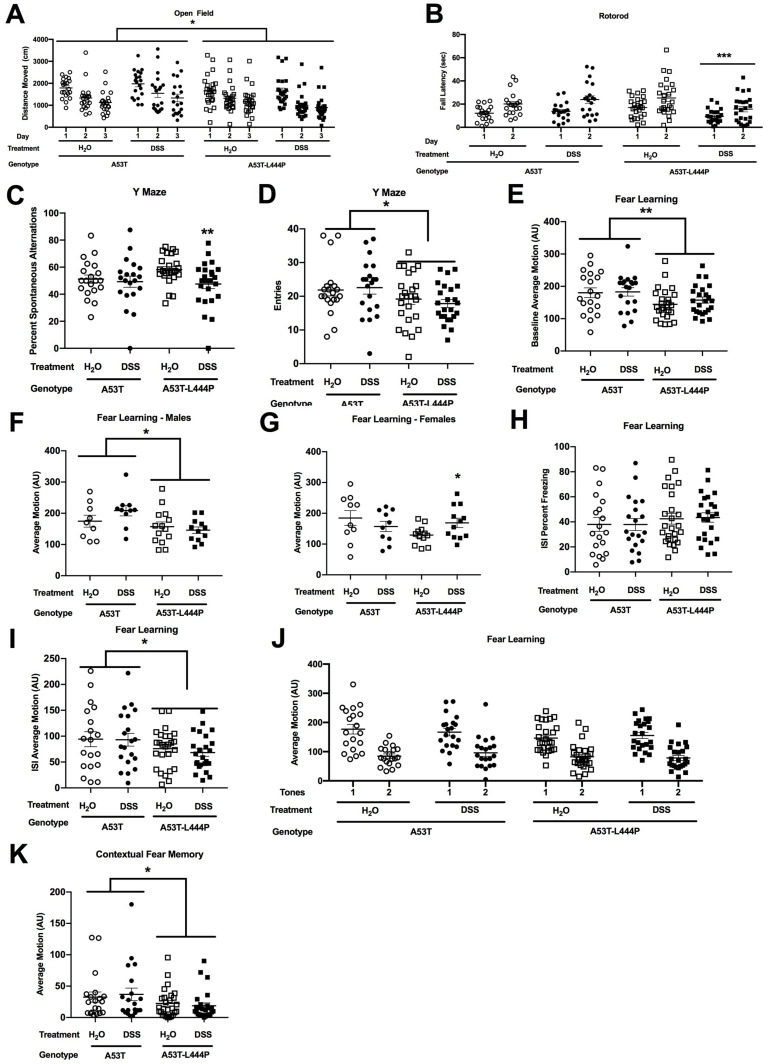
(**A**) In the open field, activity levels were higher in A53T than A54T-L444P mice (*F*(1,81) = 4.091, * *p* = 0.046). (**B**) In the rotorod test, there was a genotype × treatment interaction (*F*(1,81) = 7.773, *p* = 0.007). DSS impaired rotorod performance in A53T-L444P mice (*F*(1,45) = 9.228, *** *p* = 0.004), but not in A53T mice. (**C**) DSS reduced spontaneous alternation in A53T-L444P mice (*F*(1,45) = 7.191, * *p* = 0.01). (**D**) A53T-L44P mice showed reduced activity levels in the Y-maze (*F*(1,81) = 5.336, * *p* = 0.023). (**E**) During the baseline period in the fear conditioning test, activity levels were lower in A53T-L44P than A53T mice (*F*(1,81) = 7.565, ** *p* = 0.007). (**F**) In males, A53T-L44P mice showed lower activity levels than A53T mice during the baseline period (*F*(1,41) = 6.727, * *p* = 0.013). (**G**) In females, there was a treatment × genotype interaction (*F*(1,40) = 4.168, *p* = 0.048). DSS increased activity level in A53T-L44P females (*F*(1,22) = 5.119, * *p* = 0.034). (**H**) For the freezing levels during the ISI, there was a genotype × treatment × sex interaction (*F*(1,81) = 4.661, *p* = 0.034). (**I**) A53T-L44P mice had lower activity levels than A53T mice during the ISI (*F*(1,81) = 4.564, * *p* = 0.036). (**J**) When activity levels during the tones were analyzed, there was a genotype × treatment × sex interaction (*F*(1,81) = 8.199, *p* = 0.005). (**K**) A53T-L44P mice had lower activity levels than A53T mice during the contextual fear memory test (*F*(1,85) = 4.677, * *p* = 0.033). Circles: A53T; squares: A53T-L444P. Open symbols: H_2_O; filled symbols: DSS.

When activity levels in the open field were analyzed, all groups showed habituation to the open field (*F*(1.3,107.6) = 5.419, *p* = 0.014) (Figure 11A). There was also an effect of genotype (*F*(1,81) = 4.091, *p* = 0.046), with higher activity levels in A53T than A54T-L444P mice. In addition, there was a trend towards a genotype × treatment interaction (*F*(1,81) = 2.987, *p* = 0.088).

When measures of anxiety were assessed by analyzing the time spent in the center of the open field, there was only an effect of day (*F*(1.6,128.6) = 20.75, *p* < 0.001), with mice spending less time in the center of the open field on subsequent days, but there was no effect of genotype or treatment (Figure S5B).

When activity levels in the open field containing objects was analyzed, there was an effect of day (*F*(1,81) = 23.65, *p* < 0.001), with mice moving less on the second than first day of the open field containing objects (Figure S5C). There was no effect of genotype or treatment.

There was no effect of genotype or treatment for time spent exploring the two objects in the object recognition test (Figure S5D). There was no overall effect of genotype or treatment on the discrimination index, but DSS-treated A53T mice did not show a discrimination index different from 0 (i.e., no preference), while water-treated A53T mice (*t* = 3.860, *p* = 0.0006) and water- (*t* = 2.645, *p* = 0.011) and DSS- (*t* = 2.896, *p* = 0.0059) treated A53T-L444P mice did (Figure S5E). Consistent with this, there was a trend towards an effect of treatment in A53T mice (*t* = 1.839, *p* = 0.0750 (2-tailed *t*-test).

#### 3.4.3. Rotorod

In the rotorod test, there was an effect of day (*F*(1,81) = 65.0, *p* < 0.001), with mice performing better on the second than first day of the rotorod test (Figure 11B). There was also a genotype × treatment interaction (*F*(1,81) = 7.773, *p* = 0.007). When the two genotypes were analyzed separately, DSS impaired rotorod performance in A53T-L444P mice (*F*(1,45) = 9.228, *p* = 0.004), but not in A53T mice.

#### 3.4.4. Wire Hang

There was no effect of genotype or treatment on fall scores (Figure S5F) or reach scores (Figure S5G) in the wire hang test.

#### 3.4.5. Grip Strength

There were no effects of genotype or treatment on performance in grip strength test but there was an effect of sex (*F*(1,85) = 8.203, *p* = 0.005) (Figure S5H). There were no effects of genotype, treatment, or sex for grip strength per body weight (Figure S5I).

#### 3.4.6. Y Maze

When spontaneous alternation was assessed in the Y maze, there was an effect of treatment (*F*(1,80) = 4.181, *p* = 0.044), with reduced spontaneous alternation in DSS-treated mice (Figure 11C). This effect was clearly driven by an effect of treatment in A53T-L444P mice (*F*(1,45) = 7.191, *p* = 0.01). There was no effect of treatment in A53T mice when analyzed separately.

When the number of entries was analyzed as an activity measure, there was an effect of genotype (*F*(1,81) = 5.336, *p* = 0.023), with lower activity levels in A53T-L444P than A53T mice (Figure 11D).

#### 3.4.7. Forced Swim Test 

There were no effects of genotype or treatment on performance in the forced swim test (Figure S5J).

#### 3.4.8. Fear Conditioning

There were no effects of genotype or treatment on percent freezing during the baseline period (Figure S6A). When activity levels during the baseline period were analyzed, there was an effect of genotype (*F*(1,81) = 7.565, *p* = 0.007) and a treatment × genotype × sex interaction (*F*(1,81) = 6.176, *p* = 0.015) (Figure 11E). When activity levels during the baseline period were analyzed in only A53T mice, there were no significant effects. In A53T-L444P mice, there was a trend towards a treatment × sex interaction (*F*(1,46) = 3.860, *p* = 0.056). When activity levels during the baseline period were only analyzed in females, there was a treatment × genotype interaction (*F*(1,40) = 4.168, *p* = 0.048) (Figure 11G). In males, there was an effect of genotype for activity levels during the baseline period (*F*(1,41) = 6.727, *p* = 0.013) (Figure 11F). In A53T females, there were no significant effects but there was an effect of treatment in A53T-L44P females (*F*(1,22) = 5.119, *p* = 0.034) (Figure 11H).

For the freezing levels during the ISI, there was a genotype × treatment × sex interaction (*F*(1,81) = 4.661, *p* = 0.034). When percent freezing during the ISI was only analyzed in A53T mice, there were no significant effects. In A53T-L444P mice, there was a trend towards a treatment × sex interaction (*F*(1,46) = 2.950, *p* = 0.093). When percent freezing during the ISI was analyzed in only females, there were no significant effects. When percent freezing during the ISI was analyzed in only males, there was a trend towards a treatment × genotype interaction (*F*(1,41) = 2.904, *p* = 0.096). For activity levels during the ISI, there was an effect of genotype (*F*(1,81) = 4.564, *p* = 0.036) and a trend towards a treatment × genotype × sex interaction (*F*(1,80) = 3.152, *p* = 0.080) (Figure 11I).

When freezing during the tones was analyzed, there were no significant effects (Figure S6B). When activity levels during the tones were analyzed, there was a genotype × treatment × sex interaction (*F*(1,81) = 8.199, *p* = 0.005) and a trend towards an effect of genotype (*F*(1,81) = 2.822, *p* = 0.097) (Figure 11J). When activity levels during the tones were only analyzed in females, there was a genotype × treatment interaction (*F*(1,40) = 5.561, *p* = 0.023) and a period × genotype × treatment interaction (*F*(1,40) = 5.017, *p* = 0.031). In A53T females, there was a tone × treatment interaction (*F*(1,18) = 5.315, *p* = 0.033). In A53T-L444P females, there was only a trend towards an effect of treatment (*F*(1,22) = 4.138, *p* = 0.054). In A53T females during tone 1, there was a trend towards an effect of treatment (*F*(1,18) = 3.644, *p* = 0.072). This was not seen during tone 2. When activity levels during the tones were only analyzed in males, there was only a trend towards a genotype × treatment interaction (*F*(1,41) = 3.142, *p* = 0.084).

When response to the shocks was analyzed, there were trends towards a treatment × genotype × sex interaction (*F*(1,80) = 3.606, *p* = 0.061) and a period × sex interaction (*F*(1,80) = 2.929, *p* = 0.091) (Figure S6C).

During the contextual fear memory test, there was a trend towards an effect of genotype for percent freezing (*F*(1,85) = 3.424, *p* = 0.068) (Figure S6D). When activity levels were analyzed during the contextual fear memory test, there was an effect of genotype (*F*(1,85) = 4.677, *p* = 0.033) and trends towards an effect of sex (*F*(1,85) = 3.317, *p* = 0.072) and a genotype × sex interaction (*F*(1,85) = 3.905, *p* = 0.051) (Figure 11K).

When freezing levels were analyzed in the cued fear memory test, there was a period × sex interaction (*F*(1,85) = 4.729, *p* = 0.032) and a trend towards an effect of sex (*F*(1,85) = 3.177, *p* = 0.078) (Figure S6E). When freezing levels during the pre-tone period only were analyzed, there were no significant effects. During the tone period, there was a trend towards an effect of sex (*F*(1,85) = 3.952, *p* = 0.050). When activity levels in the cued fear memory test were analyzed, there were no significant effects (Figure S6F).

For a summary of the genotype and treatment effects in the four studies, see also Table 1.

### 3.5. Microbiome Results—Study 1

**Figure 12:**
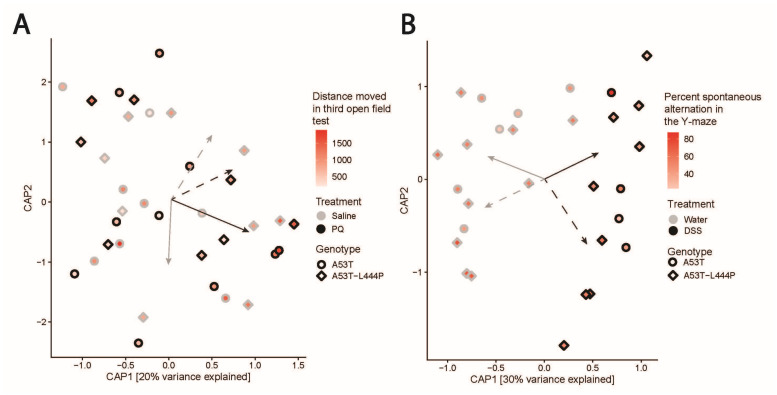
Capscale ordinations reveal genotypic and exposure dependent associations between the composition of the gut microbiome and behavior measures. (**A**) A constrained ordination illustrates the association between the taxonomic beta-diversity of the gut microbiome, as measured by the weighted unifrac metric, and PQ exposure, genotype, and the total distance moved during the third day of an open field test. (**B**) An analogous ordination illustrating the association between the taxonomic beta-diversity of the gut microbiome, as measured by the Bray–Curtis dissimilarity metric, and PQ exposure, genotype, and the total distance moved during the third day of an open field test. For both panels, arrows indicate the direction in ordination space that corresponds to the vector of association between beta-diversity and the behavioral covariate (i.e., arrowheads point towards samples with higher behavioral scores) for a given set of sample sets: control treatment and control genotype (solid grey lines), control treatment and L444P genotype (dashed grey lines), chemical exposure and control genotype (solid black lines), and chemical exposure and L444P genotype (dashed black lines). The different directionality of vectors indicates interaction effects of these various features on the association between microbiome composition and behavior, and these differences are supported by permanova tests (*p* < 0.05).

We also analyzed the microbiomes of the PQ-exposed and saline-treated mice in the aforementioned PQ-exposure study (Figure 12). Stool was collected from the mice during the second week of behavioral testing, frozen, and delivered to the OSU Microbiome Core for microbiome processing as described above (see Tables S1 and S2 for the PQ taxon and metadata, respectively). Overall, the effect of PQ on the gut microbiome was relatively modest: mice exposed to PQ exposure did not manifest a robust difference in the composition of their gut microbiome as compared to saline-treated mice (*p* > 0.05). Robust sex effects on the gut microbiome were observed across mice (*p* = 0.008) and there were no independent genotype dependent effects on the gut microbiome (*p* > 0.05). That said, mouse behavior linked to the taxonomic composition of the microbiome in ways that were influenced by PQ exposure. An open field test of exploratory behavior (total distance day 3) found that this behavior was also linked to the taxonomic composition of the gut microbiome, but that this association differed as a function of PQ treatment (*p* = 0.049, Figure 12A). Further, mouse behavior associated with gut microbiome composition in a genotype-dependent manner. Namely, the open field test of exploratory behavior (total distance day 3) linked to the composition of the gut microbiome differently as a function of the two genotypes (*p* = 0.034, Figure 12A). Additionally, several gut taxa strongly linked to behavioral outcomes in these mice as determined by negative binomial regression models (*p* < 0.001). For example, members of the genus *Bacteroides* are positively associated with all measures of open field exploration times (Figure 12A), negatively correlated with the percent spontaneous alternation, and positively associated with the grip strength score. Conversely, *Lactobacillus* is negatively associated with all measures of open field exploration times, positively associated with the percent spontaneous alternation, and negatively associated with the grip strength score. In A53T-L444P mice, *Lactobacillus* was relatively depleted while *Bacteroides* was relatively in abundance.

### 3.6. Microbiome Results—Study 4

DSS exposure significantly affected the biodiversity (Shannon entropy, *p* = 0.171) and composition (*p* = 0.001) of the gut microbiome in both genotypes (see Tables S3 and S4 for the DSS taxon and metadata, respectively). Moreover, microbiome biodiversity (Shannon entropy, *p* = 0.024) and composition (*p* = 0.001) differed as a function of sex (*p* < 0.05), but not of genotype. More specifically, A53T-L444P mice did not significantly differ in their gut microbiome biodiversity or composition as compared to A53T mice, nor did the microbiomes of these mice differ in their response to the DSS exposure in terms of biodiversity or composition. However, our analysis revealed that the L444P genotype impacts how the microbiome’s biodiversity and composition associate with cognition. Linear regression revealed that there was no association between gut microbiome diversity and hippocampus-dependent percent spontaneous alternations in the Y-maze until accounting for the variation in genotype; the biodiversity of A53T-L444P mice manifest a positive relationship with the percent spontaneous alternation, whereas microbiome biodiversity of A53T mice negatively associates with the percent spontaneous alternation (Shannon Entropy, *p* = 0.030) (Figure 12B). Additionally, the composition of the gut microbiome links to the percent spontaneous alternation, but does so differently in A53T-L444P mice than in A53T mice (Weighted Unifrac, *p* = 0.007). Similar genotype-dependent associations between gut microbiome composition and cognitive performance were observed for the improvement in rotorod performance and percent time spent freezing in the hippocampus-dependent contextual fear memory test. Additionally, we found that DSS exposure modulated the association between the gut microbiome and behavior. For example, the composition of the microbiome associated with total distance moved during an open field test (Bray–Curtis, *p* = 0.001), the number of spontaneous alternations during a Y-max (Bray–Curtis, *p* = 0.002), change in rotorod performance (Bray–Curtis, *p* = 0.014), and contextual fear memory (Bray–Curtis, *p* = 0.001).

## 4. Discussion

The results of these four studies, including the various treatment × genotype interactions, show that PQ and PQ + IR have differential effects in A53T and A53T-L444P mice. In the open field containing objects, PQ-treated A53T mice showed lower activity levels. In the Y maze, PQ-treated A53T-L444P mice showed more hippocampus-dependent spontaneous alternations and higher activity levels than saline-treated A53T-L444P mice. In addition, PQ-treated A53T-L444P female mice showed lower freezing levels during the tones during fear learning and lower freezing levels during the hippocampus-dependent contextual fear memory test than saline-treated A53T-L444P female mice. There were also effects of PQ seen in both genotypes. PQ-treated mice showed lower activity and higher anxiety levels than saline-treated mice in the open field and lower activity levels during the baseline period during fear learning.

When mice were exposed to PQ + IR, they showed lower activity levels in the open field and increased freezing and reduced activity levels during the baseline period during fear learning compared to control-treated mice. PQ + IR-treated A53T-L444P female mice showed higher anxiety levels in the open field than control-treated mice. Increased measures of anxiety of A53T-L444P mice in the elevated zero maze were observed at 20 months of age as compared to the A53T genotype alone [30]. Together with the other data in the current study, this indicates the PQ + IR environmental challenge can induce phenotypes earlier in life. In addition, PQ + IR-treated A53T female mice showed less cued fear memory and PQ + IR-treated A53T-L444P female mice more cued fear memory than genotype-type matched control-treated female mice. These data indicate that the effects of PQ and PQ + IR are model- and sex-dependent. There were also effects of PQ + IR seen in both genotypes. PQ + IR-treated mice moved less than control-treated mice in the open field and during the baseline period during fear learning, consistent with the effects of PQ alone seen on these behavioral measures. In addition, PQ + IR-treated mice showed higher freezing levels during the baseline period during fear learning, reduced motion to the shocks during fear learning, and increased freezing levels and reduced activity levels during the first tone during fear learning. These data show that the effects of PQ and PQ + IR are model- and sex-dependent.

In contrast to effects of PQ and PQ + IR that were seen in both genotypes, effects of DSS were only seen in A53T-L444P mice. DSS-treated A53T-L444P mice showed impaired performance on the rotorod and reduced spontaneous alternations in the Y-maze. In addition, A53T-L444P females showed enhanced activity levels during the baseline period during fear learning. The increased susceptibility of A53T-L444P compared to A53T mice to DSS, which primarily challenges the colon [44], is consistent with the over-representation of constipation in PD patients carrying *GBA* mutations compared to patients with idiopathic PD [45]. However, we recognize that as the effects of DSS are transient and the mice were tested during the DSS treatment period but after the PQ, radiation, and PQ + IR treatment periods, we cannot exclude that differences in post-treatment periods might have contributed to this genotype difference.

Besides genotype-dependent treatment responses, there were some genotype differences as well. A53T-L444P mice showed more depressive-like behavior than A53T mice in the forced swim test in studies 1 and 3, consistent with increased depression seen in PD patients carrying *GBA* mutations with constipation compared to patients with idiopathic PD [45]. A53T-L444P mice also show increased contextual and cued fear memory compared to A53T mice in study 1, lower activity levels than A53T mice in the open field and Y-maze, lower activity levels than A53T mice during the baseline period and the ISI during fear learning, and increased activity levels during the contextual fear memory test in study 4. These results might relate to the often earlier age at onset of PD patients carrying *GBA* mutations compared to patients with idiopathic PD [45] and are consistent with the more profound motor phenotype in A53T-L444P genotype at 14 to 15 months of age [30].

While increased anxiety levels in A53T and A53T-L444P female and male mice in the open field were seen following PQ exposure, increased anxiety levels in the open field were only seen in PQ + IR-treated A53T-L444P female mice. Thus, while PQ can interact with ionizing radiation to induce cognitive injury [37] and irradiation might increase PD risk [32], combined PQ + IR exposure might protect against detrimental effects of PQ alone in A53T mice and A53T-L444P male mice. As radiation exposure occurred on the first day of PQ treatment, this might involve irradiation-induced preconditioning [46,47,48].

For various behavioral measures, there was an increased susceptibility of A53T-L444P female mice especially to effects of PQ on performance in the fear conditioning test, to effects of PQ + IR in the open field with and without objects and the fear conditioning test, and effects of DSS in the fear conditioning test (Table 1). The increased susceptibility of female A53T-L444P mice to environmental challenges is consistent with the higher mortality and faster disease progression in female than male PD patients, although the risk of developing PD is higher in men than women [49].

PQ-treated A53T-L444P female mice showed impaired hippocampus-dependent contextual fear memory. As the hippocampus is sensitive to effects of radiation but no effects on contextual fear memory were seen in PQ + IR-treated A53T-L444P female mice, this protection might involve the earlier mentioned irradiation-induced preconditioning [46,47,48].

PQ + IR-treated A53T-L444P female mice showed enhanced hippocampus-independent cued fear memory. Increased anxiety levels might have contributed to the enhanced cued fear memory of PQ + IR-treated A53T-L444P female mice. PQ + IR-treated A53T-L444P female mice showed enhanced anxiety levels in the open field and spent less time in the center of the open field that contained the objects.

In mice only exposed to PQ or saline, there was an effect of sex, with females losing more percent body weights than males. However, there was no effect of sex on body weight when mice were exposed to PQ + IR or DSS. This suggests that, although there was no treatment × sex interaction for body weights, the presence of PQ likely contributed to female mice losing more percent body weight than males. The fact that there was no effect of sex for body weight loss when mice were exposed to PQ + IR suggests that radiation exposure mitigates this effect.

Our investigation of the gut microbiome indicates that exposure and genotype can modulate the relationship between the gut microbiome and behavioral performance. The gut microbiome and the gut–brain axis can even drive behavior and cognition in mouse models [50]. It is less well understood, however, whether the effect of different exposures or genotypes that drive behavioral variation do so, at least in part, through their effect on the gut microbiome. We can obtain insight into this possibility through analysis of the association between the gut microbiome and behavioral performance, and how this association varies as a function of exposure to PQ or DSS, as well as different genotypes.

In this study, we found that PQ and DSS elicit different direct effects on the gut microbiome, possibly as a result of the difference in the severity of intestinal inflammation that these two exposures induce. That said, both PQ and DSS exposures impacted the association between the composition of the gut microbiome and various measures of behavior and cognition. For example, mouse performance during open field tests of exploratory behavior associated with the composition of the gut microbiome, indicating that the types of taxa present in the gut relate to this behavioral phenotype; however, the nature of this relationship differed depending on whether the mice were exposed to either PQ or DSS relative to saline controls. In other words, exposure to these agents altered the association between the gut microbiome and behavior. Based on these results, we hypothesize that the gut microbiome mediates the effect of PQ or DSS exposure on behavior, at least in part, possibly because exposure to these agents impacts the overarching composition of the gut microbiome, and that the types of communities that assemble in the post-exposure gut induces different effects on brain functioning, likely due to variation in the functioning of the gut microbiome.

Moreover, we found that mouse genotype modulated the association between the gut microbiome’s composition and behavior in both of our trials, with percent time freezing in a contextual fear memory test associating with the microbiome in a genotype-dependent manner for both studies 1 and 4. These results indicate that genotype modulates the gut microbiome–brain relationship, and suggest that some of the effects of genotype on behavior may manifest as a result of genotypic alterations to the functioning of the gut microbiome.

While few studies have explored the effect of exposure or genotype on the relationship between the gut microbiome and behavioral performance, our analyses are supported by prior studies. For example, prior work demonstrated that allelic variation in humanized mouse models of Alzheimer’s disease yielded variation in the composition of the gut microbiome that associated with hippocampal epigenetic variation as well changes in the behavior of mice that received transplants of these genotype-linked microbiomes [43,51]. In addition, transplanting stool from patients with social anxiety disorder into C57BL6/J wild-type mice treated with antibiotics showed enhanced social fear in a social fear conditioning test [52]. Going forward, it will be important to validate the causal role the microbiome plays in mediating the effects of DSS and PQ on behavioral and cognitive performance through similar microbiome transplantation studies. Overall, our efforts indicate that the gut microbiome may be an important mediator of how exposures or mutations yield behavioral and cognitive impacts. Future efforts are warranted to assess whether the treatment effects on the gut microbiome observed reflect inflammation and/or degeneration of the enteric nervous system and/or central nervous system, including analysis of synucleinopathy in these two systems.

## Figures and Tables

**Table 1:** Summary of behavioral data of studies 1, 3, and 4 ^1^.

Behavioral Measure	Study 1 (PQ)	Study 3 (PQ + IR)	Study 4 (DSS)
Open field: activity	PQ < saline	PQ + IR < control	A53T-L444P < A53T
Open field: anxiety	PQ > saline	A53T-L44P females: PQ + IR > control	
Open field with objects: activity	A53T: PQ < saline		
Open field with objects: time in center where the objects are located		A53T-L44P females: PQ + IR < control	
Rotorod: fall latency			A53T-L444P: DSS < H_2_O
Y-maze: spontaneous alternations	A53T-L444P: PQ > saline		A53T-L444P: DSS < H_2_O
Y-maze: activity	A53T-L444P: PQ > saline		A53T-L444P < A53T
Forced swim test immobility: depressive like behavior	A53T-L444P > A53T	A53T-L444P > A53T	
Fear conditioning: freezing during the baseline period		PQ + IR > control	
Fear conditioning: activity levels during baseline period	PQ < saline	PQ + IR < control	A53T-L444P < A53T Males: A53T-L444P < A53T A53T-L444P females: DSS > H_2_O
Fear conditioning: freezing during tones fear learning	A53T-L444P females: PQ < saline	Tone 1: PQ + IR > control	
Fear conditioning: activity levels during the tones		Tone 1: PQ + IR < control	
Fear conditioning: activity during the shocks		PQ + IR < control (also in males only)	
Fear conditioning: activity during the ISI interval			A53T-L444P < A53T
Fear conditioning: freezing during the contextual fear memory	A53T-L444P > A53T A53T-L444P females: PQ < saline		
Fear conditioning: activity during the contextual fear memory test	A53T-L444P females: PQ > saline		A53T-L444P > A53T
Fear conditioning: freezing during The cued fear memory test	A53T-L444P > A53T Pre-tone: PQ > saline	A53T females: PQ + IR < control A53T-L444P females: PQ + IR > control	
Fear conditioning: activity during the cued fear memory test	A53T-L444P < A53T	A53T-L444P > A53T Pre-tone: A53T-L444P > A53T	

^1^ No results of study 2 were included as there were no significant radiation effects.

## Data Availability

The datasets for this study can be found in the Supplementary Materials. The 16S data were uploaded on the NCBI SRA under the following accession numbers: PRJNA1054189, and PRJNA1054190.

## Supplementary Materials

The following supporting information can be downloaded at: https://www.mdpi.com/article/10.3390/genes15030282/s1, Supplementary Figure Legends: Supplementary Figures, metadata: Tables S1–S4.

## References

[1] Chaudhuri K., Healy D., Schapira A. (2006). Non-motor symptoms of Parkinson’s disease: Diagnosis and management. Lancet Neurol..

[2] Raber J., KLD S., Savica R. (2021). Physical activity may reduce apolipoprotein E4-associated cogntive decline in Parkinson disease. Neurology.

[3] Rahman S., Griffin H., Quinn N. (2008). Quality of like in Parkinson’s disease: The relative importance of the symptoms. Mov. Disord..

[4] Raskind M.A. (2008). Diagnosis and treatment of depression comorbid with neurologic disorders. Am. J. Med..

[5] Shen C.-C., Tsai S.-J., Perng C.-L., Ing-Tiau Kuo B., Yang A. (2013). Risk of Parkinson disease after depression. Neurology.

[6] Kashani A., Betancur C., Giros B., Hirsch E., El Mestikawy S. (2007). Altered expression of vesicular glutamate transporters VGLUT1 and VGLUT2 in Parkinson disease. Neurobiol. Aging.

[7] Emre M. (2015). Cognitive Impairment and Dementia in Parkinson’s Disease.

[8] Sampson T., Debelius J., Thron T., Janssen S., Shastri G., Ilhan Z., Chalis C., Schretter C., Rocha S., Gradinaru V. (2016). Gut microbiota regulate motor deficits and neurinflammation in a model of Parkinson’s disease. Cell.

[9] McCormack A., Thiruchelvam M., Manning-Bog A., Thiffault C., Langston J. (2002). Environmental risk factors and Parkinson’s disease: Selective denegeration of nigral dopaminergic neurons caused by the herbicide paraquat. Neurobiol. Dis..

[10] Feltzin V., Wan K., Cleniker S., Bonini N.M. (2019). Role and impact of the gut microbiota in a Drosophila model for parkinsonism. bioRxiv.

[11] Pesah Y., Pham T., Burgess H., Middlebrooks B., Verstreken P., Zhou Y., Harding M., Bellen H., Mardon G. (2004). Drosophila parkin mutants have decreased mass and cell size and increased sensitivity to oxygen radical stress. Development.

[12] Ball N., Teo W.-P., Chandra S., Chapman J. (2019). Parkinson’s Disease and the Environment. Front. Neurol..

[13] Goldman S. (2014). Environmental toxins and Parkinson’s Disease. Ann. Rev. Pharmacol. Toxicol..

[14] Collaborators G.B.D.R.F., Forouzanfar M.H., Alexander L., Anderson H.R., Bachman V.F., Biryukov S., Brauer M., Burnett R., Casey D., Coates M.M. (2015). Global, regional, and national comparative risk assessment of 79 behavioural, environmental and occupational, and metabolic risks or clusters of risks in 188 countries, 1990–2013: A systematic analysis for the Global Burden of Disease Study 2013. Lancet.

[15] Wray A. (2019). Systematic Review of the Literature to Evaluate the Relationship between Paraquat Dichloride Exposure and Parkinson’s Disease. https://www.regulations.gov/document/EPA-HQ-OPP-2011-0855-0125.

[16] Tanner C.M., Kamel F., Ross G.W., Hoppin J.A., Goldman S.M., Korell M., Marras C., Bhudhikanok G.S., Kasten M., Chade A.R. (2011). Rotenone, Paraquat, and Parkinson’s disease. Environ. Health Perspect..

[17] Anselmi L., Bove C., Coleman F.H., Le K., Subramanian M.P., Venkiteswaran K., Subramanian T., Travagli R.A. (2018). Ingestion of subthreshold doses of environmental toxins induces ascending Parkinsonism in the rat. NPJ Parkinson’s Dis..

[18] Labrie V., Brundin P. (2017). α-Synuclein to the Rescue: Immune Cell Recruitment by α-Synuclein during Gastrointestinal Infection. J. Innate Immun..

[19] Keshvarzian A., Engen P., Bonvegna S., Cilian R. (2020). The gut microbiome in Parkinson’s disease: A culprit or a bystander?. Progress. Brain Res..

[20] Kishimoto Y., Zhu W., Hosoda W., Sen J.M., Mattson M.P. (2019). Chronic mild gut inflammation accelerates brain neuropathology and motor dysfunction in α-synulcein mutant mice. NeuroMol. Med..

[21] Forum A.R. (2019). Do Immune Cells Promote the Spread of α-Synuclein Pathology?. https://www.alzforum.org/news/conference-coverage/do-immune-cells-promote-spread-synuclein-pathology.

[22] Grathwohl S., Quansah E., Maroof N., Steiner J.A., Spycher L., Benmansour F., Duran-Pacheco G., Siebourg-Polster J., Oroszlan-Szovik K., Remy H. (2021). Specific immune modulation of experimental colitis drives enteric alpha-synuclein accumulation and triggers age-related Parkinson-like brain pathology. Free Neuropathol..

[23] Foster J., McVey Neufeld K.-A. (2013). Gut-brain axis: How the microbiome influences anxiety and depression. Trends Neurosci..

[24] Allen A., Dinan T., Clarke G., Cryan J. (2017). A psychology of the human brain-gut-microbiome axis. Soc. Personal. Psychol. Compass.

[25] Kelly J., Kennedy P., Cryan J.F., Dinan T., Clarke G., Hyland H. (2015). Breaking down the barriers: The gut miocrobiome, intestinal permeability and stress-related psychiatric disorders. Front. Cell. Neurosci..

[26] Malek N., Weil R., Bresner C., Lawton M., Grosset K., Tan M., Bajaj N., Barker R., Burn D., Foltynie T. (2018). Features of GBA-associated Parkinson’s disease at presentation in the UK Tracking Parkinson’s study. J. Neurol. Neurosurg. Psych..

[27] Migdalska-Richards A., Schapira A. (2016). The relationship between glucocerebrosidase mutations and Parkinson disease. J. Neurochem..

[28] Avenali M., Blandinin F., Cerri S. (2020). Glucocerebrosidase defects as a major risk factor for Parkinson’s disease. Front. Aging Neurosci..

[29] Pil Yun S., Kim D., Kim S., Kim S., Karuppagounder S., Kwon S.-H., Lee S., Kam T.-I., Lee S., Ham S. (2018). α-Synuclein accumulation and GBA deficiency due to L444P GBA mutation contributes to MPTP-induced parkinsonism. Mol. Neurodegener..

[30] Fishbein I., Kuo Y., Giasson B., Nussbaum R. (2014). Augmentation of phenotype in a transgenic Parkinson mouse heterozygous for a Gaucher mutation. Brain.

[31] Durakovic A. (2017). Medical Effects of a Transuranic “Dirty Bomb”. Mil. Med..

[32] Azizova T., Bannikova M., Grigoryaeva E., Rybkina V., Hamada N. (2020). Occupational exposure to chronic ionizing radiation increases risk of Parkinson’s diseasecincidence in Russian Mayak workers. Int. J. Epidemiol..

[33] Lam V., Moulder J., Salzman N., Dubinsky E., Andersen G., Baker J. (2012). Intestinal microbiota as novel biomarkers of prior radiation exposure. Radiat. Res..

[34] Guo H., Chou W.C., Lai Y., Liang K., Tam J.W., Brickey W.J., Chen L., Montgomery N.D., Li X., Bohannon L.M. (2020). Multi-omics analyses of radiation survivors identify radioprotective microbes and metabolites. Science.

[35] Raber J., Yamazaki J., Torres E., Kirchoff N., Stagaman K., Sharpton T.J., Turker M., Kronenberg A. (2019). Combined effects of three high energy charged particle beams important for space flight on brain, behavioral and cognitive endpoints in B6D2F1 female and male mice. Front. Physiol..

[36] Raber J., Fuentes Anaya A., Torres E., Lee J., Boutros S., Grygoryev D., Hammer A., Kasschau K., Sharpton T., Turker M. (2020). Effects of Six Sequential Charged Particle Beams on Behavioral and Cognitive Performance in B6D2F1 Female and Male Mice. Front. Physiol..

[37] Phillippot G., Stenerlow B., Frederiksson A., Sundell-Bergmman S., Eriksson P., Buratovic S. (2018). Developmental effects of neonatal fractionated co-exposure to low-dose γ radiation and paraquat on behaviour in adult mice. J. Appl. Toxicol..

[38] Jadavji N., Murray L., Emmerson J., Rudyk C., Hayley S., Smith P. (2019). Paraquat exposure increases oxidatvie stress within the dorsal striatum of male mice with a genetic deficiency in one-carbon metabolism. Toxicol. Sci..

[39] van Putten M. The Use of Hanging Wire Tests to Monitor Muscle Strength and Condition over Time. 2011, TREAT_NMD Neuromascular Network, DMD_M.2.1.004, Version 4.0. https://www.treat-nmd.org/wp-content/uploads/2023/07/DMD_M_2.1.004.pdf.

[40] Takeshita H., Yamamoto K., Nozato S., Inagaki T., Tsuchimochi H., Shirai M., Yamamoto R., Imaizumi Y., Hongyo K., Yokoyama S. (2017). Modified forelimb grip strength test detects aging-associated physiological decline in skeletal muscle function in male mice. Sci. Rep..

[41] Seok Son J., Kim H.-J., Son Y., Lee H., Chae S., Seong J., Song W. (2017). Effects of exercise-induced apelin levels on skeletal muscle and their capillarization in type 2 diabetic rats. Muscle Nerve.

[42] Taylor T., Caudle W., Shepherd K., Noorian A., Jackson C., Iuvone P.M., Weinschenker D., Greene J., Miller G. (2009). Nonmotor symptoms of Parkinson’s disease revealed in an animal model with reduced monoamine storage capacity. J. Neurosci..

[43] Kundu P., Stagaman K., Kasschau K., Holden S., Shulzhenko N., Sharpton T., Raber J. Fecal implants from AppNL-G-F and AppNL-G-F/E4 donor mice sufficient to induce behavioral phenotypes in germ-free mice. Front. Behav. Neurosci..

[44] Whittem C., Williams A., Williams C. (2010). Murine colitis modeling using dextran sulfate sodium (DSS). J. Vis. Exp..

[45] Riboldi G., Di Fonzo A. (2019). GBA, Gaucher Disease, and Parkinson’s Disease: From Genetic to Clinic to New Therapeutic Approaches. Cells.

[46] Khbouz B., Gu S., Pinto Coelho T., Lallemand F., Jouret F. (2023). Radiotherapy Advances in Renal Disease—Focus on Renal Ischemic Preconditioning. Bioengineering.

[47] Raber J., Allen A.R., Sharma S., Allen B., Rosi S., Olsen R.H.J., Davis M.J., Eiwaz M., Fike J.R., Nelson G.A. (2015). Effects of proton and combined proton and 56Fe irradiation on the hippocampus. Radiat. Res..

[48] Raber J., Holden S., Sudhakar R., Hall R., Glaeser B., Lenarczyk M., Rockwell K., Nawarawong N., Sterrett J., Perez R. (2021). Effects of 5-Ion Beam Irradiation and Hindlimb Unloading on Metabolic Pathways in Plasma and Brain of Behaviorally Tested WAG/Rij Rats. Front. Physiol..

[49] Cerri S., Mus L., Blandinin F. (2019). Parkinson’s Disease in Women and Men: What’s the Difference?. J. Parkinsons Dis..

[50] Dinan T., Cryan J.F. (2017). Brain-gut-microbiota axis—Mood, metabolism, and behaviour. Nat. Rev. Gastroenterol. Hepatol..

[51] Kundu P., Torres E., Stagaman K., Kasschau K., Okhovat M., Holden S., Ward S., Nevonen K., Davis B., Saito T. (2021). Integrated analysis of behavioral, epigenetic, and gut microbiome analyses in AppNL-G-F, AppNL-F, and wild type mice. Sci. Rep..

[52] Ritz N., Brocka M., Butler M., Cryan J.F. (2023). Social anxiety disorder-associated gut microbiota increase social fear. Proc. Natl. Acad. Sci. USA.

