# Supplementary material for: Effects of Paraquat, Dextran Sulfate Sodium, and Irradiation on Behavioral and Cognitive Performance and the Gut Microbiome in A53T and A53T-L444P Mice
Source: Genes (Basel). 2024 Feb 23;15(3):282. doi: 10.3390/genes15030282 (PMC11154584; doi:10.3390/genes15030282)
Supplement: Supplementary file 1 [file genes-15-00282-s001.zip › genes-2846669-supplementary.pdf]

Supplementary Materials

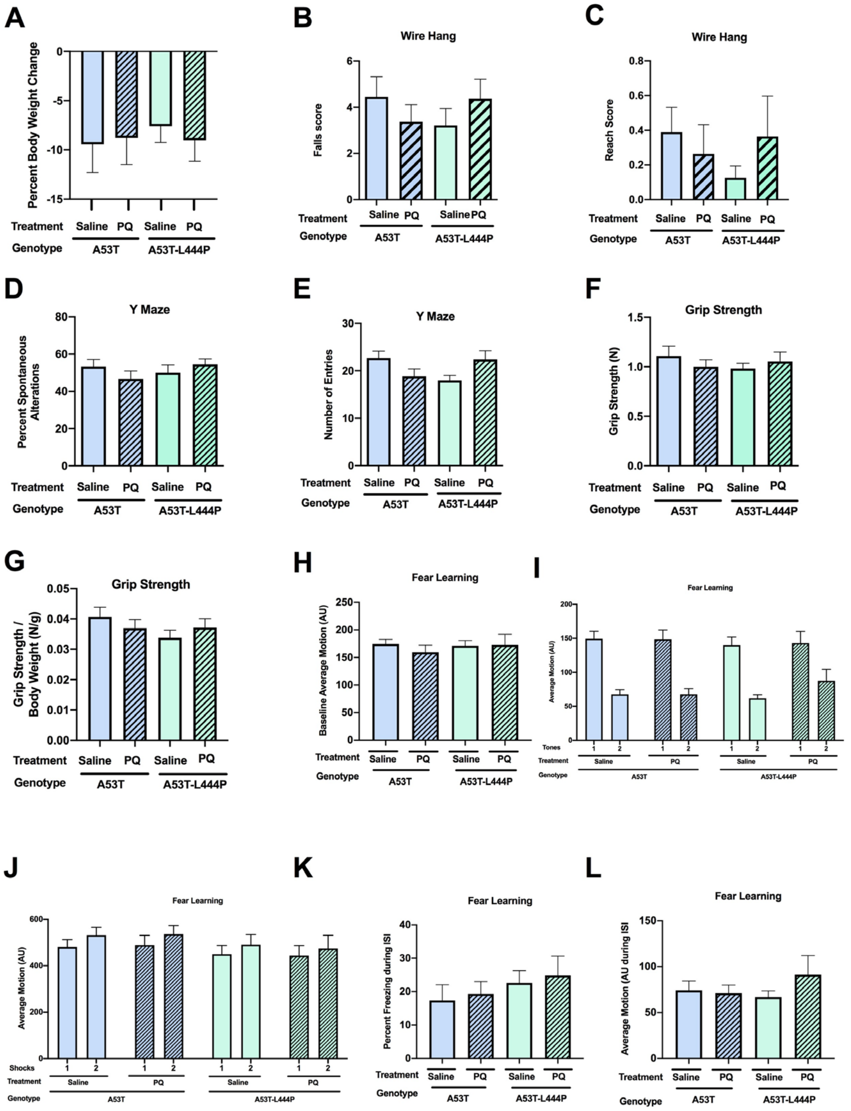

Figure S1. A. There was no effect of genotype or treatment on the percent body weight change. **B.** There was no effect of genotype or treatment on fall scores in the wire hang test. **C.** There was no effect of genotype or treatment on reach scores in the wire hang test. **D.** In the grip strength test, there was no effect of genotype or treatment. **E.** When grip strength per body weight was analyzed, there was no effect of genotype or treatment.

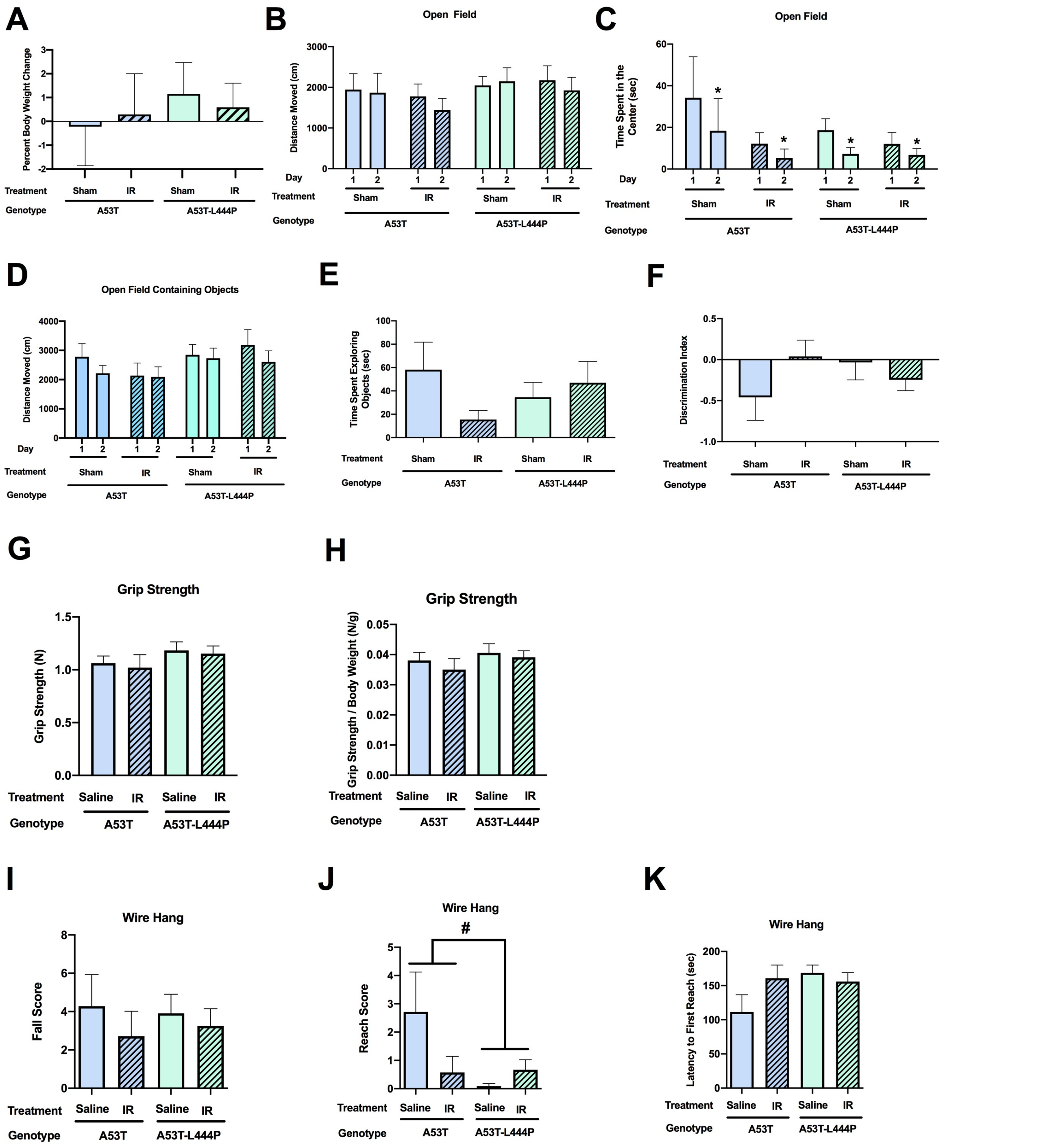

Figure S2. A. There was no effect of genotype or treatment on the percent body weight change. **B.** There was no effect of genotype or treatment on activity levels during open field testing. **C.** When time spent in the center of the open field was analyzed, there was an effect of day ($F(1,29) = 6.309$, $^*p = 0.018$). **D.** There was no effect of genotype or treatment on

activity levels in the open field containing objects. **E.** There was no effect of genotype or treatment on time spent exploring the objects in the object recognition test. **F.** There was no effect of genotype or treatment on the discrimination index in the object recognition test. **G.** There was no effect of genotype or treatment on grip strength. **H.** There was no effect of genotype or treatment on grip strength per body weight. **I.** There was no effect of genotype or treatment on fall scores in the wire hang test. **J.** There was a trend towards an effect of genotype for reach scores ($F(1,29) = 3.271$, $^{\#}p = 0.081$), with a trend towards higher reach scores in A53T than A53T-L444P mice. **K.** There was a no effect of genotype or treatment on latency to first reach in the wire hang test.

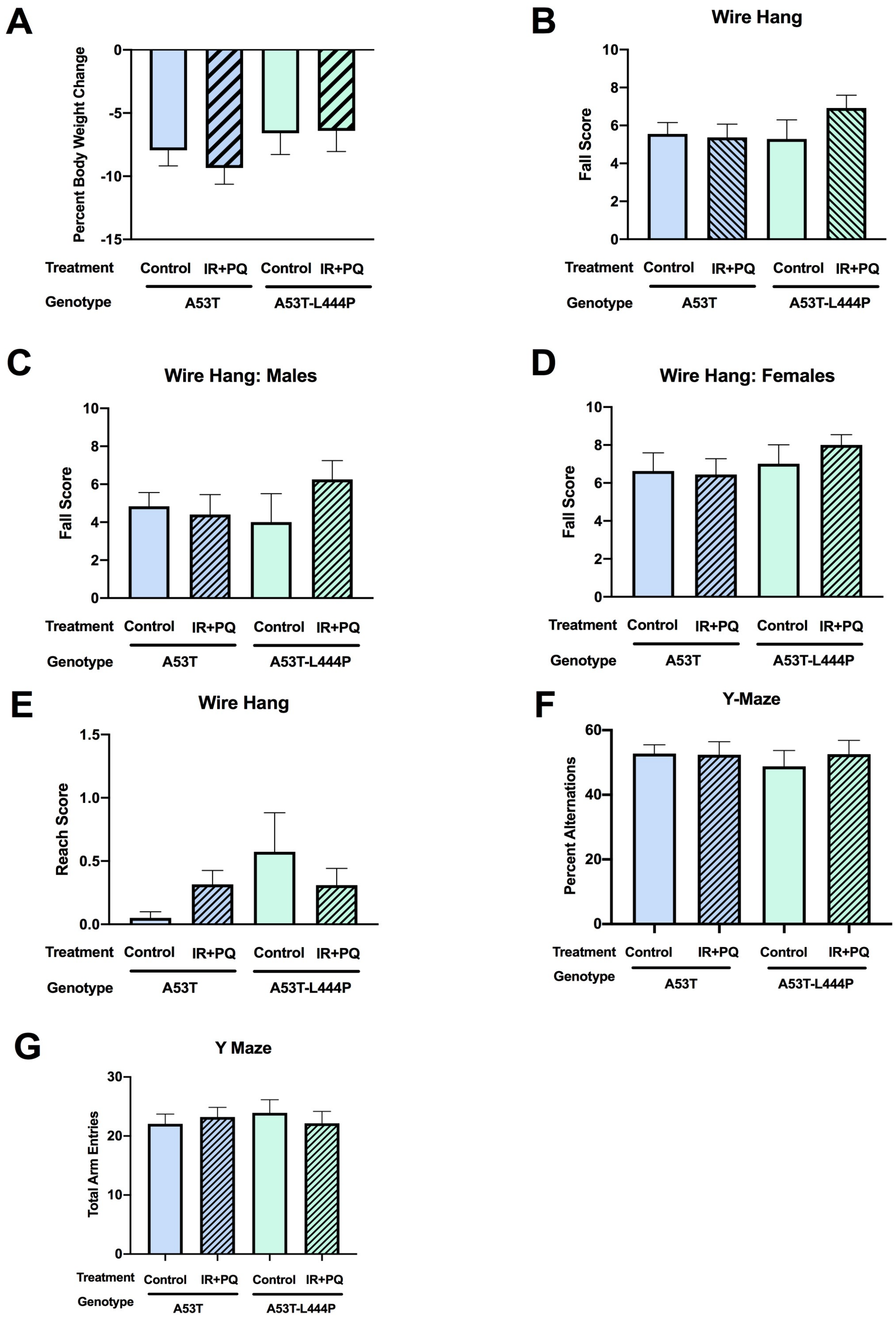

Figure S3.A. There was no effect of genotype or treatment on the percent body weight change. **B.** There was an effect of sex for fall scores in the wire hang test ($F(1,58) = 8.345$, p = 0.005). **C.** Fall scores in males. **D.** Fall score in females were higher than those in males (C).

E. For reach scores, there was a trend towards a treatment x sex x genotype interaction ($F(1,58) = 3.248$, $p = 0.077$). **F.** There was no effect of genotype or treatment on spontaneous alternation in the Y maze. There was no effect of genotype or treatment for the number of entries in the Y maze ($F(1,58) = 3.949$, $p = 0.052$).

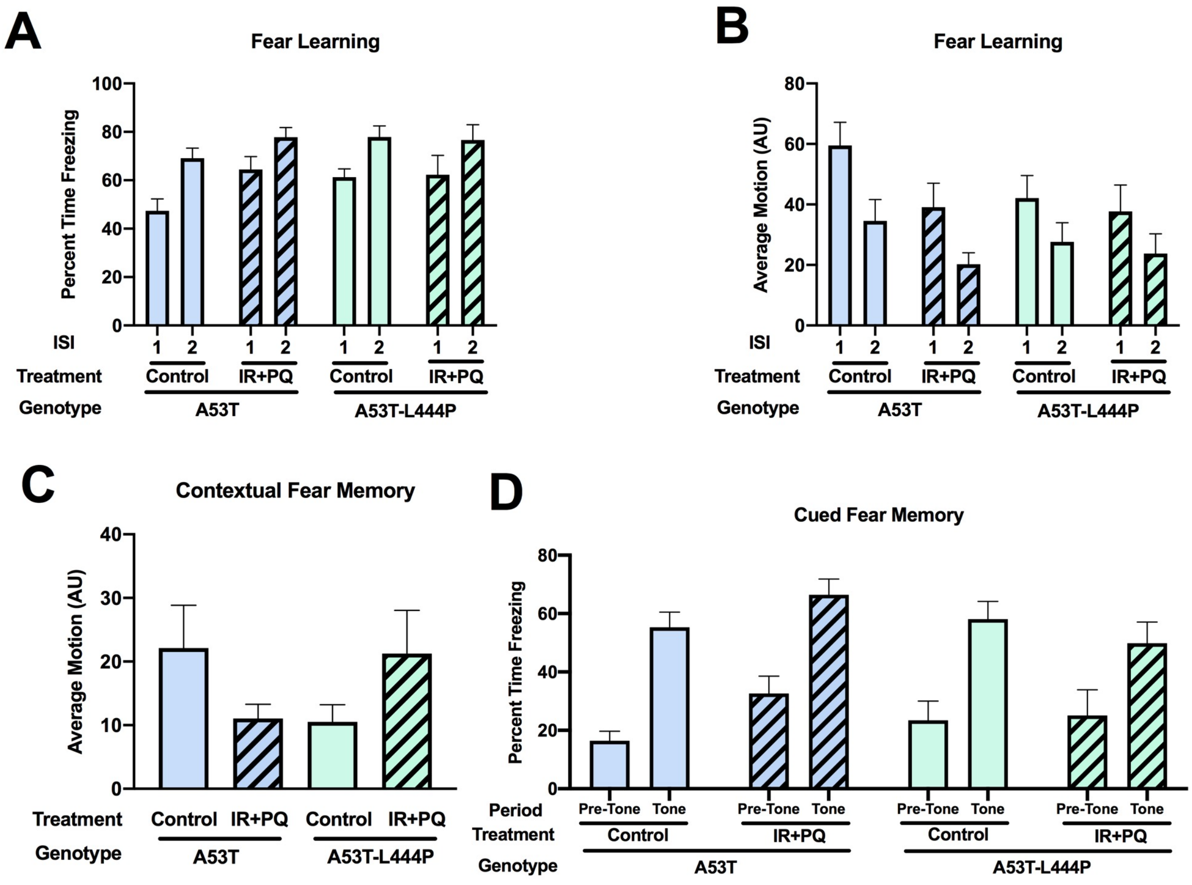

Figure S4. A. There was no effect of genotype or treatment on freezing levels during the ISIs. **B.** There was no effect of genotype or treatment on activity levels during the ISIs. **C.** There was no effect of genotype or treatment on activity levels during the contextual fear memory test. **D.** There was no effect of genotype or treatment on freezing levels during the cued fear memory test.

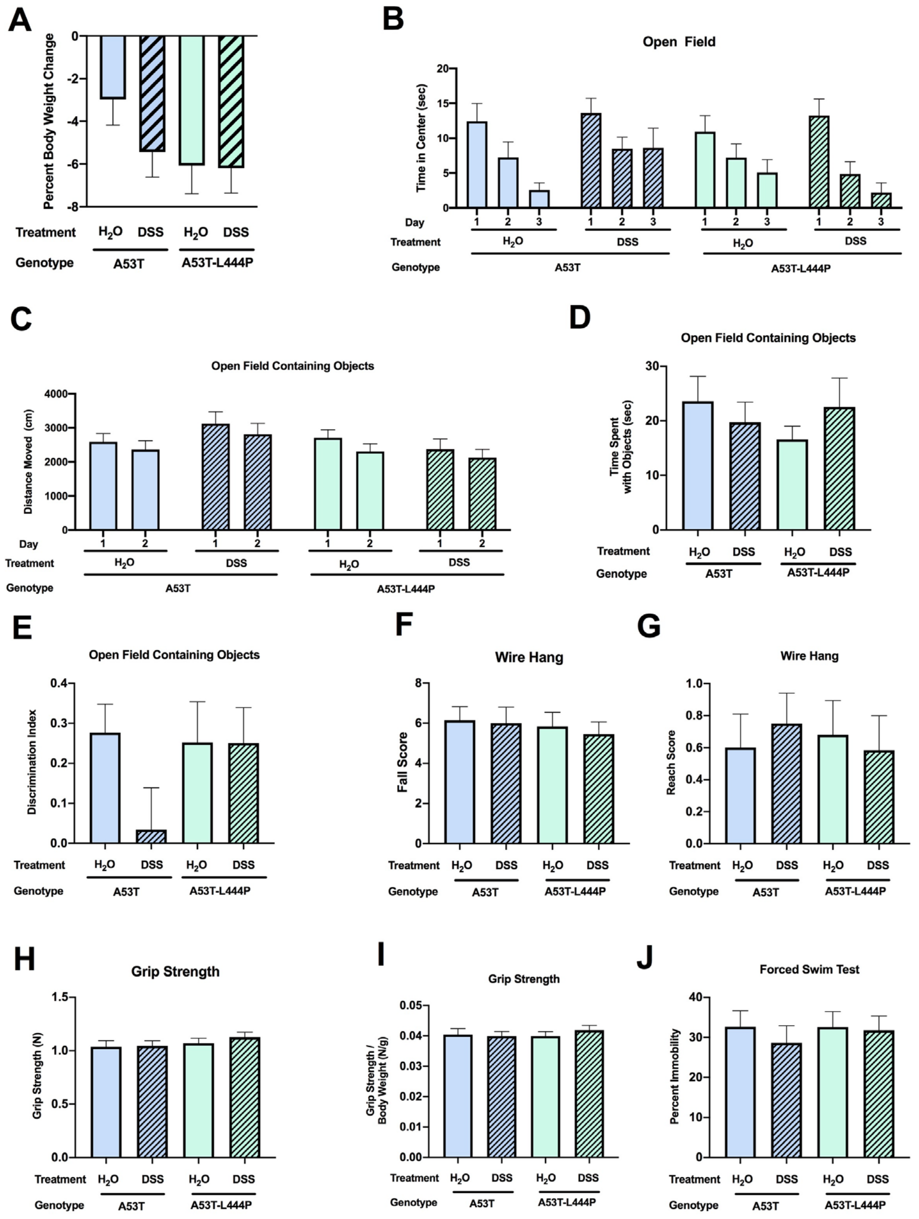

Figure S5. A. There was no effect of genotype or treatment on the percent body weight change. **B.** There was no effect of genotype or treatment on anxiety levels in the open field. **C.** There was no effect of genotype or treatment on activity levels in the open field containing objects. **D.** There was no effect of genotype or treatment on time spent exploring the objects in the object recognition test. **E.** There was no effect of genotype or treatment on the discrimination index. **F.** There was no effect of genotype or treatment on fall scores in

the wire hang test. **G.** There was no effect of genotype or treatment on reach scores in the wire hang test. **H.** There were no effects of genotype or treatment on performance in grip strength test. **I.** There was no effect of genotype, treatment on grip strength per body weight. **J.** There were no effects of genotype or treatment on depressive-like behavior in the forced swim test.

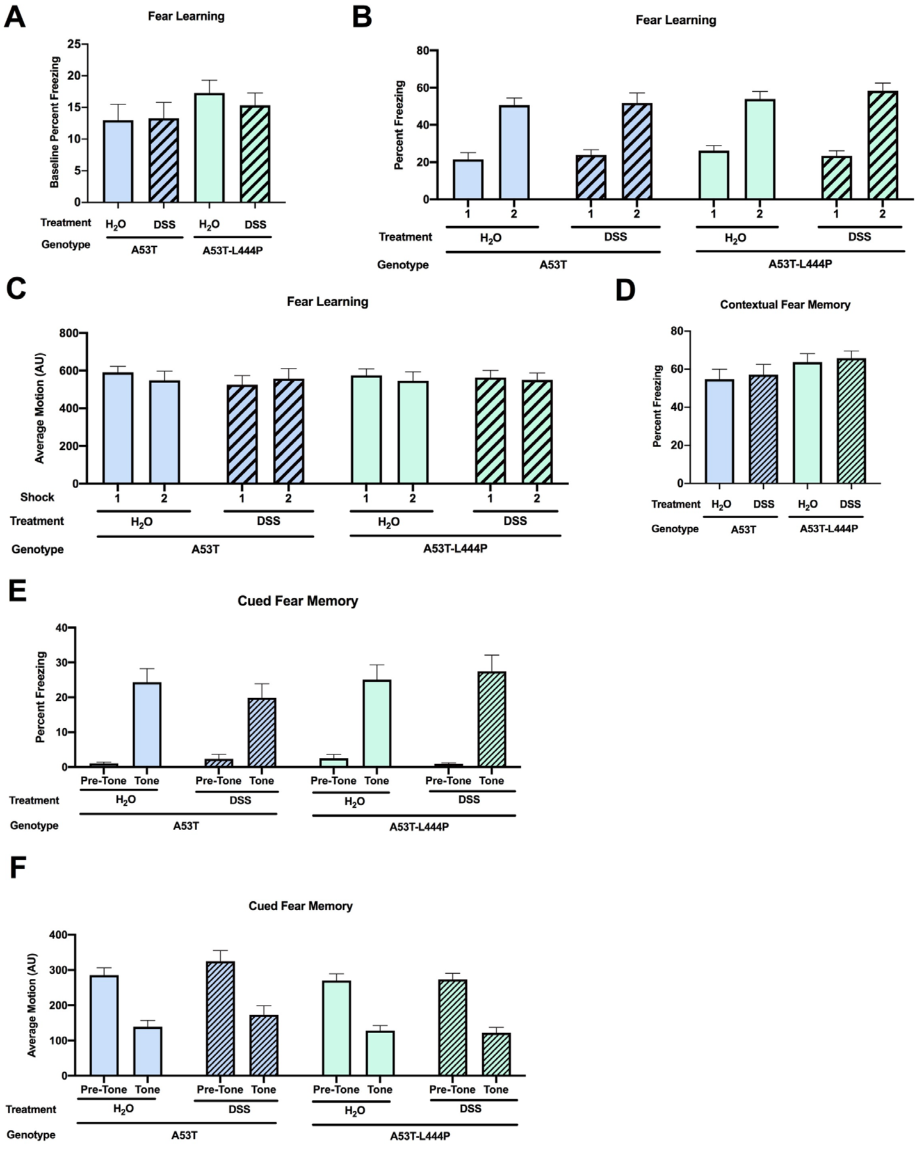

Figure S6. A. There were no effects of genotype or treatment on percent freezing during the baseline period in the fear conditioning test. **B.** There was no effect of genotype or treatment

on freezing during the tones. **C.** There was no effect of genotype or treatment on response to the shocks. **D.** There was no effect of genotype or treatment on freezing levels during the contextual fear memory test. **E.** There was no effect of genotype or treatment on freezing levels during the cued fear memory test. **F.** There was no effect of genotype or treatment on activity levels during the cued fear memory test.

Table S1. PQ_sample_data.

	Kingdom	Phylum	Class	Order	Family	Genus	Taxon
ASV015	Bacteria	Firmicutes	Clostridia	Lachnospirales	Lachnospiraceae	Lachnospiraceae_Genus	TACGTATGGAGCAAGCGTTATCCGGATTTACTGGGTGTAAAGGGAGCGCAGACGGCAGGGCAAGTCTGATGTGAAAGTCCGGGGCTCAACCCCGGGACTGCATTGGAAACTGTCCGGCTAGAGTGCAGGAGAGGTAAGTGGAATTCCTAGTGTAGCGGTGAAATGCGTAGATATTAGGAGGAACACCAGTGGCGAAGGCGGCTTACTGGACTGTAACTGACGTTGAGGCTCGAAAGCGTGGGGAGCAAACAGG
ASV360	Bacteria	Firmicutes	Clostridia	Lachnospirales	Lachnospiraceae	Lachnospiraceae_Genus	TACGTAGGGGGCAAGCGTTATCCGGATTTACTGGGTGTAAAGGGAGCGTAGACGGTATGGTAAGTCAGATGTGAAAGCCCGGGGCTTAACCCCGGAACTGCATTTGAAACTATCAAACTAGAGTGTCGGAGAGGTAAGTGGAATTCCTAGTGTAGCGGTGAAATGCGTAGATATTAGGAGGAACACCAGTGGCGAAGGCGGCTTACTGGACGATAACTGACGTTGAGGCTCGAAAGCGTGGGGAGCAAACAGG
ASV098	Bacteria	Firmicutes	Clostridia	Lachnospirales	Lachnospiraceae	Lachnospiraceae_Genus	TACGTATGGTGCAAGCGTTATCCGGAATGACTGGGTGTAAAGGGAGCGTAGACGGTTGTACAAGTCTGATGTGAAAGCCCACGGCTCAACTGTGGGAGTGCATTGGAAACTGTAGAACTAGAGTATCGGAGAGGCAAGTGGAATTCCTAGTGTAGCGGTGAAATGCGTAGATATTAGGAGGAACACCAGTGGCGAAGGCGGCTTGCTGGACGAAAACTGACGTTGAGGCTCGAAAGCGTGGGGAGCAAACAGG
ASV286	Bacteria	Firmicutes	Clostridia	Lachnospirales	Lachnospiraceae	XBB1006	TACGTAGGGGGCAAGCGTTATCCGGATTTACTGGGTGTAAAGGGAGCGCAGGCGGCAGGATGAGTCTGATGTGAAAACCCGCGGCTCAACCACGGGATTGCATTGGAAACTGTCCAGCTAGAGTGTCGGAGAGGTAAGCGGAATTCCTAGTGTAGCGGTGAAATGCGTAGATATTAGGAGGAACACCAGTGGCGAAGGCGGCTTACTGGACGATGACTGACGCTGAGGCTCGAAAGCGTGGGGAGCAAACAGG
ASV571	Bacteria	Firmicutes	Clostridia	Lachnospirales	Lachnospiraceae	Lachnospiraceae_Genus	TACGTAGGGGGCAAGCGTTATCCGGATTTACTGGGTGTAGAGGGAGCGCAGGCGGCAGGATGAGTCTGATGTGAAAACCCGCGGCTCAACCACGGGATTGCATTGGAAACTGTAGAACTAGAGTATCGGAGAGGCAAGTGGAATTCCTAGTGTAGCGGTGAAATGCGTAGATATTAGGAGGAACACCAGTGGCGAAGGCGGCTTGCTGGACGAAAACTGACGTTGAGGCTCGAAAGCGTGGGGAGCAAACAGG
ASV069	Bacteria	Firmicutes	Clostridia	Lachnospirales	Lachnospiraceae	Lachnospiraceae_Genus	TACGTAGGGGGCAAGCGTTATCCGGATTTACTGGGTGTAAAGGGAGCGTAGACGGCCATGCAAGTCTGATGTGAAAACCCGGGGCTCAACCCCGGGACTGCATTGGAAACTGTGCGGCTGGAGTGTCGGAGAGGCAAGTGGAATTCCTAGTGTAGCGGTGAAATGCGTAGATATTAGGAGGAACACCAGTGGCGAAGGCGGCTTGCTGGACGACGACTGACGTTGAGGCTCGAAAGCGTGGGGAGCAAACAGG
ASV	Bacteria	Firmicutes	Clostridia	Lachnospirales	Lachnospiraceae	Lachnospiraceae_UCG-006	TACGTATGGTGCAAGCGTTATCCGGATTTACTGGGTGTAAAGGGAGCGTAGACGGCTGTGCAAGTCTGAAGTGAAAGGCCGGGGCCCAACCCCGGGAGTGCTTTGGAAACTGTGCGGCTAGAGTGTCGGAGAGGCAAGTGGAATTCCCAGTGTAGCGGTGAAATGCGTAGATATTGGGAGGAACACCAGTGGCGAAGGCGTCTTGCTGGACGATGACTGACGTTGAGGCTCGAAAGCGTGGGGAGCAAACAGG

188							
ASV114	Bacteria	Firmicutes	Clostridia	Lachnospirales	Lachnospiraceae	Dorea	TACGTAGGGGGCAAGCGTTATCCGGATTTACTGGGTGTAAAGGGAGCGTAGACGGCTGTGCAAGTCTGGAGTGAAAGGCATGGGCTCAACCTGTGGACTGCTTTGGAAACTGTGCGGCTTGAGTGTCGGAGAGGCAAGTGGAATTCCTAGTGTAGCGGTGAAATGCGTAGATATTAGGAGGAACACCAGTGGCGAAGGCGGCTTGCTGGACGATGACTGACGTTGAGGCTCGAAAGCGTGGGGAGCAAACAGG
ASV215	Bacteria	Firmicutes	Clostridia	Lachnospirales	Lachnospiraceae	Lachnospiraceae_UCG-006	TACGTAGGGGGCAAGCGTTATCCGGATTTACTGGGTGTAAAGGGAGCGTAGACGGCAGTGCAAGTCTGAAGTGAAAGGCAAGGGCTCAACCCTTGGACTGCTTTGGAAACTGTGCTGCTGGAGTGTCGGAGAGGCAAGTGGAATTCCTAGTGTAGCGGTGAAATGCGTAGATATTAGGAGGAACACCAGTGGCGAAGGCGGCTTGCTGGACGATGACTGACGTTGAGGCTCGAAAGCGTGGGGAGCAAACAGG
ASV326	Bacteria	Firmicutes	Clostridia	Lachnospirales	Lachnospiraceae	Lachnoclostridium	TACGTAGGGGGCAAGCGTTATCCGGATTTACTGGGTGTAAAGGGAGCGTAGACGGCGATGCAAGCCAGATGTGAAAGCCCGGGGCTCAACCCCGGGACTGCATTTGGAACTGTGTGGCTGGAGTGTCGGAGAGGCAAGTGGAATTCCTAGTGTAGCGGTGAAATGCGTAGATATTAGGAGGAACACCAGTGGCGAAGGCGGCTTGCTGGACGATGACTGACGTTGAGGCTCGAAAGCGTGGGGAGCAAACAGG
ASV379	Bacteria	Firmicutes	Clostridia	Lachnospirales	Lachnospiraceae	Lachnospiraceae_Genus	TACGTAGGGGGCAAGCGTTATCCGGATTTACTGGGTGTAAAGGGAGCGTAGACGGCTTTGCAAGCCAGATGTGAAAGCCCGGGGCTCAACCTCGGGACTGCATTTGGAACTGTATGGCTGGAGTGTCGGAGAGGCAGGCGGAATTCCTAGTGTAGCGGTGAAATGCGTAGATATTAGGAGGAACACCAGTGGCGAAGGCGGCCTGCTGGACGATGACTGACGTTGAGGCTCGAAAGCGTGGGGAGCAAACAGG
ASV245	Bacteria	Firmicutes	Clostridia	Lachnospirales	Lachnospiraceae	Lachnoclostridium	TACGTAGGGGGCAAGCGTTATCCGGATTTACTGGGTGTAAAGGGAGCGTAGACGGCGGTGCAAGCCAGATGTGAAAGCCCGGGGCTCAACCCCGGGACTGCATTTGGAACTGTATGGCTGGAGTGTCGGAGAGGCAGGCGGAATTCCTAGTGTAGCGGTGAAATGCGTAGATATTAGGAGGAACACCAGTGGCGAAGGCGGCCTGCTGGACGATGACTGACGTTGAGGCTCGAAAGCGTGGGGAGCAAACAGG
ASV124	Bacteria	Firmicutes	Clostridia	Lachnospirales	Lachnospiraceae	Lachnoclostridium	TACGTAGGGGGCAAGCGTTATCCGGATTTACTGGGTGTAAAGGGAGCGTAGACGGCGATGCAAGCCAGATGTGAAAGCCCGGGGCTCAACCCCGGGACTGCATTTGGAACTGTATAGCTAGAGTGTCGGAGAGGCAGGCGGAATTCCTAGTGTAGCGGTGAAATGCGTAGATATTAGGAGGAACACCAGTGGCGAAGGCGGCCTGCTGGACGATGACTGACGTTGAGGCTCGAAAGCGTGGGGAGCAAACAGG
ASV295	Bacteria	Firmicutes	Clostridia	Lachnospirales	Lachnospiraceae	Lachnoclostridium	TACGTAGGGGGCAAGCGTTATCCGGATTTACTGGGTGTAAAGGGAGCGTAGACGGCGGTGCAAGCCAGATGTGAAAGCCCGGGGCTCAACCCCGGGACTGCATTTGGAACTGTATGGCTAGAGTGTCGGAGAGGCAGGCGGAATTCCTAGTGTAGCGGTGAAATGCGTAGATATTAGGAGGAACACCAGTGGCGAAGGCGGCCTGCTGGACGATGACTGACGTTGAGGCTCGAAAGCGTGGGGAGCAAACAGG
ASV354	Bacteria	Firmicutes	Clostridia	Lachnospirales	Lachnospiraceae	Lachnoclostridium	TACGTAGGGGGCAAGCGTTATCCGGATTTACTGGGTGTAAAGGGAGCGTAGACGGCCATTCAAGCCAGATGTGAAAGCCCGGGGCTCAACCCCGGGACTGCATTTGGAACTGTATGGCTGGAGTGTCGGAGAGGCAGGCGGAATTCCTAGTGTAGCGGTGAAATGCGTAGATATTAGGAGGAACACCAGTGGCGAAGGCGGCCTGCTGGACGATGACTGACGTTGAGGCTCGAAAGCGTGGGGAGCAAACAGG

ASV254	Bacteria	Firmicutes	Clostridia	Lachnospirales	Lachnospiraceae	Lachnoclostridium	TACGTAGGGGGCAAGCGTTATCCGGATTTACTGGGTGTAAAGGGAGCGTAGACGGTCCTGCAAGCCAGATGTGAAAGCCCGGGGCTCAACCCCGGGACTGCATTTGGAACTGTAAGGCTAGAGTGTCGGAGAGGCAGGCGGAATTCCTAGTGTAGCGGTGAAATGCGTAGATATTAGGAGGAACACCAGTGGCGAAGGCGGCCTGCTGGACGATGACTGACGTTGAGGCTCGAAAGCGTGGGGAGCAAACAGG
ASV352	Bacteria	Firmicutes	Clostridia	Lachnospirales	Lachnospiraceae	Lachnospiraceae_Genus	TACGTAGGGGGCAAGCGTTATCCGGATTTACTGGGTGTAAAGGGAGCGTAGACGGCTTTGCAAGCCAGATGTGAAAGCCCGGGGCTCAACCCCGGGACTGCATTTGGAACTGTGTGGCTAGAGTGTCGGAGAGGCAGGCGGAATTCCTAGTGTAGCGGTGAAATGCGTAGATATTAGGAGGAACACCAGTGGCGAAGGCGGCCTGCTGGACGATGACTGACGTTGAGGCTCGAAAGCGTGGGGAGCAAACAGG
ASV083	Bacteria	Firmicutes	Clostridia	Lachnospirales	Lachnospiraceae	Lachnoclostridium	TACGTAGGGGGCAAGCGTTATCCGGATTTACTGGGTGTAAAGGGAGCGTAGACGGCGGTGCAAGCCAGATGTGAAAGCCCGGGGCTCAACCCCGGGACTGCATTTGGAACTGTGCTGCTAGAGTGTCGGAGAGGCAGGCGGAATTCCTAGTGTAGCGGTGAAATGCGTAGATATTAGGAGGAACACCAGTGGCGAAGGCGGCCTGCTGGACGATGACTGACGTTGAGGCTCGAAAGCGTGGGGAGCAAACAGG
ASV289	Bacteria	Firmicutes	Clostridia	Lachnospirales	Lachnospiraceae	Lachnoclostridium	TACGTAGGGGGCAAGCGTTATCCGGATTTACTGGGTGTAAAGGGAGCGTAGACGGCGGCGCAAGCCAGATGTGAAAGCCCGGGGCTCAACCCCGGGACTGCATTTGGAACTGTGCTGCTAGAGTGTCGGAGAGGCAGGCGGAATTCCTAGTGTAGCGGTGAAATGCGTAGATATTAGGAGGAACACCAGTGGCGAAGGCGGCCTGCTGGACGATGACTGACGTTGAGGCTCGAAAGCGTGGGGAGCAAACAGG
ASV088	Bacteria	Firmicutes	Clostridia	Lachnospirales	Lachnospiraceae	Lachnoclostridium	TACGTAGGGGGCAAGCGTTATCCGGATTTACTGGGTGTAAAGGGAGCGTAGACGGCTTGGCAAGCCAGATGTGAAAGGCTGGGGCTCAACCCCAGGACTGCATTTGGAACTGTCATGCTAGAGTGTCGGAGAGGCAGGCGGAATTCCTAGTGTAGCGGTGAAATGCGTAGATATTAGGAGGAACACCAGTGGCGAAGGCGGCCTGCTGGACGATGACTGACGTTGAGGCTCGAAAGCGTGGGGAGCAAACAGG
ASV181	Bacteria	Firmicutes	Clostridia	Lachnospirales	Lachnospiraceae	Lachnoclostridium	TACGTAGGGGGCAAGCGTTATCCGGATTTACTGGGTGTAAAGGGAGCGTAGACGGCTGTGCAAGCCAGATGTGAAAGCCCGGGGCTCAACCCCGGGACTGCATTTGGAACTGTGTGGCTGGAGTGTCGGAGAGGCAGGCGGAATTCCTAGTGTAGCGGTGAAATGCGTAGATATTAGGAGGAACACCAGTGGCGAAGGCGGCCTGCTGGACGATGACTGACGTTGAGGCTCGAAAGCGTGGGGAGCAAACAGG
ASV156	Bacteria	Firmicutes	Clostridia	Lachnospirales	Lachnospiraceae	Lachnoclostridium	TACGTAGGGGGCAAGCGTTATCCGGATTTACTGGGTGTAAAGGGAGCGTAGACGGCAATGCAAGCCAGATGTGAAAGCCCGGGGCTCAACCCCGGGACTGCATTTGGAACTGTGTAGCTGGAGTGTCGGAGAGGCAGGCGGAATTCCTAGTGTAGCGGTGAAATGCGTAGATATTAGGAGGAACACCAGTGGCGAAGGCGGCCTGCTGGACGATGACTGACGTTGAGGCTCGAAAGCGTGGGGAGCAAACAGG
ASV320	Bacteria	Firmicutes	Clostridia	Lachnospirales	Lachnospiraceae	Lachnoclostridium	TACGTAGGGGGCAAGCGTTATCCGGATTTACTGGGTGTAAAGGGAGCGTAGACGGCTGTGCAAGCCAGATGTGAAAGCCCGGGGCTCAACCCCGGGACTGCATTTGGAACTGTGTAGCTGGAGTGTCGGAGAGGCAGGCGGAATTCCTAGTGTAGCGGTGAAATGCGTAGATATTAGGAGGAACACCAGTGGCGAAGGCGGCCTGCTGGACGATGACTGACGTTGAGGCTCGAAAGCGTGGGGAGCAAACAGG
ASV	Bacteria	Firmicutes	Clostridia	Lachnospirales	Lachnospiraceae	Lachnospiraceae_Genus	TACGTAGGGGGCAAGCGTTATCCGGATTTACTGGGTGTAAAGGGAGCGTAGACGGCATGGCAAGTCTGATGTGAAAACCCGGGGCTCAACCCCGGGACTGCATTGGAAACTGTCAGGCTAGAGTGTCGGAGAGGCAAGTGGAATTCCTAGTGTAGCGGTGAAATGCGTAGATATTAGGAGGAACACCAGTGGCGAAGGCGGCTTGCTGGACGATGACTGACGTTGAGGCTCGAAAGCGTGGGGAGCAAACAGG

281							
ASV106	Bacteria	Firmicutes	Clostridia	Lachnospirales	Lachnospiraceae	Roseburia	TACGTAGGGGGCAAGCGTTATCCGGATTTACTGGGTGTAAAGGGAGCGTAGACGGTATGGCAAGTCTGATGTGAAAACCCGGGGCTCAACCCCGGGACTGCATTGGAAACTGTCGGACTAGAGTGTCGGAGAGGCAAGTGGAATTCCTAGTGTAGCGGTGAAATGCGTAGATATTAGGAGGAACACCAGTGGCGAAGGCGGCTTGCTGGACGATGACTGACGTTGAGGCTCGAAAGCGTGGGGAGCAAACAGG
ASV431	Bacteria	Firmicutes	Clostridia	Lachnospirales	Lachnospiraceae	Lachnospiraceae_UCG-006	TACGTAGGGGGCAAGCGTTATCCGGATTTACTGGGTGTAAAGGGAGCGTAGACGGAATAGCAAGTCTGATGTGAAAACCCGGGGCTCAACTCCGGGAGTGCATTGGAAACTGTTGATCTAGAGTGTCGGAGAGGCAAGCGGAATTCCTAGTGTAGCGGTGAAATGCGTAGATATTAGGAGGAACACCAGTGGCGAAGGCGGCTTGCTGGACGATGACTGACGTTGAGGCTCGAAAGCGTGGGGAGCAAACAGG
ASV097	Bacteria	Firmicutes	Clostridia	Lachnospirales	Lachnospiraceae	Lachnospiraceae_UCG-006	TACGTAGGGGGCAAGCGTTATCCGGATTTACTGGGTGTAAAGGGAGCGTAGACGGAATTGCAAGTCTGATGTGAAAATCCGGGGCCCAACCCCGGAACTGCATTGGAAACTGTATTTCTAGAGTGTCGGAGAGGCAAGTGGAATTCCTGGTGTAGCGGTGAAATGCGTAGATATCAGGAGGAACACCAGTGGCGAAGGCGGCTTGCTGGACGATGACTGACGTTGAGGCTCGAAAGCGTGGGGAGCAAACAGG
ASV1011	Bacteria	Firmicutes	Clostridia	Lachnospirales	Lachnospiraceae	Lachnospiraceae_UCG-006	TACGTAGGGGGCAAGCGTTATCCGGATTTACTGGGTGTAAAGGGAGCGTAGACGGATAAGCAAGTCTGATGTGAAAATCCGGGGCCCAACCCCGGAATTGCATTGGAAACTGCATATCTAGAGTGTCGGAGAGGCAAGTGGAATTCCTGGTGTAGCGGTGAAATGCGTAGATATCAGGAGGAACACCAGTGGCGAAGGCGGCTTGCTGGACGATGACTGACGTTGAGGCTCGAAAGCGTGGGGAGCAAACAGG
ASV142	Bacteria	Firmicutes	Clostridia	Lachnospirales	Lachnospiraceae	Lachnospiraceae_UCG-006	TACGTAGGGGGCAAGCGTTATCCGGATTTACTGGGTGTAAAGGGAGCGTAGACGGACTTGCAAGTCTGATGTGAAAATCCGGGGCCCAACCCCGGAACTGCATTGGAAACTGTATATCTAGAGTGTCGGAGAGGCAAGTGGAATTCCTGGTGTAGCGGTGAAATGCGTAGATATCAGGAGGAACACCAGTGGCGAAGGCGGCTTGCTGGACGATGACTGACGTTGAGGCTCGAAAGCGTGGGGAGCAAACAGG
ASV258	Bacteria	Firmicutes	Clostridia	Lachnospirales	Lachnospiraceae	Lachnospiraceae_Genus	TACGTAGGGGGCAAGCGTTATCCGGATTTACTGGGTGTAAAGGGAGCGTAGACGGAGGAGCAAGTCTGAAGTGAAAGCCCGCGGCTCAACTGCGGGACTGCTTTGGAAACTGTTCATCTAGAGTGTCGGAGAGGTAAGTGGAATTCCTAGTGTAGCGGTGAAATGCGTAGATATTAGGAGGAACACCAGTGGCGAAGGCGGCTTACTGGACGATGACTGACGTTGAGGCTCGAAAGCGTGGGGAGCAAACAGG
ASV159	Bacteria	Firmicutes	Clostridia	Lachnospirales	Lachnospiraceae	Roseburia	TACGTAGGGGGCAAGCGTTATCCGGATTTACTGGGTGTAAAGGGAGCGCAGGCGGCGCGGTAAGTCTGATGTGAAAGCCCGGGGCTCAACCCCGGGACTGCATTGGAAACTGTCGTGCTGGAGTGTCGGAGGGGCAAGTGGAATTCCTAGTGTAGCGGTGAAATGCGTAGATATTAGGAGGAACACCAGTGGCGAAGGCGGCTTGCTGGACGACAACTGACGCTGAGGCTCGAAAGCGTGGGGAGCAAACAGG
ASV045	Bacteria	Firmicutes	Clostridia	Lachnospirales	Lachnospiraceae	Roseburia	TACGTAGGGGGCAAGCGTTATCCGGATTCACTGGGCGTAAAGGGAGCGCAGGCGGCGCGGCAAGTCTGATGTGAAAGCCCGGGGCTCAACCCCGGGTCTGCATTGGAAACTGTCGAGCTAGAGTGTCGGAGGGGTAAGCGGAATTCCTAGTGTAGCGGTGAAATGCGTAGATATTAGGAGGAACACCAGTGGCGAAGGCGGCTTACTGGACGATGACTGACGCTGAGGCTCGAAAGCGTGGGGAGCAAACAGG

ASV309	Bacteria	Firmicutes	Clostridia	Lachnospirales	Lachnospiraceae	Roseburia	TACGTAGGGGGCAAGCGTTATCCGGATTCACTGGGCGTAAAGGGAGCGCAGGCGGCGCGGCAAGTCTGATGTGAAAGCTCGGGGCTCAACCCCGGGTCTGCATTGGAAACTGTCGAGCTAGAGTGTCGGAGGGGTAAGCGGAATTCCTAGTGTAGCGGTGAAATGCGTAGATATTAGGAGGAACACCAGTGGCGAAGGCGGCTTACTGGACGATGACTGACGCTGAGGCTCGAAAGCGTGGGGAGCAAACAGG
ASV024	Bacteria	Firmicutes	Clostridia	Lachnospirales	Lachnospiraceae	Roseburia	TACGTAGGGGGCAAGCGTTATCCGGATTCACTGGGCGTAAAGGGAGCGCAGGCGGCACGGCAAGTCTGGTGTGAAAACCCGGGGCTCAACCCCGGGACTGCATTGGAAACTGCCGGGCTGGAGTGTCGGAGGGGTAAGCGGAATTCCTAGTGTAGCGGTGAAATGCGTAGATATTAGGAGGAACACCAGTGGCGAAGGCGGCTTACTGGACGATGACTGACGCTGAGGCTCGAAAGCGTGGGGAGCAAACAGG
ASV027	Bacteria	Firmicutes	Clostridia	Lachnospirales	Lachnospiraceae	Roseburia	TACGTAGGGGGCGAGCGTTATCCGGATTCACTGGGCGTAAAGGGAGCGCAGGCGGCACGGCAAGTCCGGTGTGAAAGCCCGGGGCCCAACCCCGGGACTGCACTGGAAACTGCCGGGCTGGAGTGTCGGAGGGGCAGGCGGAATTCCTAGTGTAGCGGTGAAATGCGTAGATATTAGGAGGAACACCGGTGGCGAAGGCGGCCTGCTGGACGACGACTGACGCTGAGGCTCGAAAGCGTGGGGAGCAAACAGG
ASV073	Bacteria	Firmicutes	Clostridia	Lachnospirales	Lachnospiraceae	Roseburia	TACGTATGGGGCAAGCGTTATCCGGATTTACTGGGTGTAAAGGGAGCGCAGGCGGCCTGCCAAGTCTGGTGTGAAAACCCGGGGCCCAACCCCGGGCCTGCATTGGAAACTGGCGGGCTGGAGTGTCGGAGGGGCAGGCGGAATTCCTAGTGTAGCGGTGAAATGCGTAGATATTAGGAGGAACACCGGTGGCGAAGGCGGCCTGCTGGACGATGACTGACGCTGAGGCTCGAAAGCGTGGGGAGCAAACAGG
ASV128	Bacteria	Firmicutes	Clostridia	Lachnospirales	Lachnospiraceae	Roseburia	TACGTATGGAGCAAGCGTTATCCGGATTTACTGGGTGTAAAGGGAGCGCAGGCGGTCTGGCAAGTCTGATGTGAAAGGCCGGGGCTCAACTCCGGGACTGCATTGGAAACTGTCAGACTGGAGTGTCGGAGAGGTAAGCGGAATTCCTAGTGTAGCGGTGAAATGCGTAGATATTAGGAGGAACACCAGTGGCGAAGGCGGCTTACTGGACGATGACTGACGCTGAGGCTCGAAAGCGTGGGGAGCAAACAGG
ASV164	Bacteria	Firmicutes	Clostridia	Lachnospirales	Lachnospiraceae	Roseburia	TACGTATGGAGCAAGCGTTATCCGGATTTACTGGGTGTAAAGGGAGCGCAGGCGGTCTGGCAAGTCTGATGTGAAAGGCCGGGGCTCAACCCCGGGACTGCATTGGAAACTGTCAGACTGGAGTGTCGGAGAGGTAAGCGGAATTCCTAGTGTAGCGGTGAAATGCGTAGATATTAGGAGGAACACCAGTGGCGAAGGCGGCTTACTGGACGATGACTGACGCTGAGGCTCGAAAGCGTGGGGAGCAAACAGG
ASV028	Bacteria	Firmicutes	Clostridia	Lachnospirales	Lachnospiraceae	Roseburia	TACGTATGGAGCAAGCGTTATCCGGATTTACTGGGTGTAAAGGGAGCGCAGGCGGTCTGGCAAGTCTGGTGTGAAAGGCCGGGGCTCAACCCCGGGACTGCATTGGAAACTGTCAGACTGGAGTGTCGGAGAGGCAAGCGGAATTCCTAGTGTAGCGGTGAAATGCGTAGATATTAGGAGGAACACCAGTGGCGAAGGCGGCTTGCTGGACGATGACTGACGCTGAGGCTCGAAAGCGTGGGGAGCAAACAGG
ASV090	Bacteria	Firmicutes	Clostridia	Lachnospirales	Lachnospiraceae	Roseburia	TACGTATGGAGCAAGCGTTATCCGGATTTACTGGGTGTAAAGGGAGCGTAGGCGGTAAGGCAAGTCTGATGTGAAAGGCCAGGGCTCAACCCTGGGACTGCATTGGAAACTGTTTAACTGGAGTGTCGGAGAGGCAAGTGGAATTCCTAGTGTAGCGGTGAAATGCGTAGATATTAGGAGGAACACCAGTGGCGAAGGCGGCTTGCTGGACGATGACTGACGCTGAGGCTCGAAAGCGTGGGGAGCAAACAGG
ASV	Bacteria	Firmicutes	Clostridia	Lachnospirales	Lachnospiraceae	Roseburia	TACGTATGGGGCAAGCGTTATCCGGATTTACTGGGTGTAAAGGGAGCGCAGGCGGCTTGGCAAGTCTGATGTGAAAGGCTGGGGCTCAACCCCAGGACTGCATTGGAAACTGTTAGGCTGGAGTGTCGGAGAGGTAAGCGGAATTCCTAGTGTAGCGGTGAAATGCGTAGATATTAGGAGGAACACCAGTGGCGAAGGCGGCTTACTGGACGACAACTGACGCTGAGGCTCGAAAGCGTGGGGAGCAAACAGG

197							
ASV091	Bacteria	Firmicutes	Clostridia	Lachnospirales	Lachnospiraceae	Roseburia	TACGTATGGTGCAAGCGTTATCCGGATTTACTGGGCGTAAAGGGAGCGCAGGCGGCGCGCCAAGTCTGATGTGAAACCCCGGGGCTCAACCCCGGGCATGCATTGGAAACTGGCGGGCTGGAGTGCCGGAGAGGTAAGCGGAATTCCTAGTGTAGCGGTGAAATGCGTAGATATTAGGAGGAACACCAGTGGCGAAGGCGGCTTACTGGACGGCAACTGACGCTGAGGCTCGAAAGCGTGGGGAGCAAACAGG
ASV252	Bacteria	Firmicutes	Clostridia	Lachnospirales	Lachnospiraceae	Roseburia	TACGTATGGTGCAAGCGTTATCCGGATTTACTGGGTGTAAAGGGAGCGCAGGCGGCGCGTTAAGTCTGATGTGAAAACCCGCGGCTCAACTGCGGGACTGCATTGGAAACTGGCGGGCTTGAGTGCCGGAGAGGCAAGCGGAATTCCTAGTGTAGCGGTGAAATGCGTAGATATTAGGAGGAACACCAGTGGCGAAGGCGGCTTGCTGGACGGTAACTGACGCTGAGGCTCGAAAGCGTGGGGAGCAAACAGG
ASV398	Bacteria	Firmicutes	Clostridia	Lachnospirales	Lachnospiraceae	Lachnospiraceae_Genus	TACGTATGGTGCAAGCGTTATCCGGATTTACTGGGTGTAAAGGGAGCGCAGGCGGCGCGTTAAGTCTGATGTGAAAACCCGCGGCTCAACTGCGGGACTGCATTGGAAACTGGCGGGCTTGAGTGCCGGAGAGGCAAGCGGAATTCCTAGTGTAGCGGTGAAATGCGTAGATATTAGGAGGAACACCAGTGGCGAAGGCGGCTTACTGGACTGTAACTGACGTTGAGGCTCGAAAGCGTGGGGAGCAAACAGG
ASV104	Bacteria	Firmicutes	Clostridia	Lachnospirales	Lachnospiraceae	Lachnospiraceae_Genus	TACGTATGGTGCAAGCGTTATCCGGATTTACTGGGTGTAAAGGGAGCGCAGGCGGCGCGGCAAGTCAGATGTGAAAGCCCGGGGCTCAACCCCGGGACTGCATTTGAAACTGCCGGGCTAGAGTATCGGAGAGGCAAGTGGAATTCCTAGTGTAGCGGTGAAATGCGTAGATATTAGGAGGAACACCAGTGGCGAAGGCGGCTTGCTGGACGATCACTGACGCTGAGGCTCGAAAGCGTGGGGAGCAAACAGG
ASV566	Bacteria	Firmicutes	Clostridia	Lachnospirales	Lachnospiraceae	Lachnospiraceae_Genus	TACGTATGGTGCAAGCGTTATCCGGATTTACTGGGTGTAAAGGGAGCGCAGGCGGCGCGGCAAGTCAGATGTGAAAGCCCGGGGCTCAACCCCGGGACTGCATTTGAAACTGCCGGGCTAGAGTATCGGAGAGGCAGGCGGAATTCCTAGTGTAGCGGTGAAATGCGTAGATATTAGGAGGAACACCAGTGGCGAAGGCGGTCTGCTGGACAGCAACTGACGCTGAGGCGCGAAAGCGTGGGGAGCAAACAGG
ASV186	Bacteria	Firmicutes	Clostridia	Lachnospirales	Lachnospiraceae	Roseburia	TACGTATGGTGCAAGCGTTATCCGGATTTACTGGGTGTAAAGGGAGCGCAGGCGGTTCTGCAAGTCTGATGTGAAAGCCCGGGGCTCAACCGCGGGACTGCATTGGAAACTGCGGGACTGGAGTGTCGGAGGGGTAAGCGGAATTCCTGGTGTAGCGGTGAAATGCGTAGATATCAGGAGGAACACCGGTGGCGAAGGCGGCTTACTGGACGATGACTGACGCTGAGGCTCGAAAGCGTGGGGAGCAAACAGG
ASV362	Bacteria	Firmicutes	Clostridia	Lachnospirales	Lachnospiraceae	Roseburia	TACGTATGGTGCAAGCGTTATCCGGATTTACTGGGTGTAAAGGGAGCGCAGGCGGTGCGGCAAGTCTGATGTGAAAGCCCGGGGCTCAACCCCGGGACTGCATTGGAAACTACCGAACTAGAGTGTCGGAGAGGTAAGTGGAATTCCTAGTGTAGCGGTGAAATGCGTAGATATTAGGAGGAACACCAGTGGCGAAGGCGGCTTACTGGACGATAACTGACGCTGAGGCTCGAAAGCGTGGGGAGCAAACAGG
ASV288	Bacteria	Firmicutes	Clostridia	Lachnospirales	Lachnospiraceae	Lachnospiraceae_UCG-001	TACGTATGGAGCAAGCGTTATCCGGATTTACTGGGTGTAAAGGGAGCGCAGGCGGTACGGCAAGTCCGATGTGAAAGCCCGGGGCTCAACCCCGGGACTGCATTGGAAACTGCCGCACTAGAGTGCCGGAGAGGTAAGTGGAATTCCTAGTGTAGCGGTGAAATGCGTAGATATTAGGAGGAACACCAGTGGCGAAGGCGGCTTACTGGACGGTAACTGACGCTGAGGCTCGAAAGCGTGGGGAGCAAACAGG

ASV347	Bacteria	Firmicutes	Clostridia	Lachnospirales	Lachnospiraceae	Lachnospiraceae_UCG-001	TACGTATGGAGCAAGCGTTATCCGGATTTACTGGGTGTAAAGGGAGCGCAGGCGGTACGGCAAGTCCGATGTGAAAACCCGGGGCTCAACCCCGGGACTGCATTGGAAACTGCCGCACTAGAGTGCCGGAGAGGTAAGTGGAATTCCTAGTGTAGCGGTGAAATGCGTAGATATTAGGAGGAACACCAGTGGCGAAGGCGGCTTACTGGACGGTAACTGACGCTGAGGCTCGAAAGCGTGGGGAGCAAACAGG
ASV057	Bacteria	Firmicutes	Clostridia	Lachnospirales	Lachnospiraceae	Roseburia	TACGTATGGTGCAAGCGTTATCCGGATTTACTGGGTGTAAAGGGAGCGCAGGCGGTGCGGCAAGTCTGATGTGAAAGCCCGGGGCCCAACCCCGGTACTGCATTGGAAACTGCCGTACTTGAGTGTCGGAGGGGTAAGTGGAATTCCTAGTGTAGCGGTGAAATGCGTAGATATTAGGAGGAACACCAGTGGCGAAGGCGGCTTACTGGACGACAACTGACGCTGAGGCTCGAAAGCGTGGGGAGCAAACAGG
ASV348	Bacteria	Firmicutes	Clostridia	Lachnospirales	Lachnospiraceae	Agathobacter	TACGTATGGTGCAAGCGTTATCCGGATTTACTGGGTGTAAAGGGAGCGCAGGCGGTGCGGCAAGTCTGATGTGAAAGCCCGGGGCTCAACCCCGGTACTGCATTGGAAACTGTCGTACTAGAGTGTCGGAGGGGTAAGCGGAATTCCTAGTGTAGCGGTGAAATGCGTAGATATTAGGAGGAACACCAGTGGCGAAGGCGGCTTACTGGACGATAACTGACGCTGAGGCTCGAAAGCGTGGGGAGCAAACAGG
ASV125	Bacteria	Firmicutes	Clostridia	Lachnospirales	Lachnospiraceae	Lachnospiraceae_UCG-001	TACGTAGGGGGCAAGCGTTATCCGGATTTACTGGGTGTAAAGGGAGCGCAGGCGGTATGGCAAGTCAGATGTGAAAACCCAGGGCTCAACCCTGGGACTGCATTTGAAACTGCCATACTGGAGTGCCGGAGAGGTAAGTGGAATTCCTAGTGTAGCGGTGAAATGCGTAGATATTAGGAGGAACACCAGTGGCGAAGGCGGCTTACTGGACGGTAACTGACGCTGAGGCTCGAAAGCGTGGGGAGCAAACAGG
ASV173	Bacteria	Firmicutes	Clostridia	Lachnospirales	Lachnospiraceae	Lachnospiraceae_UCG-001	TACGTAGGGGGCAAGCGTTATCCGGATTTACTGGGTGTAAAGGGAGCGCAGGCGGTATGGCAAGTCAGATGTGAAAACCCAGGGCCCAACCCTGGGACTGCATTTGAAACTGCCATACTGGAGTGCCGGAGAGGTAAGTGGAATTCCTAGTGTAGCGGTGAAATGCGTAGATATTAGGAGGAACACCAGTGGCGAAGGCGGCTTACTGGACGGTAACTGACGCTGAGGCTCGAAAGCGTGGGGAGCAAACAGG
ASV308	Bacteria	Firmicutes	Clostridia	Lachnospirales	Lachnospiraceae	Lachnospiraceae_UCG-001	TACGTAGGGGGCGAGCGTTATCCGGATTTACTGGGTGTAAAGGGAGCGCAGGCGGCGTGGCAAGTCAGATGTGAAAACCCGGGGCCCAACCCCGGGACTGCATTTGAAACTGCCATGCTGGAGTGCCGGAGAGGCAAGTGGAATTCCTAGTGTAGCGGTGAAATGCGTAGATATTAGGAGGAACACCAGTGGCGGAGGCGGCTTGCTGGACGGTAACTGACGCTGAGGCTCGAAAGCGTGGGGAGCAAACAGG
ASV036	Bacteria	Firmicutes	Clostridia	Lachnospirales	Lachnospiraceae	Lachnospiraceae_UCG-001	TACGTAGGGGGCAAGCGTTATCCGGATTTACTGGGTGTAAAGGGAGCGCAGGCGGCGTGGCAAGTCAGATGTGAAAACCCGGGGCCCAACCCCGGGAGTGCATTTGAAACTGCCATGCTGGAGTGCCGGAGAGGTAAGCGGAATTCCTAGTGTAGCGGTGAAATGCGTAGATATTAGGAGGAACACCAGTGGCGGAGGCGGCTTACTGGACGGTAACTGACGCTGAGGCTCGAAAGCGTGGGGAGCAAACAGG
ASV327	Bacteria	Firmicutes	Clostridia	Lachnospirales	Lachnospiraceae	Lachnospiraceae_UCG-001	TACGTAGGGGGCAAGCGTTATCCGGATTTACTGGGTGTAAAGGGAGCGCAGGCGGCGTGGCAAGTCAGATGTGAAAACCCGGGGCCCAACCCCGGGAGTGCATTTGAAACTGCCATGCTGGAGTGCCGGAGAGGTAAGTGGAATTCCTAGTGTAGCGGTGAAATGCGTAGATATTAGGAGGAACACCAGTGGCGGAGGCGGCTTACTGGACGGTAACTGACGCTGAGGCTCGAAAGCGTGGGGAGCAAACAGG
ASV	Bacteria	Firmicutes	Clostridia	Lachnospirales	Lachnospiraceae	Lachnospiraceae_Genus	TACGTAGGGGGCAAGCGTTATCCGGATTTACTGGGTGTAAAGGGAGCGCAGGCGGCAGCGCAAGTCTGATGTGAAAGCCCGGGGCCCAACCGCGGGACTGCATTGGAAACTGCGCAGCTGGAGTGCCGGAGGGGTAAGCGGAATTCCTAGTGTAGCGGTGAAATGCGTAGATATTAGGAGGAACACCAGTGGCGAAGGCGGCTTACTGGACGGCAACTGACGCTGAGGCTCGAAAGCGTGGGGAGCAAACAGG

355							
ASV357	Bacteria	Firmicutes	Clostridia	Lachnospirales	Lachnospiraceae	Blautia	TACGTAGGGGGCAAGCGTTATCCGGATTTACTGGGTGTAAAGGGAGCGTAGACGGATGGACAAGTCTGATGTGAAAGGCTGGGGCTCAACCCCGGGACTGCATTGGAAACTGCCCGTCTTGAGTGCCGGAGAGGTAAGCGGAATTCCTAGTGTAGCGGTGAAATGCGTAGATATTAGGAGGAACACCAGTGGCGAAGGCGGCTTACTGGACGGTAACTGACGTTGAGGCTCGAAAGCGTGGGGAGCAAACAGG
ASV053	Bacteria	Firmicutes	Clostridia	Lachnospirales	Lachnospiraceae	Marvinbryantia	TACGTATGGAGCAAGCGTTATCCGGAATTACTGGGTGTAAAGGGAGCGTAGACGGATGTGCAAGTCTGATGTGAAAGGCGGGGGCTCAACCCCTGGACTGCATTGGAAACTGTAAATCTTGAGTGCCGGAGAGGTAAGCGGAATTCCTAGTGTAGCGGTGAAATGCGTAGATATTAGGAGGAACACCAGTGGCGAAGGCGGCTTACTGGACGGTAACTGACGTTGAGGCTCGAAAGCGTGGGGAGCAAACAGG
ASV137	Bacteria	Firmicutes	Clostridia	Lachnospirales	Lachnospiraceae	Marvinbryantia	TACGTATGGAGCAAGCGTTATCCGGAATTACTGGGTGTAAAGGGAGCGTAGACGGATCTGCAAGTCTGATGTGAAAGGCGGGGGCTCAACTCCTGGACTGCATTGGAAACTGTAGATCTTGAGTGCCGGAGAGGTAAGCGGAATTCCTAGTGTAGCGGTGAAATGCGTAGATATTAGGAGGAACACCAGTGGCGAAGGCGGCTTACTGGACGGTAACTGACGTTGAGGCTCGAAAGCGTGGGGAGCAAACAGG
ASV031	Bacteria	Firmicutes	Clostridia	Lachnospirales	Lachnospiraceae	Marvinbryantia	TACGTAGGGGGCAAGCGTTATCCGGATTTACTGGGTGTAAAGGGAGCGTAGACGGCGTGCCAAGTCTGATGTGAAAGGCCGGGGCTCAACCCCGGGACTGCATTGGAAACTGGCATGCTGGAGTGCCGGAGGGGCAAGCGGAATTCCTAGTGTAGCGGTGAAATGCGTAGATATTAGGAGGAACACCGGTGGCGAAGGCGGCTTGCTGGACGGTAACTGACGTTGAGGCTCGAAAGCGTGGGGAGCAAACAGG
ASV290	Bacteria	Firmicutes	Clostridia	Lachnospirales	Lachnospiraceae	Blautia	TACGTAGGGGGCAAGCGTTATCCGGATTTACTGGGTGTAAAGGGAGCGTAGACGGTGTGGCAAGTCTGATGTGAAAGGCATGGGCTCAACCTGTGGACTGCATTGGAAACTGTCATACTTGAGTGCCGGAGGGGTAAGCGGAATTCCTAGTGTAGCGGTGAAATGCGTAGATATTAGGAGGAACACCAGTGGCGAAGGCGGCTTACTGGACGGTAACTGACGTTGAGGCTCGAAAGCGTGGGGAGCAAACAGG
ASV374	Bacteria	Firmicutes	Clostridia	Lachnospirales	Lachnospiraceae	Blautia	TACGTAGGGGGCAAGCGTTATCCGGATTTACTGGGTGTAAAGGGAGCGTAGACGGCGCAGCAAGTCTGATGTGAAAGGCAGGGGCTTAACCCCTGGACTGCATTGGAAACTGCTGTGCTTGAGTGCCGGAGGGGTAAGCGGAATTCCTAGTGTAGCGGTGAAATGCGTAGATATTAGGAGGAACACCAGTGGCGAAGGCGGCTTACTGGACGGTAACTGACGTTGAGGCTCGAAAGCGTGGGGAGCAAACAGG
ASV039	Bacteria	Firmicutes	Clostridia	Lachnospirales	Lachnospiraceae	Lachnospiraceae_NK4A136_group	TACGTAGGGGGCAAGCGTTATCCGGATTTACTGGGTGTAAAGGGAGCGTAGACGGCATTGCAAGTCTGATGTGAAAGATTGGGGCTCAACCCCAGGCCTGCATTGGAAACTGTAAAGCTGGAGTACAGGAGAGGTAAGCGGAATTCCTAGTGTAGCGGTGAAATGCGTAGATATTAGGAGGAACACCAGTGGCGAAGGCGGCTTACTGGACTGTAACTGACGTTGAGGCTCGAAAGCGTGGGGAGCAAACAGG
ASV048	Bacteria	Firmicutes	Clostridia	Lachnospirales	Lachnospiraceae	Lachnospiraceae_Genus	TACGTAGGGGGCAAGCGTTATCCGGATTTACTGGGTGTAAAGGGAGCGTAGACGGCATAGCAAGTCTGATGTGAAAGATCGGGGCTCAACCCCGGGCCTGCATTGGAAACTGTTAAGCTGGAGTACAGGAGAGGTAAGCGGAATTCCTAGTGTAGCGGTGAAATGCGTAGATATTAGGAGGAACACCAGTGGCGAAGGCGGCTTACTGGACTGTAACTGACGTTGAGGCTCGAAAGCGTGGGGAGCAAACAGG

ASV401	Bacteria	Firmicutes	Clostridia	Lachnospirales	Lachnospiraceae	Lachnospiraceae_Genus	TACGTAGGGGGCAAGCGTTATCCGGATTTACTGGGTGTAAAGGGAGCGTAGACGGCATGGCAAGTCTGAAGTGAAAGCCCGGGGCTCAACCGCGGGACTGCTTTGGAAACTGTTAAGCTGGAGTGCAGGAGAGGTAAGTGGAATTCCTAGTGTAGCGGTGAAATGCGTAGATATTAGGAGGAACACCAGTGGCGAAGGCGGCTTACTGGACTGTAACTGACGTTGAGGCTCGAAAGCGTGGGGAGCAAACAGG
ASV264	Bacteria	Firmicutes	Bacilli	Erysipelotrichales	Erysipelotrichaceae	Breznakia	TACGTAGGTGGCAAGCGTTATCCGGAATGATTGGGCGTAAAGGGTGCGTAGGCGGCAAAATAAGTCTGAAGTAAAAGGCGTTGGCTCAACCAACGTAAGCTTTGGAAACTGTTAAGCTAGCGTGCAGGAGAGGTTAGTGGAATTCCATGTGTAGCGGTAAAATGCGTAGATATATGGAGGAACACCAGTGGCGAAGGCGGCTAACTGGCCTGTAACGGACGCTGAGGCACGAAAGCGTGGGGAGCAAATAGG
ASV233	Bacteria	Firmicutes	Bacilli	Erysipelotrichales	Erysipelotrichaceae	Erysipelotrichaceae_Genus	TACGTAGGTGGCGAGCGTTATCCGGAATGATTGGGCGTAAAGGGTGCGCAGGCGGCGCATTAAGTTTGAAGTAAAAGGTAGCGGCTCAACCGCTGCAAGCTTCGAAAACTGGTGTGCTGGAGTGCAGGAGAGGGCAGTGGAATTCCATGTGTAGCGGTAAAATGCGTAGATATATGGAGGAACACCGGTGGCGAAGGCGGCTGCCTGGCCTGTAACTGACGCTGAGGCACGAAAGCGTGGGGAGCAAATAGG
ASV472	Bacteria	Firmicutes	Bacilli	Erysipelotrichales	Erysipelotrichaceae	Holdemanella	TACGTAGGTGGCGAGCGTTATCCGGAATGATTGGGCGTAAAGGGTGCGTAGGTGGCAGAACAAGTCTGGAGTAAAAGGTATGGGCTCAACCCGTACTGGCTCTGGAAACTGTTCAGCTAGAGAACAGAAGAGGACGGCGGAACTCCATGTGTAGCGGTAAAATGCGTAGATATATGGAAGAACACCGGTGGCGAAGGCGGCCGTCTGGTCTGTTGCTGACACTGAAGCACGAAAGCGTGGGGAGCAAATAGG
ASV380	Bacteria	Firmicutes	Bacilli	Erysipelotrichales	Erysipelotrichaceae	Faecalibaculum	TACGTAGGTGGCGAGCGTTATCCGGAATGATTGGGCGTAAAGGGTGCGCAGGCGGTCCTGCAAGTCTGGAGTGAAACGCATGAGCTCAACTCATGCATGGCTTTGGAAACTGGAGGACTGGAGAGCAGGAGAGGGCGGTGGAACTCCATGTGTAGCGGTAAAATGCGTAGATATATGGAAGAACACCAGTGGCGAAGGCGGCCGCCTGGCCTGTTGCTGACGCTGAGGCACGAAAGCGTGGGGAGCAAATAGG
ASV305	Bacteria	Firmicutes	Bacilli	Erysipelotrichales	Erysipelotrichaceae	Erysipelotrichaceae_Genus	TACGTAGGTGGCAAGCGTTATCCGGAATGATTGGGCGTAAAGGGTGCGCAGGCGGATGTATAAGTCTGAAGTAAAAGCCATCGGCTCAACCGATGTAAGCTTTGGAAACTGTAGATCTAGAGTGCAGGAGAGGACAGTGGAATTCCATGTGTAGCGGTAAAATGCGTAGATATATGGAGGAACACCAGTGGCGAAGGCGGCTGTCTGGCCTGTAACTGACGCTGAGGCACGAAAGCGTGGGGAGCAAATAGG
ASV163	Bacteria	Firmicutes	Bacilli	Erysipelotrichales	Erysipelotrichaceae	Erysipelotrichaceae_Genus	TACGTAGGTGGCAAGCGTTATCCGGAATGATTGGGCGTAAAGGGTGCGCAGGCGGAACCATAAGTCTGAAGTAAAAGCCATCGGCTCAACCGATGTAAGCTTTGGAAACTGTGGATCTAGAGTGCAGGAGAGGACAGTGGAATTCCATGTGTAGCGGTAAAATGCGTAGATATATGGAGGAACACCAGTGGCGAAGGCGGCTGTCTGGCCTGTAACTGACGCTGAGGCACGAAAGCGTGGGGAGCAAATAGG
ASV244	Bacteria	Firmicutes	Bacilli	Erysipelotrichales	Erysipelotrichaceae	Erysipelotrichaceae_Genus	TACGTAGGTGGCAAGCGTTATCCGGAATGATTGGGCGTAAAGGGTGCGCAGGCGGAACCATAAGTCTGAAGTAAAAGCCATCGGCTCAACCGATGTAAGCTTTGGAAACTGTGGATCTGGAGTGCAGGAGAGGACAGTGGAATTCCATGTGTAGCGGTAAAATGCGTAGATATATGGAGGAACACCAGTGGCGAAGGCGGCTGTCTGGCCTGTAACTGACGCTGAGGCACGAAAGCGTGGGGAGCAAATAGG
ASV	Bacteria	Firmicutes	Bacilli	Erysipelotrichales	Erysipelatoclostridiaceae	Erysipelatoclostridiaceae_Genus	TACGTATGGAGCAAGCGTTATCCGGATTTACTGGGTGTAAAGGGAGAGTAGGTGGCAGAGCAAGTCCGAAGTGAAAACCCAAAGCTCAACTATGGGAATGCTTTAGAAACTGCTCAGCTAGAGTGCAGTAGAGGATCGTGGAATTCCATGTGTAGCGGTGAAATGCGTAGATATATGGAGGAACACCAGTGGCGAAGGCGACGATCTGGGCTGCAACTGACGCTCAGTCCCGAAAGCGTGGGGAGCAAACAGG

522							
ASV136	Bacteria	Firmicutes	Bacilli	Erysipelotrichales	Erysipelatoclostridiaceae	Erysipelatoclostridiaceae_Genus	TACGTAGGTGGCGAGCGTTATCCGGAATTATTGGGCGTAAAGAGGGAGCAGGCGGCAGCTAAGGTCTGCGGTGAAAGCCCGAAGCTAAACTTCGGTAAGCCGTGGAAACCGAGCAGCTAGAGTGCAGTAGAGGATCGTGGAATTCCATGTGTAGCGGTGAAATGCGTAGATATATGGAGGAACACCAGTGGCGAAGGCGACGATCTGGGCTGCAACTGACGCTCAGTCCCGAAAGCGTGGGGAGCAAATAGG
ASV337	Bacteria	Firmicutes	Bacilli	Erysipelotrichales	Erysipelatoclostridiaceae	Erysipelatoclostridium	TACGTAGGTGGCGAGCGTTATCCGGAATCATTGGGCGTAAAGAGGGAGCAGGCGGCCTGGAGGGTCTATTGTTAAAAGGCAGTGGCTTAACCATTGCAAGGCGAAGAAACCGGCAGGCTAGAGTTCTAAAGAGGATCGTGGAATTCCATGTGTAGCGGTGAAATGCGTAGATATATGGAGGAACACCAGTGGCGAAGGCGACGATCTGGGAAGAAACAGACGCTGAGTCCCGAAAGCGTGGGGAGCAAATAGG
ASV356	Bacteria	Firmicutes	Bacilli	Erysipelotrichales	Erysipelatoclostridiaceae	Erysipelatoclostridium	TACGTAGGTGGCGAGCGTTATCCGGAATCATTGGGCGTAAAGAGGGAGCAGGCGGCCTGGAGGGTCTATTGTTAAAAGGCAATGGCTTAACCATTGTAAGGCGAAGAAACCGGCAGGCTAGAGTTCTAAAGAGGATCGTGGAATTCCATGTGTAGCGGTGAAATGCGTAGATATATGGAGGAACACCAGTGGCGAAGGCGACGATCTGGGAAGAAACAGACGCTGAGTCCCGAAAGCGTGGGGAGCAAATAGG
ASV370	Bacteria	Firmicutes	Bacilli	Lactobacillales	Leuconostocaceae	Weissella	TACGTATGTTCCAAGCGTTATCCGGATTTATTGGGCGTAAAGCGAGCGCAGACGGTTATTTAAGTCTGAAGTGAAAGCCCTCAGCTCAACTGAGGAATTGCTTTGGAAACTGGATGACTTGAGTGCAGTAGAGGAAAGTGGAACTCCATGTGTAGCGGTGAAATGCGTAGATATATGGAAGAACACCAGTGGCGAAGGCGGCTTTCTGGACTGTAACTGACGTTGAGGCTCGAAAGTGTGGGTAGCAAACAGG
ASV249	Bacteria	Firmicutes	Bacilli	Lactobacillales	Streptococcaceae	Streptococcus	TACGTAGGTCCCGAGCGTTATCCGGATTTATTGGGCGTAAAGCGAGCGCAGGCGGTTGGATAAGTCTGAAGTTAAAGGCTGTGGCTCAACCATAGTATGCTTTGGAAACTGTTCAACTTGAGTGCAGAAGGGGAGAGTGGAATTCCATGTGTAGCGGTGGAATGCGTAGATATATGGAGGAACACCGGTGGCGAAAGCGGCTCTCTGGTCTGTAACTGACGCTGAGGCTCGAAAGCGTGGGTAGCGAACAGG
ASV376	Bacteria	Firmicutes	Bacilli	Lactobacillales	Streptococcaceae	Streptococcus	TACGTAGGTCCCGAGCGTTGTCCGGATTTATTGGGCGTAAAGCGAGCGCAGGCGGTTTGATAAGTCTGAAGTTAAAGGCTGTGGCTCAACCATAGTTCGCTTTGGAAACTGTCAAACTTGAGTGCAGAAGGGGAGAGTGGAATTCCATGTGTAGCGGTGAAATGCGTAGATATATGGAGGAACACCGGTGGCGAAAGCGGCTCTCTGGTCTGTAACTGACGCTGAGGCTCGAAAGCGTGGGGAGCGAACAGG
ASV001	Bacteria	Firmicutes	Bacilli	Lactobacillales	Lactobacillaceae	Lactobacillus	TACGTAGGTGGCAAGCGTTGTCCGGATTTATTGGGCGTAAAGCGAGTGCAGGCGGTTCAATAAGTCTGATGTGAAAGCCTTCGGCTCAACCGGAGAATTGCATCAGAAACTGTTGAACTTGAGTGCAGAAGAGGAGAGTGGAACTCCATGTGTAGCGGTGGAATGCGTAGATATATGGAAGAACACCAGTGGCGAAGGCGGCTCTCTGGTCTGCAACTGACGCTGAGGCTCGAAAGCATGGGTAGCGAACAGG
ASV047	Bacteria	Firmicutes	Bacilli	Lactobacillales	Enterococcaceae	Enterococcus	TACGTAGGTGGCAAGCGTTGTCCGGATTTATTGGGCGTAAAGCGAGCGCAGGCGGTTTCTTAAGTCTGATGTGAAAGCCCCCGGCTCAACCGGGGAGGGTCATTGGAAACTGGGAGACTTGAGTGCAGAAGAGGAGAGTGGAATTCCATGTGTAGCGGTGAAATGCGTAGATATATGGAGGAACACCAGTGGCGAAGGCGGCTCTCTGGTCTGTAACTGACGCTGAGGCTCGAAAGCGTGGGGAGCAAACAGG

ASV512	Bacteria	Firmicutes	Bacilli	Lactobacillales	Carnobacteriaceae	Carnobacteriaceae_Genus	TACGTAGGTGGCAAGCGTTGTCCGGATTTATTGGGCGTAAAGCGAGCGCAGGCGGTTTCTTAAGTCTGATGTGAAAGCCCCCGGCTCAACCGGGGAGGGTCATTGGAAACTGGGAGACTTGAGTGCAGAAGGGGAGAGTGGAATTCCATGTGTAGCGGTAGAATGCGTAGATATTAGAAGGAACACCAGTGGCGAAGGCGACTTGCTGGGCCATCACTGACGGTCAGGGACGAAAGCGTGGGGAGCGAATAGG
ASV445	Bacteria	Firmicutes	Bacilli	Bacilli_Order	Bacilli_Order_Family	Bacilli_Order_Family_Genus	TACGTAGGTGGCAAGCGTTATCCGGAATTATTGGGCGTAAAGAGCGCGCAGGTGGTTAATTAAGTCTGATGTGAAAGCCCACGGCTTAACCGTGGAGGGTCATTGGAAACTGTGGAACTGGAGTGCTGGAGAGGCAAGCGGAATTCCTAGTGTAGCGGTGAAATGCGTAGATATCAGGAGGAACACCGGCGGCGAAGGCGGCCTGCTGGACTAAAACTGACGTTGAGGCTCGAAAGCGTGGGGAGCAAACAGG
ASV226	Bacteria	Firmicutes	Bacilli	Erysipelotrichales	Erysipelotrichaceae	Turicibacter	TACGTAGGTGGCAAGCGTTATCCGGAATTATTGGGCGTAAAGAGCGCGCAGGTGGTTAATTAAGTCTGATGTGAAAGCCCACGGCTTAACCGTGGAGGGTCATTGGAAACTGGTTGACTTGAGTGCAGAAGAGGGAAGTGGAATTCCATGTGTAGCGGTGAAATGCGTAGAGATATGGAGGAACACCAGTGGCGAAGGCGGCTTCCTGGTCTGTAACTGACACTGAGGCGCGAAAGCGTGGGGAGCAAACAGG
ASV084	Bacteria	Firmicutes	Bacilli	Erysipelotrichales	Erysipelotrichaceae	Turicibacter	TACGTAGGTGGCAAGCGTTATCCGGAATTATTGGGCGTAAAGAGCGCGCAGGTGGTTGATTAAGTCTGATGTGAAAGCCCACGGCTTAACCGTGGAGGGTCATTGGAAACTGGTTGACTTGAGTGCAGAAGAGGGAAGTGGAATTCCATGTGTAGCGGTGAAATGCGTAGAGATATGGAGGAACACCAGTGGCGAAGGCGGCTTCCTGGTCTGCAACTGACACTGAGGCGCGAAAGCGTGGGGAGCAAACAGG
ASV003	Bacteria	Firmicutes	Bacilli	Erysipelotrichales	Erysipelotrichaceae	Turicibacter	TACGTAGGTGGCAAGCGTTATCCGGAATTATTGGGCGTAAAGAGCGCGCAGGTGGTTAATTAAGTCTGATGTGAAAGCCCACGGCTTAACCGTGGAGGGTCATTGGAAACTGGTTGACTTGAGTGCAGAAGAGGGAAGTGGAATTCCATGTGTAGCGGTGAAATGCGTAGAGATATGGAGGAACACCAGTGGCGAAGGCGGCTTCCTGGTCTGCAACTGACACTGAGGCGCGAAAGCGTGGGGAGCAAACAGG
ASV077	Bacteria	Firmicutes	Bacilli	Erysipelotrichales	Erysipelotrichaceae	Turicibacter	TACGTAGGTGGTAAGCGTTATCCGGAATTATTGGGCGTAAAGAGCGCGCAGGTGGTTAATTAAGTCTGATGTGAAAGCCCACGGCTTAACCGTGGAGGGTCATTGGAAACTGGTTGACTTGAGTGCAGAAGAGGGAAGTGGAATTCCATGTGTAGCGGTGAAATGCGTAGAGATATGGAGGAACACCAGTGGCGAAGGCGGCTTCCTGGTCTGCAACTGACACTGAGGCGCGAAAGCGTGGGGAGCAAACAGG
ASV176	Bacteria	Firmicutes	Bacilli	Staphylococcales	Staphylococcaceae	Staphylococcus	TACGTAGGTGGCAAGCGTTATCCGGAATTATTGGGCGTAAAGCGCGCGTAGGCGGTTTCTTAAGTCTGATGTGAAAGCCCACGGCTCAACCGTGGAGGGTCATTGGAAACTGGGAAACTTGAGTGCAGAAGAGGAAAGTGGAATTCCATGTGTAGCGGTGAAATGCGCAGAGATATGGAGGAACACCAGTGGCGAAGGCGACTTTCTGGTCTGTAACTGACGCTGATGTGCGAAAGCGTGGGGATCAAACAGG
ASV301	Bacteria	Firmicutes	Bacilli	Staphylococcales	Staphylococcaceae	Staphylococcus	TACGTAGGTGGCAAGCGTTATCCGGAATTATTGGGCGTAAAGCGCGCGTAGGCGGTTTCTTAAGTCTGATGTGAAAGCCCACGGCTCAACCGTGGAGGGTCATTGGAAACTGGGGAACTTGAGTGCAGAAGAGGAGAGTGGAATTCCATGTGTAGCGGTGAAATGCGCAGAGATATGGAGGAACACCAGTGGCGAAGGCGGCTCTCTGGTCTGTAACTGACGCTGATGTGCGAAAGCGTGGGGATCAAACAGG
ASV	Bacteria	Firmicutes	Clostridia	Lachnospirales	Lachnospiraceae	Lachnospiraceae_Genus	TACGTAGGGGGCAAGCGTTATCCGGATTTACTGGGTGTAAAGGGAGCGTAGACGGTGCGCCAAGTCTGGAGTGAAATGCCGCAGCTTAACTGCGGAACTGCTTTGGAAACTGGCGAACTAGAGTGCAGAAGAGGAGAGTGGAACTCCATGTGTAGCGGTGGAATGCGTAGATATATGAAGAACACCAGTGGCGAAGGCGGCTCTCTGGTCTGCAACTGACGCTGAGGCTCGAAAGCATGGGTAGCGAACAGG

466							
ASV498	Bacteria	Firmicutes	Clostridia	Lachnospirales	Lachnospiraceae	Lachnospiraceae_Genus	TACGGAGGATGCGAGCGTTATCCGGATTTATTGGGTTTAAAGGGTGCGTAGACGGTGCGCCAAGTCTGGAGTGAAATGCCGCAGCTTAACTGCGGAACTGCTTTGGAAACTGGCGAACTAGAGTGCGGGAGGGGTAAGCGGAATTCCTAGTGTAGCGGTGAAATGCGTAGATATTAGGAGGAACACCAGTGGCGAAGGCGACTTTCTGGACAGTAACTGACGTTGAGGCACGAAAGTGTGGGGAGCAAACAGG
ASV012	Bacteria	Firmicutes	Clostridia	Lachnospirales	Lachnospiraceae	Lachnospiraceae_NK4A136_group	TACGTAGGGGGCAAGCGTTATCCGGATTTACTGGGTGTAAAGGGAGCGTAGACGGTGCGCCAAGTCTGGAGTGAAATGCCGCAGCTTAACTGCGGAACTGCTTTGGAAACTGGCGAACTAGAGTGCGGGAGGGGTAAGCGGAATTCCTAGTGTAGCGGTGAAATGCGTAGATATTAGGAGGAACACCGGTGGCGAAGGCGGCTTACTGGACCGTAACTGACGTTGAGGCTCGAAAGCGTGGGGAGCAAACAGG
ASV093	Bacteria	Firmicutes	Clostridia	Oscillospirales	Ruminococcaceae	Ruminococcaceae_Genus	TACGTAGGGAGCAAGCGTTGTCCGGATTTACTGGGTGTAAAGGGTGCGTAGGCGGCTTTGCAAGTCAGAAGTGAAATCCATGGGCTTAACCCATGAACTGCTTTTGAAACTGCAGAGCTTGAGTGGAGTAGAGGTAGGCGGAATTCCCGGTGTAGCGGTGAAATGCGTAGAGATCGGGAGGAACACCAGTGGCGAAGGCGGCCTGCTGGGCTCTAACTGACGCTGAGGCACGAAAGCGTGGGTAGCAAACAGG
ASV064	Bacteria	Firmicutes	Clostridia	Oscillospirales	Ruminococcaceae	Ruminococcaceae_Genus	TACGTAGGGAGCAAGCGTTGTCCGGATTTACTGGGTGTAAAGGGTGCGTAGGCGGCTTTGCAAGTCAGAAGTGAAATCTATGGGCTTAACCCATAAACTGCTTTTGAAACTGCAGGGCTTGAGTGAAGTAGAGGTAGGCGGAATTCCCGGTGTAGCGGTGAAATGCGTAGAGATCGGGAGGAACACCAGTGGCGAAGGCGGCCTACTGGGCTTTAACTGACGCTGAGGCACGAAAGCGTGGGTAGCAAACAGG
ASV302	Bacteria	Firmicutes	Clostridia	Oscillospirales	Ruminococcaceae	Ruminococcus	TACGTAGGGAGCAAGCGTTGTCCGGATTTACTGGGTGTAAAGGGTGCGTAGGCGGCTTTGCAAGTCAGATGTGAAATCTATGGGCTCAACCCATAAACTGCATTTGAAACTGTAGAGCTTGAGTGAAGTAGAGGCAGGCGGAATTCCCCGTGTAGCGGTGAAATGCGTAGAGATGGGGAGGAACACCAGTGGCGAAGGCGGCCTGCTGGGCTTTAACTGACGCTGAGGCACGAAAGCGTGGGTAGCAAACAGG
ASV499	Bacteria	Firmicutes	Clostridia	Oscillospirales	Ruminococcaceae	CAG-352	TACGTAGGGAGCGAGCGTTGTCCGGATTTACTGGGTGTAAAGGGTGCGTAGGCGGCCGAGCAAGTCAGTTGTGAAAACTATGGGCTTAACCCATAACGTGCAATTGAAACTGTCCGGCTTGAGTGAAGTAGAGGTAGGCGGAATTCCCGGTGTAGCGGTGAAATGCGTAGAGATCGGGAGGAACACCAGTGGCGAAGGCGGCCTACTGGGCTTTAACTGACGCTGAGGCACGAAAGCATGGGTAGCAAACAGG
ASV160	Bacteria	Firmicutes	Clostridia	Oscillospirales	Ruminococcaceae	Ruminococcaceae_Genus	TACGTAGGGAGCGAGCGTTGTCCGGAATTACTGGGTGTAAAGGGTGCGTAGGCGGCCTTGCAAGTCAGGCGTGAAAACCATGGGCTCAACCCGTGGACTGCGTTTGAAACTGTGAGGCTTGAGTGAAGTAGAGGTAGGCGGAATTCCCGGTGTAGCGGTGAAATGCGTAGAGATCGGGAGGAACACCAGTGGCGAAGGCGGCCTACTGGGCTTTAACTGACGCTGAGGCACGAAAGCATGGGTAGCAAACAGG
ASV315	Bacteria	Firmicutes	Clostridia	Oscillospirales	Ruminococcaceae	Ruminococcaceae_Genus	TACGTAGGGAGCGAGCGTTGTCCGGATTTACTGGGTGTAAAGGGTGCGCAGGCGGCTCTGCAAGTCAGGCGTGAAAGCTAGGGGCTCAACCCCTAAACTGCGCTTGAAACTGTGGAGCTTGAGTGAAGTAGAGGCAGGCGGAATTCCCGGTGTAGCGGTGAAATGCGTAGAGATCGGGAGGAACACCAGTGGCGAAGGCGGCCTGCTGGGCTTTAACTGACGCTCAGGCACGAAAGCATGGGTAGCAAACAGG

ASV351	Bacteria	Firmicutes	Clostridia	Oscillospirales	Ruminococcaceae	Ruminococcaceae_Genus	TACGTAGGGAGCGAGCGTTGTCCGGATTTACTGGGTGTAAAGGGTGCGCAGGCGGCTCTGCAAGTCAGGCGTGAAAGCTAGGGGCTCAACCCCTAAATTGCGCTTGAAACTGTGGAGCTTGAGTGAAGTAGAGGCAGGCGGAATTCCCGGTGTAGCGGTGAAATGCGTAGAGATCGGGAGGAACACCAGTGGCGAAGGCGGCCTGCTGGGCTTTAACTGACGCTCAGGCACGAAAGCATGGGTAGCAAACAGG
ASV487	Bacteria	Firmicutes	Clostridia	Oscillospirales	Oscillospirales_Family	Oscillospirales_Family_Genus	TACGTAGGGAGCAAGCGTTGTCCGGAATTACTGGGTGTAAAGGGAGCGTAGGCGGGAGGATAAGTTGAATGTGAAATCTATGGGCTCAACCCATAGCTGCGTTCAAAACTGTTCTTCTTGAGTGCAGAAGAGGGAAGCGGAATTCCATGTGTAGCGGTGAAATGCGTAGAGATATGGAGGAACACCAGTGGCGAAGGCGGCTTCCTGGTCTGCAACTGACACTGAGGCGCGAAAGCGTGGGGAGCAAACAGG
ASV078	Bacteria	Firmicutes	Clostridia	Oscillospirales	Ruminococcaceae	Ruminococcaceae_Genus	TACGTAGGGAGCAAGCGTTGTCCGGAATTACTGGGTGTAAAGGGAGCGTAGGCGGGAGGATAAGTTGAATGTGAAATCTATGGGCTCAACCCATAGCTGCGTTCAAAACTGTTCTTCTTGAGTGAAGTAGAGGCAGGCGGAATTCCTAGTGTAGCGGTGAAATGCGTAGATATTAGGAGGAACACCAGTGGCGAAGGCGGCCTGCTGGGCTTTTACTGACGCTGAGGCTCGAAAGCGTGGGTAGCAAACAGG
ASV236	Bacteria	Firmicutes	Clostridia	Oscillospirales	Ruminococcaceae	Ruminococcaceae_Genus	TACGTAGGTGGCAAGCGTTGTCCGGAATTACTGGGTGTAAAGGGAGCGTAGGCGGGAAAGCAAGTTGAATGTTTAAGGTACGGGCTCAACCTGTACAAGCGTTCAAAACTGTTTTTCTTGAGTGGAGTAGAGGTAAGCGGAATTCCTAGTGTAGCGGTGAAATGCGTAAATATTAGGAGGAACACCAGTGGCGAAGGCGGCTTACTGGGCTTTAACTGACGCTGAGGCTCGAAAGCGTGGGTAGCAAACAGG
ASV353	Bacteria	Firmicutes	Clostridia	Oscillospirales	Ruminococcaceae	Ruminococcaceae_Genus	TACGTAGGTGGCAAGCGTTGTCCGGAATTACTGGGTGTAAAGGGAGCGTAGGCGGGAGGATAAGTTGGACGTATAAACTATCGGCTCAACCGGTAGAGGCGTTCAAAACTGTCCTTCTTGAGTGAAGTAGAGGTAAGCGGAATTCCTAGTGTAGCGGTGAAATGCGTAAATATTAGGAGGAACACCAGTGGCGAAGGCGGCTTACTGGGCTTTAACTGACGCTGAGGCTCGAAAGCGTGGGTAGCAAACAGG
ASV123	Bacteria	Firmicutes	Clostridia	Oscillospirales	Ruminococcaceae	Anaerotruncus	TACGTAGGGAGCGAGCGTTGTCCGGAATTACTGGGTGTAAAGGGAGCGTAGGCGGGCGAGAAAGTTGAATGTTAAATCTACCGGCTTAACTGGTAGCTGCGTTCAAAACTTCTTGTCTTGAGTGAAGTAGAGGCAGGCGGAATTCCTAGTGTAGCGGTGAAATGCGTAGATATTAGGAGGAACACCAGTGGCGAAGGCGGCCTGCTGGGCTTTAACTGACGCTGAGGCTCGAAAGCGTGGGGAGCAAACAGG
ASV190	Bacteria	Firmicutes	Clostridia	Oscillospirales	Ruminococcaceae	Anaerotruncus	TACGTAGGGAGCGAGCGTTGTCCGGAATTACTGGGTGTAAAGGGAGCGTAGGCGGGCTTGTAAGTTGAATGTCTAATCCACCGGCTCAACCGGTGATCGCGTTCAAAACTGCAGGTCTTGAGTGAAGTAGAGGCAGGCGGAATTCCTAGTGTAGCGGTGAAATGCGTAGATATTAGGAGGAACACCAGTGGCGAAGGCGGCCTGCTGGGCTTTAACTGACGCTGAGGCTCGAAAGCGTGGGGAGCAAACAGG
ASV474	Bacteria	Firmicutes	Clostridia	Oscillospirales	Ruminococcaceae	Anaerotruncus	TACGTAGGGAGCGAGCGTTGTCCGGAATTACTGGGTGTAAAGGGAGCGTAGGCGGGCTTGTAAGTTGAATGTCTAATCCACCGGCTCAACCGGTGATCGCGTTCAAAACTGCAGGTCTTGAGTGAAGTAGAGGCAGGCGGAATTCCTAGTGTAGCGGTGAAATGCGTAGATATTAGGAGGAACACCAGTGGCGGAGGCGGCTTACTGGACGGTAACTGACGCTGAGGCTCGAAAGCGTGGGGAGCAAACAGG
ASV	Bacteria	Firmicutes	Clostridia	Oscillospirales	Ruminococcaceae	Ruminococcaceae_Genus	TACGTAGGGAGCGAGCGTTGTCCGGAATTACTGGGTGTAAAGGGAGCGTAGGCGGGACTGCAAGTTGGGTGTCAAATCTACCGGCTCAACCGGTAGCCGCACTCAAAACTGCAGTTCTTGAGTGAAGTAGAGGCAGGCGGAATTCCTAGTGTAGCGGTGAAATGCGTAGATATTAGGAGGAACACCAGTGGCGAAGGCGGCCTGCTGGGCTTTTACTGACGCTGAGGCTCGAAAGTGTGGGGAGCAAACAGG

269							
ASV200	Bacteria	Firmicutes	Clostridia	Oscillospirales	Ruminococcaceae	Ruminococcaceae_Genus	TACGTAGGGAGCAAGCGTTGTCCGGAATTACTGGGTGTAAAGGGAGCGTAGGCGGGGATGCAAGTTGGGTGTCAAAACTACCGGCTCAACCGATAGTCGCACTCAAAACTGCAGCTCTTGAGTGAAGTAGAGGCAGGCGGAATTCCTAGTGTAGCGGTGAAATGCGTAGATATTAGGAGGAACACCAGTGGCGAAGGCGGCCTGCTGGGCTTTTACTGACGCTGAGGCTCGAAAGTGTGGGGAGCAAACAGG
ASV132	Bacteria	Firmicutes	Clostridia	Oscillospirales	Ruminococcaceae	Ruminococcaceae_Genus	TACGTAGGGAGCGAGCGTTGTCCGGAATTACTGGGTGTAAAGGGAGCGTAGGCGGGGATGCAAGTTGGGTGTTAAATCTACCGGCTCAACTGGTAGTCGCACTCAAAACTGCAGCTCTTGAGTGAAGTAGAGGCAGGCGGAATTCCTAGTGTAGCGGTGAAATGCGTAGATATTAGGAGGAACACCAGTGGCGAAGGCGGCCTGCTGGGCTTTACTGACGCTGAGGCTCGAAAGTGTGGGGAGCAAACAGG
ASV381	Bacteria	Firmicutes	Clostridia	Oscillospirales	Ruminococcaceae	Ruminococcaceae_Genus	TACGTAGGGAGCAAGCGTTGTCCGGAATTACTGGGTGTAAAGGGAGCGTAGGCGGGATGGCAAGTTGGGTGTTAAATCTACCGGCTCAACTGGTAGTCGCACTCAAAACTGCTATTCTTGAGTGAAGTAGAGGCAGGCGGAATTCCTAGTGTAGCGGTGAAATGCGTAGATATTAGGAGGAACACCAGTGGCGAAGGCGGCCTGCTGGGCTTTACTGACGCTGAGGCTCGAAAGTGTGGGGAGCAAACAGG
ASV247	Bacteria	Firmicutes	Clostridia	Oscillospirales	Ruminococcaceae	Anaerotruncus	TACGTAGGGAGCGAGCGTTGTCCGGAATTACTGGGTGTAAAGGGAGCGTAGGCGGGGATGCAAGTTGGATGTTTAAACTATCGGCTCAACCGGTAATTGCAACCAAAACTGCAACTCTTGAGTGAAGTAGAGGCAGGCGGAATTCCTAGTGTAGCGGTGAAATGCGTAGATATTAGGAGGAACACCAGTGGCGAAGGCGGCCTGCTGGGCTTTAACTGACGCTGAGGCTCGAAAGCGTGGGGAGCAAACAGG
ASV322	Bacteria	Firmicutes	Clostridia	Oscillospirales	Ruminococcaceae	Ruminococcaceae_Genus	TACGTAGGTGGCGAGCGTTGTCCGGAATTACTGGGTGTAAAGGGAGCGTAGGCGGGATATTAAGTTGAATGTATAAACTACCGGCTTAACCGATAGAAGCGTTCAAAACTGATATTCTTGAGTGAAGTAGAGGCAGGCGGAATTCCTAGTGTAGCGGTGAAATGCGTAGATATTAGGAGGAACACCAGTGGCGAAGGCGGCCTGCTGGGCTTTAACTGACGCTGAGGCTCGAAAGCGTGGGTAGCAAACAGG
ASV321	Bacteria	Firmicutes	Clostridia	Oscillospirales	Ruminococcaceae	Ruminococcaceae_Genus	TACGTAGGTGGCGAGCGTTGTCCGGAATTACTGGGTGTAAAGGGAGCGTAGGCGGGAGTGCAAGTTGAATGTATAAACTATCGGCTCAACTGATAGAAGCGTTCAAAACTGCACTTCTTGAGTGAAGTAGAGGCAGGCGGAATTCCTAGTGTAGCGGTGAAATGCGTAGATATTAGGAGGAACACCAGTGGCGAAGGCGGCCTGCTGGGCTTTAACTGACGCTGAGGCTCGAAAGCGTGGGTAGCAAACAGG
ASV334	Bacteria	Firmicutes	Clostridia	Oscillospirales	Ruminococcaceae	Negativibacillus	TACGTAGGTGGCAAGCGTTGTCCGGAATTACTGGGTGTAAAGGGAGCGTAGGCGGGAATGCAAGTTGAATGTTTAAACTATGGGCTCAACCCATAGTCGCGTTCAAAACTGCATTTCTTGAGTGGAGTAGAGGCAGGCGGAATTCCTAGTGTAGCGGTGAAATGCGTAGATATTAGGAGGAACACCAGTGGCGAAGGCGGCCTGCTGGGCTCTAACTGACGCTGAGGCTCGAAAGCGTGGGTAGCAAACAGG
ASV230	Bacteria	Firmicutes	Clostridia	Oscillospirales	Ruminococcaceae	Ruminococcaceae_Genus	TACGTAGGTGGCGAGCGTTGTCCGGAATTACTGGGTGTAAAGGGAGCGTAGGCGGGAGAGCAAGTCGACTGTGAAATCTATGGGCTTAACCCATAGCTGCGATCGAAACTGTTCATCTTGAGTGAAGTAGAGGCAGGCGGAATTCCTAGTGTAGCGGTGAAATGCGTAAATATTAGGAGGAACACCAGTGGCGAAGGCGGCCTGCTGGGCTTTAACTGACGCTGAGGCTCGAAAGCGTGGGTAGCAAACAGG

ASV366	Bacteria	Firmicutes	Clostridia	Oscillospirales	Ruminococcaceae	Faecalibacterium	AACGTAGGTCACAAGCGTTGTCCGGAATTACTGGGTGTAAAGGGAGCGCAGGCGGGAAGACAAGTTGGAAGTGAAATCTATGGGCTCAACCCATAAACTGCTTTCAAAACTGTTTTTCTTGAGTAGTGCAGAGGTAGGCGGAATTCCCGGTGTAGCGGTGGAATGCGTAGATATCGGGAGGAACACCAGTGGCGAAGGCGGCCTACTGGGCACCAACTGACGCTGAGGCTCGAAAGTGTGGGTAGCAAACAGG
ASV373	Bacteria	Firmicutes	Clostridia	Oscillospirales	Ruminococcaceae	Faecalibacterium	AACGTAGGTCACAAGCGTTGTCCGGAATTACTGGGTGTAAAGGGAGCGCAGGCGGGAAGACAAGTTGGAAGTGAAATCCATGGGCTCAACCCATGAACTGCTTTCAAAACTGTTTTTCTTGAGTAGTGCAGAGGTAGGCGGAATTCCCGGTGTAGCGGTGGAATGCGTAGATATCGGGAGGAACACCAGTGGCGAAGGCGGCCTACTGGGCACCAACTGACGCTGAGGCTCGAAAGTGTGGGTAGCAAACAGG
ASV473	Bacteria	Firmicutes	Clostridia	Oscillospirales	Ruminococcaceae	Subdoligranulum	AACGTAGGGTGCAAGCGTTGTCCGGAATTACTGGGTGTAAAGGGAGCGCAGGCGGGAAGACAAGTTGGAAGTGAAAACCATGGGCTCAACCCATGAATTGCTTTCAAAACTGTTTTTCTTGAGTAGTGCAGAGGTAGATGGAATTCCCGGTGTAGCGGTGGAATGCGTAGATATCGGGAGGAACACCAGTGGCGAAGGCGGTCTACTGGGCACCAACTGACGCTGAGGCTCGAAAGCATGGGTAGCAAACAGG
ASV074	Bacteria	Firmicutes	Clostridia	Oscillospirales	Ruminococcaceae	Ruminococcaceae_Genus	TACGTAGGTGGCAAGCGTTGTCCGGAATTACTGGGTGTAAAGGGAGCGTAGGCGGGATGTCAAGTCAGAAGTGAAAATTATGGGCTCAACTCATAACCTGCTTTTGAAACTGATATTCTTGAGTGAAGTAGAGGCAAGCGGAATTCCTAGTGTAGCGGTGAAATGCGTAGATATTAGGAGGAACACCAGTGGCGAAGGCGGCTTGCTGGGCTTTTACTGACGCTGAGGCTCGAAAGCGTGGGGAGCAAACAGG
ASV427	Bacteria	Firmicutes	Clostridia	Lachnospirales	Lachnospiraceae	Lachnospiraceae_Genus	TACGGAGGATGCGAGCGTTATCCGGATTTATTGGGTTTAAAGGGTGCGTAGGCGGTCCGTTAAGTCAGCGGTAAAATTGCGGGGCTCAACCCCGGACTGCATTGGAAACTGGCATGCTGGAGTGCCGGAGGGGCAAGCGGAATTCCTAGTGTAGCGGTGAAATGCGTAGATATTAGGAGGAACACCGGTGGCGAAGGCGGCTTACTGGACCGTAACTGACGTTGAGGCTCGAAAGCGTGGGGAGCAAACAGG
ASV509	Bacteria	Firmicutes	Clostridia	Lachnospirales	Lachnospiraceae	Lachnospiraceae_Genus	TACGGAGGATGCGAGCGTTATCCGGATTTATTGGGTTTAAAGGGTGCGTAGGCGGTCCGTTAAGTCAGCGGTAAAATTGCGGGGCTCAACCCCGGGGCTGCATTGGGAACTGCCAGGCTGGAGTGCCGGAGAGGCAGGCGGAATTCCTAGTGTAGCGGTGAAATGCGTAGATATTAGGAGGAACACCAGTGGCGAAGGCGGCTCACTGGACTGTAACTGACGTTGAGGCTCGAAAGCGTGGGGAGCAAACAGG
ASV476	Bacteria	Firmicutes	Clostridia	Oscillospirales	Oscillospiraceae	Pseudoflavonifractor	TACGGAGGATGCGAGCGTTATCCGGATTTATTGGGTTTAAAGGGTGCGTAGGCGGGGAGCCAAGTCAGCGGTAAAATCGTGGGGCTCAACCCCATCCCGCCGTTGAAACTGTATTTCTTGAGTGCTGGAGAGGCAATCGGAATTCCGTGTGTAGCGGTGAAATGCGTAGATATACGGAGGAACACCAGTGGCGAAGGCGGATTGCTGGACAGTAACTGACGCTGAGGCACGAAAGCGTGGGTAGCAAATAGG
ASV577	Bacteria	Bacteria_Phylum	Bacteria_Phylum_Class	Bacteria_Phylum_Class_Order	Bacteria_Phylum_Class_Order_Family	Bacteria_Phylum_Class_Order_Family_Genus	TACGGAGGATGCGAGCGTTATCCGGATTTATTGGGTTTAAAGGGTGCGTAGGCGGGCTTTTAAGTCAGCGGTAAAAATCCGGGGCCCAACCCCGGAACTGCATTGGAAACTGTATTTCTAGAGTGTCGGAGAGGCAGACGGAATTCCTTGTGTAGCGGTGAAATGCGTAGATATAAGGAGGAACACCAGTGGCGAAGGCGGTCTGCTGGACAGCAACTGACGGTGAGGCGCGAAAGCGTGGGGAGCGAACAGG
ASV	Bacteria	Bacteria_Phylum	Bacteria_Phylum_Class	Bacteria_Phylum_Class_Order	Bacteria_Phylum_Class_Order_Family	Bacteria_Phylum_Class_Order_Family_Genus	TACGGAGGATGCGAGCGTTATCCGGATTTATTGGGTTTAAAGGGTGCGCAGGCGGAAGATCAAGTCAGCGGTAAAATTGAGAGGCTCAACCTCTTCGAGCCGTTGAAACTGGTTGACTTGAGTGCAGAAGAGGGAAGTGGAATTCCATGTGTAGCGGTGAAATGCGTAGAGATATGGAAGAACACCAGTGGCGAAGGCGGCTCTCTGGTCTGCAACTGACGCTGAGGCTCGAAAGCATGGGTAGCGAACAGG

569							
ASV588	Bacteria	Firmicutes	Clostridia	Oscillospirales	Oscillospirales_Family	Oscillospirales_Family_Genus	TACGGAGGATGCGAGCGTTATCCGGATTTATTGGGTTTAAAGGGTGCGCAGGCGGAGGTGCAAGTCAGCGGTCAAATTGCGGGGCTCAACCTCGTACTGCCGTTGAAACTGCAGAGCTTGAGTGGAGTAGAGGTAGGCGGAATTCCCGGTGTAGCGGTGAAATGCGTAGAGATCGGGAGGAACACCAGTGGCGAAGGCGGCCTGCTGGGCTCTAACTGACGCTGAGGCACGAAAGCGTGGGTAGCAAACAGG
ASV442	Bacteria	Bacteroidota	Bacteroidia	Bacteroidales	Bacteroidales_Family	Bacteroidales_Family_Genus	TACGGAGGATGCGAGCGTTATCCGGATTTATTGGGTTTAAAGGGTGCGTAGGCGGGCTTTTAAGTCAGCGGTAAAAATTCGGGGCTCAACCCCGTCCGGCCGTTGAAACTGGGGGCCTTGAGTGAAGTGGAGGTTGGCGGAATTCCGAGTGTAGCGGTGAAATGCGTAGATATTCGGAGGAACACCGGTGGCGAAGGCGGCCAACTGGGCTTTAACTGACGCTGAGGCTCGAAAGTGTGGGGAGCAAACAGG
ASV450	Bacteria	Bacteroidota	Bacteroidia	Bacteroidales	Muribaculaceae	Muribaculaceae_Genus	TACGGAGGATGCGAGCGTTATCCGGATTTATTGGGTTTAAAGGGTGCGTAGGCGGATTTTTAAGTCAGCGGTAAAATGTCCGGGCTCAACCCGGGCCGGCCGTTGAAACTGGGGATCTTGAGTGGGCGAGAAGTATGCGGAATGCGTGGTGTAGCGGTGAAATGCGTAGATATTAGGGGGAACACCAGTGGCGAAGGCGGCCTGCTGGACTGCAACTGACGTTGAGGCTCGAAAGCGTGGGGATCGAACAGG
ASV411	Bacteria	Bacteroidota	Bacteroidia	Bacteroidales	Bacteroidales_Family	Bacteroidales_Family_Genus	TACGGAGGATGCGAGCGTTATCCGGATTTATTGGGTTTAAAGGGTGGGCAGGCGGCGAGTTAAGTCAGCGGTAAAATTTCGGGGCTCAACCCCGACAAGCCGTTGAAACTGGCTTGCTAGAGTGGGCGAGAAGTATGCGGAATGCGTGGTGTAGCGGTGAAATGCATAGATATTAGGAGGAACACCAGTGGCGAAGGCGGCTTACTGGACGATGACTGACGCTGAGGCTCGAAAGCGTGGGGAGCAAACAGG
ASV433	Bacteria	Bacteroidota	Bacteroidia	Bacteroidales	Bacteroidales_Family	Bacteroidales_Family_Genus	TACGGAGGATGCGAGCGTTATCCGGATTTATTAGGTTTAAAGGGTGCGCAGGCGGCGAGTTAAGTCAGCGGTAAAATTTCGGGGCTCAACCCCGACAAGCCGTTGAAACTGGCTTGCTAGAGTGGGCGAGAAGTATGCGGAATGCGTGGTGTAGCGGTGAAATGCGTAGATATTAGGAGGAACACCAGTGGCGAAGGCGGCCTGCTGGACTGCAACTGACGTTGAGGCTCGAAGGCGTGGGGAGCAAACAGG
ASV471	Bacteria	Firmicutes	Clostridia	Lachnospirales	Lachnospiraceae	Lachnospiraceae_Genus	TACGGAGGATGCGAGCGTTATCCGGATTTATTGGGTTTAAAGGGTGCGCAGGCGGCGAGTTAAGTCAGCGGTAAAATTTCGGGGCTCAACCCCGACAAGCCATTGAAACTGGCTTGCTAGAGTGGGCGAGAAGTATGCGGAATTCCTAGTGTAGCGGTGAAATGCGTAGATATTAGGAGGAACACCGGTGGCGAAGGCGGCTTACTGGACTGCAACTGACGTTGAGGCTCGAAGGCGTGGGGAGCAAACAGG
ASV425	Bacteria	Bacteroidota	Bacteroidia	Bacteroidales	Bacteroidales_Family	Bacteroidales_Family_Genus	TACGGAGGATGCGAGCGTTATCCGGATTTATTGGGTTTAAAGGGTGCGCAGGCGGAGGTGCAAGTCAGCGGTCAAATTGCGGGGCTCAACCCCGTACTGCCGTTGAAACTGCATCCCTTGAGTGCGCGAGAAGTATGCGGAATGCGTGGTGTAGCGGTGAAATGCGTAGATATTAGGAAGAACACCAGTGGCGAAGGCGGCCTGCTGGACTGCAACTGACGTTGAGGCTCGAAGGCGTGGGGAGCAAACAGG
ASV383	Bacteria	Bacteroidota	Bacteroidia	Bacteroidales	Bacteroidales_Family	Bacteroidales_Family_Genus	TACGGAGGATGCGAGCGTTATCCGGATTTATTGGGTTTAAAGGGTGCGTAGACGGAGCGTTAAGTCAGTGGTCAAATTGAGGGGCTCAACCCCTTCCCGCCATTGAAACTGGCGCTCTTGAGTGGAAGAGAAGTATGCGGAATGCGTGGTGTAGCGGTGAAATGCGTAGATATTAGGAGGAACACCAGTGGCGAAGGCGGCCTGCTGGACGGTGACTGACGCTGAGGCTCGAAAGCGTGGGGAGCAAACAGG

ASV410	Bacteria	Bacteroidota	Bacteroidia	Bacteroidales	Muribaculaceae	Muribaculum	TACGGAGGATGCGAGCGTTATCCGGATTTATTGGGTTTAAAGGGTGCGTAGGCGGCTTTTAAGTCAGCGGTAAAAATTCGGGGCTCAACCCCGTCCGGCCGTTGAAACTGGGGGCCTTGAGTGGGCGAGAAGAAGGCGGAATGCGTGGTGTAGCGGTGAAATGCATAGATATTAGGAGGAACACCAGTGGCGAAGGCGGCTTTCTGGACTGAAACTGACGCTGAGGCTCGAAAGCGTGGGGAGCGAACAGG
ASV563	Bacteria	Bacteroidota	Bacteroidia	Bacteroidales	Bacteroidales_Family	Bacteroidales_Family_Genus	TACGGAGGATGCGAGCGTTATCCGGATTTATTGGGTTTAAAGGGTGCGTAGGCGGCTTTTAAGTCAGCGGTAAAAATTCGGGGCTCAACCCCGTCCGGCCGTTGAAACTGGGGGCCTTGAGTGGGCGGGAAGAAGGCGGAATGCGTGGTGTAGCGGTGAAATGCGTAGATATTAGGAAGAACACCAGTGGCGAAGGCGGCTTGCTGGACAGTAACTGACGTTGAGGCTCGAAAGCGTGGGGAGCAAACAGG
ASV359	Bacteria	Firmicutes	Clostridia	Oscillospirales	Butyricicoccaceae	Butyricicoccus	TACGTAGGGAGCAAGCGTTATCCGGATTTACTGGGTGTAAAGGGCGAGTAGGCGGGCTGGCAAGTTGGGAGTGAAATCCCGGGGCTTAACCCCGGAACTGCTTTCAAAACTGCTGGTCTTGAGTGATGGAGAGGCAGGCGGAATTCCGTGTGTAGCGGTGAAATGCGTAGATATACGGAGGAACACCAGTGGCGAAGGCGGCCTGCTGGACATTAACTGACGCTGAGGAGCGAAAGCGTGGGGAGCAAACAGG
ASV066	Bacteria	Firmicutes	Clostridia	Oscillospirales	Butyricicoccaceae	Butyricicoccus	TACGTAGGGAGCAAGCGTTATCCGGATTTACTGGGTGTAAAGGGCGTGTAGGCGGGCCTGCAAGTCAGAAGTGAAATCTGGGGGCTTAACCCCCAAACTGCTTTTGAAACTGTGGGTCTTGAGTGATGGAGAGGCAGGCGGAATTCCCAGTGTAGCGGTGAAATGCGTAGATATTGGGAGGAACACCAGTGGCGAAGGCGGCCTGCTGGACATTAACTGACGCTGAGGCGCGAAAGCGTGGGGAGCAAACAGG
ASV239	Bacteria	Firmicutes	Clostridia	Oscillospirales	Butyricicoccaceae	Butyricicoccus	TACGTAGGGAGCAAGCGTTATCCGGATTTACTGGGTGTAAAGGGCGTGTAGGCGGGCCTGCAAGTCAGAAGTGAAATCTGGGGGCTTAACCCCCAAACTGCTTTTGAAACTGTGGATCTTGAGTGATGGAGAGGCAGGCGGAATTCCCAGTGTAGCGGTGAAATGCGTAGATATTGGGAGGAACACCAGTGGCGAAGGCGGCCTGCTGGACATTAACTGACGCTGAGGCGCGAAAGCGTGGGGAGCAAACAGG
ASV505	Bacteria	Firmicutes	Clostridia	Monoglobales	Monoglobaceae	Monoglobus	TACGTAGGTGGCGAGCGTTGTCCGGAATTACTGGGTGTAAAGGGCGAGTAGGCGGACTGACAAGTCAGAAGTGAAATCTAGGGGCTCAACCCCTAAACTGCTTTTGAAACTGTCGGCCTTGAGTGCCGGAGAGGAAAGCGGAATTCCTAGTGTAGCGGTGAAATGCGTAGATATTAGGAGGAACACCAGTGGCGAAGGCGGCTTTCTGGACGGTAACTGACGCTGAGGCGCGAAAGCGTGGGGAGCAAACAGG
ASV325	Bacteria	Firmicutes	Clostridia	Oscillospirales	Oscillospiraceae	UCG-005	TACGTAGGGAGCGAGCGTTGTCCGGATTTACTGGGTGTAAAGGGCGTGTAGGCGGAGCTGCAAGTCAGAAGTGAAATCTCCGGGCTCAACCCGGAAACTGCTTTTGAAACTGTAGCCCTTGAGTATCGGAGAGGCAGGCGGAATTCCTAGTGTAGCGGTGAAATGCGTAGATATTAGGAGGAACACCAGTGGCGAAGGCGGCCTGCTGGACGACAACTGACGCTGAGGCGCGAAAGCGTGGGGAGCAAACAGG
ASV513	Bacteria	Firmicutes	Clostridia	Oscillospirales	Oscillospiraceae	UCG-005	TACGTAGGGAGCGAGCGTTGTCCGGATTTACTGGGTGTAAAGGGCGTGTAGGCGGAGCTGCAAGTCAGAAGTGTAATCTCCGGGCTCAACCCGGAAACTGCTTTTGAAACTGTAGCCCTTGAGTATCGGAGAGGCAGGCGGAATTCCTAGTGTAGCGGTGAAATGCGCAGATATTAGGAGGAACACCAGTGGCGAAGGCGGCTTACTGGACAGTAACTGACGTTGAGGCTCGAAAGCGTGGGGAGCAAACAGG
ASV	Bacteria	Firmicutes	Clostridia	Oscillospirales	Oscillospiraceae	Oscillospiraceae_Genus	TACGTAGGTGGCAAGCGTTATCCGGATTTACTGGGTGTAAAGGGCGTGTAGGCGGGGAAGCAAGTCAGATGTGAAAACCACGGGCTCAACCTGTGGCCTGCATTTGAAACTGTTTTTCTTGAGTGCTGGAGAGGCAATCGGAATTCCGTGTGTAGCGGTGAAATGCGTAGATATACGGAGGAACACCAGTGGCGAAGGCGGATTGCTGGACAGTAACTGACGCTGAGGCGCGAAAGCGTGGGGAGCAAACAGG

115							
ASV323	Bacteria	Firmicutes	Clostridia	Oscillospirales	Oscillospiraceae	Oscillospiraceae_Genus	TACGTAGGTGGCAAGCGTTATCCGGATTTACTGGGTGTAAAGGGCGTGTAGGCGGGGAGGCAAGTCAGATGTGAAAACCACGGGCTCAACCTGTGGCCTGCATTTGAAACTGTTTTTCTTGAGTGCTGGAGAGGCAATCGGAATTCCGTGTGTAGCGGTGAAATGCGTAGATATACGGAGGAACACCAGTGGCGAAGGCGGATTGCTGGACAGTAACTGACGCTGAGGCGCGAAAGCGTGGGGAGCAAACAGG
ASV412	Bacteria	Firmicutes	Clostridia	Oscillospirales	Oscillospiraceae	Oscillospiraceae_Genus	TACGTAGGTGGCAAGCGTTATCCGGATTTATTGGGTGTAAAGGGCGTGTAGGCGGGAAAGCAAGTCAGATGTGAAAACTCAGGGCTCAACCCTGAGCCTGCATTTGAAACTGTTTTTCTTGAGTGCTGGAGAGGCAATCGGAATTCCGTGTGTAGCGGTGAAATGCGTAGATATATGGAAGAACACCAGTGGCGAAGGCGGCTCTCTGGTCTGCAACTGACGCTGAGGCTCGAAAGCATGGGTAGCGAACAGG
ASV151	Bacteria	Firmicutes	Clostridia	Oscillospirales	Oscillospiraceae	Oscillospiraceae_Genus	TACGTAGGTGGCAAGCGTTATCCGGATTTATTGGGTGTAAAGGGCGTGTAGGCGGGAAAGCAAGTCAGATGTGAAAACTCAGGGCTCAACCCTGAGCCTGCATTTGAAACTGTTTTTCTTGAGTGCTGGAGAGGCAATCGGAATTCCGTGTGTAGCGGTGAAATGCGTAGATATACGGAGGAACACCAGTGGCGAAGGCGGATTGCTGGACAGTAACTGACGCTGAGGCGCGAAAGCGTGGGGAGCAAACAGG
ASV144	Bacteria	Firmicutes	Clostridia	Oscillospirales	Oscillospiraceae	Oscillospiraceae_Genus	TACGTAGGTGGCAAGCGTTATCCGGATTTATTGGGTGTAAAGGGCGTGTAGGCGGGAGCGCAAGTCAGATGTGAAAACTCAGGGCTCAACCCTGAGCCTGCATTTGAAACTGTGTTTCTTGAGTGCTGGAGAGGCAATCGGAATTCCGTGTGTAGCGGTGAAATGCGTAGATATACGGAGGAACACCAGTGGCGAAGGCGGATTGCTGGACAGTAACTGACGCTGAGGCGCGAAAGCGTGGGGAGCAAACAGG
ASV055	Bacteria	Firmicutes	Clostridia	Oscillospirales	Oscillospiraceae	Oscillospiraceae_Genus	TACGTAGGTGGCAAGCGTTATCCGGATTTATTGGGTGTAAAGGGCGTGTAGGCGGGAATGCAAGTCAGATGTGAAAACTCAGGGCTCAACCCTGAGCCTGCATTTGAAACTGTATTTCTTGAGTGCTGGAGAGGCAATCGGAATTCCGTGTGTAGCGGTGAAATGCGTAGATATACGGAGGAACACCAGTGGCGAAGGCGGATTGCTGGACAGTAACTGACGCTGAGGCGCGAAAGCGTGGGGAGCAAACAGG
ASV133	Bacteria	Firmicutes	Clostridia	Oscillospirales	Oscillospiraceae	Oscillospiraceae_Genus	TACGTAGGTGGCAAGCGTTATCCGGATTTATTGGGTGTAAAGGGCGTGTAGGCGGGAATGCAAGTCAGATGTGAAAACTATGGGCTCAACCCATAGCCTGCATTTGAAACTGTATTTCTTGAGTGCTGGAGAGGCAATCGGAATTCCGTGTGTAGCGGTGAAATGCGTAGATATACGGAGGAACACCAGTGGCGAAGGCGGATTGCTGGACAGTAACTGACGCTGAGGCGCGAAAGCGTGGGGAGCAAACAGG
ASV279	Bacteria	Firmicutes	Clostridia	Oscillospirales	Oscillospiraceae	Oscillospiraceae_Genus	TACGTAGGTGGCGAGCGTTATCCGGATTTACTGGGTGTAAAGGGCGTGTAGGCGGGATTGCAAGTCAGATGTGAAAACTATGGGCTCAACCCATAGCCTGCATTTGAAACTGTAGTTCTTGAGTGCTGGAGAGGCAATCGGAATTCCGTGTGTAGCGGTGAAATGCGTAGATATACGGAGGAACACCAGTGGCGAAGGCGGATTGCTGGACAGTAACTGACGCTGAGGCGCGAAAGCGTGGGGAGCAAACAGG
ASV182	Bacteria	Firmicutes	Clostridia	Oscillospirales	Oscillospiraceae	Intestinimonas	TACGTAGGTGGCAAGCGTTATCCGGATTTACTGGGTGTAAAGGGCGTGTAGGCGGGAGTGCAAGTCAGATGTGAAAACTATGGGCTCAACCCATAGCCTGCATTTGAAACTGTACTTCTTGAGTGCTGGAGAGGCAATCGGAATTCCGTGTGTAGCGGTGAAATGCGTAGATATACGGAGGAACACCAGTGGCGAAGGCGGATTGCTGGACAGTAACTGACGCTGAGGCGCGAAAGCGTGGGGAGCAAACAGG

ASV1199	Bacteria	Firmicutes	Clostridia	Oscillospirales	Oscillospiraceae	Colidextribacter	TACGTAGGTGGCAAGCGTTATCCGGATTTACTGGGTGTAAAGGGCGTGTAGGCGGGAGAGCAAGTCAGACGTGAAATTCCAGGGCTCAACCCTGGAACTGCGTTTGAAACTGTTCTTCTTGAGTGATGGAGAGGCAGGCGGAATTCCGTGTGTAGCGGTGAAATGCGTAGATATACGGAGGAACACCAGTGGCGAAGGCGGCCTGCTGGACATTAACTGACGCTGAGGCGCGAAAGCGTGGGGAGCAAACAGG
ASV191	Bacteria	Firmicutes	Clostridia	Oscillospirales	Oscillospiraceae	Intestinimonas	TACGTAGGTGGCAAGCGTTATCCGGATTTACTGGGTGTAAAGGGCGTGTAGGCGGGACTGCAAGTCAGATGTGAAATTCCAGGGCTCAACCCTGGAACTGCATTTGAAACTGTAGTTCTTGAGTGATGGAGAGGCAGGCGGAATTCCGTGTGTAGCGGTGAAATGCGTAGATATACGGAGGAACACCAGTGGCGAAGGCGGCCTGCTGGACATTAACTGACGCTGAGGCGCGAAAGCGTGGGGAGCAAACAGG
ASV227	Bacteria	Firmicutes	Clostridia	Oscillospirales	Oscillospiraceae	NK4A214_group	TACGTAGGGAGCAAGCGTTATCCGGATTTACTGGGTGTAAAGGGCGCGTAGGCGGGATGGCAAGTCAGATGTGAAATCCAGGGGCTCAACCCCTGAACTGCATTTGAAACTGTCGTTCTTGAGTACTGGAGAGGTTGACGGAATTCCTAGTGTAGCGGTGAAATGCGTAGATATTAGGAGGAACACCAGTGGCGAAGGCGGTCAACTGGACAGCAACTGACGCTGAGGCGCGAAAGCGTGGGGAGCAAACAGG
ASV152	Bacteria	Firmicutes	Clostridia	Oscillospirales	Oscillospiraceae	Oscillospiraceae_Genus	TACGTAGGTGGCAAGCGTTGTCCGGATTTACTGGGTGTAAAGGGCGTGCAGCCGGGAGTGCAAGTCAGATGTGAAATCCCGCGGCTCAACCGCGGAACTGCATTTGAAACTGTGCTTCTTGAGTGCTGGAGAGGCAGACGGAATTCCTAGTGTAGCGGTGAAATGCGTAGATATTAGGAGGAACACCAGTGGCGAAGGCGGTCTGCTGGACAGTAACTGACGGTGAGGCGCGAAAGCGTGGGGAGCAAACAGG
ASV099	Bacteria	Firmicutes	Clostridia	Oscillospirales	Oscillospiraceae	Oscillospiraceae_Genus	TACGTAGGTGGCAAGCGTTGTCCGGATTTACTGGGTGTAAAGGGCGTGTAGCCGGGTTGACAAGTCAGATGTGAAATCCTGCGGCTTAACCGCAGAACTGCATTTGAAACTGTTGATCTTGAGTACTGGAGAGGCAGACGGAATTCCTAGTGTAGCGGTGAAATGCGTAGATATTAGGAGGAACACCAGTGGCGAAGGCGGTCTGCTGGACAGCAACTGACGGTGAGGCGCGAAAGCGTGGGGAGCAAACAGG
ASV174	Bacteria	Firmicutes	Clostridia	Oscillospirales	Oscillospiraceae	Oscillospiraceae_Genus	TACGTAGGTGGCAAGCGTTGTCCGGATTTACTGGGTGTAAAGGGCGTGCAGCCGGGTTTATAAGTCAGATGTGAAATCCCGCGGCTCAACCGCGGAACTGCATTTGAAACTGTAGATCTTGAGTACTGGAGAGGCAGGCGGAATTCCTAGTGTAGCGGTGAAATGCGTAGATATTAGGAGGAACACCAGTGGCGAAGGCGGCCTGCTGGACAGCAACTGACGGTGAGGCGCGAAAGCGTGGGGAGCAAACAGG
ASV446	Bacteria	Firmicutes	Clostridia	Oscillospirales	Oscillospiraceae	Oscillospiraceae_Genus	TACGTAGGTGGCAAGCGTTGTCCGGATTTACTGGGTGTAAAGGGCGTGCAGCCGGGTTTATAAGTCAGATGTGAAATCCCGCGGCTCAACCGCGGAACTGCATTTGAAACTGTAGATCTTGAGTACTGGAGAGGCAGGCGGAATTCCTAGTGTAGCGGTGAAATGCGTAGATATTAGGAGGAACACCAGTGGCGAAGGCGGCCTGCTGGACTGCAACTGACGTTGAGGCTCGAAGGCGTGGGGAGCAAACAGG
ASV204	Bacteria	Firmicutes	Clostridia	Oscillospirales	Oscillospiraceae	Oscillospiraceae_Genus	TACGTAGGTGGCAAGCGTTGTCCGGATTTACTGGGTGTAAAGGGCGTGTAGCCGGGAAGGCAAGTCAGATGTGAAATCCGGAGGCTCAACCTCCGAATTGCATTTGAAACTGTTTTTCTTGAGTACTGGAGAGGCAGACGGAATTCCTTGTGTAGCGGTGAAATGCGTAGATATAAGGAGGAACACCAGTGGCGAAGGCGGTCTGCTGGACAGCAACTGACGGTGAGGCGCGAAAGCGTGGGGAGCAAACAGG
ASV	Bacteria	Firmicutes	Clostridia	Oscillospirales	Oscillospiraceae	Oscillospiraceae_Genus	TACGTAGGTGGCAAGCGTTGTCCGGATTTACTGGGTGTAAAGGGCGTGTAGCCGGGCTGACAAGTCAGATGTGAAATCCCGTGGCTCAACCACGGAACTGCATTTGAAACTGTCGGTCTTGAGTATCGGAGAGGCAGGCGGAATTCCTAGTGTAGCGGTGAAATGCGTAGATATTAGGAGGAACACCAGTGGCGAAGGCGGCCTGCTGGACGACAACTGACGGTGAGGCGCGAAAGCGTGGGGAGCAAACAGG

273							
ASV421	Bacteria	Firmicutes	Clostridia	Oscillospirales	Oscillospiraceae	Oscillospiraceae_Genus	TACGTAGGTGGCAAGCGTTGTCCGGATTTACTGGGTGTAAAGGGCGTGTAGCCGGGCTGACAAGTCAGATGTGAAATCCCGTGGCTCAACCACGGAACTGCATTTGAAACTGTCGGTCTTGAGTATCGGAGAGGCAGGCGGAATTCCTAGTGTAGCGGTGAAATGCGTAGATATTAGGAGGAACACCAGTGGCGAAGGCGGCCTGCTGGACGGTGACTGACGCTGAGGCTCGAAAGCGTGGGGAGCAAACAGG
ASV110	Bacteria	Firmicutes	Clostridia	Oscillospirales	Oscillospiraceae	Oscillospiraceae_Genus	TACGTAGGTGGCAAGCGTTGTCCGGATTTACTGGGTGTAAAGGGCGTGTAGCCGGGCTGACAAGTCAGATGTGAAATCCGGGGGCTCAACCCCCGAACTGCATTTGAAACTGTTGGTCTTGAGTATCGGAGAGGCAGGCGGAATTCCTAGTGTAGCGGTGAAATGCGTAGATATTAGGAGGAACACCAGTGGCGAAGGCGGCCTGCTGGACGACAACTGACGGTGAGGCGCGAAAGCGTGGGGAGCAAACAGG
ASV168	Bacteria	Firmicutes	Clostridia	Oscillospirales	Oscillospiraceae	Oscillospiraceae_Genus	TACGTAGGTGGCAAGCGTTGTCCGGATTTACTGGGTGTAAAGGGCGTGTAGCCGGGCTGACAAGTCAGATGTGAAATTCCGGGGCTCAACCCCGGACCTGCATTTGAAACTGTTGGTCTTGAGTATCGGAGAGGCAGGCGGAATTCCTAGTGTAGCGGTGAAATGCGTAGATATTAGGAGGAACACCAGTGGCGAAGGCGGCCTGCTGGACGACAACTGACGGTGAGGCGCGAAAGCGTGGGGAGCAAACAGG
ASV271	Bacteria	Firmicutes	Clostridia	Oscillospirales	Oscillospiraceae	Oscillospiraceae_Genus	TACGTAGGTGGCAAGCGTTGTCCGGATTTACTGGGTGTAAAGGGCGTGTAGCCGGGCCGGCAAGTCAGATGTGAAATCCCAAGGCTCAACCTTGGAACTGCATTTGAAACTGTTGGTCTTGAGTATCGGAGAGGCAGGCGGAATTCCTAGTGTAGCGGTGAAATGCGTAGATATTAGGAGGAACACCAGTGGCGAAGGCGGCCTGCTGGACGACAACTGACGGTGAGGCGCGAAAGCGTGGGGAGCAAACAGG
ASV059	Bacteria	Firmicutes	Clostridia	Oscillospirales	Oscillospiraceae	Oscillibacter	TACGTAGGTGGCAAGCGTTGTCCGGATTTACTGGGTGTAAAGGGCGTGCAGCCGGGAAGACAAGTCAGATGTGAAATCCCGCGGCTCAACCGCGGAACTGCATTTGAAACTGTTTTTCTTGAGTACCGGAGAGGTCATCGGAATTCCTTGTGTAGCGGTGAAATGCGTAGATATAAGGAAGAACACCAGTGGCGAAGGCGGATGACTGGACGGCAACTGACGGTGAGGCGCGAAAGCGTGGGGAGCAAACAGG
ASV143	Bacteria	Firmicutes	Clostridia	Oscillospirales	Oscillospiraceae	Oscillibacter	TACGTAGGTGGCAAGCGTTGTCCGGATTTACTGGGTGTAAAGGGCGTGCAGCCGGGCTGACAAGTCAGATGTGAAATCCCGCGGCTCAACCGCGGAACTGCATTTGAAACTGTTAGTCTTGAGTACCGGAGAGGTCATCGGAATTCCTTGTGTAGCGGTGAAATGCGTAGATATAAGGAAGAACACCAGTGGCGAAGGCGGATGACTGGACGGCAACTGACGGTGAGGCGCGAAAGCGTGGGGAGCAAACAGG
ASV229	Bacteria	Firmicutes	Clostridia	Oscillospirales	Oscillospiraceae	Oscillibacter	TACGTAGGTGGCAAGCGTTGTCCGGATTTACTGGGTGTAAAGGGCGTGCAGCCGGGAAGACAAGTCAGATGTGAAATCCCGCGGCTCAACCGCGGAACTGCATTTGAAACTGTTTTTCTTGAGTATCGGAGAGGTAACCGGAATTCCTAGTGTAGCGGTGAAATGCGTAGATATTAGGAAGAACACCAGTGGCGAAGGCGGGTTACTGGACGACAACTGACGGTGAGGCGCGAAAGCGTGGGGAGCAAACAGG
ASV287	Bacteria	Firmicutes	Clostridia	Oscillospirales	Oscillospiraceae	Oscillibacter	TACGTAGGTGGCAAGCGTTGTCCGGATTTACTGGGTGTAAAGGGCGTGCAGCCGGGAAGACAAGTCAGATGTGAAATCCCGCGGCTCAACCGCGGAACTGCATTTGAAACTGTTTTTCTTGAGTATCGGAGAGGTAATCGGAATTCCTAGTGTAGCGGTGAAATGCGTAGATATTAGGAAGAACACCAGTGGCGAAGGCGGATTACTGGACGACAACTGACGGTGAGGCGCGAAAGCGTGGGGAGCAAACAGG

ASV293	Bacteria	Firmicutes	Clostridia	Oscillospirales	Oscillospiraceae	UCG-003	TACGTAGGTGGCAAGCGTTGTCCGGATTTACTGGGTGTAAAGGGCGTGCAGCCGGAGAGGCAAGTCAGATGTGAAATCCGCGGGCTCAACCCGCGAACTGCATTTGAAACTGCTTCCCTTGAGTATCGGAGAGGTAATCGGAATTCCTAGTGTAGCGGTGAAATGCGTAGATATTAGGAAGAACACCAGTGGCGAAGGCGGATTACTGGACGACAACTGACGGTGAGGCGCGAAAGCGTGGGGAGCAAACAGG
ASV205	Bacteria	Firmicutes	Clostridia	Oscillospirales	Oscillospiraceae	Oscillibacter	TACGTAGGTGGCAAGCGTTGTCCGGATTTACTGGGTGTAAAGGGCGTGCAGCCGGGACTGCAAGTCAGATGTGAAATCCGCGGGCTTAACCCGCGAACTGCATTTGAAACTGCGGATCTTGAGTACCGGAGAGGTTATCGGAATTCCTTGTGTAGCGGTGAAATGCGTAGATATAAGGAAGAACACCAGTGGCGAAGGCGGATAACTGGACGGCAACTGACGGTGAGGCGCGAAAGCGTGGGGAGCAAACAGG
ASV248	Bacteria	Firmicutes	Clostridia	Oscillospirales	Oscillospiraceae	Oscillibacter	TACGTAGGTGGCAAGCGTTGTCCGGATTTACTGGGTGTAAAGGGCGTGCAGCCGGGCCGACAAGTCAGATGTGAAATCCGCGGGCTTAACCCGCGAACTGCATTTGAAACTATTGGTCTTGAGTACCGGAGAGGTCATCGGAATTCCTTGTGTAGCGGTGAAATGCGTAGATATAAGGAAGAACACCAGTGGCGAAGGCGGATGACTGGACGGCAACTGACGGTGAGGCGCGAAAGCGTGGGGAGCAAACAGG
ASV278	Bacteria	Firmicutes	Clostridia	Oscillospirales	Oscillospiraceae	Oscillibacter	TACGTAGGTGGCAAGCGTTGTCCGGATTTACTGGGTGTAAAGGGCGTGCAGCCGGAGAGACAAGTCAGATGTGAAATCCGCAGGCTCAACCTGCGAACTGCATTTGAAACTGTTTCCCTTGAGTATCGGAGAGGTCATCGGAATTCCTAGTGTAGCGGTGAAATGCGTAGATATTAGGAAGAACACCAGTGGCGAAGGCGGATGACTGGACGACAACTGACGGTGAGGCGCGAAAGCGTGGGGAGCAAACAGG
ASV231	Bacteria	Firmicutes	Clostridia	Oscillospirales	Oscillospiraceae	Oscillibacter	TACGTAGGTGGCAAGCGTTGTCCGGATTTACTGGGTGTAAAGGGCGTGCAGCCGGAGAGACAAGTCAGATGTGAAATCCGCGGGCTCAACCCGCGAACTGCATTTGAAACTGTTTCCCTTGAGTATCGGAGAGGTCATCGGAATTCCTAGTGTAGCGGTGAAATGCGTAGATATTAGGAAGAACACCAGTGGCGAAGGCGGATGACTGGACGACAACTGACGGTGAGGCGCGAAAGCGTGGGGAGCAAACAGG
ASV058	Bacteria	Firmicutes	Clostridia	Oscillospirales	Oscillospiraceae	Oscillibacter	TACGTAGGTGGCAAGCGTTGTCCGGATTTACTGGGTGTAAAGGGCGTGCAGCCGGAGAGACAAGTCAGATGTGAAATCCACGGGCTCAACCCGTGAACTGCATTTGAAACTGTTTCCCTTGAGTGTCGGAGAGGTAATCGGAATTCCTTGTGTAGCGGTGAAATGCGTAGATATAAGGAAGAACACCAGTGGCGAAGGCGGATTACTGGACGATAACTGACGGTGAGGCGCGAAAGCGTGGGGAGCAAACAGG
ASV275	Bacteria	Firmicutes	Clostridia	Oscillospirales	Oscillospiraceae	Colidextribacter	TACGTAGGTGGCGAGCGTTATCCGGATTTACTGGGTGTAAAGGGCGTGTAGGCGGGAGAGCAAGTCAGATGTGAAAACTACAGGCTTAACCTGTAGCCTGCATTTGAAACTGTTCTTCTTGAGTGATGGAGAGGCAGGCGGAATTCCTAGTGTAGCGGTGAAATGCGTAGATATTAGGAGGAACACCAGTGGCGAAGGCGGCCTGCTGGACATTAACTGACGCTGAGGCGCGAAAGCGTGGGGAGCAAACAGG
ASV405	Bacteria	Firmicutes	Clostridia	Oscillospirales	Oscillospiraceae	Colidextribacter	TACGTAGGTGGCGAGCGTTATCCGGATTTACTGGGTGTAAAGGGCGTGTAGGCGGGAGAGCAAGTCAGATGTGAAAACTACAGGCTTAACCTGTAGCCTGCATTTGAAACTGTTTTTCTTGAGTGATGGAGAGGCAGGCGGAATTCCTAGTGTAGCGGTGAAATGCGTAGATATTAGGAGGAACACCAGTGGCGAAGGCGGCCTGCTGGACATTAACTGACGCTGAGGCGCGAAAGCGTGGGGAGCAAACAGG
ASV	Bacteria	Firmicutes	Clostridia	Oscillospirales	Oscillospiraceae	Oscillospiraceae_Genus	TACGTAGGTGGCGAGCGTTATCCGGATTTACTGGGTGTAAAGGGCGTGTAGGCGGGATTGCAAGTCAGATGTGAAAACTATGGGCTCAACCCATAGCCTGCATTTGAAACTGTAGTTCTTGAGTGCTGGAGAGGCAATCGGAATTCCGTGTGTAGCGGTGAAATGCTTAGATATCACGAAGAACTCCGATTGCGAAGGCAGCCTGCTGGACTGTAACTGACGCTGATGCTCGAAAGTGTGGGTATCAAACAGG

449							
ASV368	Bacteria	Firmicutes	Clostridia	Oscillospirales	Oscillospiraceae	Oscillospiraceae_Genus	TACGTAGGGAGCGAGCGTTATCCGGATTTACTGGGTGTAAAGGGCGTGTAGGCGGGATGGCAAGTCAGGCGTGAAAACTATGGGCTCAACCCATAGCCTGCGTTTGAAACTGTCGTTCTTGAGTGATGGAGAGGCAAGCGGAATTCCGTGTGTAGCGGTGAAATGCGTAGATATACGGAGGAACACCAGTGGCGAAGGCGGCTTGCTGGACATTAACTGACGCTGAGGCGCGAAAGCGTGGGGAGCAAACAGG
ASV147	Bacteria	Firmicutes	Clostridia	Oscillospirales	Oscillospiraceae	Intestinimonas	TACGTAGGTGGCAAGCGTTATCCGGATTTACTGGGTGTAAAGGGCGTGTAGGCGGGACTGCAAGTCAGATGTGAAACCCATGGGCTCAACCCATGGCCTGCATTTGAAACTGTAGTTCTTGAGTGATGGAGAGGCAGGCGGAATTCCGTGTGTAGCGGTGAAATGCGTAGATATACGGAGGAACACCAGTGGCGAAGGCGGCCTGCTGGACATTAACTGACGCTGAGGCGCGAAAGCGTGGGGAGCAAACAGG
ASV126	Bacteria	Firmicutes	Clostridia	Oscillospirales	Butyricicoccaceae	UCG-009	TACGTAGGTGGCAAGCGTTATCCGGATTTACTGGGTGTAAAGGGCGAGTAGGCGGGATTGCAAGTCAGATGTGAAAACTATGGGCTCAACCGATAGAGTGCATTTGAAACTGCAGTTCTTGAGTGATGGAGAGGCAGGCGGAATTCCCGGTGTAGCGGTGGAATGCGTAGATATCGGGAGGAACACCAGTGGCGAAGGCGGCCTGCTGGACATTAACTGACGCTGAGGCGCGAAAGCGTGGGGAGCAAACAGG
ASV109	Bacteria	Firmicutes	Clostridia	Oscillospirales	Oscillospiraceae	Colidextribacter	TACGTAGGTGGCAAGCGTTATCCGGATTTACTGGGTGTAAAGGGCGTGTAGGCGGGACTGCAAGTCAGATGTGAAAACCACGGGCTCAACCTGTGGCCTGCATTTGAAACTGTAGTTCTTGAGTACTGGAGAGGCAGACGGAATTCCTAGTGTAGCGGTGAAATGCGTAGATATTAGGAGGAACACCAGTGGCGAAGGCGGTCTGCTGGACAGCAACTGACGCTGAGGCGCGAAAGCGTGGGGAGCAAACAGG
ASV331	Bacteria	Firmicutes	Clostridia	Oscillospirales	Oscillospiraceae	Colidextribacter	TACGTAGGTGGCAAGCGTTATCCGGATTTACTGGGTGTAAAGGGCGTGTAGGCGGGAGTGCAAGTCAGATGTGAAAACCACGGGCTCAACCTGTGGCCTGCATTTGAAACTGTACTTCTTGAGTACTGGAGAGGCAGACGGAATTCCTAGTGTAGCGGTGAAATGCGTAGATATTAGGAGGAACACCAGTGGCGAAGGCGGTCTGCTGGACAGCAACTGACGCTGAGGCGCGAAAGCGTGGGGAGCAAACAGG
ASV243	Bacteria	Firmicutes	Clostridia	Oscillospirales	Oscillospiraceae	Colidextribacter	TACGTAGGTGGCAAGCGTTATCCGGATTTACTGGGTGTAAAGGGCGTGTAGGCGGGGACGCAAGTCAGATGTGAAAACCACGGGCTCAACCTGTGGCCTGCATTTGAAACTGTGTTTCTTGAGTACTGGAGAGGCAGACGGAATTCCTAGTGTAGCGGTGAAATGCGTAGATATTAGGAGGAACACCAGTGGCGAAGGCGGTCTGCTGGACAGCAACTGACGCTGAGGCGCGAAAGCGTGGGGAGCAAACAGG
ASV081	Bacteria	Firmicutes	Clostridia	Oscillospirales	Oscillospiraceae	Colidextribacter	TACGTAGGTGGCAAGCGTTATCCGGATTTACTGGGTGTAAAGGGCGTGTAGGCGGGAAAGCAAGTCAGATGTGAAAACCATGGGCTCAACCTGTGGCCTGCATTTGAAACTGTTTTTCTTGAGTACTGGAGAGGCAGACGGAATTCCTAGTGTAGCGGTGAAATGCGTAGATATTAGGAGGAACACCAGTGGCGAAGGCGGTCTGCTGGACAGCAACTGACGCTGAGGCGCGAAAGCGTGGGGAGCAAACAGG
ASV372	Bacteria	Firmicutes	Clostridia	Oscillospirales	Oscillospiraceae	Colidextribacter	TACGTAGGTGGCAAGCGTTATCCGGATTTACTGGGTGTAAAGGGCGTGTAGGCGGGAAAGCAAGTCAGATGTGAAAACCATGGGCTCAACCTGTGGCCTGCATTTGAAACTGTTTTTCTTGAGTACTGGAGAGGCAGACGGAATTCCTCGTGTAGCGGTGAAATGCGTAGATATGAGGAGGAACACCAGTGGCGAAGGCGGTCTGCTGGACAGCAACTGACGCTGAGGCGCGAAAGCGTGGGGAGCAAACAGG

ASV192	Bacteria	Firmicutes	Clostridia	Oscillospirales	Oscillospiraceae	Colidextribacter	TACGTAGGTGGCAAGCGTTATCCGGATTTACTGGGTGTAAAGGGCGTGTAGGCGGGACTGCAAGTCAGATGTGAAAACCATGGGCTCAACCTGTGGCCTGCATTTGAAACTGTAGTTCTTGAGTACTGGAGAGGCAGACGGAATTCCTAGTGTAGCGGTGAAATGCGTAGATATTAGGAGGAACACCAGTGGCGAAGGCGGTCTGCTGGACAGCAACTGACGCTGAGGCGCGAAAGCGTGGGGAGCAAACAGG
ASV369	Bacteria	Firmicutes	Clostridia	Oscillospirales	Oscillospiraceae	Colidextribacter	TACGTAGGGGGCAAGCGTTATCCGGATTTACTGGGTGTAAAGGGCGTGTAGGCGGGACTGCAAGTCAGATGTGAAAACCATGGGCTCAACCTGTGGCCTGCATTTGAAACTGTAGTTCTTGAGTACTGGAGAGGCAGACGGAATTCCTAGTGTAGCGGTGAAATGCGTAGATATTAGGAGGAACACCAGTGGCGAAGGCGGCCTGCTGGACGGTGACTGACGCTGAGGCTCGAAAGCGTGGGGAGCAAACAGG
ASV330	Bacteria	Firmicutes	Clostridia	Oscillospirales	Oscillospiraceae	Colidextribacter	TACGTAGGTGGCAAGCGTTATCCGGATTTACTGGGTGTAAAGGGCGTGTAGGCGGGACTGCAAGTCAGATGTGAAAACTCAGGGCTCAACCCTGAGCCTGCATTTGAAACTGTAGTTCTTGAGTACTGGAGAGGCAGACGGAATTCCTAGTGTAGCGGTGAAATGCGTAGATATTAGGAGGAACACCAGTGGCGAAGGCGGTCTGCTGGACAGCAACTGACGCTGAGGCGCGAAAGCGTGGGGAGCAAACAGG
ASV534	Bacteria	Firmicutes	Clostridia	Lachnospirales	Lachnospiraceae	Lachnospiraceae_Genus	TACGTATGGAGCAAGCGTTATCCGGATTTACTGGGCGTAAAGGGAGCGTAGGCGGCCTGCCAAGTCTGATGTGAAATACCGGGGCTCAACCCCAGGCCTGCATTGGAAACTGTAAAGCTGGAGTACAGGAGAGGTAAGCGGAATTCCTAGTGTAGCGGTGAAATGCGTAGATATTAGGAGGAACACCAGTGGCGAAGGCAGCGTACCGGCGCCAGACTGACGCTGAGGCACGAAAGCGTGGGGATCGAACAGG
ASV562	Bacteria	Bacteroidota	Bacteroidia	Bacteroidales	Muribaculaceae	Muribaculaceae_Genus	TACGTATGGTGCAAGCGTTATCCGGATTTACTGGGCGTAAAGGGAGCGCAGGCGGCGCGCCAAGTCTGATGTGAAACCCCGGGGCTCAACCCCGGGCATGCATTGGAAACTGGCAGACTTGAGTTGGCGAGAAGTACGCGGAATGCGCGGTGTAGCGGTGAAATGCATAGATATCGCGCATAACTCCGATTGCGAAGGCAGCATGCCGGCTCCACACTGACGCTGAGGCACGAAAGCGTGGGTATCGAACAGG
ASV440	Bacteria	Bacteroidota	Bacteroidia	Bacteroidales	Muribaculaceae	CAG-873	TACGTAGGGGGCAAGCGTTATCCGGATTTACTGGGTGTAAAGGGAGCGTAGACGGCGTGCCAAGTCTGATGTGAAAGGCCGGGGCTCAACCCCGGGACTGCATTGGAAACTGGCAGACTTGAGTTGGCGAGAAGTACGCGGAATGCGCGGTATAGCGGTGAAATGCATAGATATCGCGCAGAACTCCGATTGCGAAGGCAGCGTACCGGCACCAGACTGACGCTGAGGCACGAAAGCGTGGGGATCGAACAGG
ASV554	Bacteria	Bacteroidota	Bacteroidia	Bacteroidales	Muribaculaceae	CAG-873	TACGTAGGGGGCAAGCGTTATCCGGATTTACTGGGTGTAAAGGGAGCGCAGGCGGCGCGTTAAGCCTGGTGTGAAAGCCCGCGGCCCAACCGCGGGACTGCATTGGAAACTGGCAGACTTGAGTTGGCGAGAAGTACGCGGAATGCGCGGTGTAGCGGTGAAATGCATAGATATCGCGCAGAACTCCGATTGCGAAGGCAGCGTACCGGCGCTAGACTGACGCTGAGGCACGAAAGCGTGGGGATCGAACAGG
ASV589	Bacteria	Bacteroidota	Bacteroidia	Bacteroidales	Tannerellaceae	Parabacteroides	TACGGAGGATGCGAGCGTTATCCGGATTTATTGGGTTTAAAGGGTGCGTAGGTGGTGATTTAAGTCAGCGGTGAAAGTTTGTGGCTCAACCATAAAATTGCCGTTGAAACTGGGTTACTTGAGTGTGTTTGAGGTAGGCGGAATGCGTGGTGTAGCGGTGAAATGCATAGATATCACGCAGAACTCCGATTGCGAAGGCAGCTTACTAAACCATAACTGACACTGAAGCACGAAAGCGTGGGGATCAAACAGG
ASV	Bacteria	Bacteroidota	Bacteroidia	Bacteroidales	Bacteroidaceae	Bacteroides	TACGGAGGATCCGAGCGTTATCCGGATTTATTGGGTTTAAAGGGAGCGTAGATGGATGTTTAAGTCAGTTGTGAAAGTTTGCGGCTCAACCGTAAAATTGCAGTTGATACTGGATATCTTGAGTGCAGTTGAGGCAGGCGGAATTCGTGGTGTAGCGGTGAAATGCTTAGATATCACGAAGAACTCCGATTGCGAAGGCAGCCTGCTAAGCTGCAACTGACATTGAGGCTCGAAAGTGTGGGTATCAAACAGG

343							
ASV365	Bacteria	Bacteroidota	Bacteroidia	Bacteroidales	Bacteroidaceae	Bacteroides	TACGGAGGATCCGAGCGTTATCCGGATTTATTGGGTTTAAAGGGAGCGTAGATGGATGTTTAAGTCAGTTGTGAAAGTTTGCGGCTCAACCGTAAAATTGCAGTTGATACTGGATGTCTTGAGTGCAGTTGAGGCAGGCGGAATTCGTGGTGTAGCGGTGAAATGCTTAGATATCACGAAGAACTCCGATTGCGAAGGCAGCCTGCTAAGCTGCAACTGACATTGAGGCTCGAAAGTGTGGGTATCAAACAGG
ASV441	Bacteria	Bacteroidota	Bacteroidia	Bacteroidales	Bacteroidaceae	Bacteroides	TACGGAGGATCCGAGCGTTATCCGGATTTATTGGGTTTAAAGGGAGCGTAGGCGGACGCTTAAGTCAGTTGTGAAAGTTTGCGGCTCAACCGTAAAATTGCAGTTGATACTGGGTGTCTTGAGTACAGTAGAGGCAGGCGGAATTCGTGGTGTAGCGGTGAAATGCTTAGATATCACGAAGAACTCCGATTGCGAAGGCAGCCTGCTGGACTGTAACTGACGCTGATGCTCGAAAGTGTGGGTATCAAACAGG
ASV011	Bacteria	Bacteroidota	Bacteroidia	Bacteroidales	Bacteroidaceae	Bacteroides	TACGGAGGATCCGAGCGTTATCCGGATTTATTGGGTTTAAAGGGAGCGTAGGCGGGTTGTTAAGTCAGTTGTGAAAGTTTGCGGCTCAACCGTAAAATTGCAGTTGATACTGGCATCCTTGAGTACAGTAGAGGTAGGCGGAATTCGTGGTGTAGCGGTGAAATGCTTAGATATCACGAAGAACTCCGATTGCGAAGGCAGCCTGCTGGACTGTAACTGACGCTGATGCTCGAAAGTGTGGGTATCAAACAGG
ASV430	Bacteria	Bacteroidota	Bacteroidia	Bacteroidales	Prevotellaceae	Prevotella	TACGGAAGGTCCGGGCGTTATCCGGATTTATTGGGTTTAAAGGGAGCGTAGGCCGGAGATTAAGCGTGTTGTGAAATGTAGACGCTCAACGTCTGCACTGCAGCGCGAACTGGTTTCCTTGAGTACGCACAAAGTGGGCGGAATTCGTGGTGTAGCGGTGAAATGCTTAGATATCACGAAGAACTCCGATTGCGAAGGCAGCTCACTGGAGCGCAACTGACGCTGAAGCTCGAAAGTGCGGGTATCGAACAGG
ASV447	Bacteria	Bacteroidota	Bacteroidia	Bacteroidales	Prevotellaceae	Prevotella	TACGGAAGGTCCGGGCGTTATCCGGATTTATTGGGTTTAAAGGGAGCGTAGGCCGGAGATTAAGCGTGTTGTGAAATGTAGATGCTCAACATCTGAACTGCAGCGCGAACTGGTTTCCTTGAGTACGCACAAAGTGGGCGGAATTCGTGGTGTAGCGGTGAAATGCTTAGATATCACGAAGAACTCCGATTGCGAAGGCAGCTCACTGGAGCGCAACTGACGCTGAAGCTCGAAAGTGCGGGTATCGAACAGG
ASV494	Bacteria	Bacteroidota	Bacteroidia	Bacteroidales	Rikenellaceae	Alistipes	TACGGAGGATTCAAGCGTTATCCGGATTTATTGGGTTTAAAGGGTGCGTAGGCGGTTTGATAAGTTAGAGGTGAAATCCCGGGGCTTAACTCCGGAACTGCCTCTAATACTGTTAGACTAGAGAGTAGTTGCGGTAGGCGGAATGTATGGTGTAGCGGTGAAATGCTTAGAGATCATACAGAACACCGATTGCGAAGGCAGCTTACCAAACTATATCTGACGTTGAGGCACGAAAGCGTGGGGAGCAAACAGG
ASV459	Bacteria	Bacteroidota	Bacteroidia	Bacteroidales	Rikenellaceae	Alistipes	TACGGAGGATTCAAGCGTTATCCGGATTTATTGGGTTTAAAGGGTGCGTAGGCGGTTTGATAAGTTAGAGGTGAAATTTCGGGGCTCAACCCTGAACGTGCCTCTAATACTGTTGAGCTAGAGAGTAGTTGCGGTAGGCGGAATGTATGGTGTAGCGGTGAAATGCTTAGAGATCATACAGAACACCGATTGCGAAGGCAGCTTACCAAACTATATCTGACGTTGAGGCACGAAAGCGTGGGGAGCAAACAGG
ASV478	Bacteria	Bacteroidota	Bacteroidia	Bacteroidales	Rikenellaceae	Alistipes	TACGGAGGATCCAAGCGTTATCCGGATTTATTGGGTTTAAAGGGTGCGTAGGCGGTTTGATAAGTTAGAGGTGAAAGCTCGGGGCTTAACTCCGAGACTGCCTCTAATACTGTTGAGCTAGAGAGTAGTTGCGGTAGGCGGAATGTATGGTGTAGCGGTGAAATGCGTAGAGATCATACAGAACACCGATTGCGAAGGCAGCTTACCAAACTATATCTGACGTTGAGGCACGAAAGCGTGGGGAGCAAACAGG

ASV2500	Bacteria	Bacteroidota	Bacteroidia	Bacteroidales	Muribaculaceae	Muribaculaceae_Genus	TACGGAGGATGCGAGCGTTATCCGGATTTATTGGGTTTAAAGGGTGCGCAGGCGGTCTGTTAAGTCAGCGGTCAAAGCCCGGGGCTCAACCCCGGCCCGCCGTTGAAACTGGCAGTCTCGAGTTGGAGAGAAGTATGCGGAATGCGCGGTGTAGCGGTGAAATGCATAGATATCTCGCAGAACTCCGATTGCGAAGGCAGCATGCCGGCTCCACACTGACGCTGAGGCACGAAAGCGTGGGTATCGAACAGG
ASV008	Bacteria	Bacteroidota	Bacteroidia	Bacteroidales	Muribaculaceae	Muribaculaceae_Genus	TACGGAGGATGCGAGCGTTATCCGGATTTATTGGGTTTAAAGGGTGCGCAGGCGGTCTGTTAAGTCAGCGGTCAAAGCCCGGGGCTCAACCCCGGCCCGCCGTTGAAACTGGCAGTCTCGAGTTGGAGAGAAGTATGCGGAATGCGCGGTGTAGCGGTGAAATGCATAGATATCGCGCAGAACTCCGATTGCGAAGGCAGCATGCCGGCTCCACACTGACGCTGAGGCACGAAAGCGTGGGTATCGAACAGG
ASV002	Bacteria	Bacteroidota	Bacteroidia	Bacteroidales	Muribaculaceae	Muribaculaceae_Genus	TACGGAGGATGCGAGCGTTATCCGGATTTATTGGGTTTAAAGGGTGCGTAGGCGGTCCGTTAAGTCAGCGGTAAAATTGCGGGGCTCAACCCCGTCGAGCCGTTGAAACTGGCAGACTTGAGTTGGCGAGAAGTACGCGGAATGCGCGGTGTAGCGGTGAAATGCATAGATATCGCGCAGAACTCCGATTGCGAAGGCAGCGTACCGGCGCCAGACTGACGCTGAGGCACGAAAGCGTGGGGATCGAACAGG
ASV179	Bacteria	Bacteroidota	Bacteroidia	Bacteroidales	Muribaculaceae	Muribaculaceae_Genus	TACGGAGGATGCGAGCGTTATCCGGATTTATTGGGTTTAAAGGGTGCGTAGGCGGTCCGTTAAGTCAGCGGTAAAATTGCGGGGCTCAACCCCGTCGAGCCGTTGAAACTGGCAGACTTGAGTTGGCGAGAAGTACGCGGAATGCGCGGTGTAGCGGTGAAATGCATAGATATCGCGCAGAACTCCGATTGCGAAGGCAGCGTACCGGCGCCACACTGACGCTGAGGCACGAAAGCGTGGGGATCGAACAGG
ASV022	Bacteria	Bacteroidota	Bacteroidia	Bacteroidales	Muribaculaceae	Muribaculaceae_Genus	TACGGAGGATGCGAGCGTTATCCGGATTTATTGGGTTTAAAGGGTGCGTAGGCGGTCCGTTAAGTCAGCGGTAAAATTGCGGGGCTCAACCCCGTCGAGCCGTTGAAACTGGCAGACTTGAGTTGGCGAGAAGTACGCGGAATGCGCGGTGTAGCGGTGAAATGCATAGATATCGCGCAGAACTCCGATTGCGAAGGCAGCGTACCGGTGCCACACTGACGCTGAGGCACGAAAGCGTGGGGATCGAACAGG
ASV014	Bacteria	Bacteroidota	Bacteroidia	Bacteroidales	Muribaculaceae	Muribaculaceae_Genus	TACGGAGGATGCGAGCGTTATCCGGATTTATTGGGTTTAAAGGGTGCGCAGGCGGCGAGTTAAGTCAGCGGTAAAATTTCGGGGCTCAACCCCGACAAGCCGTTGAAACTGGCTTGCTAGAGTGGGCGAGAAGTATGCGGAATGCGTGGTGTAGCGGTGAAATGCATAGATATCACGCAGAACTCCGATTGCGAAGGCAGCATACCGGCGCCCAACTGACGCTCAGGCACGAAAGCGTGGGTATCGAACAGG
ASV004	Bacteria	Bacteroidota	Bacteroidia	Bacteroidales	Muribaculaceae	Muribaculaceae_Genus	TACGGAGGATGCGAGCGTTATCCGGATTTATTGGGTTTAAAGGGTGCGCAGGCGGAAGATCAAGTCAGCGGTAAAATTGAGAGGCTCAACCTCTTCGAGCCGTTGAAACTGGTTTTCTTGAGTGAGCGAGAAGTATGCGGAATGCGTGGTGTAGCGGTGAAATGCATAGATATCACGCAGAACTCCGATTGCGAAGGCAGCATACCGGCGCTCAACTGACGCTCATGCACGAAAGTGTGGGTATCGAACAGG
ASV005	Bacteria	Bacteroidota	Bacteroidia	Bacteroidales	Muribaculaceae	Muribaculaceae_Genus	TACGGAGGATGCGAGCGTTATCCGGATTTATTGGGTTTAAAGGGTGCGCAGGCGGAGGTGCAAGTCAGCGGTCAAATTGCGGGGCTCAACCCCGTACTGCCGTTGAAACTGCATCCCTTGAGTGCGCGAGAAGTATGCGGAATGCGTGGTGTAGCGGTGAAATGCATAGATATCACGCAGAACTCCGATTGCGAAGGCAGCATACCGGCGCGCAACTGACGCTCATGCACGAAAGCGTGGGTATCGAACAGG
ASV	Bacteria	Bacteroidota	Bacteroidia	Bacteroidales	Muribaculaceae	Muribaculaceae_Genus	TACGGAGGATGCGAGCGTTATCCGGATTTATTGGGTTTAAAGGGTGCGTAGGCGGGGAGCCAAGTCAGCGGTAAAATCGTGGGGCTCAACCCCATCCCGCCGTTGAAACTGGTTTTCTTGAGTGGATGAGAAGTATGCGGAATGCGTGGTGTAGCGGTGAAATGCATAGATATCACGCAGAACTCCGATTGCGAAGGCAGCATACCGGCATCCAACTGACGCTGAAGCACGAAGGCGTGGGTATCAAACAGG

006							
ASV046	Bacteria	Bacteroidota	Bacteroidia	Bacteroidales	Muribaculaceae	Muribaculaceae_Genus	TACGGAGGATGCGAGCGTTATCCGGATTTATTGGGTTTAAAGGGTGCGTAGGCGGAGCGTTAAGTCAGTGGTCAAATTGAGGGGCTCAACCCCTTCCCGCCATTGAAACTGGCGCTCTTGAGTGGAAGAGAAGTATGCGGAATGCGTGGTGTAGCGGTGAAATGCATAGATATCACGCAGAACCCCGATTGCGAAGGCAGCATGCCGGCTTCCTACTGACGCTGAAGCACGAAAGCGTGGGGATCGAACAGG
ASV018	Bacteria	Bacteroidota	Bacteroidia	Bacteroidales	Muribaculaceae	Muribaculaceae_Genus	TACGGAGGATGCGAGCGTTATCCGGATTTATTGGGTTTAAAGGGTGCGTAGGCGGATCGTTAAGTCAGTGGTCAAATTGAGGGGCTCAACCCCTTCCCGCCATTGAAACTGGCGATCTTGAGTGGAAGAGAAGTATGCGGAATGCGTGGTGTAGCGGTGAAATGCATAGATATCACGCAGAACCCCGATTGCGAAGGCAGCATGCCGGCTTCCTACTGACGCTGAAGCACGAAAGCGTGGGGATCGAACAGG
ASV038	Bacteria	Bacteroidota	Bacteroidia	Bacteroidales	Muribaculaceae	Muribaculaceae_Genus	TACGGAGGATGCGAGCGTTATCCGGATTTATTGGGTTTAAAGGGTGCGTAGGCGGATTTTTAAGTCAGCGGTAAAATGTCCGGGCTCAACCCGGGCCGGCCGTTGAAACTGGGGATCTTGAGTGGGCGAGAAGTATGCGGAATGCGTGGTGTAGCGGTGAAATGCATAGATATCACGCAGAACTCCGATTGCGAAGGCAGCATACCGGCGCCCGACTGACGCTGAGGCACGAAAGCGTGGGGATCGAACAGG
ASV007	Bacteria	Bacteroidota	Bacteroidia	Bacteroidales	Muribaculaceae	Muribaculaceae_Genus	TACGGAGGATGCGAGCGTTATCCGGATTTATTGGGTTTAAAGGGTGCGTAGGCGGGCTTTTAAGTCAGCGGTAAAAATTCGGGGCTCAACCCCGTCCGGCCGTTGAAACTGGGGGCCTTGAGTGGGCGAGAAGAAGGCGGAATGCGTGGTGTAGCGGTGAAATGCATAGATATCACGCAGAACCCCGATTGCGAAGGCAGCCTTCCGGCGCCCTACTGACGCTGAGGCACGAAAGTGCGGGGATCGAACAGG
ASV502	Bacteria	Bacteroidota	Bacteroidia	Bacteroidales	Bacteroidales_Family	Bacteroidales_Family_Genus	TACGTAGGTGGCAAGCGTTGTCCGGATTTATTGGGCGTAAAGTGAGCGCAGGCGGTTTCTTAAGTCTGATGTGAAAGCCCCCGGCTCAACCGGGGAGGGTCATTGGAAACTGGTTTTCTTGAGTGGATGAGAAGTATGCGGAATGCGTGGTGTAGCGGTGAAATGCATAGATATCACGCAGAACTCCGATTGCGAAGGCAGCATACCGGCATCCAACTGACGCTGAAGCACGAAGGCGTGGGTATCAAACAGG
ASV399	Bacteria	Firmicutes	Clostridia	Clostridia_Order	Clostridia_Order_Family	Clostridia_Order_Family_Genus	TACGTAGGGAGCAAGCGTTATCCGGATTTATTGGGTGTAAAGGGTGCGTAGACGGAAGGCAAGTTAGTTGTGAAATCCCTCGGCTTAACTGAGGAACTGCAACTAAAACTATCTTTCTTGAGTGCTGGAGAGGAAAGTGGAATTCCTAGTGTAGCGGTGAAATGCGTAGATATCACGCAGAACTCCGATTGCGAAGGCAGCATACCGGCGCTCAATTGACGCTCATGCACGAAAGTGTGGGTATCAAACAGG
ASV490	Bacteria	Firmicutes	Clostridia	Clostridia_UCG-014	Clostridia_UCG-014_Family	Clostridia_UCG-014_Family_Genus	TACGTAGGGAGCGAGCGTTATCCGGAATTATTGGGTGTAAAGGGTGCGTAGACGGGAAAGCAAGTTAGTTGTGAAATCCCTCGGCTTAACTGAGGAACTGCAACTAAAACTACTTTTCTTGAGTGCAGGAGAGGTAAGTGGAATTCCTAGTGTAGCGGTGAAATGCGTAGATATCACGCAGAACTCCGATTGCGAAGGCAGCATACCGGCGCTCAACTGACGCTCATGCACGAAAGTGTGGGTATCGAACAGG
ASV605	Bacteria	Bacteria_Phylum	Bacteria_Phylum_Class	Bacteria_Phylum_Class_Order	Bacteria_Phylum_Class_Order_Family	Bacteria_Phylum_Class_Order_Family_Genus	TACGTAGGGAGCAAGCGTTGTTCGGAATGACTGGGCGTAAAGGGTGCGTAGGCGGTTGTACAAGTTAGAAGTGAAATACCCAGGGCTTAACTCGGGTGCTGCTTCTAAAACTGTATGACTTGAGTGCAGAAGAGGGAAGTGGAATTCCATGTGTAGCGGTGAAATGCGTAGATATCGCGCAGAACTCCGATTGCGAAGGCAGCGTACCGGTGCCACACTGACGCTCATGCACGAAAGCGTGGGTATCGAACAGG

ASV550	Bacteria	Bacteroidota	Bacteroidia	Bacteroidales	Porphyromonadaceae	Porphyromonadaceae_Genus	TACGTAGGAGGCAAGCGTTATCCGGAATGACTGGGCGTAAAGGGTGCGTAGGTGGTTTGACAAGTTAGTAGCGCAACTCCGGAGCTTAACTTCGGAACTACTACTAAAACTGTTGGACTTGAGTGCAGGAGGGGCAATCGGAATTCCATGTGTAGCGGTGGAATGCATAGATATCACGCAGAACTCCGATTGCGAAGGCAGCATACCGGCGCGCAACTGACGCTCATGCACGAAAGCGTGGGTATCGAACAGG
ASV557	Bacteria	Firmicutes	Clostridia	Oscillospirales	Ruminococcaceae	Ruminococcaceae_Genus	TACGTAGGTGGCGAGCGTTGTCCGGAATTACTGGGTGTAAAGGGAGCGTAGGCGGGAGAGCAAGTCGACTGTGAAATCTATGGGCTTAACCCATAGCTGCGATCGAAACTGTTCATCTTGAGTGAAGTAGAGGCAGGCGGAATTCCTAGTGTAGCGGTGAAATGCATAGATATCGCGCAGAACTCCGATTGCGAAGGCAGCATGCCGGCTCCACACTGACGCTGAGGCACGAAAGCGTGGGTATCGAACAGG
ASV559	Bacteria	Firmicutes	Clostridia	Oscillospirales	Ruminococcaceae	Anaerotruncus	TACGTAGGGAGCGAGCGTTGTCCGGAATTACTGGGTGTAAAGGGAGCGTAGGCGGGCGAGAAAGTTGAATGTTAAATCTACCGGCTTAACTGGTAGCAGCGTTCAAAACTTCTTGTCTTGAGTGAAGTAGAGGCAGGCGGAATTCCTAGTGTAGCGGTGAAATGCATAGATATCGCGCAGAACTCCGATTGCGAAGGCAGCGTACCGGCGCCAGACTGACGCTGAGGCACGAAAGCGTGGGGATCGAACAGG
ASV504	Bacteria	Bacteroidota	Bacteroidia	Bacteroidales	Bacteroidales_Family	Bacteroidales_Family_Genus	TACGTAGGTGGCAAGCGTTGTCCGGAATTACTGGGTGTAAAGGGAGCGCAGGCGGGAAGCCAAGTCAGCTGTGAAAACTACGGGCTTAACTTGTAGACTGCAGTTGAAACTGGTTTTCTTGAGTGAAGTGGAGGTTGGCGGAATTCCGAGTGTAGCGGTGAAATGCATAGATATCACGCAGAACTCCGATTGCGAAGGCAGCATACCGGCGCGCAACTGACGCTCATGCACGAAAGCGTGGGTATCGAACAGG
ASV516	Bacteria	Bacteroidota	Bacteroidia	Bacteroidales	Bacteroidales_Family	Bacteroidales_Family_Genus	TACGGAGGATGCGAGCGTTGTCCGGAATTACTGGGTGTAAAGGGAGCGCAGGCGGGAAGCCAAGTCAGCTGTGAAAACTACGGGCTTAACTTGTAGACTGCAGTTGAAACTGGTTTTCTTGAGTGAAGTAGAGGTTGGCGGAATTCGTGGTGTAGCGGTGAAATGCTTAGATATCACGAAGAACTCCGATTGCGAAGGCAGCCTGCTGGACTGTAACTGACGCTGATGCTCGAAAGTGTGGGTATCAAACAGG
ASV035	Bacteria	Firmicutes	Clostridia	Oscillospirales	Oscillospirales_Family	Oscillospirales_Family_Genus	TACGTAGGTGGCAAGCGTTGTCCGGAATTACTGGGTGTAAAGGGAGCGCAGGCGGGAAGCCAAGTCAGCTGTGAAAACTACGGGCTTAACTTGTAGACTGCAGTTGAAACTGGTTTTCTTGAGTGAAGTAGAGGTTGGCGGAATTCCGAGTGTAGCGGTGAAATGCGTAGATATTCGGAGGAACACCGGTGGCGAAGGCGGCCAACTGGGCTTTAACTGACGCTGAGGCTCGAAAGTGTGGGGAGCAAACAGG
ASV507	Bacteria	Firmicutes	Clostridia	Oscillospirales	Butyricicoccaceae	Butyricicoccaceae_Genus	TACGTAGGGAGCAAGCGTTATCCGGATTTACTGGGTGTAAAGGGCGTGTAGGCGGGCCTGCAAGTCAGAAGTGAAATCTGGGGGCTTAACCCCCAAACTGCTTTTGGAACTGTGGGTCTTGAGTGATGGAGAGGCAGGCGGAATTCCCAGTGTAGCGGTGAAATGCATAGATATCGCGCAGAACTCCGATTGCGAAGGCAGCGTACCGGCGCCAGACTGACGCTGAGGCACGAAAGCGTGGGGATCGAACAGG
ASV454	Bacteria	Firmicutes	Clostridia	Oscillospirales	Ruminococcaceae	Ruminococcaceae_Genus	TACGTATGGAGCAAGCGTTGTCCGGATTTACTGGGTGTAAAGGGTGCGTAGGCGGCTTTGCAAGTCAGAAGTGAAATCCATGGGCTTAACCCATGAACTGCTTTTGAAACTGCAGAGCTTGAGTGGAGTAGAGGTAGGCGGAATTCCCGGTGTAGCGGTGAAATGCGTAGATATCACGCAGAACCCCGATTGCGAAGGCAGCCTTCCGGCGCCCTACTGACGCTGAGGCACGAAAGTGCGGGGATCGAACAGG
ASV	Bacteria	Bacteria_Phylum	Bacteria_Phylum_Class	Bacteria_Phylum_Class_Order	Bacteria_Phylum_Class_Order_Family	Bacteria_Phylum_Class_Order_Family_Genus	TACGTATGGGGCAAGCGTTATCCGGATTTACTGGGTGTAAAGGGTGCGTAGGCGGCTTTGCAAGTCAGAAGTGAAATCTATGGGCTTAACCCATAAACTGCTTTTGAAACTGCAGGGCTTGAGTGAAGTAGAGGTAGGCGGAATTCCCGGTGTAGCGGTGAAATGCATAGATATCACGCAGAACCCCGATTGCGAAGGCAGCCTTCCGGCGCCCTACTGACGCTGAGGCACGAAAGTGCGGGGATCGAACAGG

493							
ASV481	Bacteria	Firmicutes	Clostridia	Clostridia_Order	Clostridia_Order_Family	Clostridia_Order_Family_Genus	TACGTAGGTGGCAAGCGTTGTCCGGATTTACTGGGTGTAAAGGGCGTGCAGCCGGGTTTATAAGTCAGATGTGAAATCCCGCGGCTCAACCGCGGAACTGCATTTGAAACTGTAGATCTTGAGTACTGGAGAGGCAGGCGGAATTCCTAGTGTAGCGGTGAAATGCGTAGATATCGCGCAGGACTCCGATTGCGAAGGCAGCATGCCGGCTCCACACTGACGCTGAGGCACGAAAGCGTGGGTATCGAACAGG
ASV495	Bacteria	Firmicutes	Clostridia	Oscillospirales	Oscillospiraceae	Oscillibacter	TACGTAGGTGGCAAGCGTTGTCCGGATTTACTGGGTGTAAAGGGCGTGCAGTCGGGAAGACAAGTCAGATGTGAAATCCCGCGGCTCAACCGCGGAACTGCATTTGAAACTGTTTTTCTTGAGTACCGGAGAGGTCATCGGAATTCCTTGTGTAGCGGTGAAATGCGTAGATATCGCGCAGAACTCCGATTGCGAAGGCAGCATGCCGGCTCCACACTGACGCTGAGGCACGAAAGCGTGGGTATCGAACAGG
ASV437	Bacteria	Firmicutes	Clostridia	Oscillospirales	Oscillospiraceae	Pseudoflavonifractor	TACGTAGGTGGCAAGCGTTATCCGGATTTACTGGGTGTAAAGGGCGTGTAGGCGGGAATGCAAGTCAGATGTGAAAACTCAGGGCTCAACCCTGAGCCTGCATTTGAAACTGTATTTCTTGAGTGCTGGAGAGGCAATCGGAATTCCGTGTGTAGCGGTGAAATGCGTAGATATCACGCAGAACTCCGATTGCGAAGGCAGCATACCGGCGCTCAACTGACGCTCATGCACGAAAGTGTGGGTATCGAACAGG
ASV422	Bacteria	Firmicutes	Clostridia	Lachnospirales	Lachnospiraceae	Lachnospiraceae_Genus	TACGTAGGGGGCAAGCGTTATCCGGATTCACTGGGCGTAAAGGGAGCGCAGGCGGCACGGCAAGTCTGGTGTGAAAACCCGGGGCTCAACCCCGGGACTGCATTGGAAACTGCCGGGCTGGAGTGTCGGAGGGGTAAGCGGAATTCCTAGTGTAGCGGTGAAATGCATAGATATCACGCAGAACCCCGATTGCGAAGGCAGCATGCCGGCTTCCTACTGACGCTGAAGCACGAAGGCGTGGGGATCGAACAGG
ASV458	Bacteria	Bacteria_Phylum	Bacteria_Phylum_Class	Bacteria_Phylum_Class_Order	Bacteria_Phylum_Class_Order_Family	Bacteria_Phylum_Class_Order_Family_Genus	TACGTAGGGGGCGAGCGTTATCCGGATTCACTGGGCGTAAAGGGAGCGCAGGCGGCACGGCAAGTCCGGTGTGAAAGCCCGGGGCCCAACCCCGGGACTGCACTGGAAACTGCCGGGCTGGAGTGTCGGAGGGGTAAGCGGAATTCCTGGTGTAGCGGTGAAATGCGTAGATATCGCGCAGAACTCCGATTGCGAAGGCAGCGTACCGGCGCCAGACTGACGCTGAGGCACGAAAGCGTGGGGATCGAACAGG
ASV424	Bacteria	Firmicutes	Clostridia	Lachnospirales	Lachnospiraceae	Lachnospiraceae_Genus	TACGTATGGAGCAAGCGTTATCCGGATTTACTGGGTGTAAAGGGAGCGCAGGCGGTCTGGCAAGTCTGGTGTGAAAGGCCGGGGCTCAACCCCGGGACTGCATTGGAAACTGGCGGGCTGGAGTGCCGGAGAGGTAAGCGGAATTCCTAGTGTAGCGGTGAAATGCATAGATATCGCGCAGAACTCCGATTGCGAAGGCAGCGTACCGGCGCCAGACTGACGCTGAGGCACGAAAGCGTGGGGATCGAACAGG
ASV463	Bacteria	Firmicutes	Clostridia	Lachnospirales	Lachnospiraceae	A2	TACGTATGGAGCAAGCGTTATCCGGATTTACTGGGTGTAAAGGGAGCGCAGACGGCAGGGCAAGTCTGATGTGAAATACCGGGGCCCAACCCCGGGGCTGCATTGGAACTGCCAGGCTGGAGTGCCGGAGAGGCAGGCGGAATTCCTAGTGTAGCGGTGAAATGCATAGATATCGCGCAGAACCCCGATTGCGAAGGCAGCCTTCCGGCGCCCTACTGACGCTGAGGCACGAAAGTGCGGGGATCGAACAGG
ASV519	Bacteria	Firmicutes	Clostridia	Lachnospirales	Lachnospiraceae	Lachnospiraceae_Genus	TACGTATAGAGCAAGCGTTATCCGGATTTACTGGGTGTAAAGGGAGCGCAGGCGGCATGGCAAGTCAGATGTGAAAGCCCGGGGCTCAACCCCGGGACTGCATTTGAAACTGTCAGGCTGGAGTGCAGGAGAGGCAGGCGGAATTCCTAGTGTAGCGGTGAAATGCATAGATATCACGCAGAACCCCGATTGCGAAGGCAGCCTTCCGGCGCCCTACTGACGCTGAGGCACGAAAGTGCGGGGATCGAACAGG

ASV436	Bacteria	Firmicutes	Clostridia	Lachnospirales	Lachnospiraceae	A2	TACGTATGGAGCAAGCGTTATCCGGATTTACTGGGTGTAAAGGGAGCGTAGGCGGCCTGGCAAGCCTGATGTGAAATACCGGGGCCCAACCCCGGGGCTGCATTGGGAACTGCCAGGCTGGAGTGCCGGAGAGGCAGGCGGAATTCCTAGTGTAGCGGTGAAATGCGTAGATATCGCGCAGAACTCCGATTGCGAAGGCAGCGTACCGGCGCCAGACTGACGCTGAGGCACGAAAGCGTGGGGATCGAACAGG
ASV451	Bacteria	Firmicutes	Clostridia	Lachnospirales	Lachnospiraceae	A2	TACGTATGGAGCAAGCGTTATCCGGATTTACTGGGTGTAAAGGGAGCGTAGGCGGCCTGGCAAGCCTGATGTGAAATACCGGGGCCCAACCCCGGGGCTGCATTGGGAACTGCCAGGCTGGAGTGCCGGAGAGGCAGGCGGAATTCCTAGTGTAGCGGTGAAATGCGTAGATATCACGCAGAACTCCGATTGCGAAGGCAGCATACCGGCGCTCAACTGACGCTCATGCACGAAAGTGTGGGTATCGAACAGG
ASV408	Bacteria	Bacteroidota	Bacteroidia	Bacteroidales	Porphyromonadaceae	Porphyromonadaceae_Genus	TACGGAGGATGCGAGCGTTATCCGGATTTACTGGGTGTAAAGGGAGCGCAGGCGGCACGGCAAGCCTGATGTGAAAGCCCGCGGCCCAACCGCGGGACTGCATTGGGAACTACCGGGCTGGAGTGCCGGAGAGGTAGGCGGAATTCCTAGTGTAGCGGTGAAATGCATAGATATCACGCAGAACTCCGATTGCGAAGGCAGCATACCGGCGCGCAACTGACGCTCATGCACGAAAGCGTGGGTATCGAACAGG
ASV468	Bacteria	Firmicutes	Clostridia	Clostridia_Order	Clostridia_Order_Family	Clostridia_Order_Family_Genus	TACGTAGGGGGCAAGCGTTATCCGGATTTACTGGGTGTAAAGGGGGCGCAGACGGCCATGGCAAGCCCGGTGTGAAAGGCAGGGGCATAACCCCTGGACTGCACTGGGAACTGTCAGGCTGGAGTGCCGGAGGGGTAAGCGGAATTCCTAGTGTAGCGGTGAAATGCATAGATATCACGCAGAACCCCGATTGCGAAGGCAGCCTTCCGGCGCCCTACTGACGCTGAGGCACGAAAGTGCGGGGATCGAACAGG
ASV510	Bacteria	Bacteria_Phylum	Bacteria_Phylum_Class	Bacteria_Phylum_Class_Order	Bacteria_Phylum_Class_Order_Family	Bacteria_Phylum_Class_Order_Family_Genus	TACGTAGGGGGCAAGCGTTATCCGGATTTACTGGGTGTAAAGGGGGCGCAGACGGCCATGGCAAGCCCGGTGTGAAAGGCAGGGGCATAACCCCTGGACTGCACTGGGAACTGTCAGACTGGAGTGTCGGAGAGGCAAGCGGAATTCCTAGTGTAGCGGTGAAATGCGTAGATATCACGCAGAACTCCGATTGCGAAGGCAGCATGCCGGCTCCACACTGACGCTGAGGCACGAAAGCGTGGGTATCGAACAGG
ASV403	Bacteria	Firmicutes	Clostridia	Lachnospirales	Lachnospiraceae	Lachnospiraceae_Genus	TACGTATGGGGCAAGCGTTATCCGGATTTACTGGGTGTAAAGGGAGCGCAGACGGCAGGGCAAGTCTGATGTGAAAGTCCGGGGCTCAACCCCGGGACTGCATTGGAAACTGTCCGGCTAGAGTGCAGGAGAGGTAAGTGGAATTCCTAGTGTAGCGGTGAAATGCATAGATATCGCGCAGAACTCCGATTGCGAAGGCAGCGTACCGGCGCCAGACTGACGCTGAGGCACGAAAGCGTGGGGATCGAACAGG
ASV501	Bacteria	Firmicutes	Clostridia	Lachnospirales	Lachnospiraceae	Lachnospiraceae_Genus	TACGTATGGAGCAAGCGTTATCCGGATTTACTGGGTGTAAAGGGAGCGCAGACGGCAGGGCAAGTCTGATGTGAAAGTCCGGGGCTCAACCCCGGGACTGCATTGGAAACTGTCCGGCTAGAGTGCAGGAGAGGTAAGTGGAATTCCTAGTGTAGCGGTGAAATGCGTAGATATTACGCAGAACTCCGATTGCGAAGGCAGCATACCGGCGCTCAACTGACGCTCATGCACGAAAGCGTGGGTATCGAACAGG
ASV467	Bacteria	Firmicutes	Clostridia	Lachnospirales	Lachnospiraceae	28-4	TACGTAGGGGGCAAGCGTTATCCGGATTTACTGGGTGTAAAGGGAGCGTAGACGGCAGGGCAAGCCAGAAGTGGAAACGCAGAGCTTAACTTTGCGACTGCTTTTGGAACTGCCCAGCTAGAGTACAGGAGAGGTAAGTGGAATTCCTAGTGTAGCGGTGAAATGCATAGATATCGCGCAGAACTCCGATTGCGAAGGCAGCGTACCGGTGCCACACTGACGCTGAGGCACGAAAGCGTGGGGATCGAACAGG
ASV	Bacteria	Firmicutes	Clostridia	Lachnospirales	Lachnospiraceae	ASF356	TACGTAGGGGGCAAGCGTTATCCGGAATTACTGGGTGTAAAGGGAGAGTAGGCGGCAAGGTAAGCGATATGTGAAAGCCTTAAGCTTAACTTAAGGATGGCATAACGAACTATCTAGCTAGAGTACAGGAGAGGAAAGCGGAATTCCTAGTGTAGCGGTGAAATGCATAGATATCGCGCAGAACTCCGATTGCGAAGGCAGCGTACCGGCGCCAGACTGACGCTGAGGCACAAAAGCGTGGGGATCGAACAGG

540							
ASV539	Bacteria	Bacteroidota	Bacteroidia	Bacteroidales	Bacteroidales_Family	Bacteroidales_Family_Genus	TACGGAGGATGCGAGCGTTATCCGGATTTATTGGGTTTAAAGGGAGCGTAGACGGCGATGCAAGTCTGAAGTGAAAGGCGGGGGCCCAACCCCCGGACTGCTTTGGAAACTGTATGGCTGGAGTGCAGGAGAGGTAAGTGGAATGCGTGGTGTAGCGGTGAAATGCATAGATATCACGCAGAACTCCGATTGCGAAGGCAGCATGCCGGCTCCACACTGACGCTGAGGCACGAAAGCGTGGGTATCGAACAGG
ASV404	Bacteria	Firmicutes	Clostridia	Lachnospirales	Lachnospiraceae	Lachnospiraceae_Genus	TACGTAGGGGGCAAGCGTTATCCGGATTTACTGGGTGTAAAGGGAGCGTAGACGGTGATGCAAGTCAGAAGTGAAAGCCCGGGGCTCAACTCCGGGACTGCTTTTGAAACTGTGTAACTGGAGTGCAGGAGAGGTAAGCGGAATTCCTGGTGTAGCGGTGAAATGCATAGATATCACGCAGAACCCCGATTGCGAAGGCAGCATGCCGGCTTCCTACTGACGCTGAAGCACGAAAGCGTGGGGATCGAACAGA
ASV455	Bacteria	Firmicutes	Clostridia	Lachnospirales	Lachnospiraceae	Lachnospiraceae_Genus	TACGTAGGGGGCAAGCGTTTCCGGATTTACTGGGTGTAAAGGGAGCGTAGACGGTGATGCAAGTCAGAAGTGAAAGCCCGGGGCTCAACTCCGGGACTGCTTTTGAAACTGTGTGACTGGAGTGCAGGAGAGGTAAGCGGAATGCGCGGTGTAGCGGTGAAATGCATAGATATCGCGCAGAACTCCGATTGCGAAGGCAGCGTACCGGCGCCAGACTGACGCTCATGCACGAAAGCGTGGGTATCGAACAGG
ASV496	Bacteria	Firmicutes	Clostridia	Lachnospirales	Lachnospiraceae	Lachnospiraceae_Genus	TACGGAGGATGCGAGCGTTATCCGGATTTACTGGGTGTAAAGGGAGCGTAGACGGTGATGCAAGTCAGAAGTGAAAGCCCGGGGCTCAACTCCGGGACTGCTTTTGAAACTGTGTGACTGGAGTGCAGGAGAGGTAAGCGGAATTCCATGTGTAGCGGTGAAATGCATAGATATCGCGCAGAACTCCGATTGCGAAGGCAGCGTACCGGCGCTAGACTGACGCTGAGGCACGAAAGCGTGGGGATCGAACGGG
ASV396	Bacteria	Firmicutes	Clostridia	Lachnospirales	Lachnospiraceae	Lachnospiraceae_Genus	TACGTAGGGGGCAAGCGTTATCCGGATTTACTGGGTGTAAAGGGAGCGTAGGCGGCGATGCAAGTCAGAAGTGAAAGCCCGGGGCTCAACTCCGGGACTGCTTTTGAAACTGTGTGACTGGAGTGCAGGAGAGGTAAGCGGAATTCCTGGTGTAGCGGTGAAATGCGTAGATATCGCGCAGAACTCCGATTGCGAAGGCAGCGTACCGGCGCCAGACTGACGCTGAGGCACGAAAGCGTGGGGATCGAACAGG
ASV416	Bacteria	Firmicutes	Clostridia	Lachnospirales	Lachnospiraceae	Lachnospiraceae_Genus	TACGTAGGGGGCAAGCGTTATCCGGATTTACTGGGTGTAAAGGGAGCGTAGACGGATGTGCAAGTCTGATGTGAAAGGCGGGGGCTCAACCCCTGGACTGCATTGGAAACTGTAAATCTTGAGTGCCGGAGAGGTAAGCGGAATTCCTAGTGTAGCGGTGAAATGCGTAGATATCGCGCAGAACTCCGATTGCGAAGGCAGCGTACCGGCGCCAGACTGACGCTGAGGCACGAAAGCGTGGGGATCGAACAGG
ASV387	Bacteria	Firmicutes	Clostridia	Lachnospirales	Lachnospiraceae	Lachnospiraceae_Genus	TACGTAGGGGGCAAGCGTTATCCGGATTTACTGGGTGTAAAGGGAGCGCAGACGGCTGTGCAAGTCTGGAGTGAAAGGCGGGGGCCCAACCCCCGGACTGCTCTGGAAACTGTATGGCTGGAGTGCAGGAGAGGTAAGCGGAATTCCTAGTGTAGCGGTGAAATGCATAGATATCGCGCAGAACTCCGATTGCGAAGGCAGCGTACCGGCGCCAGACTGACGCTGAGGCACGAAAGCGTGGGGATCGAACAGG
ASV528	Bacteria	Firmicutes	Clostridia	Lachnospirales	Lachnospiraceae	UC5-1-2E3	TACGTAGGTGGCAAGCGTTATCCGGATTTACTGGGTGTAAAGGGAGCGCAGACGGCTGTGCAAGTCTGGAGTGAAAGCCCGGGGCTCAACCCCGGGACTGCTTTGGAAACTGTAGGGCTGGAGTGCAGGAGGGGTAAGCGGAATTCCTAGTGTAGCGGTGAAATGCGTAGATATCGCGCAGAACTCCGATTGCGAAGGCAGCGTACCGGTGCCACACTGACGCTGAGGCACGAAAGCGTGGGGATCGAACAGG

ASV393	Bacteria	Firmicutes	Clostridia	Lachnospirales	Lachnospiraceae	Lachnospiraceae_Genus	TACGTATGGAGCAAGCGTTATCCGGATTTACTGGGTGTAAAGGGAGCGTAGACGGCTGTGCAAGTCTGAAGTGAAATGCCGGGGCTCAACCCCGGAACTGCTTTGGAAACTGTACAGCTGGAGTGCAGGAGGGGTGAGCGGAATTCCTAGTGTAGCGGTGAAATGCATAGATATCGCGCAGAACTCCGATTGCGAAGGCAGCGTACCGGCGCCAGACTGACGCTGAGGCACGAAAGCGTGGGGATCGAACAGG
ASV457	Bacteria	Firmicutes	Clostridia	Lachnospirales	Lachnospiraceae	Lachnospiraceae_Genus	TACGTAGGGGGCAAGCGTTATCCGGATTTACTGGGTGTAAAGGGAGCGTAGACGGTGAGGCAAGTCTGAAGTGAAAGCCCGGGGCTTAACCGCGGGACTGCTTTGGAAACTGTCTGACTGGAGTGCTGGAGAGGTAAGTGGAATTCCCAGTGTAGCGGTGAAATGCGTAGATATCACGCAGAACTCCGATTGCGAAGGCAGCATACCGGCGCCCAACTGACGCTCAGGCACGAAAGCGTGGGTATCGAACAGG
ASV484	Bacteria	Firmicutes	Clostridia	Lachnospirales	Lachnospiraceae	Lachnospiraceae_Genus	TACGTAGGGGGCAAGCGTTATCCGGATTTACTGGGTGTAAAGGGAGCGTAGACGGCATTGCAAGTCTGAAGTGAAAGCCCGTGGCTTAACCGCGGAACGGCTTTGGAAACTGTGGAACTGGAGTGCTGGAGAGGCAAGCGGAATTCCTAGTGTAGCGGTGAAATGCGTAGATATCACGCAGAACTCCGATTGCGAAGGCAGCGTACCGGCGCCAGACTGACGCTGAGGCACGAAAGCGTGGGGATCGAACAGG
ASV547	Bacteria	Firmicutes	Clostridia	Lachnospirales	Lachnospiraceae	Acetatifactor	TACGTAGGGGGCAAGCGTTATCCGAATTTACTGGGTGTAAAGGGAGCGTAGACGGCAGCGCAAGTCCGAAGTGAAAGCCCGGGGCCCAACCCCGGGACTGCTTTGGAAACTGTGAAGCTGGAGTGCGGGAGGGGCAGGCGGAATTCCTGGTGTAGCGGTGAAATGCATAGATATCACGCAGAACTCCGATTGCGAAGGCAGCATACCGGCGCCCGACTGACGCTGAGGCACGAAAGCGTGGGGATCGAACAGG
ASV596	Bacteria	Bacteria_Phylum	Bacteria_Phylum_Class	Bacteria_Phylum_Class_Order	Bacteria_Phylum_Class_Order_Family	Bacteria_Phylum_Class_Order_Family_Genus	TACGTAGGGGGCAAGCGTTATCCGGATTTACTGGGTGTAAAGGGAGCGTAGGCGGTAAGGCAAGTCTGATGTGAAAGGCCAGGGCTCAACCCTGGGACTGCATTGGAAACTGTTTAACTGGAGTGTCGGAGAGGCAAGTGGAATTCCTAGTGTAGCGGTGAAATGCATAGATATCACGCAGAACTCCGATTGCGAAGGCAGCATACCGGCGCGCAACTGACGCTCATGCACGAAAGCGTGGGTATCGAACAGG
ASV417	Bacteria	Bacteroidota	Bacteroidia	Bacteroidia_Order	Bacteroidia_Order_Family	Bacteroidia_Order_Family_Genus	TACGTAGGTGGCAAGCGTTATCCGGATTTACTGGGTGTAAAGGGAGCGTAGACGGTGCGCCAAGTCTGGAGTGAAATGCCGCAGCTTAACTGCGGAACTGCTTTGGAAACTGGCGAACTAGAGTGCGGGAGGGGTAAGCGGAATTCCTAGTGTAGCGGTGAAATGCATAGATATCACGCAGAACTCCGATTGCGAAGGCAGCATACCGGCGCTCAACTGACGCTCATGCACGAAAGTGTGGGTATCGAACAGG
ASV382	Bacteria	Firmicutes	Clostridia	Lachnospirales	Lachnospiraceae	Acetatifactor	TACGTAGGGGGCAAGCGTTATCCGGATTTACTGGGTGTAAAGGGAGCGTAGACGGTTGTGTAAGTCTGGAGTGAAAGCCCGGGGCCCAACCCCGGGACTGCTTTGGAAACTGTGTAACTGGAGTACAGGAGGGGCAGGCGGAATTCCTGGTGTAGCGGTGAAATGCGTAGATATCACGCAGAACTCCGATTGCGAAGGCAGCATACCGGCGCGCAACTGACGCTCATGCACGAAAGCGTGGGTATCGAACAGG
ASV439	Bacteria	Bacteria_Phylum	Bacteria_Phylum_Class	Bacteria_Phylum_Class_Order	Bacteria_Phylum_Class_Order_Family	Bacteria_Phylum_Class_Order_Family_Genus	TACGTAGGTGGCAAGCGTTGTCCGGATTTACTGGGTGTAAAGGGAGCGTAGACGGTTATGTAAGTCTGGAGTGAAAGCCCGGGGCCCAACCCCGGGACTGCTTTGGAAACTGTGTAACTGGAGTACAGGAGGGGCAGGCGGAATTCCTGGTGTAGCGGTGAAATGCATAGATATCACGCAGAACTCCGATTGCGAAGGCAGCATACCGGCATCCAACTGACGCTGAAGCACGAAGGCGTGGGTATCAAACAGG
ASV	Bacteria	Bacteroidota	Bacteroidota_Class	Bacteroidota_Class_Order	Bacteroidota_Class_Order_Family	Bacteroidota_Class_Order_Family_Genus	TACGTAGGGGGCGAGCGTTATCCGGATTTACTGGGTGTAAAGGGAGCGTAGGCGGCCACGCAAGTCAGAAGTGAAAACCCGGGGCTCAACCCTGGGAGTGCTTTTGAAACTGTGCGGCTAGAGTGTCGGAGGGGTAAGTGGAATTCCTAGTGTAGCGGTGAAATGCATAGATATCACGCAGAACTCCGATTGCGAAGGCAGCATACCGGCGCTCAACTGACGCTCATGCACGAAAGTGTGGGTATCGAACAGG

456							
ASV460	Bacteria	Bacteroidota	Bacteroidia	Bacteroidales	Porphyromonadaceae	Porphyromonadaceae_Genus	TACGTAGGGGGCAAGCGTTATCCGGATTCACTGGGCGTAAAGGGAGAGTAGGTGGCAGAGCAAGTCCGAAGTGAAAACCCAAAGCTCAACTATGGGAATGCTTTAGAAACTGCTCAGCTAGAGTGCAGGAGAGGCAAGTGGAATTCTTAGTGTAGCGGTGAAATGCATAGATATCACGCAGAACTCCGATTGCGAAGGCAGCATACCGGCGCTCAACTGACGCTCATGCACGAAAGTGTGGGTATCGAACAGG
ASV525	Bacteria	Bacteroidota	Bacteroidia	Bacteroidales	Porphyromonadaceae	Porphyromonadaceae_Genus	TACGTATGGAGCAAGCGTTATCCGGACTTACTGGGTGTAAAGGGAGAGTAGGTGGCAGAGCAAGTCCGAAGTGAAAACCCAAAGCTCAACTATGGGAATGCTTTAGAAACTGCTCAGCTAGAGTGCAGGAGAGGCAAGTGGAATTCTTAGTGTAGCGGTGAAATGCATAGATATCACGCAGAACTCCGATTGCGAAGGCAGCATACCGGCGCTCAACTGACGCTCATGCACGAAAGTGTGGGTATCGAACAGG
ASV418	Bacteria	Firmicutes	Clostridia	Lachnospirales	Lachnospiraceae	Lachnospiraceae_Genus	TACGTAGGGGGCAAGCGTTATCCGGATTTACTGGGTGTAAAGGGAGCGTAGGTGGCAGGGCAAGTCAGATGTGAAAACCCGGGGCTCAACTCCGGGAGTGCATTTGAAACTGTCCAGCTAGAGTGCAGGAGAGGTAAGCGGAATTCCTAGTGTAGCGGTGAAATGCATAGATATCACGCAGAACTCCGATTGCGAAGGCAGCATACCGGCATCCAACTGACGCTGAAGCACGGAGGCGTGGGTATCAAACAGG
ASV538	Bacteria	Firmicutes	Clostridia	Lachnospirales	Lachnospiraceae	A2	TACGCATGGTGCAAGCGTTATCCGGATTTACTGGGTGTAAAGGGAGCGCAGGCGGTGCGGCAAGTCTGATGTGAAAGCCCGGGGCCCAACCCCGGGGCTGCATTGGGAACTGCCAGGCTGGAGTGCCGGAGAGGCAGGCGGAATTCCTAGTGTAGCGGTGAAATGCGTAGATATTAGGAGGAACACCAGTGGCGAAGGCAGCATACCGGCGCCCAACTGACGCTCAGGCACGAAAGCGTGGGTATCGAACAGG
ASV586	Bacteria	Firmicutes	Clostridia	Oscillospirales	Oscillospiraceae	Pseudoflavonifractor	TACGTATGGTGCAAGCGTTATCCGGATTTACTGGGTGTAAAGGGAGCGCAGGCGGCGCGGCAAGTCAGATGTGAAAGCCCGGGGCTCAACCCCGGGACTGCATTTGAAACTGTATTTCTTGAGTGCTGGAGAGGCAATCGGAATTCCGTGTGTAGCGGTGAAATGCGTAGATATACGGAGGAACACCAGTGGCGAAGGCAGCATACCGGCATCCAACTGACGCTGAAGCACGAAGGCGTGGGTACCAAACAGG
ASV298	Bacteria	Firmicutes	Clostridia	Lachnospirales	Lachnospiraceae	Coprococcus	TACGTATGGTGCAAGCGTTATCCGGATTTACTGGGTGTAAAGGGTGCGTAGGTGGCAAGGCAAGTCTGAAGTGAAAATCCGGGGCTCAACCCCGGAACTGCTTTGGAAACTGTTTAGCTGGAGTACAGGAGAGGTAAGTGGAATTCCTAGTGTAGCGGTGAAATGCGTAGATATTAGGAGGAACACCAGTGGCGAAGGCGACTTACTGGACTGCTACTGACACTGAGGCACGAAAGCGTGGGGAGCAAACAGG
ASV041	Bacteria	Firmicutes	Clostridia	Lachnospirales	Lachnospiraceae	Lachnospiraceae_NK4B4_group	TACGTATGGAGCAAGCGTTATCCGGATTTACTGGGTGTAAAGGGAGCGCAGGCGGCATGGCAAGTCAGATGTGAAAGCCCGGGGCTCAACCCCGGGACTGCATTTGAAACTGTCAGGCTGGAGTGCAGGAGAGGCAGGCGGAATTCCTAGTGTAGCGGTGAAATGCGTAGATATTAGGAGGAACACCAGTGGCGAAGGCGGCCTGCTGGACTGTAACTGACGCTGAGGCTCGAAAGCGTGGGGAGCAAACAGG
ASV070	Bacteria	Firmicutes	Clostridia	Lachnospirales	Lachnospiraceae	A2	TACGTATGGGGCAAGCGTTATCCGGATTTACTGGGTGTAAAGGGAGCGTAGGCGGCCTGGCAAGCCTGATGTGAAATACCGGGGCTCAACCCCGGGGCTGCATTGGGAACTGCCAGGCTGGAGTGCCGGAGAGGCAGGCGGAATTCCTAGTGTAGCGGTGAAATGCGTAGATATTAGGAGGAACACCAGTGGCGAAGGCGGCCTGCTGGACGGTGACTGACGCTGAGGCTCGAAAGCGTGGGGAGCAAACAGG

ASV530	Bacteria	Firmicutes	Clostridia	Lachnospirales	Lachnospiraceae	A2	TACGTATGGGGCAAGCGTTATCCGGATTTACTGGGTGTAAAGGGAGCGTAGGCGGCCTGGCAAGCCTGATGTGAAATACCGGGGCCCAACCCCCGGACTGCTCTGGAAACTGTATGGCTGGAGTGCAGGAGAGGTAAGCGGAATTCCTAGTGTAGCGGTGAAATGCGTAGATATTAGGAGGAACACCAGTGGCGAAGGCGGCCTGCTGGACGGTGACTGACGCTGAGGCTCGAAAGCATGGGTAGCGAACAGG
ASV574	Bacteria	Firmicutes	Clostridia	Lachnospirales	Lachnospiraceae	A2	TACGTATGGGGCAAGCGTTATCCGGATTTACTGGGTGTAAAGGGAGCGTAGGCGGCCTGGCAAGCCTGATGTGAAATACCGGGGCCCAACCCCGTCTCGTTCTATAAACTGATAGACTAGAGTGTGGTAGAGGCAAGTGGAATTTCTAGTGTAGCGGTAGAATGCGTAGATATTAGAAGGAACACCAGTGGCGAAGGCGGCCTGCTGGACGGTGACTGACGCTGAGGCTCGAAAGCGTGGGGAGCAAACAGG
ASV216	Bacteria	Firmicutes	Clostridia	Lachnospirales	Lachnospiraceae	A2	TACGTATGGGGCAAGCGTTATCCGGATTTACTGGGTGTAAAGGGAGCGCAGGCGGCCTGGCAAGCCTGATGTGAAATACCGGGGCCCAACCCCGGGGCTGCATTGGGAACTGCCAGGCTGGAGTGCCGGAGAGGCAGGCGGAATTCCTAGTGTAGCGGTGAAATGCGTAGATATTAGGAGGAACACCAGTGGCGAAGGCGGCCTGCTGGACGGTGACTGACGCTGAGGCTCGAAAGCGTGGGGAGCAAACAGG
ASV009	Bacteria	Firmicutes	Clostridia	Lachnospirales	Lachnospiraceae	A2	TACGTATGGGGCAAGCGTTATCCGGATTTACTGGGTGTAAAGGGAGCGTAGGCGGCCTGGCAAGCCTGATGTGAAATACCGGGGCCCAACCCCGGGGCTGCATTGGGAACTGCCAGGCTGGAGTGCCGGAGAGGCAGGCGGAATTCCTAGTGTAGCGGTGAAATGCGTAGATATTAGGAGGAACACCAGTGGCGAAGGCGGCCTGCTGGACGGTGACTGACGCTGAGGCTCGAAAGCGTGGGGAGCAAACAGG
ASV082	Bacteria	Firmicutes	Clostridia	Lachnospirales	Lachnospiraceae	A2	TACGTATGGGGCAAGCGTTATCCGGATTTACTGGGTGTAAAGGGAGCGTAGGCGGCAGGGCAAGCCTGATGTGAAAGCCCGGGGCTCAACCCCGGGACTGCATTGGGAACTGCCCCGCTCGAGTGCCGGAGGGGCAGGCGGAATTCCTAGTGTAGCGGTGAAATGCGTAGATATTAGGAGGAACACCAGTGGCGAAGGCGGCCTGCTGGACGGTGACTGACGCTGAGGCTCGAAAGCGTGGGGAGCAAACAGG
ASV033	Bacteria	Firmicutes	Clostridia	Lachnospirales	Lachnospiraceae	Roseburia	TACGTATGGTGCAAGCGTTATCCGGATTTACTGGGTGTAAAGGGAGCGCAGGCGGCACGGCAAGCCTGATGTGAAAGCCCGCGGCCCAACCGCGGGACTGCATTGGGAACTACCGGGCTGGAGTGCCGGAGAGGTAGGCGGAATTCCTAGTGTAGCGGTGAAATGCGTAGATATTAGGAGGAACACCAGTGGCGAAGGCGGCCTACTGGACGGTGACTGACGCTGAGGCTCGAAAGCGTGGGGAGCAAACAGG
ASV060	Bacteria	Firmicutes	Clostridia	Lachnospirales	Lachnospiraceae	Lachnospiraceae_Genus	TACGTATGGTGCAAGCGTTATCCGGATTTACTGGGTGTAAAGGGAGCGCAGGCGGCGCGTTAAGCCTGGTGTGAAAGCCCGCGGCCCAACCGCGGGACTGCATTGGGAACTGGCGCGCTGGAGTGCCGGAGGGGTAGGCGGAATTCCTAGTGTAGCGGTGAAATGCGTAGATATTAGGAGGAACACCAGTGGCGAAGGCGGCCTACTGGACGGTGACTGACGCTGAGGCTCGAAAGCGTGGGGAGCAAACAGG
ASV317	Bacteria	Firmicutes	Clostridia	Lachnospirales	Lachnospiraceae	Lachnospiraceae_Genus	TACGTATGGTGCAAGCGTTATCCGGATTTACTGGGTGTAAAGGGAGCGCAGGCGGCACGCTAAGCCTGATGTGAAAACCCGCGGCCCAACCGCGGGAGTGCATTGGGAACTGGCGGGCTGGAGTACCGGAGGGGCAGGCGGAATTCCTAGTGTAGCGGTGAAATGCGTAGATATTAGGAGGAACACCAGTGGCGAAGGCGGCCTGCTGGACGGAGACTGACGCTGAGGCTCGAAAGCGTGGGGAGCAAACAGG
ASV	Bacteria	Firmicutes	Clostridia	Lachnospirales	Lachnospiraceae	Lachnospiraceae_Genus	TACGTATGGAGCAAGCGTTATCCGGATTTACTGGGTGTAAAGGGAGCGTAGGCGGCTCTGTAAGTCTGATGTGAAAATTTGGGGCTCAACCCCAAACGTGCATTGGAAACTGCAGGGCTTGAGTGCCGGAGAGGCAGGCGGAATTCCTAGTGTAGCGGTGAAATGCGTAGATATTAGGAGGAACACCAGTGGCGAAGGCGGCCTGCTGGACGGTAACTGACGCTGAGGCTCGAAAGCGTGGGGAGCAAACAGG

225							
ASV419	Bacteria	Firmicutes	Clostridia	Lachnospirales	Lachnospiraceae	Lachnospiraceae_Genus	TACGTATGGAGCAAGCGTTATCCGGATTTACTGGGTGTAAAGGGAGCGTAGGCGGCTCTGTAAGTCTGATGTGAAAATTTGGGGCTCAACTCCAAACGTGCATTGGAAACTGCAGGGCTTGAGTGTCGGAGAGGCAGGCGGAATTCCTAGTGTAGCGGTGAAATGCGTAGATATTAGGAGGAACACCAGTGGCGAAGGCGGCCTGCTGGACGGTAACTGACGCTGAGGCTCGAAAGCGTGGGGAGCAAACAGG
ASV544	Bacteria	Firmicutes	Clostridia	Lachnospirales	Lachnospiraceae	Lachnospiraceae_Genus	TACGTATGGAGCAAGCGTTATCCGGATTTACTGGGCGTAAAGGGAGCGTAGGCGGCCTGCCAAGTCTGATGTGAAAGTCCGGGGCTCAACCCCGGGACTGCATTGGAAACTGTCCGGCTAGAGTGCAGGAGGGGCAGGCGGAATTCCTAGTGTAGCGGTGAAATGCGTAGATATTAGGAGGAACACCAGTGGCGAAGGCGGCCTGCTGGACGGTAACTGACGTTGAGGCTCGAAAGCGTGGGGAGCAAACAGG
ASV034	Bacteria	Firmicutes	Clostridia	Lachnospirales	Lachnospiraceae	A2	TACGTATGGAGCAAGCGTTATCCGGATTTACTGGGCGTAAAGGGAGCGTAGGCGGCCTGCCAAGTCTGATGTGAAATACCGGGGCTCAACCCCGGGGCTGCATTGGAAACTGGCAGGCTGGAGTGCCGGAGAGGCAGGCGGAATTCCTAGTGTAGCGGTGAAATGCGTAGATATTAGGAGGAACACCAGTGGCGAAGGCGGCCTGCTGGACGGTGACTGACGCTGAGGCTCGAAAGCGTGGGGAGCAAACAGG
ASV475	Bacteria	Firmicutes	Clostridia	Lachnospirales	Lachnospiraceae	Lachnospira	TACGTATGGAGCAAGCGTTATCCGGATTTACTGGGTGTAAAGGGAGTGTAGGTGGCATCACAAGTCAGAAGTGAAAGCCCGGGGCTCAACCCCGGGACTGCTTTTGAAACTGTGGAGCTGGAGTGCAGGAGAGGCAAGTGGAATTCCTAGTGTAGCGGTGAAATGCGTAGATATTAGGAGGAACACCAGTGGCGAAGGCGGCTTGCTGGACTGTAACTGACACTGAGGCTCGAAAGCGTGGGGAGCAAACAGG
ASV010	Bacteria	Firmicutes	Clostridia	Lachnospirales	Lachnospiraceae	Lachnospiraceae_Genus	TACGTATGGAGCAAGCGTTATCCGGATTTACTGGGTGTAAAGGGAGAGTAGGTGGCAGAGCAAGTCCGAAGTGAAAACCCAAAGCTCAACTATGGGAATGCTTTAGAAACTGCTCAGCTAGAGTGCAGGAGAGGCAAGTGGAATTCTTAGTGTAGCGGTGAAATGCGTAGATATTAGGAGGAACACCAGTGGCGAAGGCGGCTTGCTGGACTGTAACTGACACTGAGGCTCGAAAGCGTGGGGAGCAAACAGG
ASV461	Bacteria	Firmicutes	Clostridia	Lachnospirales	Lachnospiraceae	Lachnospira	TACGTATGGAGCAAGCGTTATCCGGATTTACTGGGTGTAAAGGGAGTGTAGGTGGCCAGGCAAGTCAGAAGTGAAAGCCCGGGGCTCAACCCCGGGACTGCTTTTGAAACTGCAGGGCTAGAGTGCAGGAGGGGCAAGTGGAATTCCTAGTGTAGCGGTGAAATGCGTAGATATTAGGAGGAACACCAGTGGCGAAGGCGGCTTGCTGGACTGTAACTGACACTGAGGCTCGAAAGCGTGGGGAGCAAACAGG
ASV196	Bacteria	Firmicutes	Clostridia	Lachnospirales	Lachnospiraceae	Lachnospiraceae_Genus	TACGTAGGGGGCGAGCGTTATCCGGAATTACTGGGTGTAAAGGGTGAGTAGGCGGCTGTGCAAGTCATATGTGAAATACAGGGGCTTAACCCCTGTGCCGCATAAGAAACTGTACAGCTTAGAGTGCAGGAGAGGCAAGCGGAATTCCTGGTGTAGCGGTGAAATGCGTAGATATCAGGAAGAACACCGGTGGCGAAGGCGGCTTGCTGGACTGTAACTGACGCTGAGTCACGAAAGCGTGGGGAGCGAACAGG
ASV207	Bacteria	Firmicutes	Clostridia	Peptostreptococcales-Tissierellales	Peptostreptococcaceae	Romboutsia	TACGTAGGGGGCTAGCGTTATCCGGAATTACTGGGCGTAAAGGGTGCGTAGGTGGTTTCTTAAGTCAGAGGTGAAAGGCTACGGCTCAACCGTAGTAAGCCTTTGAAACTGGGAAACTTGAGTGCAGGAGAGGAGAGTGGAATTCCTAGTGTAGCGGTGAAATGCGTAGATATTAGGAGGAACACCAGTTGCGAAGGCGGCTCTCTGGACTGTAACTGACACTGAGGCACGAAAGCGTGGGGAGCAAACAGG

ASV209	Bacteria	Firmicutes	Clostridia	Peptostreptococcales-Tissierellales	Peptostreptococcaceae	Romboutsia	TACGTAGGGGGCTAGCGTTATCCGGAATTACTGGGCGTAAAGGGTGCGTAGGTGGTTTCTTAAGTCAGAGGTGAAAGGCTACGGCTCAACCGTAGTAAGCCTTTGAAACTGGGGAACTTGAGTGCAGGAGAGGAGAGTGGAATTCCTAGTGTAGCGGTGAAATGCGTAGATATTAGGAGGAACACCAGTTGCGAAGGCGGCTCTCTGGACTGTAACTGACACTGAGGCACGAAAGCGTGGGGAGCAAACAGG
ASV443	Bacteria	Firmicutes	Clostridia	Peptostreptococcales-Tissierellales	Peptostreptococcaceae	Romboutsia	TACGCAGGGGGCTAGCGTTATCCGGAATTACTGGGCGTAAAGGGTGCGTAGGTGGTTTCTTAAGTCAGAGGTGAAAGGCTACGGCTCAACCGTAGTAAGCCTTTGAAACTGGGGAACTTGAGTGCAGGAGAGGAGAGTGGAATTCCTAGTGTAGCGGTGAAATGCGTAGATATTAGGAGGAACACCAGTTGCGAAGGCGGCTCTCTGGACTGTAACTGACACTGAGGCACGAAAGCGTGGGGAGCAAACAGG
ASV240	Bacteria	Firmicutes	Clostridia	Peptostreptococcales-Tissierellales	Anaerovoracaceae	Anaerovoraceae_Genus	TACGTAGGGGGCAAGCGTTATCCGGAATTATTGGGCGTAAAGAGTACGTAGGCGGTTCTTTAAGCGCAGGGTTTAAGGCGATAGCTTAACTATCGTTCGCCCTGTGAACTGGGGGACTTGAGTATCGGAGAGGAAAGCGGAATTCCTAGTGTAGCGGTGAAATGCGTAGATATTAGGAGGAACACCAGTGGCGAAGGCGGCTTTCTGGACGAAAACTGACGCTGAGGTACGAAAGCGTGGGGAGCAAACAGG
ASV338	Bacteria	Firmicutes	Clostridia	Peptostreptococcales-Tissierellales	Anaerovoracaceae	Anaerovoraceae_Genus	CACGTAGGGGGCGAGCGTTATCCGGAATCATTGGGCGTAAAGGGTGCGTAGGCGGCCTTTTAAGCGCGGGGTCTAAGGCGGTGGCCCAACCACCGTTCGCCCCGCGAACTGGAGGGCTAGAGTGTCGGAGAGGGAAGCGGAATTCCTAGTGTAGCGGTGAAATGCGTAGATATTAGGAGGAACACCAGTGGCGAAGGCGGCTTCCTGGACGAAGACTGACGCTGAGGCACGAAAGCGTGGGGAGCGAACAGG
ASV150	Bacteria	Firmicutes	Clostridia	Peptostreptococcales-Tissierellales	Anaerovoracaceae	Anaerovoraceae_Genus	TACGTAGGGGGCAAGCGTTATCCGGAATTATTGGGCGTAAAGAGTACGTAGGTGGCAACCTAAGCGCAGGGTTTAAGGCAATGGCTCAACCATTGTTCGCCCTGCGAACTGGGATGCTTGAGTGCAGGAGAGGAAAGCGGAATTCCTAGTGTAGCGGTGAAATGCGTAGATATTAGGAGGAACACCAGTGGCGAAGGCGGCTTTCTGGACTGTAACTGACACTGAGGTACGAAAGCGTGGGGAGCAAACAGG
ASV234	Bacteria	Firmicutes	Clostridia	Peptostreptococcales-Tissierellales	Anaerovoracaceae	Family_XIII_AD3011_group	TACGTAGGGGGCAAGCGTTATCCGGAATTATTGGGCGTAAAGAGTGCGTAGGTGGTTACCTAAGCAGGGGGTGAAAGGCAATGGCTCAACCATTGTTAGCCCTCTGAACTGGGTTACTTGAGTGCAGGAGAGGAAAGCGGAATTCCTAGTGTAGCGGTGAAATGCGTAGATATTAGGAGGAACACCAGTGGCGAAGGCGGCTTTCTGGACTGAAACTGACACTGAGGCACGAAAGTGTGGGGAGCAAACAGG
ASV297	Bacteria	Firmicutes	Clostridia	Peptostreptococcales-Tissierellales	Anaerovoracaceae	Family_XIII_AD3011_group	TACGTAGGGGGCAAGCGTTATCCGGAATTATTGGGCGTAAAGAGTGCGTAGGTGGTTACTTAAGCGCGGGGTTTAAGGCAATGGCTCAACCATTGTTCGCCCTGCGAACTGGGTTACTTGAGTGCAGGAGAGGAAAGCGGAATTCCTAGTGTAGCGGTGAAATGCGTAGATATTAGGAGGAACACCAGTGGCGAAGGCGGCTTTCTGGACTGTTACTGACACTGAGGCACGAAAGTGTGGGGAGCAAACAGG
ASV307	Bacteria	Firmicutes	Clostridia	Peptococcales	Peptococcaceae	Peptococcaceae_Genus	TACGTAGGGAGCGAGCGTTGTCCGGAATTACTGGGCGTAAAGGGCGCGTAGGCGGGAACTTAAGTTAGATGTGAAAACTCCGGGCTTAACCTGGAGACTGCATTTAAAACTGGGTTTCTTGAGGGCAGGAGAGGAAAGTGGAATTCCTAGTGTAGCGGTGAAATGCGTAGATATTAGGAGGAACACCAGTGGCGAAGGCGACTTTCTGGACTGTACCTGACGCTGAGGCGCGAAAGCATGGGGAGCGAACAGG
ASV	Bacteria	Firmicutes	Clostridia	Peptococcales	Peptococcaceae	Peptococcaceae_Genus	TACGTAGGGAGCAAGCGTTGTCCGGAATTACTGGGCGTAAAGGGCGCGTAGGTGGGCGCTTAAGTCAGGTGTGAAAACTCCGGGCTCAACCTGGAGACTGCACTTGAAACTGGGTGTCTTGAGGGCAGGAGAGGAAAGTGGAATTCCTAGTGTAGCGGTGAAATGCGTAGATATTAGGAGGAACACCAGTGGCGAAGGCGACTTTCTGGACTGTACCTGACGCTGAGGCGCGAAAGCGTGGGTAGCGAACGGG

221							
ASV517	Bacteria	Firmicutes	Clostridia	Peptococcales	Peptococcaceae	Peptococcaceae_Genus	TACGGAGGATGCGAGCGTTATCCGGATTTATTGGGTTTAAAGGGCGCGTAGGTGGGCGCTTAAGTCAGGTGTGAAAACTCCGGGCTCAACCTGGAGACTGCACTTGAAACTGGGTGTCTTGAGGGCAGGAGAGGAAAGTGGAATTCCTAGTGTAGCGGTGAAATGCGTAGATATTAGGAGGAACACCAGTGGCGAAGGCGACTTTCTGGACTGTACCTGACGCTGAGGCGCGAAAGCGTGGGTAGCGAACGGG
ASV464	Bacteria	Firmicutes	Clostridia	Clostridiales	Clostridiaceae	Clostridiaceae_Genus	TACGTAGGGGGCAAGCGTTGTCCGGATTTACTGGGCGTAAAGGGAGCGTAGGCGGATTTTTAAGTGGGATGTGAAATACCCGGGCTCAACTTGGGTGCTGCATTCCAAACTGGAAGTCTAGAGTGCAGGAGAGGAAAGTGGAATTCCTAGTGTAGCGGTGAAATGCGTAGAGATTAGGAGGAACACCAGTGGCGAAGGCGGCTTTCTGGACTGTAACTGACACTGAGGTACGAAAGCGTGGGGAGCAAACAGG
ASV527	Bacteria	Firmicutes	Clostridia	Clostridiales	Clostridiaceae	Clostridium_sensu_stricto_1	TACGTAGGTGGCAAGCGTTGTCCGGATTTACTGGGCGTAAAGGGAGCGTAGGCGGATTTTTAAGTGGGATGTGAAATACCCGGGCTCAACTTGGGTGCTGCATTCCAAACTGGAAGTCTAGAGTGCAGGAGAGGAAAGTGGAATTCCTAGTGTAGCGGTGAAATGCGTAGAGATTAGGAAGAACACCAGTGGCGAAGGCGACTTTCTGGACTGTAACTGACGCTGAGGCTCGAAAGCGTGGGGAGCAAACAGG
ASV080	Bacteria	Firmicutes	Clostridia	Clostridiales	Clostridiaceae	Clostridium_sensu_stricto_1	TACGTAGGTGGCGAGCGTTGTCCGGATTTACTGGGCGTAAAGGGAGCGTAGGCGGACTTTTAAGTGAGATGTGAAATACTCGGGCTCAACTTGAGTGCTGCATTTCAAACTGGAAGTCTAGAGTGCAGGAGAGGAGAATGGAATTCTTAGTGTAGCGGTGAAATGCGTAGAGATTAGGAAGAACACCAGTGGCGAAGGCGATTCTCTGGACTGTAACTGACGCTGAGGCTCGAAAGCGTGGGGAGCAAACAGG
ASV155	Bacteria	Firmicutes	Clostridia	Clostridiales	Clostridiaceae	Clostridium_sensu_stricto_1	TACGTAGGTGGCGAGCGTTGTCCGGATTTACTGGGCGTAAAGGGAGCGTAGGCGGACTTTTAAGTGAGATGTGAAATACTCGGGCTCAACTTGAGTGCTGCATTTCAAACTGTAAGTCTAGAGTGCAGGAGAGGAGAATGGAATTCCTAGTGTAGCGGTGAAATGCGTAGAGATTAGGAAGAACACCAGTGGCGAAGGCGATTCTCTGGACTGTAACTGACGCTGAGGCTCGAAAGCGTGGGGAGCAAACAGG
ASV025	Bacteria	Firmicutes	Clostridia	Clostridiales	Clostridiaceae	Clostridium_sensu_stricto_1	TACGTAGGTGGCGAGCGTTGTCCGGATTTACTGGGCGTAAAGGGAGCGTAGGCGGACTTTTAAGTGAGATGTGAAATACTCGGGCTCAACTTGAGTGCTGCATTTCAAACTGGAAGTCTAGAGTGCAGGAGAGGAGAATGGAATTCCTAGTGTAGCGGTGAAATGCGTAGAGATTAGGAAGAACACCAGTGGCGAAGGCGATTCTCTGGACTGTAACTGACGCTGAGGCTCGAAAGCGTGGGGAGCAAACAGG
ASV607	Bacteria	Firmicutes	Clostridia	Clostridiales	Clostridiaceae	Clostridium_sensu_stricto_6	TACGTAGGTGGCAAGCGTTGTCCGGATTTACTGGGCGTAAAGGGAGCGTAGGCGGACCTTTAAGTGAGATGTGAAATACCCGAGCTCAACTTGGGTGCTGCATTTCAAACTGGAGGTCTAGAGTGTCGGAGAGGTAAGTGGAATTCCTAGTGTAGCGGTGAAATGCGTAGAGATTAGGAAGAACCCCGGTGGCGAAGGCGACTTACTGGACGATAACTGACGCTGAGGCTCGAAAGCGTGGGGAGCAAACAGG
ASV434	Bacteria	Firmicutes	Firmicutes_Class	Firmicutes_Class_Order	Firmicutes_Class_Order_Family	Firmicutes_Class_Order_Family_Genus	TACGTAGGGGGCAAGCGTTATCCGGAATGACTGGGCGTAAAGGGTGCGTAGGTGGTTTGGCAAGTTAGTAGCGTAACTCCGGGGCTCAACTCCGGAACTACTACTAAAACTGTCGGACTTGAGTGCAGAAGAGGGAAGTGGAATTCCATGTGTAGCGGTGAAATGCGTAGAGATATGGAGGAACACCAGTGGCGAAGGCGGCTTCCTGGTCTGCAACTGACACTGAGGCGCGAAAGCGTGGGGAGCAAACAGG

ASV0611	Bacteria	Firmicutes	Clostridia	Clostridia_vadinBB60_group	Clostridia_vadinBB60_group_Family	Clostridia_vadinBB60_group_Family_Genus	TACGTAGGAGGCAAGCGTTATCCGGAATGACTGGGCGTAAAGGGTGCGTAGGTGGTTTGACAAGTTAGTAGCGCAACTCCGGAGCTTAACTTCGGAACTACTACTAAAACTGTTGGACTTGAGTGCAGGAGGGGCAATCGGAATTCCTAGTGTAGCGGTGGAATGCGTAGATATTAGGAGGAACACCAGTGGCGAAGGCGGATTGCTAGACTGTAACTGACACTGAGGCACGAAAGCGTGGGGAGCAAACAGG
ASV3500	Bacteria	Firmicutes	Clostridia	Clostridia_vadinBB60_group	Clostridia_vadinBB60_group_Family	Clostridia_vadinBB60_group_Family_Genus	TACGTAGGAGGCAAGCGTTATCCGGAATGACTGGGCGTAAAGGGTGCGTAGGTGGTTTGATAAGTTAGTAGCGCAACTCCGGAGCTTAACTTCGGAACTACTACTAAAACTGTCGGACTTGAGTGCAGAAGGGGCAACCGGAATTCCTAGTGTAGCGGTGGAATGCGTAGATATTAGGAGGAACACCAGTGGCGAAGGCGGGTTGCTAGGCTGTAACTGACACTGAGGCACGAAAGCGTGGGGAGCAAACAGG
ASV1388	Bacteria	Firmicutes	Clostridia	Clostridia_vadinBB60_group	Clostridia_vadinBB60_group_Family	Clostridia_vadinBB60_group_Family_Genus	TACGTAGGAGGCAAGCGTTATCCGGAATGACTGGGCGTAAAGGGTGCGTAGGTGGTTTGACAAGTTGGTAGCGTAACTCCGGGGCTCAACCTCGGCACTACTACCAAAACTGTTGGACTTGAGTGCAGGAGGGGCAAATGGAATTCCTAGTGTAGCGGTGGAATGCGTAGATATTAGGAGGAACACCAGTGGCGAAGGCGATTTGCTGGACTGTAACTGACACTGAGGCACGAAAGCGTGGGGAGCAAACAGG
ASV1277	Bacteria	Firmicutes	Clostridia	Clostridia_vadinBB60_group	Clostridia_vadinBB60_group_Family	Clostridia_vadinBB60_group_Family_Genus	TACGTAGGAGGCAAGCGTTATCCGGAATGACTGGGCGTAAAGGGTGCGTAGGTGGTTTGACAAGTTGGTAGCGTAACTCCGAGGCTTAACCTCGGCACTACTACCAAAACTGTTGGACTCGAGTGCAGGAGGGGCAAATGGAATTCCTAGTGTAGCGGTGGAATGCGTAGATATTAGGAGGAACACCAGTGGCGAAGGCGATTTGCTGGACTGTAACTGACACTGAGGCACGAAAGCGTGGGGAGCAAACAGG
ASV2100	Bacteria	Firmicutes	Clostridia	Clostridia_vadinBB60_group	Clostridia_vadinBB60_group_Family	Clostridia_vadinBB60_group_Family_Genus	TACGTAGGAGGCAAGCGTTATCCGGAATGACTGGGCGTAAAGGGTGCGTAGGTGGTTTGACAAGTTGGTAGCGTAATTCCGAGGCTCAACCTCGGCACTACTACCAAAACTGTTGGACTTGAGTGCAGGAGGGGCAACTGGAATTCCTAGTGTAGCGGTGGAATGCGTAGATATTAGGAGGAACACCAGTGGCGAAGGCGAGTTGCTGGACTGTAACTGACACTGAGGCACGAAAGCGTGGGGAGCAAACAGG
ASV0655	Bacteria	Firmicutes	Clostridia	Clostridia_vadinBB60_group	Clostridia_vadinBB60_group_Family	Clostridia_vadinBB60_group_Family_Genus	TACGTAGGAGGCAAGCGTTATCCGGAATGACTGGGCGTAAAGGGTGCGTAGGTGGTTTGGCAAGTTAGTAGCGTAACTCCGGGGCTCAACTCCGGAACTACTACTAAAACTGTCGGACTTGAGTGCAGGAGGGGCAATTGGAATTCCTAGTGTAGCGGTGGAATGCGTAGATATTAGGAAGAACACCAGTGGCGAAGGCGAATTGCTGGACTGTAACTGACACTGAGGCACGAAAGCGTGGGGAGCAAACAGG
ASV2633	Bacteria	Firmicutes	Clostridia	Clostridia_vadinBB60_group	Clostridia_vadinBB60_group_Family	Clostridia_vadinBB60_group_Family_Genus	TACGTAGGAGGCAAGCGTTATCCGGAATGACTGGGCGTAAAGGGTGCGTAGGTGGTTTGACAAGTTAGTAGCGTAATTCCGGGGCTCAACTTCGGAACTACTACTAAAACTGTTGGGCTTGAGTGCAGGAGAGGTGAATGGAATTCCTAGTGTAGCGGTGGAATGCGTAGATATTAGGAGGAACACCAGCGGCGAAAGCGATTCACTGGACTGTAACTGACACTGAGGCACGAAAGCGTGGGGAGCAAACAGG
ASV2222	Bacteria	Firmicutes	Clostridia	Clostridia_vadinBB60_group	Clostridia_vadinBB60_group_Family	Clostridia_vadinBB60_group_Family_Genus	TACGTAGGAGGCAAGCGTTATCCGGAATGACTGGGCGTAAAGGGTGCGTAGGTGGTTTGACAAGTTGGTAGCGTAACTCCGGGGCTCAACCTCGGATCTACTACCAAAACTGTTGGGCTTGAGTGCAGGAGGGGTGAATGGAATTCCTAGTGTAGCGGTGGAATGCGTAGATATTAGGAGGAACACCAGCGGCGAAGGCGATTCACTGGACTGTAACTGACACTGAGGCACGAAAGCGTGGGGAGCAAACAGG
ASV	Bacteria	Firmicutes	Clostridia	Clostridia_vadinBB60_group	Clostridia_vadinBB60_group_Family	Clostridia_vadinBB60_group_Family_Genus	TACGTAGGAGGCAAGCGTTATCCGGAATGACTGGGCGTAAAGGGTGCGTAGGTGGTTTGGCAAGTTGGTAGCGTAATCCCGGGGCTCAACCTCGGTGCTACTACCAAAACTGTTGAACTTGAGTGCAGGAGGGGTGAATGGAATTCCCAGTGTAGCGGTGGAATGCGTAGATATTGGGAAGAACACCAGCGGCGAAGGCGATTCACTGGACTGTAACTGACACTGAGGCACGAAAGCGTGGGGAGCAAACAGG

255							
ASV329	Bacteria	Firmicutes	Clostridia	Clostridia_vadinBB60_group	Clostridia_vadinBB60_group_Family	Clostridia_vadinBB60_group_Family_Genus	TACGTAGGAGGCAAGCGTTATCCGGAATGACTGGGCGTAAAGGGTGCGTAGGTGGTTTGGCAAGTTGGTAGCGTAATTCCGGGGCTCAACCTCGGCGCTACTACCAAAACTGCTGGACTTGAGTGCAGGAGGGGTGAATGGAATTCCTAGTGTAGCGGTGGAATGCGTAGATATTAGGAAGAACACCAGCGGCGAAGGCGATTCACTGGACTGTAACTGACACTGAGGCACGAAAGCGTGGGGAGCAAACAGG
ASV146	Bacteria	Firmicutes	Clostridia	Clostridia_UCG-014	Clostridia_UCG-014_Family	Clostridia_UCG-014_Family_Genus	TACGTAGGGAGCGAGCGTTATCCGGAATTATTGGGTGTAAAGGGTGCGTAGACGGGAAAGCAAGTTAGTTGTGAAATCCCTCGGCTTAACTGAGGAACTGCAACTAAAACTACTTTTCTTGAGTGCAGGAGAGGTAAGTGGAATTCCTAGTGTAGCGGTGAAATGCGTAGATATTAGGAGGAACACCAGTGGCGAAGGCGACTTACTGGACTGTAACTGACGTTGAGGCACGAAAGTGTGGGGAGCAAACAGG
ASV201	Bacteria	Firmicutes	Clostridia	Clostridia_UCG-014	Clostridia_UCG-014_Family	Clostridia_UCG-014_Family_Genus	TACGTAGGGAGCGAGCGTTATCCGGAATTATTGGGTGTAAAGGGTGCGTAGACGGGAAAACAAGTTAGTTGTGAAATCCCTCGGCTTAACTGAGGAACTGCAACTAAAACTATTTTTCTTGAGTGCAGGAGAGGTAAGTGGAATTCCTAGTGTAGCGGTGAAATGCGTAGATATTAGGAGGAACACCAGTGGCGAAGGCGACTTACTGGACTGTAACTGACGTTGAGGCACGAAAGTGTGGGGAGCAAACAGG
ASV283	Bacteria	Firmicutes	Clostridia	Clostridia_UCG-014	Clostridia_UCG-014_Family	Clostridia_UCG-014_Family_Genus	TACGTAGGGAGCGAGCGTTATCCGGATTTATTGGGTGTAAAGGGTGCGTAGACGGGAAAACAAGTTAGTTGTGAAATCCCTCGGCTTAACTGAGGAACTGCAACTAAAACTATTTTTCTTGAGTGCAGGAGAGGTAAGTGGAATTCCTAGTGTAGCGGTGAAATGCGTAGATATTAGGAGGAACACCAGTGGCGAAGGCGACTTACTGGACTGTAACTGACGTTGAGGCACGAAAGTGTGGGGAGCAAACAGG
ASV162	Bacteria	Firmicutes	Clostridia	Clostridia_UCG-014	Clostridia_UCG-014_Family	Clostridia_UCG-014_Family_Genus	TACGTAGGGAGCAAGCGTTATCCGGATTTATTGGGTGTAAAGGGTGCGTAGACGGGCTAACAAGTTAGTTGTGAAATCCCTCGGCTTAACTGAGGAACTGCAACTAAAACTATTAGTCTCTGAGTGCGGGAGAGGAAAGTGGAATTCCTAGTGTAGCGGTGAAATGCGTAGATATTAGGAGGAACACCAGTGGCGAAGGCGACTTTCTGGACCGTAACTGACGTTGAGGCACGAAAGTGTGGGGAGCAAACAGG
ASV241	Bacteria	Firmicutes	Clostridia	Clostridia_UCG-014	Clostridia_UCG-014_Family	Clostridia_UCG-014_Family_Genus	TACGTAGGGAGCAAGCGTTATCCGGATTTATTGGGTGTAAAGGGTGCGTAGACGGGAAAACAAGTTAGTTGTGAAATCCCTCAGCTTAACTGAGGAACTGCAACTAAAACTATTTTTCTTGAGTGCTGGAGAGGAAAGTGGAATTCCTAGTGTAGCGGTGAAATGCGTAGATATTAGGAGGAACACCAGTGGCGAAGGCGACTTTCTGGACAGTAACTGACGTTGAGGCACGAAAGTGTGGGGAGCAAACAGG
ASV217	Bacteria	Firmicutes	Clostridia	Clostridia_UCG-014	Clostridia_UCG-014_Family	Clostridia_UCG-014_Family_Genus	TACATAGGGAGCGAGCGTTATCCGGATTTATTGGGTGTAAAGGGTGCGTAGACGGGAATACAAGTTGGTTGTGAAATCCCTCGGCTCAACCGAGGAACTGCAACCAAAACTATATTTCTTGAGTACTGGAGAGGAAAGTGGAATTCCTAGTGTAGCGGTGAAATGCGTAGATATTAGGAGGAACACCAGTGGCGAAGGCGACTTTCTGGACAGAAACTGACGTTGAGGCACGAAAGTGTGGGGAGCAAACAGG
ASV364	Bacteria	Firmicutes	Clostridia	Clostridia_UCG-014	Clostridia_UCG-014_Family	Clostridia_UCG-014_Family_Genus	TACATAGGGAGCGAGCGTTATCCGGATTTATTGGGTGTAAAGGGTGCGTAGACGGGAATACAAGTTGGTTGTGAAAGCCCTCGGCTTAACCGAGGAACTGCAACCGAAACTATATTTCTTGAGTATCGGAGAGGAAAGTGGAATTCCTAGTGTAGCGGTGAAATGCGTAGATATTAGGAGGAACACCAGTGGCGAAGGCGACTTTCTGGACGAAAACTGACGTTGAGGCACGAAAGTGTGGGGAGCAAACAGG

ASV340	Bacteria	Firmicutes	Clostridia	Clostridia_UCG-014	Clostridia_UCG-014_Family	Clostridia_UCG-014_Family_Genus	TACGTAGGGAGCGAGCGTTATCCGGATTTATTGGGTGTAAAGGGTGCGTAGACGGGTATACAAGTTGGTTGTGAAATCCCTCGGCTTAACTGAGGAACTGCAACCAAAACTGTATATCTTGAGTACAGGAGAGGTAAGTGGAATTCCTAGTGTAGCGGTGAAATGCGTAGATATTAGGAGGAACACCAGTGGCGAAGGCGACTTACTGGACTGAAACTGACGTTGAGGCACGAAAGTGTGGGGAGCAAACAGG
ASV184	Bacteria	Firmicutes	Clostridia	Clostridia_UCG-014	Clostridia_UCG-014_Family	Clostridia_UCG-014_Family_Genus	TACGTAGGGAGCAAGCGTTATCCGGATTTATTGGGTGTAAAGGGTGCGTAGATGGATAAACAAGTTGTTTGTGAAATACCTCGGCTCAACTGAGGAACTGCAAGCAAAACTGTATATCTTGAGTACTGGAGGGGAAAGCGGAATTCCTAGTGTAGCGGTGAAATGCGTAGATATTAGGAAGAACACCGGTGGCGAAGGCGGCTTTCTGGACAGAAACTGACATTGAGGCACGAAAGTGTGGGGAGCAAACAGG
ASV139	Bacteria	Firmicutes	Clostridia	Clostridia_UCG-014	Clostridia_UCG-014_Family	Clostridia_UCG-014_Family_Genus	TACGTAGGGAGCAAGCGTTATCCGGATTTATTGGGTGTAAAGGGTGCGTAGACGGGAAGGCAAGTTGGTTGTGAAATCCCTCGGCTTAACTGAGGAACTGCAACCAAAACTATCTTTCTTGAGTGCTGGAGAGGAAAGTGGAATTCCTAGTGTAGCGGTGAAATGCGTAGATATTAGGAGGAACACCAGTGGCGAAGGCGACTTTCTGGACAGTAACTGACGTTGAGGCACGAAAGTGTGGGGAGCAAACAGG
ASV121	Bacteria	Firmicutes	Clostridia	Clostridia_UCG-014	Clostridia_UCG-014_Family	Clostridia_UCG-014_Family_Genus	TACGTAGGGAGCAAGCGTTATCCGGATTTATTGGGTGTAAAGGGTGCGTAGACGGGAAGGCAAGTTAGTTGTGAAATCCCTCGGCTTAACTGAGGAACTGCAACTAAAACTATCTTTCTTGAGTGCTGGAGAGGAAAGTGGAATTCCTAGTGTAGCGGTGAAATGCGTAGATATTAGGAGGAACACCAGTGGCGAAGGCGACTTTCTGGACAGTAACTGACGTTGAGGCACGAAAGTGTGGGGAGCAAACAGG
ASV316	Bacteria	Firmicutes	Clostridia	Clostridia_UCG-014	Clostridia_UCG-014_Family	Clostridia_UCG-014_Family_Genus	TACGTAGGGAGCAAGCGTTATCCGGATTTATTGGGTGTAAAGGGTGCGTAGACGGGAAGTCAAGTTAGTTGTGAAATCCCTCGGCTTAACTGAGGAACTGCAACTAAAACTAACTTTCTTGAGTGCTGGAGAGGAAAGTGGAATTCCTAGTGTAGCGGTGAAATGCGTAGATATTAGGAGGAACACCAGTGGCGAAGGCGACTTTCTGGACAGTAACTGACGTTGAGGCACGAAAGTGTGGGGAGCAAACAGG
ASV339	Bacteria	Firmicutes	Clostridia	Clostridia_UCG-014	Clostridia_UCG-014_Family	Clostridia_UCG-014_Family_Genus	TACGTAGGGAGCAAGCGTTATCCGGATTTATTGGGCGTAAAGGGTGCGTAGACGGGAAGGCAAGTTGGTTGTGAAATCCCTCGGCTTAACTGAGGAACTGCAACCAAAACTATCTTTCTTGAGTGCTGGAGAGGAAAGTGGAATTCCTAGTGTAGCGGTGAAATGCGTAGATATTAGGAGGAACACCAGTGGCGAAGGCGACTTTCTGGACAGTAACTGACGTTGAGGCACGAAAGTGTGGGGAGCAAACAGG
ASV134	Bacteria	Firmicutes	Clostridia	Clostridia_UCG-014	Clostridia_UCG-014_Family	Clostridia_UCG-014_Family_Genus	TACGTAGGGAGCAAGCGTTATCCGGATTTATTGGGTGTAAAGGGTGCGTAGACGGGAAGGCAAGTTGGTTGTGAAATCCCTCGGCTTAACTGAGGAACTGCAACCAAAACTACATTTCTTGAGTGCTGGAGAGGAAAGTGGAATTCCTAGTGTAGCGGTGAAATGCGTAGATATTAGGAGGAACACCAGTGGCGAAGGCGACTTTCTGGACAGTAACTGACGTTGAGGCACGAAAGTGTGGGGAGCAAACAGG
ASV206	Bacteria	Firmicutes	Clostridia	Clostridia_UCG-014	Clostridia_UCG-014_Family	Clostridia_UCG-014_Family_Genus	TACGTAGGGAGCAAGCGTTATCCGGATTTATTGGGTGTAAAGGGTGCGTAGACGGGAATGCAAGTTAGTTGTGAAATCCCTCGGCTTAACTGAGGAACTGCAACTAAAACTACATTTCTTGAGTGCTGGAGAGGAAAGTGGAATTCCTAGTGTAGCGGTGAAATGCGTAGATATTAGGAGGAACACCAGTGGCGAAGGCGACTTTCTGGACAGTAACTGACGTTGAGGCACGAAAGTGTGGGGAGCAAACAGG
ASV	Bacteria	Firmicutes	Clostridia	Clostridia_UCG-014	Clostridia_UCG-014_Family	Clostridia_UCG-014_Family_Genus	TACGTAGGGAGCAAGCGTTATCCGGATTTATTGGGTGTAAAGGGTGCGTAGACGGGAATGCAAGTTGGTTGTGAAATCCCTCGGCTTAACTGAGGAACTGCAACCAAAACTACATTTCTTGAGTGCTGGAGAGGAAAGTGGAATTCCTAGTGTAGCGGTGAAATGCGTAGATATTAGGAGGAACACCAGTGGCGAAGGCGACTTTCTGGACAGTAACTGACGTTGAGGCACGAAAGTGTGGGGAGCAAACAGG

346							
ASV390	Bacteria	Firmicutes	Clostridia	Clostridia_UCG-014	Clostridia_UCG-014_Family	Clostridia_UCG-014_Family_Genus	TACGTAGGGAGCAAGCGTTATCCGGATTTATTGGGTGTAAAGGGTGCGTAGACGGGAAGACAAGTTGGTTGTGAAATCCCTCGGCTTAACTGAGGAACTGCAACCAAAACTATCTTTCTTGAGTGCTGGAGAGGAAAGTGGAATTCCTAGTGTAGCGGTGAAATGCGTAGATATTAGGAGGAACACCAGTGGCGAAGGCGACTTTCTGGACAGTAACTGACGTTGAGGCACGAAAGTGTGGGGAGCAAACAGG
ASV300	Bacteria	Firmicutes	Clostridia	Clostridia_UCG-014	Clostridia_UCG-014_Family	Clostridia_UCG-014_Family_Genus	TACGTAGGGAGCAAGCGTTATCCGGATTTATTGGGTGTAAAGGGTGCGTAGACGGGAAAACAAGTTGGTTGTGAAATCCCTCGGCTTAACTGAGGAACTGCAACCAAAACTATTTTTCTTGAGTGCTGGAGAGGAAAGTGGAATTCCTAGTGTAGCGGTGAAATGCGTAGATATTAGGAGGAACACCAGTGGCGAAGGCGACTTTCTGGACAGTAACTGACGTTGAGGCACGAAAGTGTGGGGAGCAAACAGG
ASV185	Bacteria	Firmicutes	Clostridia	Clostridia_UCG-014	Clostridia_UCG-014_Family	Clostridia_UCG-014_Family_Genus	TACATAGGGAGCAAGCGTTATCCGGATTTATTGGGTGTAAAGGGTGCGTAGACGGGAAATTAAGTTAGTTGTGAAATCCCTCGGCTTAACTGAGGAACTGCAACTAAAACTGATTTTCTTGAGTACTGGAGAGGAAAGTGGAATTCCTAGTGTAGCGGTGAAATGCGTAGATATTAGGAGGAACACCAGTGGCGAAGGCGACTTTCTGGACAGTAACTGACGTTGAGGCACGAAAGTGTGGGGAGCAAACAGG
ASV203	Bacteria	Firmicutes	Clostridia	Clostridia_UCG-014	Clostridia_UCG-014_Family	Clostridia_UCG-014_Family_Genus	TACATAGGGGGCGAGCGTTATCCGGAATTATTGGGTGTAAAGGGTGCGTAGACGGGAAATTAAGTTAGTTGTGAAATCCCTTGGCTTAACTGAGGAACTGCAACTAAAACTGATTTTCTTGAGTATTGGAGAGGAAAGTGGAATTCCTAGTGTAGCGGTGAAATGCGTAGATATTAGGAGGAACACCAGTGGCGAAGGCGACTTTCTGGACAAAAACTGACGTTGAGGCACGAAAGTGTGGGGAGCAAACAGG
ASV324	Bacteria	Firmicutes	Clostridia	Clostridia_UCG-014	Clostridia_UCG-014_Family	Clostridia_UCG-014_Family_Genus	TACATAGGGGGCAAGCGTTATCCGGAATTATTGGGTGTAAAGGGTGCGTAGACGGGAAATTAAGTTAGTTGTGAAATACCTTGGCTCAACTAAGGAACTGCAACTAAAACTGGTTTTCTTGAGTACTGGAGAGGAAAGTGGAATTCCTAGTGTAGCGGTGAAATGCGTAGATATTAGGAGGAACACCAGTGGCGAAGGCGACTTTCTGGACAGAAACTGACGTTGAGGCACGAAAGTGTGGGGAGCAAACAGG
ASV367	Bacteria	Firmicutes	Clostridia	Clostridia_UCG-014	Clostridia_UCG-014_Family	Clostridia_UCG-014_Family_Genus	TACGTAGGGAGCAAGCGTTATCCGGATTTATTGGGTGTAAAGGGTGCGTAGACGGGAATTTAAGTTAGTTGTGAAATCCCTCGGCTCAACTGAGGAACTGCAACTAAAACTGGATTTCTTGAGTGCTGGAGAGGAAAGCGGAATTCCTAGTGTAGCGGTGAAATGCGTAGATATTAGGAGGAACACCAGTGGCGAAGGCGGCTTTCTGGACAGTAACTGACGTTGAGGCACGAAAGTGTGGGGAGCAAACAGG
ASV157	Bacteria	Firmicutes	Clostridia	Clostridia_UCG-014	Clostridia_UCG-014_Family	Clostridia_UCG-014_Family_Genus	TACATAGGGAGCGAGCGTTATCCGGATTTATTGGGTGTAAAGGGTGCGTAGACGGGAATTTAAGTTGGTTGTGAAATCCCTCAGCTCAACTGAGGAACTGCAACCAAAACTGGATTTCTTGAGTGCTGGAGAGGAAAGTGGAATTCCTAGTGTAGCGGTGAAATGCGTAGATATTAGGAGGAACACCAGTGGCGAAGGCGACTTTCTGGACAGTAACTGACGTTGAGGCACGAAAGTGTGGGGAGCAAACAGG
ASV313	Bacteria	Firmicutes	Clostridia	Clostridia_UCG-014	Clostridia_UCG-014_Family	Clostridia_UCG-014_Family_Genus	TACATAGGGAGCAAGCGTTATCCGGATTTATTGGGTGTAAAGGGTGCGTAGACGGGAATTTAAGTTGGTTGTGAAAGCCCTCGGCTCAACTGAGGAACTGCAACCAAAACTGGATTTCTTGAGTATTGGAGAGGAAAGTGGAATTCCTAGTGTAGCGGTGAAATGCGTAGATATTAGGAGGAACACCAGTGGCGAAGGCGGCTTTCTGGACAATAACTGACGTTGAGGCACGAAAGTGTGGGGAGCAAACAGG

ASV172	Bacteria	Firmicutes	Clostridia	Clostridia_UCG-014	Clostridia_UCG-014_Family	Clostridia_UCG-014_Family_Genus	TACGTAGGGAGCAAGCGTTATCCGGATTTATTGGGTGTAAAGGGTGCGTAGACGGAATTTAAGTTAGTTGTGAAATCCCTCGGCTTAACTGAGGAACTGCAACTAAAACTGAATTTCTTGAGTGCGGGAGAGGAAAGTGGAATTCCTAGTGTAGCGGTGAAATGCGTAGATATTAGGAGGAACACCAGTGGCGAAGGCGACTTTCTGGACCGTAACTGACGTTGAGGCACGAAAGTGTGGGGAGCAAACAGG
ASV361	Bacteria	Firmicutes	Clostridia	Clostridia_UCG-014	Clostridia_UCG-014_Family	Clostridia_UCG-014_Family_Genus	TACGTAGGGAGCAAGCGTTATCCGGAATTATTGGGTGTAAAGGGTGCGTAGACGGAAAATAAGTTAGTTGTGAAATCCCTCGGCTCAACTGAGGAACTGCAACTAAAACTGTAATTCTTGAGTGCAGGAGAGGAAAGTGGAATTCCTAGTGTAGCGGTGAAATGCGTAGATATTAGGAGGAACACCAGTGGCGAAGGCGACTTTCTGGACTGTAACTGACGTTGAGGCACGAAAGTGTGGGGAGCAAACAGG
ASV291	Bacteria	Firmicutes	Clostridia	Clostridia_UCG-014	Clostridia_UCG-014_Family	Clostridia_UCG-014_Family_Genus	TACGTAGGGAGCAAGCGTTATCCGGAATTATTGGGTGTAAAGGGTGCGTAGACGGAGTCTAAGTTAGTTGTGAAATCCCTCGGCTCAACTGAGGAACTGCAACTAAAACTGGGCATCTTGAGTGCAGGAGAGGAAAGTGGAATTCCTAGTGTAGCGGTGAAATGCGTAGATATTAGGAGGAACACCAGTGGCGAAGGCGACTTTCTGGACTGTAACTGACGTTGAGGCACGAAAGTGTGGGGAGCAAACAGG
ASV386	Bacteria	Firmicutes	Clostridia	Clostridia_UCG-014	Clostridia_UCG-014_Family	Clostridia_UCG-014_Family_Genus	TACGTAGGGAGCGAGCGTTATCCGGAATTATTGGGTGTAAAGGGTGCGTAGACGGTAAATAAGTTAGTTGTGAAATCCCTCGGCTCAACTGAGGAACTGCAACTAAAACTGTATATCTTGAGTGCGGGAGAGGAAAGTGGAATTCCTAGTGTAGCGGTGAAATGCGTAGATATTAGGAGGAACACCAGTGGCGAAGGCGACTTTCTGGACCGTAACTGACGTTGAGGCACGAAAGTGTGGGGAGCAAACAGG
ASV274	Bacteria	Firmicutes	Clostridia	Clostridia_UCG-014	Clostridia_UCG-014_Family	Clostridia_UCG-014_Family_Genus	TACGTAGGGAGCAAGCGTTATCCGGATTTATTGGGTGTAAAGGGTGCGTAGACGGTATATAAGTTAGTTGTGAAATCCCTCGGCTCAACTGAGGAACTGCAACTAAAACTGTAAATCTTGAGTGCGGGAGAGGAAAGTGGAATTCCTAGTGTAGCGGTGAAATGCGTAGATATTAGGAGGAACACCAGTGGCGAAGGCGACTTTCTGGACCGTAACTGACGTTGAGGCACGAAAGTGTGGGGAGCAAACAGG
ASV198	Bacteria	Firmicutes	Clostridia	Clostridia_UCG-014	Clostridia_UCG-014_Family	Clostridia_UCG-014_Family_Genus	TACGTAGGGAGCAAGCGTTATCCGGATTTATTGGGTGTAAAGGGTGCGTAGACGGAAAACAAGTTAGTTGTGAAATCCCTCGGCTTAACTGAGGAACTGCAACTAAAACTATTTTTCTTGAGTGCTGGAGAGGAAAGTGGAATTCCTAGTGTAGCGGTGAAATGCGTAGATATTAGGAGGAACACCAGTGGCGAAGGCGACTTTCTGGACAGTAACTGACGTTGAGGCACGAAAGTGTGGGGAGCAAACAGG
ASV260	Bacteria	Firmicutes	Clostridia	Clostridia_UCG-014	Clostridia_UCG-014_Family	Clostridia_UCG-014_Family_Genus	TACATAGGGGGCAAGCGTTATCCGGATTTATTGGGTGTAAAGGGTGCGTAGACGGAGAAACAAGTTGGTTGTGAAATCCCTCGGCTCAACTGAGGAACTGCAACCAAAACTATTTCCCTTGAGTGTCGGAGAGGAAAGTGGAATTCCTAGTGTAGCGGTGAAATGCGTAGATATTAGGAGGAACACCGGTGGCGAAGGCGACTTTCTGGACGATAACTGACGTTGAGGCACGAAAGTGTGGGGAGCAAACAGG
ASV392	Bacteria	Firmicutes	Clostridia	Clostridia_UCG-014	Clostridia_UCG-014_Family	Clostridia_UCG-014_Family_Genus	TACGTAGGGAGCAAGCGTTATCCGGATTTATTGGGTGTAAAGGGTGCGTAGACGGAGAAGCAAGTTAGTTGTGAAATCCCTCGGCTTAACTGAGGAACTGCAACTAAAACTACTTCCCTTGAGTGTTGGAGAGGAAAGTGGAATTCCTAGTGTAGCGGTGAAATGCGTAGATATTAGGAGGAACACCAGTGGCGAAGGCGACTTTCTGGACAATAACTGACGTTGAGGCACGAAAGTGTGGGGAGCAAACAGG
ASV	Bacteria	Firmicutes	Clostridia	Clostridia_UCG-014	Clostridia_UCG-014_Family	Clostridia_UCG-014_Family_Genus	TACGTAGGGAGCAAGCGTTATCCGGAATTATTGGGTGTAAAGGGTGCGTAGACGGAATACAAGTTAGTTGTGAAAGCCCTCGGCTTAACTGAGGAACTGCAACTAAAACTATATTTCTTGAGTGCAGGAGAGGAAAGCGGAATTCCTAGTGTAGCGGTGAAATGCGTAGATATTAGGAGGAACACCAGTGGCGAAGGCGGCTTTCTGGACTGTAACTGACGTTGAGGCACGAAAGTGTGGGGAGCAAACAGG

268							
ASV377	Bacteria	Firmicutes	Clostridia	Clostridia_UCG-014	Clostridia_UCG-014_Family	Clostridia_UCG-014_Family_Genus	TACGTAGGGAGCAAGCGTTATCCGGAATTATTGGGTGTAAAGGGTGCGTAGACGGGACAGCAAGTTAGTTGTGAAAGCCCTCGGCTTAACTGAGGAACTGCAACTAAAACTATTGTTCTTGAGTGCAGGAGAGGAAAGCGGAATTCCTAGTGTAGCGGTGAAATGCGTAGATATTAGGAGGAACACCAGTGGCGAAGGCGGCTTTCTGGACTGTAACTGACGTTGAGGCACGAAAGTGTGGGGAGCAAACAGG
ASV212	Bacteria	Firmicutes	Clostridia	Clostridia_UCG-014	Clostridia_UCG-014_Family	Clostridia_UCG-014_Family_Genus	TACGTAGGGAGCAAGCGTTATCCGGAATTATTGGGTGTAAAGGGTGCGTAGACGGGATAACAAGTTGGTTGTGAAACCCCTCGGCTCAACTGAGGAACTGCAACCAAAACTATTATTCTTGAGTGCAGGAGAGGAAAGTGGAATTCCTAGTGTAGCGGTGAAATGCGTAGATATTAGGAAGAACACCGGTGGCGAAGGCGACTTTCTGGACTGTAACTGACGTTGAGGCACGAAAGTGTGGGGAGCAAACAGG
ASV414	Bacteria	Firmicutes	Clostridia	Clostridia_UCG-014	Clostridia_UCG-014_Family	Clostridia_UCG-014_Family_Genus	TACGTAGGGAGCAAGCGTTATCCGGATTTATTGGGTGTAAAGGGTGCGTAGACGGGATTACAAGTTGGTTGTGAAACCCCTCAGCTCAACTGAGGAACTGCAACCAAAACTATAATTCTTGAGTGCAGGAGAGGAAAGTGGAATTCCTAGTGTAGCGGTGAAATGCGTAGATATTAGGAGGAACACCAGTGGCGAAGGCGGCCTGCTGGACGGTGACTGACGCTGAGGCTCGAAAGCGTGGGGAGCAAACAGG
ASV328	Bacteria	Firmicutes	Clostridia	Clostridia_UCG-014	Clostridia_UCG-014_Family	Clostridia_UCG-014_Family_Genus	TACGTAGGGAGCAAGCGTTATCCGGATTTATTGGGTGTAAAGGGTGCGTAGACGGGATTACAAGTTGGTTGTGAAACCCCTCAGCTCAACTGAGGAACTGCAACCAAAACTATAATTCTTGAGTGCAGGAGAGGAAAGTGGAATTCCTAGTGTAGCGGTGAAATGCGTAGATATTAGGAAGAACACCGGTGGCGAAGGCGACTTTCTGGACTGTAACTGACGTTGAGGCACGAAAGTGTGGGGAGCAAACAGG
ASV423	Bacteria	Firmicutes	Clostridia	Clostridia_UCG-014	Clostridia_UCG-014_Family	Clostridia_UCG-014_Family_Genus	TACGTAGGGAGCAAGCGTTATCCGGAATTATTGGGTGTAAAGGGTGCGTAGACGGGATAACAAGTTGGTTGTGAAACCCCTCGGCTCAACTGAGGAACTGCAACCAAAACTATTATTCTTGAGTGCAGGAGAGGAAAGTGGAATTCCTAGTGTAGCGGTGAAATGCGTAGATATTAGGAGGAACACCAGTGGCGAAGGCGGCTTACTGGACTGTAACTGACGTTGAGGCTCGAAAGCGTGGGGAGCAAACAGG
ASV312	Bacteria	Firmicutes	Clostridia	Clostridia_vadinBB60_group	Clostridia_vadinBB60_group_Family	Clostridia_vadinBB60_group_Family_Genus	TACGTAGGGGGCGAGCGTTGTCCGGAATGACTGGGCGTAAAGGGAGTGTAGGCGGCTGTGCAAGTTAGATGTGAAATCCCATGGCTTAACCATGGAACTGCATCTAAAACTGCATAGCTGGAGTGCAGGAGAGGTGAGTGGAATTCCTAGTGTAGCGGTGGAATGCGTAGATATTAGGAGGAACACCAGAGGCGAAGGCGACTCACTGGACTGTAACTGACGCTGAGGCTCGAAAGCGTGGGGAGCAAACAGG
ASV211	Bacteria	Firmicutes	Clostridia	Clostridia_vadinBB60_group	Clostridia_vadinBB60_group_Family	Clostridia_vadinBB60_group_Family_Genus	TACGTAGGGGGCAAGCGTTGTCCGGAATGACTGGGCGTAAAGGGAGTGTAGGCGGCTTATCAAGTTAGATGTGAAATCCCGCGGCTTAACCGCGGAACTGCATCTAAAACTGGTGAGCTGGAGTGCAGGAGAGGTGAGTGGAATTCCTAGTGTAGCGGTGGAATGCGTAGATATTAGGAGGAACACCAGAGGCGAAGGCGACTCACTGGACTGTAACTGACGCTGAGGCTCGAAAGCGTGGGGAGCAAACAGG
ASV270	Bacteria	Firmicutes	Clostridia	Clostridia_vadinBB60_group	Clostridia_vadinBB60_group_Family	Clostridia_vadinBB60_group_Family_Genus	TACGTAGGGGGCAAGCGTTGTCCGGAATGACTGGGCGTAAAGGGAGTGTAGGCGGCCTTGTAAGTTAGATGTGAAATCCTACGGCTTAACCGTAGAACTGCATCTAAAACTGTGAGGCTGGAGTGCAGGAGAGGTGAGTGGAATTCCTAGTGTAGCGGTGGAATGCGTAGATATTAGGAGGAACACCAGAGGCGAAGGCGACTCACTGGACTGTAACTGACGCTGAGGCTCGAAAGCGTGGGGAGCAAACAGG

ASV251	Bacteria	Firmicutes	Clostridia	Clostridia_vadinBB60_group	Clostridia_vadinBB60_group_Family	Clostridia_vadinBB60_group_Family_Genus	TACGTAGGGGGCGAGCGTTGTCCGGAATGACTGGGCGTAAAGGGAGTGTAGGCGGCTCTTTAAGTTATATGTGAAATCCCGCGGCTTAACCGTGGAAGTGCATATAAAACTGGGGAGCTGGAGTGCAGGAGAGGTGAGTGGAATTCCTAGTGTAGCGGTGGAATGCGTAGATATTAGGAAGAACACCGGTGGCGAAGGCGGCTCACTGGACTGTAACTGACGCTGAGGCTCGAAAGCGTGGGGAGCAAACAGG
ASV306	Bacteria	Firmicutes	Clostridia	Clostridia_Order	Clostridia_Order_Family	Clostridia_Order_Family_Genus	TACGTAGGGGGCGAGCGTTGTCCGGAATGACTGGGCGTAAAGGGAGTGTAGGCGGCCTTTTAAGTTATGTGTGAAAGCCCACGGCTCAACTGTGGAATTGCACATAAAACTGGGAGGCTGGAGTGCAGGAGAGGTAAGCGGAATTTCCAGTGTAGCGGTGGAATGCGTAGATATTGGGAAGAACACCGGAGGCGAAGGCGGCTTACTGGACTGTAACTGACGCTGAGGCTCGAAAGCGTGGGGAGCAAACAGG
ASV531	Bacteria	Firmicutes	Clostridia	Clostridia_Order	Clostridia_Order_Family	Clostridia_Order_Family_Genus	TACGTAGGGGGCGAGCGTTATCCGGATTCACTGGGCGTAAAGGGAGTGTAGGCGGCCTTTTAAGTTATGTGTGAAAGCCCACGGCTCAACTGTGGAATTGCACATAAAACTGGGAGGCTGGAGTGCAGGAGAGGTAAGCGGAATTTCCAGTGTAGCGGTGGAATGCGTAGATATTGGGAAGAACACCGGAGGCGAAGGCGGCTTACTGGACTGTAACTGACGCTGAGGCTCGAAAGCGTGGGGAGCAAACAGG
ASV175	Bacteria	Firmicutes	Clostridia	Clostridiales	Clostridiaceae	Candidatus_Arthromitus	TACGTAGGTGGCAAGCGTTGTTCGGAATAACTGGGCGTAAAGGATGCGTAGGCGGTTAAACAAGTTATATGTTAAATATATAGGCTTAACCTGTAGAAAGCATATAAAACTGTTTAACTAGAGTGCAGGAGAGGTAAGTGGAATTCCTAGTGTAGCGGTGAAATGCGTAGAGATTAGGAAGAACACCAGTGGCGAAGGCGACTTACTGGACTGTAACTGACGCTGAGGCATGAGAGCATGGGGAGCAAACAGG
ASV167	Bacteria	Firmicutes	Clostridia	Clostridia_vadinBB60_group	Clostridia_vadinBB60_group_Family	Clostridia_vadinBB60_group_Family_Genus	TACGTAGGGAGCAAGCGTTGTTCGGAATGACTGGGCGTAAAGGGTGCGTAGGCGGTTGTACAAGTTAGAAGTGAAATACCCAGGGCTTAACTCGGGTGCTGCTTCTAAAACTGTATGACTTGAGTGCAGTAGAGGTTAGTGGAATTCCTAGTGTAGCGGTGGAATGCGTAGATATTAGGAGGAACACCAGAGGCGAAGGCGGCTAACTGGACTGCAACTGACGCTGAGGCACGAAAGTGTGGGGAGCAAACAGG
ASV259	Bacteria	Firmicutes	Clostridia	Clostridia_vadinBB60_group	Clostridia_vadinBB60_group_Family	Clostridia_vadinBB60_group_Family_Genus	TACGTAGGGAGCAAGCGTTGTTCGGAATGACTGGGCGTAAAGGGTGCGTAGGCGGTTATACAAGTTAGAAGTGAAATACCCAGGGCTTAACTCGGGTGCTGCTTCTAAAACTGTAAGACTTGAGTACAGTAGAGGTTAGTGGAATTCCTAGTGTAGCGGTGGAATGCGTAGATATTAGGAGGAACACCAGAGGCGAAGGCGGCTAACTGGACTGAAACTGACGCTGAGGCACGAAAGTGTGGGGAGCAAACAGG
ASV452	Bacteria	Actinobacteriota	Actinobacteria	Bifidobacteriales	Bifidobacteriaceae	Bifidobacterium	TACGTAGGGTGCAAGCGTTATCCGGAATTATTGGGCGTAAAGGGCTCGTAGGCGGTTCGTCGCGTCCGGTGTGAAAGTCCATCGCTTAACGGTGGATCCGCGCCGGGTACGGGCGGGCTTGAGTGCGGTAGGGGAGACTGGAATTCCCGGTGTAACGGTGGAATGTGTAGATATCGGGAAGAACACCAATGGCGAAGGCAGGTCTCTGGGCCGTCACTGACGCTGAGGAGCGAAAGCGTGGGGAGCGAACAGG
ASV116	Bacteria	Actinobacteriota	Actinobacteria	Bifidobacteriales	Bifidobacteriaceae	Bifidobacterium	TACGTAGGGTGCAAGCGTTATCCGGATTTATTGGGCGTAAAGGGCTCGTAGGCGGTTCGTCGCGTCCGGTGTGAAAGTCCATCGCTTAACGGTGGATCCGCGCCGGGTACGGGCGGGCTTGAGTGCGGTAGGGGAGACTGGAATTCCCGGTGTAACGGTGGAATGTGTAGATATCGGGAAGAACACCAATGGCGAAGGCAGGTCTCTGGGCCGTTACTGACGCTGAGGAGCGAAAGCGTGGGGAGCGAACAGG
ASV	Bacteria	Firmicutes	Bacilli	RF39	RF39_Family	RF39_Family_Genus	TACGTAGGTGGCGAGCGTTATCCGGATTTATTGGGCGTAAAGCGTGCGCAGGCGGCTTATTAAGTCTAAGATTAAAGCCCGGAGCTTAACTCCGGTTCGTCTTAGAAACTGGTAGGCTTGAGTATGGTAGAGGCAAACGGAATTCCTAGTGTAGCGGTGGAATGCGTAGATATTAGGAGGAACACCAGTGGCGAAGGCGGTTTGCTGGGCCATTACTGACGCTCATGCACGAAAGCGTGGGGAGCAAATAGG

237							
ASV341	Bacteria	Firmicutes	Bacilli	RF39	RF39_Family	RF39_Family_Genus	TACGTAGGTGGCGAGCGTTATCCGGATTTATTGGGCGTAAAGCGTGCGCAGGCGGTTTGTTAAGTCTAAAATTAAAGTCCGAAGCTTAACTTCGGTTCGTTTTAGAAACTGGCAGGCTTGAGTATGGTAGAGGCAAACGGAATTTCTAGTGTAGCGGTAAAATGCGTAGATATTAGAAGGAACACCAGTGGCGAAGGCGGTTTGCTGGCCATTACTGACGCTCATGCACGAAAGCGTGGGGAGCAAATAGG
ASV489	Bacteria	Firmicutes	Bacilli	RF39	RF39_Family	RF39_Family_Genus	TACGTAGGTGGCGAGCGTTATCCGGATTTATTGGGCGTAAAGCGTGCGCAGGCGGTTTGTTAGGTCTAAAATTAAAGCCCGAAGCTTAACTTCGGTTCGTTTTAGAAACCGGCAGGCTTGAGTATGGTAGAGGCAAACGGAATTTCTAGTGTAGCGGTAAAATGCGTAGATATTAGAAGGAACACCAGTGGCGAAGGCGGTTTGCTGGCCATTACTGACGCTCATGCACGAAAGCGTGGGGAGCAAATAGG
ASV214	Bacteria	Firmicutes	Bacilli	RF39	RF39_Family	RF39_Family_Genus	TACGTAGGTGGCGAGCGTTATCCGGATTTATTGGGCGTAAAGCGTGCGCAGGCGGTTTGTTAGGTCTAAAATTAAAGTCCGAAGCTTAACTTCGGTTCGTTTTAGAAACCGGCAGGCTTGAGTATGGTAGAGGCAAACGGAATTTCTAGTGTAGCGGTAAAATGCGTAGATATTAGAAGGAACACCAGTGGCGAAGGCGGTTTGCTGGCCATTACTGACGCTCATGCACGAAAGCGTGGGGAGCAAATAGG
ASV397	Bacteria	Firmicutes	Bacilli	RF39	RF39_Family	RF39_Family_Genus	TACGTAGGGGGCGAGCGTTATCCGGATTTATTGGGCGTAAAGGGTGTGTAGGCGGTTTGTTAAGTCTAAAATCAAAGCCCGAGGCTTAACCTCGGTTCGTTTTAGAAACTGGCAAACTAGAGTACAGTAGAGGCAAGTGGAATTTCTAGTGTAGCGGTTAAATGCGTAGATATTAGAAGGAACACCAGTGGCGAAGGCGGCTTGCTGGGCTGTTACTGACGCTGAGACACGAAAGCGTGGGGAGCAAATAGG
ASV266	Bacteria	Firmicutes	Bacilli	RF39	RF39_Family	RF39_Family_Genus	TACGTAGGGGGCGAGCGTTATCCGGATTTATTGGGCGTAAAGCGTGTGTAGGCGGTTTATTAAGTCTAAGATTAAAGCCTGGAGCTTAACTTCAGTTCGTCTTAGAAACTGGTAGACTTGAGTGTGGTAGAGGCAAGTGGAATTTCTAGTGTAGCGGTTAAATGCGTAGATATTAGAAGGAACACCAGTGGCGAAGGCGACTTGCTGGCCATTACTGACGCTGAGACACGAAAGCGTGGGGAGCAAATAGG
ASV371	Bacteria	Firmicutes	Bacilli	RF39	RF39_Family	RF39_Family_Genus	TACGTAGGGGGCGAGCGTTATCCGGATTTATTGGGCGTAAAGCGTGTGTAGGCGGTTTATTAAGTATAAGATTAAAGCCCGAGGCTTAACCTCGGTTCGTCTTATAAACTGGTAGACTTGAGTGTGGTAGAGGCAAGTGGAATTTCTAGTGTAGCGGTTAAATGCGTAGATATTAGAAGGAACACCAGTGGCGAAGGCGGCTTGCTGGCCATTACTGACGCTGAGACACGAAAGCGTGGGGAGCAAATAGG
ASV508	Bacteria	Firmicutes	Bacilli	RF39	RF39_Family	RF39_Family_Genus	TACGGAGGGGGCGAGCGTTATCCGGATTTATTGGGCGTAAAGCGTGTGTAGGCGGTTTATTAAGTATAAGATTAAAGCCCGAGGCTTAACCTCGGTTCGTCTTATAAACTGGTAGACTTGAGTGTGGTAGAGGCAAGTGGAATTTCTAGTGTAGCGGTTAAATGCGTAGATATTAGAAGGAACACCAGTGGCGAAGGCGGCTTGCTGGCCATTACTGACGCTGAGACACGAAAGCGTGGGGAGCAAATAGG
ASV604	Bacteria	Firmicutes	Bacilli	Bacilli_Order	Bacilli_Order_Family	Bacilli_Order_Family_Genus	TACGTAGGTGGCGAGCGTTATCCGGAATTATTGGGCGTAAAGAGTGCGCAGGCGGTTTGATAAGTCTAAAATAAAAGCCCGAAGCTTAACTTCGGTTCGTTTTAGAAACTGTCAGACTAGAGTGTGGTAGAGGCAAATGGAATTTCTAGTGCAGCGGTAAAATGCGTAGATATCGCGCAGAACTCCGATTGCGAAGGCAGCATGCCGGCTCCACACTGACGCTGAGGCACGAAAGCGTGGGTATCGAACAGG
AS	Bacteria_	Bacteria_Ph	Bacteria_Phylu	Bacteria_Phylum_Cl	Bacteria_Phylum_Class_	Bact	TACGTAGGTGGCGAGCGTTATCCGGATTTATTGGGCGTAAAGCGTGCGCAGGCGGCTTATTAAGTCTAAGATTAAAGCCCGGAGCTTAACTCCGGTTCGTCTTAGAAACTGGTAGGCTTGAGTATGGTAGAGGCAAACGGAATT

V583	eria	Phylum	ylum_Class	m_Class_Order	ass_Order_Family	Order_Family_Genus	CCTAGTGTAGCGGTGAAATGCATAGATATCGCGCAGAACTCCGATTGCGAAGGCAGCATGCCGGCTCCACACTGACGCTGAGGCACGAAAGCGTGGGTATCGAACAGG
ASV037	Bacteria	Firmicutes	Bacilli	RF39	RF39_Family	RF39_Family_Genus	TACGTAGGTGGCGAGCGTTATCCGGATTTATTGGGCGTAAAGCGTCCGCAGCCGGTTTATTAAGTATAGAATAAAACTTCGGGGCTCAACCCCGTCTCGTTCTATAAACTGATAGACTAGAGTGTGGTAGAGGCAAGTGGAATTTCTAGTGTAGCGGTAGAATGCGTAGATATTAGAAGGAACACCAGTGGCGAAGGCGACTTGCTGGGCCATCACTGACGGTCAGGGACGAAAGCGTGGGGAGCGAATAGG
ASV599	Bacteria	Firmicutes	Bacilli	Bacilli_Order	Bacilli_Order_Family	Bacilli_Order_Family_Genus	TACGTAGGTGGCGAGCGTTATCCGGAATTATTGGGCGTAAAGAGTGCGCAGGCGGTTTGATAAGTCTAAAATAAAAGCCCGAAGCTTAACTTCGGTTCGTTTTAGAAACTGTCAGACTAGAGTGTGGTAGAGGCAAATGGAATTTCTAGTGTAGCGGTAAAATGCGTAGATATTAGGAGGAACACCGATTGCGAAGGCAGCCTGCTGGACTGTAACTGACGCTGATGCTCGAAAGTGTGGGTATCAAACAGG
ASV262	Bacteria	Firmicutes	Bacilli	RF39	RF39_Family	RF39_Family_Genus	TACGTAGGTGGCGAGCGTTATCCGGAATTATTGGGCGTAAAGAGTGCGCAGGCGGTTTGATAAGTCTAAAATAAAAGCCCGAAGCTTAACTTCGGTTCGTTTTAGAAACTGTCAGACTAGAGTGTGGTAGAGGCAAATGGAATTTCTAGTGTAGCGGTAAAATGCGTAGATATTAGAAGGAACACCAGTGGCGAAGGCGATTTGCTGGGCCACAACTGACGCTCATGCACGAAAGCGTGGGGAGCAAATAGG
ASV553	Bacteria	Firmicutes	Bacilli	RF39	RF39_Family	RF39_Family_Genus	TACGTAGGTGGCGAGCGTTATCCGGATTTATTGGGCGTAAAGCGTGCGCAGGCGGCTTATTAAGTCTAAGATTAAAGCCCGGAGCTTAACTCCGGTTCGTCTTAGAAACTGGTAGGCTTGAGTATGGTAGAGGCAAACGGAATTCCTAGTGTAGCGGTGGAATGCGTAGATATTAGGAGGAACACCAGTGGCGAAGGCGGCTTACTGGACCGTAACTGACGTTGAGGCTCGAAAGCGTGGGGAGCAAACAGG
ASV238	Bacteria	Actinobacteriota	Coriobacteriia	Coriobacteriales	Eggerthellaceae	Enterorhabdus	TACGTAGGGGGCGAGCGTTATCCGGATTCATTGGGCGTAAAGCGCGCGTAGGCGGCCGCCTAAGCGGAACCTCTAACCCCGGGGCTCAACCTCGGGCCGGGTTCCGGACTGGGCGGCTCGAGTGCGGTAGAGGAGAGCGGAATTCCCGGTGTAGCGGTGGAATGCGCAGATATCGGGAAGAACACCGATGGCGAAGGCAGCTCTCTGGGCCGTCACTGACGCTGAGGCGCGAAAGCTGGGGGAGCGAACAGG
ASV336	Bacteria	Actinobacteriota	Coriobacteriia	Coriobacteriales	Eggerthellaceae	Enterorhabdus	TACGTAGGGAGCGAGCGTTATCCGGATTCATTGGGCGTAAAGCGCGCGTAGGCGGCTGCCTAAGCGGAACCTCTAACCCCGGGGCTCAACCTCGGGCCGGGTTCCGAACTGGGCGGCTCGAGTGCGGTAGAGGAGAGCGGAATTCCCGGTGTAGCGGTGGAATGCGCAGATATCGGGAAGAACACCGATGGCGAAGGCAGCTCTCTGGGCCGGCACTGACGCTGAGGCGCGAAAGCTGGGGGAGCGAACAGG
ASV253	Bacteria	Actinobacteriota	Coriobacteriia	Coriobacteriales	Eggerthellaceae	Parvibacter	TACGTAGGGAGCGAGCGTTATCCGGATTCATTGGGCGTAAAGCGCGCGTAGGCGGCTGCCTAAGCGGAACCTCTAATCCCGGGGCTCAACCTCGGGCCGGGTTCCGAACTGGGCGGCTCGAGTGCGGTAGAGGCAGGCGGAATTCCCGGTGTAGCGGTGGAATGCGCAGATATCGGGAAGAACACCGATGGCGAAGGCAGCCTGCTGGGCCGTCACTGACGCTGAGGCGCGAAAGCTGGGGGAGCGAACAGG
ASV193	Bacteria	Actinobacteriota	Coriobacteriia	Coriobacteriales	Eggerthellaceae	Adlercreutzia	TACGTAGGGGGCGAGCGTTATCCGGATTCATTGGGCGTAAAGCGCGCGTAGGCGGCCGCCTAAGCGGAACCTCTAATCCCGGGGCTCAACCTCGGGCCGGGTTCCGGACTGGGCGGCTCGAGTGCGGTAGAGGCAGGCGGAATTCCCGGTGTAGCGGTGGAATGCGCAGATATCGGGAAGAACACCGATGGCGAAGGCAGCCTGCTGGGCCGCCACTGACGCTGAGGCGCGAAAGCTGGGGGAGCGAACAGG

ASV187	Bacteria	Actinobacteriota	Coriobacteriia	Coriobacteriales	Eggerthellaceae	DNF00809	TACGTAGGGGGCAAGCGTTATCCGGATTCATTGGGCGTAAAGCGCGCGCAGGCGGGCCGCTAAGCGGGACCTCTAATCTTGGGGCTCAACCTCAAGCCGGGTCCCGAACTGGCAGCCTCGAGTGCGGTAGGGGTAAGCGGAATTCCCGGTGTAGCGGTGGAATGCGCAGATATCGGGAAGAACACCGATGGCGAAGGCAGCTTACTGGGCCGACACTGACGCTGAGGCGCGAAAGCTAGGGGAGCGAACAGG
ASV332	Bacteria	Actinobacteriota	Coriobacteriia	Coriobacteriales	Eggerthellaceae	DNF00809	TACGTAGGGGGCGAGCGTTATCCGGATTCATTGGGCGTAAAGCGCGCGCAGGCGGACCGTTAAGCGGGACCTCTAATCCTGGGGCTCAACCTCAGGCCGGGTCCCGAACTGGCGGTCTCGAGTGCGGTAGGGGAGGTCGGAATTCCCGGTGTAGCGGTGGAATGCGCAGATATCGGGAAGAACACCGATGGCGAAGGCAGACCTCTGGGCCGACACTGACGCTGAGGCGCGAAAGCTAGGGGAGCGAACAGG
ASV292	Bacteria	Actinobacteriota	Coriobacteriia	Coriobacteriales	Eggerthellaceae	Enterorhabdus	TACGTAGGGGGCGAGCGTTATCCGGATTCATTGGGCGTAAAGCGCGCGCAGGCGGGCTTGCAAGCGGGGCCTTTAATCTTGGGGCTTAACCTCAAGTCGGGTTCCGAACTGCAAGCCTCGAGTGTGGTAGGGGAAGGCGGAATTCCCGGTGTAGCGGTGGAATGCGCAGATATCGGGAAGAACACCGATGGCGAAGGCAGCCTTCTGGGCCACTACTGACGCTGAGGCGCGAAAGCTGGGGGAGCGAACAGG
ASV153	Bacteria	Actinobacteriota	Coriobacteriia	Coriobacteriales	Eggerthellaceae	Eggerthellaceae_Genus	TACGTATGGGGCAAGCGTTATCCGGATTCATTGGGCGTAAAGCGCGCGCAGGCGGGCGCCTAAGCGGGACCTCTAATCTCGGGGCTCAACCCCGAGCCGGGTTCCGAACTGGGCACCTCGGGTTCGGTAGGGGTAGACGGAATTCCCGGTGTAGCGGTGGAATGCGCAGATATCGGGAAGAACACCGATGGCGAAGGCAGTCTACTGGGCCGCAACCGACGCTGAGGCGCGAAAGCTGGGGGAGCGAACAGG
ASV223	Bacteria	Actinobacteriota	Coriobacteriia	Coriobacteriales	Eggerthellaceae	DNF00809	TACGTAGGGAGCGAGCGTTATCCGGATTCATTGGGCGTAAAGCGCGCGTAGGCGGGCATCTAAGCGGGACCTCTAATCTCGGGGCTCAACCTCGAGCCGGGTTCCGAACTGGATGCCTCGAGTCCGGTAGGGGCAAGTGGAATTCCCGGTGTAGCGGTGGAATGCGCAGATATCGGGAAGAACACCGATGGCGAAGGCAGCTTGCTGGGCCGCGACTGACGCTGAGGCGCGAAAGCTAGGGGAGCGAACAGG
ASV564	Bacteria	Actinobacteriota	Coriobacteriia	Coriobacteriales	Eggerthellaceae	Eggerthellaceae_Genus	TACGTAGGTGGCAAGCGTTGTCCGGATTTATTGGGCGTAAAGCGCGCGTAGGCGGCGCGTCAAGCGGGACCTCTAACCCCGGGGCTCAACCCCGGGCCGGGTCCCGGACTGGCGCGCTCGAGTGCGGTAGAGGCGAGTGGAATTCCCGGTGTAGCGGTGGAATGCGTAGATATATGGAAGAACACCAGTGGCGAAGGCGGCTCTCTGGTCTGCAACTGACGCTGAGGCTCGAAAGCATGGGTAGCGAACAGG
ASV400	Bacteria	Actinobacteriota	Coriobacteriia	Coriobacteriales	Eggerthellaceae	DNF00809	TACGTAGGGGGCGAGCGTTATCCGGATTCATTGGGCGTAAAGCGCGCGTAGGCGGCGCGTCAAGCGGGACCTCTAACCCCGGGGCTCAACCCCGGGCCGGGTCCCGGACTGGCGCGCTCGAGTGCGGTAGAGGCGAGTGGAATTCCCGGTGTAGCGGTGGAATGCGCAGATATCGGGAAGAACACCGATGGCGAAGGCAGCTCGCTGGGCCGACACTGACGCTGAGGCGCGAAAGCTGGGGGAGCGAACAGG
ASV113	Bacteria	Actinobacteriota	Coriobacteriia	Coriobacteriales	Eggerthellaceae	Enterorhabdus	TACGTAGGGGGCGAGCGTTATCCGGATTCATTGGGCGTAAAGCGCGCGTAGGCGGCCGCCTAAGCGGGACCTCTAACCCCGGGGCTCAACCCCGGGCCGGGTCCCGGACTGGGCGGCTCGAGTGCGGTAGAGGAGAGCGGAATTCCCGGTGTAGCGGTGGAATGCGCAGATATCGGGAAGAACACCGATGGCGAAGGCAGCTCTCTGGGCCGTCACTGACGCTGAGGCGCGAAAGCTGGGGGAGCGAACAGG
ASV	Bacteria	Actinobacteriota	Coriobacteriia	Coriobacteriales	Eggerthellaceae	Enterorhabdus	TACGTAGGGGGCGAGCGTTATCCGGATTCATTGGGCGTAAAGCGCGCGTAGGCGGCCGCCTAAGCGGGACCTCTAACCCCGGGGCTCAACCCCGGGCCGGGTCCCGGACTGGGCGGCTCGAGTGCGGTTGAGGAGAGCGGAATTCCCGGTGTAGCGGTGGAATGCGCAGATATATGGAGGAACACCAGTGGCGAAGGCGGCTTACTGGACTGTAACTGACGTTGAGGCTCGAAAGCGTGGGGAGCAAACAGG

480							
ASV603	Bacteria	Proteobacteria	Gammaproteobacteria	Burkholderiales	Comamonadaceae	Comamonadaceae_Genus	TACGTAGGGTGCAAGCGTTAATCGGAATTACTGGGCGTAAAGCGTGCGCAGGCGGTTATGTAAGACAGAGGTGAAATCCCCGGGCTCAACCTGGGAACTGCCTTTGTGACTGCATGACTAGAGTACGGTAGAGGGGGATGGAATTCCACGTGTAGCAGTGAAATGCGTAGATATGTGGAGGAACACCGATGGCGAAGGCAATCCCCTGGACCTGTACTGACGCTCATGCACGAAAGCGTGGGGAGCAAACAGG
ASV428	Bacteria	Patescibacteria	Saccharimonadia	Saccharimonadales	Saccharimonadaceae	Candidatus_Saccharimonas	TACGTAGGATCCGAGCATTATCCGGAGTGACTGGGTGTAAAGAGTTGCGTAGGTGGCATAATAAGTAGCTAGTGAAATCTGGTGGCTCAACCATTCAGACTATTAGCTAAACTGTTAAGCTCGAGACCGTTAGGGGTAACTGGAATTTCTAGTGTAGGAGTGAAATCCGTAGATATTAGAAAGAACACCAATAGCGTAGGCAGGTTACTGGGACGGTTCTGACACTAAGGCACGAAAGCGTAGGGAGCAAACGGG
ASV486	Bacteria	Patescibacteria	Saccharimonadia	Saccharimonadales	Saccharimonadaceae	Candidatus_Saccharimonas	TACGTAGGACCCGAGCATTATCCGGAGTGACTGGGTGTAAAGAGTTGCGTAGGTGGCATAATAAGTAGCTAGTGAAATCTGGTGGCTCAACTATTCAGACTATTAGCTAAACTGTTAAGCTCGAGACCGTTAGGGGTAACTGGAATTTCTAGTGTAGGAGTGAAATCCGTAGATATTAGAAAGAACACCAATAGCGTAGGCAGGTTACTGGGACGGTTCTGACACTAAGGCACGAAAGCGTAGGGAGCAAACGGG
ASV213	Bacteria	Verrucomicrobiota	Chlamydiae	Chlamydiales	Chlamydiaceae	Chlamydia	TACGGAGGGTGCTAGCGTTAATCGGATTTATTGGGCGTAAAGGGCGTGTAGGCGGAAAGGTAAGTTAGTTGTCAAATCTCGGGGCTCAACCCCGAATCGGCATCTAAAACTATTTTTCTAGAGGGTAGATGGAGAAAAGGGAATTTCACGTGTAGCGGTGAAATGCGTAGATATGTGGAAGAACACCAGTGGCGAAGGCGCTTTTCTAATTTATACCTGACGCTAAGGCGCGAAAGCAAGGGGAGCAAACAGG
ASV567	Bacteria	Verrucomicrobiota	Verrucomicrobiae	Verrucomicrobiales	Akkermansiaceae	Akkermansia	TACAGAGGTCTCAAGCGTTGTTCGGAATCACTGGGCGTAAAGCGTGCGTAGGCTGTTTCGTAAGTCGTGTGTGAAAGGCGCGGGCTCAACCCGCGGACGGCACATGATACTGCGAGACTAGAGTAATGGAGGGGGAACCGGAATTCTCGGTGTAGCAGTGAAATGCGTAGATATCGAGAGGAACACTCGTGGCGAAGGCGGGTTCCTGGACATTAACTGACGCTGAGGCACGAAGGCCAGGGGAGCGAAAGGG
ASV542	Bacteria	Firmicutes	Clostridia	Lachnospirales	Lachnospiraceae	Lachnospiraceae_Genus	TACGTAGGGGGCAAGCGTTATCCGGATTTACTGGGTGTAAAGGGAGCGTAGACGGCTTGGCAAGCCAGATGTGAAAGGCTGGGGCTCAACCCCAGGACTGCATTTGAAACTGTTTTTCTTGAGTACCGGAGAGGTCATCGGAATTCCTTGTGTAGCGGTGAAATGCGTAGATATAAGGAAGAACACCAGTGGCGAAGGCGGCTTCCTGGTCTGCAACTGACACTGAGGCGCGAAAGCGTGGGGAGCAAACAGG
ASV429	Bacteria	Firmicutes	Clostridia	Lachnospirales	Lachnospiraceae	Lachnospiraceae_Genus	TACGTATGGGGCAAGCGTTATCCGGATTTACTGGGTGTAAAGGGAGCGTAGGCGGCCATGCAAGTCAGAAGTGAAAACCCGGGGCTCAACCCTGGGAGTGCTTTTGAAACTGTGCGGCTAGAGTGTCGGAGGGGTAAGTGGAATTCCATGTGTAGCGGTGAAATGCGTAGAGATATGGAGGAACACCAGTGGCGAAGGCGGCTTCCTGGTCTGCAACTGACACTGAGGCGCGAAAGCGTGGGGAGCAAACAGG
ASV052	Bacteria	Firmicutes	Clostridia	Lachnospirales	Lachnospiraceae	Lachnospiraceae_Genus	TACGTAGGGGGCAAGCGTTATCCGGATTTACTGGGTGTAAAGGGAGCGTAGGCGGCCATGCAAGTCAGAAGTGAAAACCCGGGGCTCAACCCTGGGAGTGCTTTTGAAACTGTGCGGCTAGAGTGTCGGAGGGGTAAGTGGAATTCCTAGTGTAGCGGTGAAATGCGTAGATATTAGGAGGAACACCAGTGGCGAAGGCGGCTTACTGGACGATGACTGACGCTGAGGCTCGAAAGCGTGGGGAGCAAACAGG

ASV068	Bacteria	Firmicutes	Clostridia	Lachnospirales	Lachnospiraceae	Lachnospiraceae_Genus	TACGTAGGGGGCAAGCGTTATCCGGATTTACTGGGTGTAAAGGGAGCGTAGGCGGCCACGCAAGTCAGAAGTGAAAACCCGGGGCTCAACCCTGGGAGTGCTTTTGAAACTGTGCGGCTAGAGTGTCGGAGGGGTAAGTGGAATTCCTAGTGTAGCGGTGAAATGCGTAGATATTAGGAGGAACACCAGTGGCGAAGGCGGCTTACTGGACGATGACTGACGCTGAGGCTCGAAAGCGTGGGGAGCAAACAGG
ASV117	Bacteria	Firmicutes	Clostridia	Lachnospirales	Lachnospiraceae	GCA-900066575	TACGTAGGGGGCAAGCGTTATCCGGATTTACTGGGTGTAAAGGGAGCGTAGGCGGCAGTGCAAGTCAGAAGTGAAAGCCCAAGGCTCAACCATGGGACTGCTTTTGAAACTGTACAGCTAGATTGCAGGAGAGGTAAGTGGAATTCCTAGTGTAGCGGTGAAATGCGTAGATATTAGGAGGAACACCAGTGGCGAAGGCGGCTTACTGGACTGTAAATGACGCTGAGGCTCGAAAGCGTGGGGAGCAAACAGG
ASV314	Bacteria	Firmicutes	Clostridia	Lachnospirales	Lachnospiraceae	Lachnospiraceae_Genus	TACGTAGGGGGCAAGCGTTATCCGGATTTACTGGGTGTAAAGGGAGCGTAGGCGGCAAGGTAAGCCAGAAGTGAAAGCCCGCGGCTCAACTGCGGGACTGCTTTTGGAACTATCTAGCTAGATTGCAGGAGAGGTAAGTGGAATTCCTAGTGTAGCGGTGAAATGCGTAGATATTAGGAGGAACACCAGTGGCGAAGGCGGCTTACTGGACTGTAAATGACGCTGAGGCTCGAAAGCGTGGGGAGCAAACAGG
ASV415	Bacteria	Firmicutes	Clostridia	Lachnospirales	Lachnospiraceae	Lachnospiraceae_UCG-010	TACGTAGGGGGCAAGCGTTATCCGGATTTACTGGGTGTAAAGGGAGCGTAGGCGGCATGGTAAGCCAGATGTGAAAGCCCGCGGCTTAACCGCGCGGATTGCATTTGGAACTATCAAGCTAGAGTACAGGAGAGGAAAGCGGAATTCCTAGTGTAGCGGTGAAATGCGTAGATATTAGGAAGAACACCAGTGGCGAAGGCGGCTTTCTGGACTGAAACTGACGCTGAGGCTCGAAAGCGTGGGGAGCAAACAGG
ASV277	Bacteria	Firmicutes	Clostridia	Lachnospirales	Lachnospiraceae	Lachnospiraceae_UCG-010	TACGTAGGGGGCAAGCGTTATCCGGAATTACTGGGTGTAAAGGGAGCGTAGGCGGCATGGTAAGCCAGATGTGAAAGCCCGCGGCTTAACCGCGCGGATTGCATTTGGAACTATCAAGCTAGAGTACAGGAGAGGAAAGCGGAATTCCTAGTGTAGCGGTGAAATGCGTAGATATTAGGAAGAACACCAGTGGCGAAGGCGGCTTTCTGGACTGAAACTGACGCTGAGGCTCGAAAGCGTGGGGAGCAAACAGG
ASV511	Bacteria	Firmicutes	Clostridia	Lachnospirales	Lachnospiraceae	Lachnospiraceae_UCG-010	TACGTAGGGGGCAAGCGTTATCCGGAATTACTGGGTGTAAAGGGAGCGTAGGCGGCATGGTAAGCCAGATGTGAAAGCCCGCGGCTTAACCGCGCGGATTGCATTTGGAACTATCAAGCTAGAGTACAGGAGAGGAAAGCGGAATTCCTAGTGTAGCGGTGAAATGCGTAGATATCAGGAGGAACACCGGCGGCGAAGGCGGCCTGCTGGACTGAAACTGACGTTGAGGCTCGAAAGCGTGGGGAGCAAACAGG
ASV272	Bacteria	Firmicutes	Clostridia	Lachnospirales	Lachnospiraceae	Tuzzerella	TACGTAGGGGGCAAGCGTTATCCGGAATTACTGGGTGTAAAGGGTGAGTAGGCGGCATGGTAAGCCAGATGTGAAAGCCTTGGGCTTAACCCGAGGATTGCATTTGGAACTATCAAGCTAGAGTACAGGAGAGGAAAGCGGAATTCCTAGTGTAGCGGTGAAATGCGTAGATATTAGGAAGAACACCAGTGGCGAAGGCGGCTTTCTGGACTGAAACTGACGCTGAGGCACGAAAGCGTGGGGAGCGAACAGG
ASV087	Bacteria	Firmicutes	Clostridia	Lachnospirales	Lachnospiraceae	ASF356	TACGTAGGGGGCAAGCGTTATCCGGAATTACTGGGTGTAAAGGGAGAGTAGGCGGCAAGGTAAGCGATATGTGAAAGCCTTAGGCTTAACCAAAGGATTGCATAACGAACTATCTAGCTAGAGTACAGGAGAGGAAAGCGGAATTCCTAGTGTAGCGGTGAAATGCGTAGATATTAGGAAGAACACCAGTGGCGAAGGCGGCTTTCTGGACTGAAACTGACGCTGAGGCTCGAAAGCGTGGGGAGCGAACAGG
ASV	Bacteria	Firmicutes	Clostridia	Lachnospirales	Lachnospiraceae	ASF356	TACGTAGGGGGCAAGCGTTATCCGGAATTACTGGGTGTAAAGGGAGAGTAGGCGGCAAGGTAAGCGATATGTGAAAGCCTTAAGCTTAACTTAAGGATGGCATAACGAACTATCTAGCTAGAGTACAGGAGAGGAAAGCGGAATTCCTAGTGTAGCGGTGAAATGCGTAGATATTAGGAAGAACACCAGTGGCGAAGGCGGCTTTCTGGACTGAAACTGACGCTGAGGCTCGAAAGCGTGGGGAGCGAACAGG

111							
ASV129	Bacteria	Firmicutes	Clostridia	Lachnospirales	Lachnospiraceae	Lachnospiraceae_Genus	TACGTAGGGGGCAAGCGTTATCCGGATTTACTGGGTGTAAAGGGAGCGTAGGTGGCATGGTAAGCCAGAAGTGAAAACCCAGGGCTCAACTCTGTGGATTGCTTTTGGAACTATCAAGCTAGAGTGCTGGAGGGGTAAGCGGAATTCCTAGTGTAGCGGTGAAATGCGTAGATATTAGGAGGAACACCGGTGGCGAAGGCGGCTTACTGGACAGAAACTGACACTGAGGCTCGAAAGCGTGGGGAGCAAACAGG
ASV532	Bacteria	Firmicutes	Clostridia	Lachnospirales	Lachnospiraceae	Lachnospiraceae_Genus	TACGTAGGGGGCAAGCGTTATCCGGATTTACTGGGTGTAAAGGGAGCGTAGGTGGCATGGTAAGCCAGAAGTGAAAACCCAGGGCTCAACTCTGTGGATTGCTTTTGGAACTATCAAGCTAGAGTGCCGGAGGGGCAAGCGGAATTCCTAGTGTAGCGGTGAAATGCGTAGATATTAGGAGGAACACCAGTGGCGAAGGCGGTCTGCTGGACAGCAACTGACGGTGAGGCGCGAAAGCGTGGGGAGCAAACAGG
ASV149	Bacteria	Firmicutes	Clostridia	Lachnospirales	Lachnospiraceae	Lachnospiraceae_FCS020_group	TACGTAGGGGGCAAGCGTTATCCGGATTTACTGGGTGTAAAGGGAGCGTAGGTGGCATGGCAAGCCAGAAGTGAAAACCCGGGGCTTAACCCCGCGGATTGCTTTTGGAACTGTCAGGCTGGAGTGCAGGAGGGGCAGGCGGAATTCCTGGTGTAGCGGTGAAATGCGTAGATATCAGGAGGAACACCGGTGGCGAAGGCGGCCTGCTGGACTGTAACTGACACTGAGGCTCGAAAGCGTGGGGAGCAAACAGG
ASV299	Bacteria	Firmicutes	Clostridia	Lachnospirales	Lachnospiraceae	Lachnospiraceae_FCS020_group	TACGTAGGGGGCAAGCGTTATCCGGATTTACTGGGTGTAAAGGGAGCGTAGGTGGCAAGGCAAGCCAGAAGTGAAACCCCGGGGCTTAACCCCGCGGATTGCTTTTGGAACTGTCTGGCTGGAGTGCAGGAGGGGCAGGCGGAATTCCTGGTGTAGCGGTGAAATGCGTAGATATCAGGAGGAACACCGGTGGCGAAGGCGGCCTGCTGGACTGTAACTGACACTGAGGCTCGAAAGCGTGGGGAGCAAACAGG
ASV388	Bacteria	Firmicutes	Clostridia	Lachnospirales	Lachnospiraceae	Anaerostipes	TACGTAGGGGGCAAGCGTTATCCGGAATTACTGGGTGTAAAGGGTGCGTAGGTGGTATGGCAAGTCAGAAGTGAAAACCCAGGGCTTAACTCTGGGACTGCTTTTGAAACTGTCAGACTGGAGTGCAGGAGAGGTAAGCGGAATTCCTAGTGTAGCGGTGAAATGCGTAGATATTAGGAGGAACATCAGTGGCGAAGGCGGCTTACTGGACTGAAACTGACACTGAGGCACGAAAGCGTGGGGAGCAAACAGG
ASV043	Bacteria	Firmicutes	Clostridia	Lachnospirales	Lachnospiraceae	Lachnospiraceae_Genus	TACGTAGGGGGCAAGCGTTATCCGGATTTACTGGGTGTAAAGGGAGCGTAGGTGGCAGGGCAAGTCAGATGTGAAAACCCGGGGCTCAACTCCGGGAGTGCATTTGAAACTGTCCAGCTAGAGTGCAGGAGAGGTAAGCGGAATTCCTAGTGTAGCGGTGAAATGCATAGATATTAGGAGGAACACCAGTGGCGAAGGCGGCTTACTGGACTGTAACTGACACTGAGGCTCGAAAGCGTGGGGAGCAAACAGG
ASV067	Bacteria	Firmicutes	Clostridia	Lachnospirales	Lachnospiraceae	Anaerostipes	TACGTAGGGGGCAAGCGTTATCCGGATTTACTGGGTGTAAAGGGTGCGTAGGTGGCAGGGCAAGTCAGATGTGAAAGCCCGGGGCTCAACCCCGGTACTGCATTTGAAACTGTCCAGCTGGAGTGCAGGAGAGGTAAGCGGAATTCCTAGTGTAGCGGTGAAATGCGTAGATATTAGGAGGAACACCAGTGGCGAAGGCGGCTTACTGGACTGTAACTGACACTGAGGCACGAAAGCGTGGGGAGCAAACAGG
ASV165	Bacteria	Firmicutes	Clostridia	Lachnospirales	Lachnospiraceae	Lachnospiraceae_Genus	TACGTAGGGGGCAAGCGTTATCCGGATTTACTGGGTGTAAAGGGAGCGTAGGTGGCGTGCCAAGTCAGGAGTGAAAACCCAGGGCCCAACCCTGGGACTGCTCTTGAAACTGGCAGGCTTGAGTGCTGGAGAGGTAAGCGGAATTCCTAGTGTAGCGGTGAAATGCGTAGATATTAGGAGGAACACCAGTGGCGAAGGCGGCTTACTGGACAGTGACTGACACTGAGGCTCGAAAGCGTGGGGAGCAAACAGG

ASV284	Bacteria	Firmicutes	Clostridia	Lachnospirales	Lachnospiraceae	Lachnospiraceae_Genus	TACGTAGGGGGCAAGCGTTATCCGGATTTACTGGGTGTAAAGGGAGCGTAGGTGGCAAGGCAAGTCAGAAGTGAAACCCTCGGGCTCAACCTGAGGCATGCTTTTGAAACTGTCAAGCTGGAGTGCTGGAGAGGTAAGCGGAATTCCTAGTGTAGCGGTGAAATGCGTAGATATTAGGAGGAACACCGGTGGCGAAGGCGGCTTACTGGACAGTAACTGACACTGAGGCTCGAAAGCGTGGGGAGCAAACAGG
ASV019	Bacteria	Firmicutes	Clostridia	Lachnospirales	Lachnospiraceae	Lachnospiraceae_Genus	TACGTAGGGGGCAAGCGTTATCCGGATTTACTGGGTGTAAAGGGAGCGTAGACGGCAGGGCAAGCCAGAAGTGAAAACGCAGAGCTTAACTTTGCGACTGCTTTTGGAACTGCCCAGCTAGAGTACAGGAGAGGTAAGTGGAATTCCTAGTGTAGCGGTGAAATGCGTAGATATTAGGAGGAACACCAGTGGCGAAGGCGGCTTACTGGACTGAGACTGACGTTGAGGCTCGAAAGCGTGGGGAGCAAACAGG
ASV246	Bacteria	Firmicutes	Clostridia	Lachnospirales	Lachnospiraceae	GCA-900066575	TACGTAGGGGGCAAGCGTTATCCGGATTTACTGGGTGTAAAGGGAGCGTAGGCGGTAATGCAAGTCAGAAGTGAAAGCCCGGGGCTCAACTCCGGGACTGCTTTTGAAACTGTGTTGCTAGATTGCAGGAGAGGTAAGTGGAATTCCTAGTGTAGCGGTGAAATGCGTAGATATTAGGAGGAACACCAGTGGCGAAGGCGGCTTACTGGACTGTAAATGACGCTGAGGCTCGAAAGCGTGGGGAGCAAACAGG
ASV208	Bacteria	Firmicutes	Clostridia	Lachnospirales	Lachnospiraceae	GCA-900066575	TACGTAGGGGGCAAGCGTTATCCGGATTTACTGGGTGTAAAGGGAGCGTAGGCGGTGATGCAAGTCAGAAGTGAAAGCCCGCAGCTCAACTGCGGGACTGCTTTTGAAACTGTATGACTAGATTGCAGGAGAGGTAAGTGGAATTCCTAGTGTAGCGGTGAAATGCGTAGATATTAGGAGGAACACCAGTGGCGAAGGCGGCTTACTGGACTGTAAATGACGCTGAGGCTCGAAAGCGTGGGGAGCAAACAGG
ASV100	Bacteria	Firmicutes	Clostridia	Lachnospirales	Lachnospiraceae	GCA-900066575	TACGTAGGGGGCAAGCGTTATCCGGATTTACTGGGTGTAAAGGGAGCGTAGGCGGTTCAGCAAGTCAGAAGTGAAAGCCCGGGGCTCAACTCCGGGACTGCTTTTGAAACTGTTGAACTAGATTGCAGGAGAGGTAAGTGGAATTCCTAGTGTAGCGGTGAAATGCGTAGATATTAGGAGGAACACCAGTGGCGAAGGCGGCTTACTGGACTGTAAATGACGCTGAGGCTCGAAAGCGTGGGGAGCAAACAGG
ASV202	Bacteria	Firmicutes	Clostridia	Lachnospirales	Lachnospiraceae	GCA-900066575	TACGTAGGGGGCAAGCGTTATCCGGATTTACTGGGTGTAAAGGGAGCGTAGGCGGTGCAGCAAGTCAGAAGTGAAAGCCCGGGGCTCAACTCCGGGACTGCTTTTGAAACTGTTGAACTAGATTGCAGGAGAGGTAAGTGGAATTCCTAGTGTAGCGGTGAAATGCGTAGATATTAGGAGGAACACCAGTGGCGAAGGCGGCTTACTGGACTGTAAATGACGCTGAGGCTCGAAAGCGTGGGGAGCAAACAGG
ASV107	Bacteria	Firmicutes	Clostridia	Lachnospirales	Lachnospiraceae	GCA-900066575	TACGTAGGGGGCAAGCGTTATCCGGATTTACTGGGTGTAAAGGGAGCGTAGGCGGCGATGCAAGTCAGAAGTGAAAGCCCGGGGCTCAACTCCGGGACTGCTTTTGAAACTGTATGGCTAGATTGCAGGAGAGGCAAGTGGAATTCCTAGTGTAGCGGTGAAATGCGTAGATATTAGGAGGAACACCAGTGGCGAAGGCGGCTTGCTGGACTGTAAATGACGCTGAGGCTCGAAAGCGTGGGGAGCAAACAGG
ASV189	Bacteria	Firmicutes	Clostridia	Lachnospirales	Lachnospiraceae	Lachnospiraceae_FCS020_group	TACGTAGGGGGCAAGCGTTATCCGGATTTACTGGGTGTAAAGGGAGCGTAGACGGTGATGCAAGTCAGAAGTGAAAGCCCGGGGCTCAACTCCGGGACTGCTTTTGAAACTGTGTAACTGGAGTGCAGGAGAGGTAAGCGGAATTCCTGGTGTAGCGGTGAAATGCGTAGATATCAGGAGGAACACCAGTGGCGAAGGCGGCTTACTGGACTGTAACTGACGTTGAGGCTCGAAAGCGTGGGGAGCAAACAGG
ASV	Bacteria	Firmicutes	Clostridia	Lachnospirales	Lachnospiraceae	Lachnospiraceae_FCS020_group	TACGTAGGGGGCAAGCGTTATCCGGATTTACTGGGTGTAAAGGGAGCGTAGACGGTGATGCAAGTCAGAAGTGAAAGCCCGGGGCTCAACTCCGGGACTGCTTTTGAAACTGTGTGACTGGAGTGCAGGAGAGGTAAGCGGAATTCCTGGTGTAGCGGTGAAATGCGTAGATATCAGGAGGAACACCAGTGGCGAAGGCGGCTTACTGGACTGTAACTGACGTTGAGGCTCGAAAGCGTGGGGAGCAAACAGG

021							
ASV148	Bacteria	Firmicutes	Clostridia	Lachnospirales	Lachnospiraceae	Lachnospiraceae_Genus	TACGTAGGGGGCAAGCGTTATCCGGATTTACTGGGTGTAAAGGGAGCGTAGACGGCTGTGCAAGTCAGAAGTGAAAGCCCGGGGCTCAACCGCGGGACTGCTTTTGAAACTGTGTAGCTGGAGTGCAGGAGAGGTAAGTGGAATTCCTAGTGTAGCGGTGAAATGCGTAGATATTAGGAGGAACACCGGTGGCGAAGGCGGCTTACTGGACTGTAACTGACGTTGAGGCTCGAAAGCGTGGGGAGCAAACAGG
ASV199	Bacteria	Firmicutes	Clostridia	Lachnospirales	Lachnospiraceae	Lachnospiraceae_Genus	TACGTAGGGGGCAAGCGTTATCCGGATTTACTGGGTGTAAAGGGAGCGTAGACGGCTGCGCAAGTCAGAAGTGAAAGCCCGGGGCTCAACCGCGGGACTGCTTTTGAAACTGTGTAGCTAGAGTGCAGGAGAGGTAAGTGGAATTCCTAGTGTAGCGGTGAAATGCGTAGATATTAGGAGGAACACCGGTGGCGAAGGCGGCTTACTGGACTGTAACTGACGTTGAGGCTCGAAAGCGTGGGGAGCAAACAGG
ASV345	Bacteria	Firmicutes	Clostridia	Lachnospirales	Lachnospiraceae	Lachnospiraceae_Genus	TACGTAGGGGGCAAGCGTTATCCGGATTTACTGGGTGTAAAGGGAGCGTAGACGGCTGTGCAAGTCAGAAGTGAAAGCCCGGGGCTCAACCGCGGGACTGCTTTTGAAACTGTGTAGCTAGAGTGCAGGAGAGGTAAGTGGAATTCCTAGTGTAGCGGTGAAATGCGTAGATATTAGGAGGAACACCGGTGGCGAAGGCGGCTTACTGGACTGTAACTGACGTTGAGGCTCGAAAGCGTGGGGAGCAAACAGG
ASV378	Bacteria	Firmicutes	Clostridia	Lachnospirales	Lachnospiraceae	Lachnospiraceae_Genus	TACGTAGGGGGCAAGCGTTATCCGGATTTACTGGGTGTAAAGGGAGCGTAGACGGCCAGACAAGTCAGAAGTGAAAACCCGCGGCTCAACTGCGGGAGTGCTTTTGAAACTGTTTGGCTGGAGTGCAGGAGAGGTAAGTGGAATTCCTAGTGTAGCGGTGAAATGCGTAGATATTAGGAGGAACACCAGTGGCGAAGGCGGCTTACTGGACTGTAACTGACGTTGAGGCTCGAAAGCGTGGGGAGCAAACAGG
ASV054	Bacteria	Firmicutes	Clostridia	Lachnospirales	Lachnospiraceae	Eisenbergiella	TACGTAGGGGGCAAGCGTTATCCGGATTTACTGGGTGTAAAGGGAGCGTAGACGGCAATGCAAGCCAGATGTGAAAACCCGCAGCTCAACTGGGGGAGTGCATTTGGAACTGTGTAGCTGGAGTGCAGGAGAGGTAAGCGGAATTCCTGGTGTAGCGGTGAAATGCGTAGATATCAGGAGGAACACCAGTGGCGAAGGCGGCTTACTGGACTGTAACTGACGTTGAGGCTCGAAAGCGTGGGGAGCAAACAGG
ASV075	Bacteria	Firmicutes	Clostridia	Lachnospirales	Lachnospiraceae	Lachnospiraceae_Genus	TACGTAGGGGGCAAGCGTTATCCGGATTTACTGGGTGTAAAGGGAGCGTAGACGGCGATGCAAGCCAGATGTGAAAGCCCGCGGCTCAACTGCGGTAGTGCATTTGGAACTGTATGGCTGGAGTGCAGGAGAGGCAGGCGGAATTCCTAGTGTAGCGGTGAAATGCGTAGATATTAGGAGGAACACCAGTGGCGAAGGCGGCCTGCTGGACTGTAACTGACGTTGAGGCTCGAAAGCGTGGGGAGCAAACAGG
ASV219	Bacteria	Firmicutes	Clostridia	Lachnospirales	Lachnospiraceae	Lachnospiraceae_Genus	TACGTAGGGGGCGAGCGTTATCCGGATTTACTGGGTGTAAAGGGAGCGTAGACGGCTGGTCAAGTCTGAAGTGAAAGCCCGGGGCTTAACCCCGGAACGGCTTTGGAAACTGAGCAGCTAGAGTGCTGGAGAGGTAAGCGGAATTCCTAGTGTAGCGGTGAAATGCGTAGATATTAGGAGGAACACCAGTGGCGAAGGCGGCTTACTGGACAGTAACTGACGTTGAGGCTCGAAAGCGTGGGGAGCAAACAGG
ASV438	Bacteria	Firmicutes	Clostridia	Lachnospirales	Lachnospiraceae	Lachnospiraceae_Genus	TACGTAGGGGGCGAGCGTTATCCGGATTTACTGGGTGTAAAGGGAGCGTAGACGGCTGGTCAAGTCTGAAGTGAAAGCCCGGGGCTTAACCCCGGAACGGTTTTGGAAACTGAGCAGCTAGAGTGCTGGAGAGGTAAGCGGAATTCCTAGTGTAGCGGTGAAATGCGTAGATATTAGGAGGAACACCAGTGGCGGAGGCGGCTTACTGGACAGTAACTGACGTTGAGGCTCGAAAGCGTGGGGAGCAAACAGG

ASV092	Bacteria	Firmicutes	Clostridia	Lachnospirales	Lachnospiraceae	Lachnospiraceae_Genus	TACGTAGGGGGCGAGCGTTATCCGGATTTACTGGGTGTAAAGGGAGCGTAGACGGTTCTGCAAGTCTGAAGTGAAAGCCCGTGGCTTAACCGCGGAACGGCTTTGGAAACTGTGGAACTGGAGTGCTGGAGAGGCAAGCGGAATTCCTAGTGTAGCGGTGAAATGCGTAGATATTAGGAGGAACACCAGTGGCGAAGGCGGCTTGCTGGACAGTAACTGACGTTGAGGCTCGAAAGCGTGGGGAGCAAACAGG
ASV079	Bacteria	Firmicutes	Clostridia	Lachnospirales	Lachnospiraceae	Lachnospiraceae_Genus	TACGTAGGGGGCAAGCGTTATCCGGATTTACTGGGTGTAAAGGGAGCGTAGACGGTTGTGCAAGTCTGAAGTGAAAGCCCGCGGCTCAACTGCGGAACGGCTTTGGAAACTGTGTAACTGGAGTGCTGGAGAGGTAAGCGGAATTCCTGGTGTAGCGGTGAAATGCGTAGAGATCAGGAGGAACACCGGCGGCGAAGGCGGCTTACTGGACAGTAACTGACGTTGAGGCTCGAAAGCGTGGGGAGCAAACAGG
ASV120	Bacteria	Firmicutes	Clostridia	Lachnospirales	Lachnospiraceae	Lachnospiraceae_Genus	TACGTAGGGGGCGAGCGTTATCCGGATTTACTGGGTGTAAAGGGAGCGTAGACGGCGTCGCAAGTCTGAAGTGAAAGCCCGTGGCTCAACCGCGGAACCGCTTTGGAAACTGCGAGGCTGGAGTGCTGGAGAGGTAAGCGGAATTCCTGGTGTAGCGGTGAAATGCGTAGATATCAGGAGGAACACCGGTGGCGAAGGCGGCTTACTGGACAGTGACTGACGTTGAGGCTCGAAAGCGTGGGGAGCGAACAGG
ASV342	Bacteria	Firmicutes	Clostridia	Lachnospirales	Lachnospiraceae	Lachnospiraceae_Genus	TACGTAGGGGGCGAGCGTTATCCGGATTTACTGGGTGTAAAGGGAGCGTAGACGGCGTCGCAAGTCTGAAGTGAAAGCCCGTGGCTCAACTGCGGAACCGCTTTGGAAACTGCGAGGCTGGAGTGCTGGAGAGGTAAGCGGAATTCCTGGTGTAGCGGTGAAATGCGTAGATATCAGGAGGAACACCGGTGGCGAAGGCGGCTTACTGGACAGTGACTGACGTTGAGGCTCGAAAGCGTGGGGAGCGAACAGG
ASV108	Bacteria	Firmicutes	Clostridia	Lachnospirales	Lachnospiraceae	Lachnospiraceae_Genus	TACGTAGGGGGCAAGCGTTATCCGGATTTACTGGGTGTAAAGGGAGCGTAGACGGCTGTGCAAGTCTGAAGTGAAAGCCCGTGGCTCAACCGCGGAACTGCTTTGGAAACTGTATAGCTTGAGTACTGGAGAGGCAGGCGGAATTCCTAGTGTAGCGGTGAAATGCGTAGATATTAGGAGGAACACCAGTGGCGAAGGCGGCCTGCTGGACAGAAACTGACGTTGAGGCTCGAAAGCGTGGGGAGCAAACAGG
ASV118	Bacteria	Firmicutes	Clostridia	Lachnospirales	Lachnospiraceae	Lachnospiraceae_Genus	TACGTAGGGGGCAAGCGTTATCCGGATTTACTGGGTGTAAAGGGAGCGTAGACGGCTGTACAAGTCTGAAGTGAAAGCCCGTGGCTCAACCGCGGAACTGCTTTGGAAACTGTAAAGCTGGAGTACTGGAGAGGCAGGCGGAATTCCTAGTGTAGCGGTGAAATGCGTAGATATTAGGAGGAACACCAGTGGCGAAGGCGGCCTGCTGGACAGAAACTGACGTTGAGGCTCGAAAGCGTGGGGAGCAAACAGG
ASV105	Bacteria	Firmicutes	Clostridia	Lachnospirales	Lachnospiraceae	Lachnospiraceae_Genus	TACGTAGGGGGCAAGCGTTATCCGGATTTACTGGGTGTAAAGGGAGCGCAGACGGCAGTGCAAGTCTGGAGTGAAAGCCCGGGGCCCAACCCCGGAACTGCTCTGGAAACTGTGCGGCTAGAGTACTGGAGGGGCAGGCGGAATTCCTAGTGTAGCGGTGAAATGCGTAGATATTAGGAGGAACACCAGTGGCGAAGGCGGCCTGCTGGACAGTAACTGACGTTGAGGCTCGAAAGCGTGGGGAGCAAACAGG
ASV319	Bacteria	Firmicutes	Clostridia	Lachnospirales	Lachnospiraceae	Lachnospiraceae_Genus	TACGTAGGGGGCAAGCGTTATCCGGATTTACTGGGTGTAAAGGGAGCGCAGACGGCAGCGCAAGTCTGGAGTGAAAGCCCGGGGCCCAACCCCGGAACTGCTCTGGAAACTGTGCGGCTAGAGTACTGGAGGGGCAGGCGGAATTCCTAGTGTAGCGGTGAAATGCGTAGATATTAGGAGGAACACCAGTGGCGAAGGCGGCCTGCTGGACAGTAACTGACGTTGAGGCTCGAAAGCGTGGGGAGCAAACAGG
ASV	Bacteria	Firmicutes	Clostridia	Lachnospirales	Lachnospiraceae	Acetatifactor	TACGTAGGGGGCAAGCGTTATCCGGATTTACTGGGTGTAAAGGGAGCGTAGACGGCGCTGCAAGTCCGGAGTGAAAGCCCATGGCTTAACCATGGTACTGCTCTGGAAACTGTGGAGCTTGAGTGCGGGAGGGGCAGGCGGAATTCCTAGTGTAGCGGTGAAATGCGTAGATATTAGGAGGAACACCGGCGGCGAAGGCGGCCTGCTGGACCGTAACTGACGTTGAGGCTCGAAAGCGTGGGGAGCAAACAGG

062							
ASV076	Bacteria	Firmicutes	Clostridia	Lachnospirales	Lachnospiraceae	Acetatifactor	TACGTAGGGGGCAAGCGTTATCCGGATTTACTGGGTGTAAAGGGAGCGTAGACGGCAGCGCAAGTCCGAAGTGAAAGCCCGGGGCCCAACCCCGGGACTGCTTTGGAAACTGTGAAGCTGGAGTGCGGGAGGGGCAGGCGGAATTCCTGGTGTAGCGGTGAAATGCGTAGATATCAGGAGGAACACCGGCGGCGAAGGCGGCCTGCTGGACCGTAACTGACGTTGAGGCTCGAAAGCGTGGGGAGCAAACAGG
ASV363	Bacteria	Firmicutes	Clostridia	Lachnospirales	Lachnospiraceae	Acetatifactor	TACGTAGGGGGCAAGCGTTATCCGGATTTACTGGGTGTAAAGGGAGCGTAGACGGCAGCGCAAGTCCGAAGTGAAAGCCCGGGGCCCAACCCCGGGACTGCTTTGGAAACTGTGTAACTGGAGTACAGGAGGGGCAGGCGGAATTCCTGGTGTAGCGGTGAAATGCGTAGATATCAGGAGGAACACCGGCGGCGAAGGCGGCCTGCTGGACTGAAACTGACGTTGAGGCTCGAAAGCGTGGGGAGCAAACAGG
ASV026	Bacteria	Firmicutes	Clostridia	Lachnospirales	Lachnospiraceae	Lachnospiraceae_Genus	TACGTAGGGGGCAAGCGTTATCCGGATTTACTGGGTGTAAAGGGAGCGTAGACGGTTATGTAAGTCTGGAGTGAAAGCCCGGGGCCCAACCCCGGGACTGCTTTGGAAACTGTGTAACTGGAGTACAGGAGGGGCAGGCGGAATTCCTGGTGTAGCGGTGAAATGCGTAGATATCAGGAGGAACACCGGCGGCGAAGGCGGCCTGCTGGACTGAAACTGACGTTGAGGCTCGAAAGCGTGGGGAGCAAACAGG
ASV384	Bacteria	Firmicutes	Clostridia	Lachnospirales	Lachnospiraceae	Acetatifactor	TACGTAGGGGGCAAGCGTTATCCGGATTTACTGGGTGTAAAGGGAGCGTAGACGGTTGTATAAGTCTGGAGTGAAAGCCCGGGGCCCAACCCCGGGACTGCTTTGGAAACTGTGTAACTTGAGTACAGGAGGGGCAGGCGGAATTCCTGGTGTAGCGGTGAAATGCGTAGATATCAGGAGGAACACCGGCGGCGAAGGCGGCCTGCTGGACTGAAACTGACGTTGAGGCTCGAAAGCGTGGGGAGCAAACAGG
ASV096	Bacteria	Firmicutes	Clostridia	Lachnospirales	Lachnospiraceae	Acetatifactor	TACGTAGGGGGCAAGCGTTATCCGGATTTACTGGGTGTAAAGGGAGCGTAGACGGCAGAGCAAGTCCGGAGTGAAAGCCCGGGGCCCAACCCCGGGACTGCTCTGGAAACTGTTAAGCTGGAGTGCGGGAGGGGCAGGCGGAATTCCTGGTGTAGCGGTGAAATGCGTAGATATCAGGAGGAACACCGGCGGCGAAGGCGGCCTGCTGGACCGTGACTGACGTTGAGGCTCGAAAGCGTGGGGAGCAAACAGG
ASV242	Bacteria	Firmicutes	Clostridia	Lachnospirales	Lachnospiraceae	Acetatifactor	TACGTAGGGGGCAAGCGTTATCCGGATTTACTGGGTGTAAAGGGAGCGTAGACGGCAGAGCAAGTCCGGAGTGAAAGCCCGGGGCCCAACCCCGGGACTGCTCTGGAAACTGTTAAGCTGGAGTACGGGAGGGGCAGGCGGAATTCCTGGTGTAGCGGTGAAATGCGTAGATATCAGGAGGAACACCGGCGGCGAAGGCGGCCTGCTGGACCGTGACTGACGTTGAGGCTCGAAAGCGTGGGGAGCAAACAGG
ASV409	Bacteria	Firmicutes	Clostridia	Lachnospirales	Lachnospiraceae	Lachnospiraceae_Genus	TACGTAGGGGGCAAGCGTTATCCGGATTCACTGGGTGTAAAGGGAGCGTAGACGGCCATGCAAGCCTGGAGTGAAAGCCCGGGGCCCAACCCCGGGACTGCTCTGGGAACTGTGAAGCTGGAGTGCGGGAGGGGCAGGCGGAATTCCTGGTGTAGCGGTGAAATGCGTAGATATTAGGAGGAACACCAGTGGCGAAGGCGGCTTACTGGACTGTAACTGACGTTGAGGCTCGAAAGCGTGGGGAGCAAACAGG
ASV056	Bacteria	Firmicutes	Clostridia	Lachnospirales	Lachnospiraceae	Acetatifactor	TACGTAGGGGGCAAGCGTTATCCGGATTCACTGGGTGTAAAGGGAGCGTAGACGGCCATGCAAGCCTGGAGTGAAAGCCCGGGGCCCAACCCCGGGACTGCTCTGGGAACTGTGCGGCTGGAGTGCGGGAGGGGCAGGCGGAATTCCTGGTGTAGCGGTGAAATGCGTAGATATCAGGAGGAACACCGGCGGCGAAGGCGGCCTGCTGGACCGCGACTGACGTTGAGGCTCGAAAGCGTGGGGAGCAAACAGG

ASV257	Bacteria	Firmicutes	Clostridia	Lachnospirales	Lachnospiraceae	Acetatifactor	TACGTAGGGGGCGAGCGTTATCCGGATTCACTGGGTGTAAAGGGAGCGTAGACGGCCGTGCAAGCCAGGAGTGAAAGCCCGGGGCCCAACCCCGGGACTGCTCTTGGAACTGTGCGGCTGGAGTGCGGGAGGGGCAGGCGGAATTCCTGGTGTAGCGGTGAAATGCGTAGATATCAGGAGGAACACCGGCGGCGAAGGCGGCCTGCTGGACCGCGACTGACGTTGAGGCTCGAAAGCGTGGGGAGCGAACAGG
ASV335	Bacteria	Firmicutes	Clostridia	Lachnospirales	Lachnospiraceae	Acetatifactor	TACGTAGGGGGCGAGCGTTATCCGGATTCACTGGGTGTAAAGGGAGCGTAGACGGCCGTGCAAGCCAGGAGTGAAAGCCCGGGGCCCAACCCCGGGACTGCTCTTGGAACTGTGCGACTGGAGTGCGGGAGGGGCAGGCGGAATTCCTGGTGTAGCGGTGAAATGCGTAGATATCAGGAGGAACACCGGCGGCGAAGGCGGCCTGCTGGACCGCGACTGACGTTGAGGCTCGAAAGCGTGGGGAGCGAACAGG
ASV235	Bacteria	Firmicutes	Clostridia	Lachnospirales	Lachnospiraceae	Acetatifactor	TACGTAGGGGGCGAGCGTTATCCGGATTCACTGGGTGTAAAGGGAGCGTAGACGGCCATGCAAGCCAGGGGTGAAAGCCCGGGGCCCAACCCCGGGACTGCCCTTGGAACTGTGCGGCTAGAGTGCGGGAGGGGCAGGCGGAATTCCTGGTGTAGCGGTGAAATGCGTAGATATCAGGAGGAACACCGGCGGCGAAGGCGGCCTGCTGGACCGCGACTGACGTTGAGGCTCGAAAGCGTGGGGAGCGAACAGG
ASV102	Bacteria	Firmicutes	Clostridia	Lachnospirales	Lachnospiraceae	Acetatifactor	TACGTAGGGGGCGAGCGTTATCCGGATTCACTGGGTGTAAAGGGAGCGTAGACGGCCATGCAAGCCAGGGGTGAAAGCCCGGGGCCCAACCCCGGGACTGCCCTTGGAACTGCATGGCTGGAGTGCGGGAGGGGCAGGCGGAATTCCTGGTGTAGCGGTGAAATGCGTAGATATCAGGAGGAACACCGGCGGCGAAGGCGGCCTGCTGGACCGCGACTGACGTTGAGGCTCGAAAGCGTGGGGAGCGAACAGG
ASV232	Bacteria	Firmicutes	Clostridia	Lachnospirales	Lachnospiraceae	Acetatifactor	TACGTAGGGGGCGAGCGTTATCCGGATTCACTGGGTGTAAAGGGAGCGTAGACGGCCAAGCAAGCCAGGGGTGAAAGCCCGGGGCCCAACCCCGGGACTGCCCTTGGAACTGCATGGCTGGAGTGCGGGAGGGGCAGGCGGAATTCCTGGTGTAGCGGTGAAATGCGTAGATATCAGGAGGAACACCGGCGGCGAAGGCGGCCTGCTGGACCGCGACTGACGTTGAGGCTCGAAAGCGTGGGGAGCGAACAGG
ASV304	Bacteria	Firmicutes	Clostridia	Lachnospirales	Lachnospiraceae	Acetatifactor	TACGTAGGGGGCAAGCGTTATCCGGATTCACTGGGTGTAAAGGGAGCGTAGACGGCCAGGCAAGCCTGGAGTGAAAGCCCGGGGCCCAACCCCGGGACTGCTCTGGGAACTGTCCGGCTGGAGTGCGGGAGGGGCAGGCGGAATTCCTGGTGTAGCGGTGAAATGCGTAGATATCAGGAGGAACACCGGCGGCGAAGGCGGCCTGCTGGACCGCGACTGACGTTGAGGCTCGAAAGCGTGGGGAGCGAACAGG
ASV375	Bacteria	Firmicutes	Clostridia	Lachnospirales	Lachnospiraceae	Lachnospiraceae_Genus	TACGTAGGGGGCAAGCGTTATCCGGATTTACTGGGTGTAAAGGGGGCGCAGACGGCAGCGCAAGCCAGGAGTGAAAGCCCGGGGCCCAACCCCGGGACTGCTCTTGGAACTGCGCGGCTGGAGTGCAGGAGGGGCAGGCGGAATTCCTGGTGTAGCGGTGAAATGCGTAGATATCAGGAGGAACACCGGTGGCGAAGGCGGCCTGCTGGACTGCGACTGACGTTGAGGCCCGAAAGCGTGGGGAGCAAACAGG
ASV0722	Bacteria	Firmicutes	Clostridia	Lachnospirales	Lachnospiraceae	Lachnospiraceae_Genus	TACGTAGGGGGCAAGCGTTATCCGGATTTACTGGGTGTAAAGGGGGCGCAGACGGCGATGCAAGCCAGGAGTGAAAGCCCGGGGCCCAACCCCGGGACTGCTCTTGGAACTGCGTGGCTGGAGTGCAGGAGGGGCAGGCGGAATTCCTGGTGTAGCGGTGAAATGCGTAGATATCAGGAGGAACACCGGTGGCGAAGGCGGCCTGCTGGACTGCAACTGACGTTGAGGCCCGAAAGCGTGGGGAGCAAACAGG
ASV	Bacteria	Firmicutes	Clostridia	Lachnospirales	Lachnospiraceae	Lachnospiraceae_Genus	TACGTAGGGGGCAAGCGTTATCCGGATTTACTGGGTGTAAAGGGGGCGCAGACGGCAATGCAAGCCAGGAGTGAAAGCCCGGGGCCCAACCCCGGGACTGCTCTTGGAACTGCATGGCTGGAGTACAGGAGGGGCAGGCGGAATTCCTAGTGTAGCGGTGAAATGCGTAGATATTAGGAGGAACACCGGTGGCGAAGGCGGCCTGCTGGACTGTGACTGACGTTGAGGCCCGAAGGCGTGGGGAGCAAACAGG

112							
ASV296	Bacteria	Firmicutes	Clostridia	Lachnospirales	Lachnospiraceae	Lachnospiraceae_Genus	TACGTAGGGGGCAAGCGTTATCCGGATTTACTGGGTATAAAGGGGGCGCAGACGGCAATGCAAGCCAGGAGTGAAAGCCCGGGGCCCAACCCCGGGACTGCTCTTGGAACTGCATGGCTGGAGTACAGGAGGGGCAGGCGGAATTCCTAGTGTAGCGGTGAAATGCGTAGATATTAGGAGGAACACCGGTGGCGAAGGCGGCCTGCTGGACTGTGACTGACGTTGAGGCCCGAAGGCGTGGGGAGCAAACAGG
ASV178	Bacteria	Firmicutes	Clostridia	Lachnospirales	Lachnospiraceae	Lachnoclostridium	TACGTAGGGGGCAAGCGTTATCCGGATTTACTGGGTGTAAAGGGAGCGTAGACGGCTGCGCAAGTCTGAAGTGAAAGCCCGGGGCTCAACCCCGGGACTGCTTTGGAAACTGTGTAGCTAGAGTGCTGGAGAGGCAAGCGGAATTCCTAGTGTAGCGGTGAAATGCGTAGATATTAGGAAGAACACCAGTGGCGAAGGCGGCTTGCTGGACAGTAACTGACGTTGAGGCTCGAAAGCGTGGGGAGCAAACAGG
ASV086	Bacteria	Firmicutes	Clostridia	Lachnospirales	Lachnospiraceae	Lachnospiraceae_Genus	TACGTAGGGGGCAAGCGTTATCCGGATTTACTGGGTGTAAAGGGAGCGTAGACGGTGAGACAAGTCTGAAGTGAAAGCCCGGGGCTCAACCCCGGGACTGCTTTGGAAACTGCCTGACTGGAGTGCTGGAGAGGCAAGCGGAATTCCTAGTGTAGCGGTGAAATGCGTAGATATTAGGAAGAACACCAGTGGCGAAGGCGGCTTGCTGGACAGTAACTGACGTTGAGGCTCGAAAGCGTGGGGAGCAAACAGG
ASV140	Bacteria	Firmicutes	Clostridia	Lachnospirales	Lachnospiraceae	Lachnoclostridium	TACGTAGGGGGCAAGCGTTATCCGGATTTACTGGGTGTAAAGGGAGCGTAGACGGCGATGCAAGTCTGAAGTGAAAGCCCGGGGCTCAACCCCGGGACTGCTTTGGAAACTGTATGGCTGGAGTGCTGGAGAGGCAAGCGGAATTCCTAGTGTAGCGGTGAAATGCGTAGATATTAGGAAGAACACCAGTGGCGAAGGCGGCTTGCTGGACAGTAACTGACGTTGAGGCTCGAAAGCGTGGGGAGCAAACAGG
ASV358	Bacteria	Firmicutes	Clostridia	Lachnospirales	Lachnospiraceae	Lachnospiraceae_UCG-002	TACGTAGGGGGCAAGCGTTATCCGGATTTACTGGGTGTAAAGGGAGCGTAGACGGCAGAGAAAGTCTGAAGTGAAAGCCCGCGGCTCAACCGCGGAACTGCTTTGGAAACTTTTCGGCTAGAGTACCGGAGAGGTAAGCGGAATTCCTAGTGTAGCGGTGAAATGCGTAGATATTAGGAGGAACACCAGTGGCGAAGGCGGCTTACTGGACGGTAACTGACGTTGAGGCTCGAAAGCGTGGGGAGCAAACAGG
ASV130	Bacteria	Firmicutes	Clostridia	Lachnospirales	Lachnospiraceae	Lachnospiraceae_Genus	TACGTAGGGGGCAAGCGTTATCCGGATTTACTGGGTGTAAAGGGAGCGTAGACGGCGAAGAAAGTCTGAAGTGAAAGCCCGCGGCTCAACCGCGGAACTGCTTTGGAAACTTTTTTGCTGGAGTACCGGAGAGGTAAGCGGAATTCCTAGTGTAGCGGTGAAATGCGTAGATATTAGGAGGAACACCAGTGGCGAAGGCGGCTTACTGGACGGTAACTGACGTTGAGGCTCGAAAGCGTGGGGAGCAAACAGG
ASV303	Bacteria	Firmicutes	Clostridia	Lachnospirales	Lachnospiraceae	Lachnospiraceae_Genus	TACGTAGGGGGCAAGCGTTATCCGGATTTACTGGGTGTAAAGGGAGCGTAGACGGCGAAGAAAGTCTGAAGTGAAAGCCCGCGGCTTAACCGCGGAACTGCTTTGGAAACTTTTTTGCTGGAGTACCGGAGAGGTAAGCGGAATTCCTAGTGTAGCGGTGAAATGCGTAGATATTAGGAGGAACACCAGTGGCGAAGGCGGCTTACTGGACGGTAACTGACGTTGAGGCTCGAAAGCGTGGGGAGCAAACAGG
ASV071	Bacteria	Firmicutes	Clostridia	Lachnospirales	Lachnospiraceae	Lachnospiraceae_Genus	TACGTAGGGGGCAAGCGTTATCCGGATTTACTGGGTGTAAAGGGAGCGTAGACGGCAGAGCAAGTCTGAAGTGAAAGCCCGCGGCCCAACTGCGGGACTGCTTTGGAAACTGCCCGGCTAGAGTGCTGGAGAGGTAAGCGGAATTCCTAGTGTAGCGGTGAAATGCGTAGATATTAGGAGGAACACCAGTGGCGAAGGCGGCTTACTGGACAGTAACTGACGTTGAGGCTCGAAAGCGTGGGGAGCAAACAGG

ASV2244	Bacteria	Firmicutes	Clostridia	Lachnospirales	Lachnospiraceae	Lachnoclostridium	TACGTAGGGGGCAAGCGTTATCCGGATTTACTGGGTGTAAAGGGAGCGTAGACGGTAGAGCAAGTCTGAAGTGAAAGCCCGCGGCTCAACTGCGGGACTGCTTTGGAAACTGTTCAACTGGAGTGCTGGAGAGGTAAGCGGAATTCCTAGTGTAGCGGTGAAATGCGTAGATATTAGGAGGAACACCAGTGGCGAAGGCGGCTTACTGGACAGTAACTGACGTTGAGGCTCGAAAGCGTGGGGAGCAAACAGG
ASV2188	Bacteria	Firmicutes	Clostridia	Lachnospirales	Lachnospiraceae	Lachnoclostridium	TACGTAGGGGGCAAGCGTTATCCGGATTTACTGGGTGTAAAGGGAGCGTAGACGGCAGGGCAAGTCTGGAGTGAAAGCCCGGGGCCCAACCCCGGGACTGCTTTGGAAACTGCCATGCTGGAGTGCTGGAGAGGTAAGCGGAATTCCTAGTGTAGCGGTGAAATGCGTAGATATTAGGAGGAACACCAGTGGCGAAGGCGGCTTACTGGACAGTAACTGACGTTGAGGCTCGAAAGCGTGGGGAGCAAACAGG
ASV0422	Bacteria	Firmicutes	Clostridia	Lachnospirales	Lachnospiraceae	Lachnospiraceae_Genus	TACGTAGGGGGCAAGCGTTATCCGGATTTACTGGGTGTAAAGGGAGCGTAGACGGCGCGGCAAGTCTGAAGTGAAAGGCAGGGGCTTAACCCCTGAACTGCTTTGGAAACTGCCATGCTAGAGTGCTGGAGAGGTAAGTGGAATTCCTAGTGTAGCGGTGAAATGCGTAGATATTAGGAGGAACACCAGTGGCGAAGGCGGCTTACTGGACAGTAACTGACGTTGAGGCTCGAAAGCGTGGGGAGCAAACAGG
ASV1611	Bacteria	Firmicutes	Clostridia	Lachnospirales	Lachnospiraceae	Lachnospiraceae_Genus	TACGTAGGGGGCAAGCGTTATCCGGATTTACTGGGTGTAAAGGGAGCGTAGACGGTGAGGCAAGTCTGAAGTGAAAGCCCGGGGCTTAACCGCGGGACTGCTTTGGAAACTGTCTGACTGGAGTGCTGGAGAGGTAAGTGGAATTCCTAGTGTAGCGGTGAAATGCGTAGATATTAGGAGGAACACCAGTGGCGAAGGCGGCTTACTGGACAGTAACTGACGTTGAGGCTCGAAAGCGTGGGGAGCAAACAGG
ASV1944	Bacteria	Firmicutes	Clostridia	Lachnospirales	Lachnospiraceae	Lachnospiraceae_Genus	TACGTAGGGGGCAAGCGTTATCCGGATTTACTGGGTGTAAAGGGAGCGTAGACGGTGAGGCAAGTCTGAAGTGAAAGCCCGGGGCTTAACCCCGGGACTGCTTTGGAAACTGTCTGACTGGAGTGCTGGAGAGGTAAGTGGAATTCCTAGTGTAGCGGTGAAATGCGTAGATATTAGGAGGAACACCAGTGGCGAAGGCGGCTTACTGGACAGTAACTGACGTTGAGGCTCGAAAGCGTGGGGAGCAAACAGG
ASV1455	Bacteria	Firmicutes	Clostridia	Lachnospirales	Lachnospiraceae	Lachnospiraceae_Genus	TACGTAGGGGGCAAGCGTTATCCGGATTTACTGGGTGTAAAGGGAGCGTAGACGGTGCGTCAAGTCTGAAGTGAAAGCCCGGGGCTTAACCCCGGGACTGCTTTGGAAACTGGCGCGCTAGAGTGCTGGAGAGGTAAGTGGAATTCCTAGTGTAGCGGTGAAATGCGTAGATATTAGGAGGAACACCAGTGGCGAAGGCGGCTTACTGGACAGTAACTGACGTTGAGGCTCGAAAGCGTGGGGAGCAAACAGG
ASV3911	Bacteria	Firmicutes	Clostridia	Lachnospirales	Lachnospiraceae	Fusicatenibacter	TACGTAGGGGGCAAGCGTTATCCGGATTTACTGGGTGTAAAGGGAGCGTAGACGGCAAGGCAAGTCTGATGTGAAAACCCAGGGCTTAACCCTGGGACTGCATTGGAAACTGTCTGGCTCGAGTGCCGGAGAGGTAAGCGGAATTCCTAGTGTAGCGGTGAAATGCGTAGATATTAGGAAGAACACCAGTGGCGAAGGCGGCTTACTGGACGGTAACTGACGTTGAGGCTCGAAAGCGTGGGGAGCAAACAGG
ASV0444	Bacteria	Firmicutes	Clostridia	Lachnospirales	Lachnospiraceae	Lachnospiraceae_NK4A136_group	TACGTAGGGGGCAAGCGTTATCCGGATTTACTGGGTGTAAAGGGAGCGTAGACGGCCAGACAAGTCTGAAGTGAAAATCCAGCGCTTAACGTTGGAAGTGCTTTGGAAACTGCCGGGCTAGAGTGCAGGAGGGGCAGGCGGAATTCCTAGTGTAGCGGTGAAATGCGTAGATATTAGGGGGAACACCAGTGGCGAAGGCGGCCTGCTGGACTGCAACTGACGTTGAGGCTCGAAGGCGTGGGGAGCAAACAGG
ASV	Bacteria	Firmicutes	Clostridia	Lachnospirales	Lachnospiraceae	Lachnospiraceae_NK4A136_group	TACGTAGGGGGCAAGCGTTATCCGGATTTACTGGGTGTAAAGGGAGCGTAGACGGCCAGACAAGTCTGAAGTGAAAATCCAGCGCTTAACGTTGGAAGTGCTTTGGAAACTGCCGGGCTAGAGTGCAGGAGGGGCAGGCGGAATTCCTAGTGTAGCGGTGAAATGCGTAGATATTAGGAGGAACACCAGTGGCGAAGGCGGCCTGCTGGACTGCAACTGACGTTGAGGCTCGAAGGCGTGGGGAGCAAACAGG

013							
ASV310	Bacteria	Firmicutes	Clostridia	Lachnospirales	Lachnospiraceae	Lachnospiraceae_Genus	TACGTAGGGGGCAAGCGTTATCCGGATTTACTGGGTGTAAAGGGAGCGTAGACGGCTAGTCAAGTCAGAAGTGAAAGCCCAGGGCTCAACCTTGGGACTGCTTTTGAAACTGAGTAGCTAGAGTGCAGGAGAGGTAAGCGGAATTCCTAGTGTAGCGGTGAAATGCGTAGATATTAGGAGGAACACCAGTGGCGAAGGCGGCTTACTGGACTGTAACTGACGTTGAGGCTCGAAAGCGTGGGGAGCAAACAGG
ASV220	Bacteria	Firmicutes	Clostridia	Lachnospirales	Lachnospiraceae	Lachnospiraceae_Genus	TACGTAGGGGGCAAGCGTTATCCGGATTTACTGGGTGTAAAGGGAGCGTAGACGGCTAATCAAGTCAGAAGTGAAAACCCAGGGCTCAACTTTGGGACTGCTTTTGAAACTGAGTAGCTAGAGTGCAGGAGAGGTAAGCGGAATTCCTAGTGTAGCGGTGAAATGCGTAGATATTAGGAGGAACACCAGTGGCGAAGGCGGCTTACTGGACTGTAACTGACGTTGAGGCTCGAAAGCGTGGGGAGCAAACAGG
ASV158	Bacteria	Firmicutes	Clostridia	Lachnospirales	Lachnospiraceae	Lachnospiraceae_Genus	TACGTAGGGGGCAAGCGTTATCCGGATTTACTGGGTGTAAAGGGAGCGTAGACGGTTAATCAAGTCAGAAGTGAAAACCCAGGGCTCAACCTTGGGACTGCTTTTGAAACTGAGTAGCTAGAGTGCAGGAGAGGTAAGCGGAATTCCTAGTGTAGCGGTGAAATGCGTAGATATTAGGAGGAACACCAGTGGCGAAGGCGGCTTACTGGACTGTAACTGACGTTGAGGCTCGAAAGCGTGGGGAGCAAACAGG
ASV488	Bacteria	Firmicutes	Clostridia	Lachnospirales	Lachnospiraceae	Lachnospiraceae_Genus	TACGGAGGATGCGAGCGTTATCCGGATTTACTGGGTGTAAAGGGAGCGTAGACGGTTAATCAAGTCAGAAGTGAAAACCCAGGGCTCAACCTTGGGAGTGCTTTTGAAACTGAGTAGCTAGAGTGCAGGAGAGGTAAGCGGAATTCCTAGTGTAGCGGTGAAATGCGTAGATATTAGGAGGAACACCAGTGGCGAAGGCGGCTTCCTGGTCTGCAACTGACACTGAGGCGCGAAAGCGTGGGGAGCAAACAGG
ASV311	Bacteria	Firmicutes	Clostridia	Lachnospirales	Lachnospiraceae	Lachnospiraceae_Genus	TACGTAGGGGGCAAGCGTTATCCGGATTTACTGGGTGTAAAGGGAGCGTAGACGGTTAATCAAGTCAGAAGTGAAAACCCAGGGCTCAACCTTGGGAGTGCTTTTGAAACTGAGTAGCTAGAGTGCAGGAGAGGTAAGCGGAATTCCTAGTGTAGCGGTGAAATGCGTAGATATTAGGAGGAACACCAGTGGCGAAGGCGGCTTACTGGACTGTAACTGACGTTGAGGCTCGAAAGCGTGGGGAGCAAACAGG
ASV256	Bacteria	Firmicutes	Clostridia	Lachnospirales	Lachnospiraceae	Lachnospiraceae_Genus	TACGTAGGGGGCAAGCGTTATCCGGATTTACTGGGTGTAAAGGGAGCGTAGACGGCTAATCAAGTCAGAAGTGAAAACCCAGGGCTCAACCTTGGGACTGCTTTTGAAACTGAGTAGCTAGAGTGCAGGAGAGGTAAGCGGAATTCCTAGTGTAGCGGTGAAATGCGTAGATATTAGGAGGAACACCAGTGGCGAAGGCGGCTTACTGGACTGTAACTGACGTTGAGGCTCGAAAGCGTGGGGAGCAAACAGG
ASV276	Bacteria	Firmicutes	Clostridia	Lachnospirales	Lachnospiraceae	Lachnospiraceae_Genus	TACGTAGGGGGCAAGCGTTATCCGGATTTACTGGGTGTAAAGGGAGCGTAGACGGCCAGGCAAGTCAGAAGTGAAAGCCCAGGGCTCAACCCTGGGACTGCTTTTGAAACTGCCGGGCTGGAGTGCAGGAGAGGTAAGCGGAATTCCTAGTGTAGCGGTGAAATGCGTAGATATTAGGAGGAACACCAGTGGCGAAGGCGGCTTACTGGACTGTAACTGACGTTGAGGCTCGAAAGCGTGGGGAGCAAACAGG
ASV280	Bacteria	Firmicutes	Clostridia	Lachnospirales	Lachnospiraceae	Lachnospiraceae_Genus	TACGTAGGGGGCAAGCGTTATCCGGATTTACTGGGTGTAAAGGGAGCGTAGACGGCCTGACAAGTCAGAAGTGAAAGCCCAGGGCTCAACCCTGGGACTGCTTTTGAAACTGCCGGGCTGGAGTGCAGGAGAGGTAAGCGGAATTCCTAGTGTAGCGGTGAAATGCGTAGATATTAGGAGGAACACCAGTGGCGAAGGCGGCTTACTGGACTGTAACTGACGTTGAGGCTCGAAAGCGTGGGGAGCAAACAGG

ASV063	Bacteria	Firmicutes	Clostridia	Lachnospirales	Lachnospiraceae	Lachnospiraceae_Genus	TACGTAGGGGGCAAGCGTTATCCGGATTTACTGGGTGTAAAGGGAGCGTAGACGGCCAGACAAGTCAGAAGTGAAAGCCCAGGGCTCAACCCTGGGACTGCTTTTGAAACTGCCGGGCTGGAGTGCAGGAGAGGTAAGCGGAATTCCTAGTGTAGCGGTGAAATGCGTAGATATTAGGAGGAACACCAGTGGCGAAGGCGGCTTACTGGACTGTAACTGACGTTGAGGCTCGAAAGCGTGGGGAGCAAACAGG
ASV333	Bacteria	Firmicutes	Clostridia	Lachnospirales	Lachnospiraceae	Lachnospiraceae_Genus	TACGTAGGGGGCAAGCGTTATCCGGATTTACTGGGTGTAAAGGGAGCGTAGACGGCCAGACAAGTCAGAAGTGAAAGCCCAGGGCTCAACTCTGGGACTGCTTTTGAAACTGCCGGGCTGGAGTGCAGGAGAGGTAAGCGGAATTCCTAGTGTAGCGGTGAAATGCGTAGATATTAGGAGGAACACCAGTGGCGAAGGCGGCTTACTGGACTGTAACTGACGTTGAGGCTCGAAAGCGTGGGGAGCAAACAGG
ASV095	Bacteria	Firmicutes	Clostridia	Lachnospirales	Lachnospiraceae	Lachnospiraceae_UCG-004	TACGTAGGGGGCAAGCGTTATCCGGATTTACTGGGTGTAAAGGGAGCGTAGACGGCAATACAAGTCGGAAGTGAAATACCCGGGCTCAACCTGGGAACTGCTTTGGAAACTGTATGGCTGGAGTGCTGGAGAGGTAAGCGGAATTCCTAGTGTAGCGGTGAAATGCGTAGATATTAGGAGGAACACCAGTGGCGAAGGCGGCTTACTGGACAGTAACTGACGTTGAGGCTCGAAAGCGTGGGGAGCAAACAGG
ASV294	Bacteria	Firmicutes	Clostridia	Lachnospirales	Lachnospiraceae	Lachnospiraceae_UCG-004	TACGTAGGGGGCAAGCGTTATCCGGATTTACTGGGTGTAAAGGGAGCGTAGACGGCAATACAAGTCGGAAGTGAAATACCCGGGCTTAACCTGGGAACTGCTTTGGAAACTGTATGGCTGGAGTGCTGGAGAGGTAAGCGGAATTCCTAGTGTAGCGGTGAAATGCGTAGATATTAGGAGGAACACCAGTGGCGAAGGCGGCTTACTGGACAGTAACTGACGTTGAGGCTCGAAAGCGTGGGGAGCAAACAGG
ASV085	Bacteria	Firmicutes	Clostridia	Lachnospirales	Lachnospiraceae	Lachnospiraceae_Genus	TACGTAGGGGGCAAGCGTTATCCGGATTTACTGGGTGTAAAGGGAGCGTAGACGGCATAGCAAGTCTGAAGTGAAAGCCCAAGGCTCAACCATGGGACTGCTTTGGAAACTGTTAAGCTAGAGTGCTGGAGAGGTAAGCGGAATTCCTAGTGTAGCGGTGAAATGCGTAGATATTAGGAAGAACACCAGTGGCGAAGGCGGCTTACTGGACAGTAACTGACGTTGAGGCTCGAAAGCGTGGGGAGCAAACAGG
ASV318	Bacteria	Firmicutes	Clostridia	Lachnospirales	Lachnospiraceae	Lachnospiraceae_NK4A136_group	TACGTAGGGGGCAAGCGTTATCCGGATTTACTGGGTGTAAAGGGAGCGTAGACGGTTATGCAAGTCTGAAGTGAAAGCCCAAGGCTCAACCATGGGACTGCTTTGGAAACTGTAAAACTGGAGTGCAGGAGAGGTAAGCGGAATTCCTAGTGTAGCGGTGAAATGCGTAGATATTAGGAGGAACACCAGTGGCGAAGGCGGCTTACTGGACTGTAACTGACGTTGAGGCTCGAAGGCGTGGGGAGCAAACAGG
ASV344	Bacteria	Firmicutes	Clostridia	Lachnospirales	Lachnospiraceae	Lachnoclostridium	TACGTAGGGGGCAAGCGTTATCCGGATTTACTGGGTGTAAAGGGAGCGTAGACGGCTGTGCAAGTCTGAAGTGAAAGCCCAGGGCTCAACCCTGGGACTGCTTTGGAAACTGTGGAGCTAGAGTGCTGGAGAGGTAAGTGGAATTCCTAGTGTAGCGGTGAAATGCGTAGATATTAGGAGGAACACCAGTGGCGAAGGCGGCTTACTGGACAGTAACTGACGTTGAGGCTCGAAAGCGTGGGGAGCAAACAGG
ASV051	Bacteria	Firmicutes	Clostridia	Lachnospirales	Lachnospiraceae	Lachnospiraceae_NK4A136_group	TACGTAGGGGGCAAGCGTTATCCGGATTTACTGGGTGTAAAGGGAGCGTAGACGGCTGTGCAAGTCTGAAGTGAAATGCCGGGGCTCAACCCCGGAACTGCTTTGGAAACTGTACAGCTAGAGTGCAGGAGGGGTGAGCGGAATTCCTAGTGTAGCGGTGAAATGCGTAGATATTAGGAGGAACACCAGTGGCGAAGGCGGCTCACTGGACTGTAACTGACGTTGAGGCTCGAAAGCGTGGGGAGCAAACAGG
ASV	Bacteria	Firmicutes	Clostridia	Lachnospirales	Lachnospiraceae	Lachnospiraceae_NK4A136_group	TACGTAGGGGGCAAGCGTTATCCGGATTTACTGGGTGTAAAGGGAGCGTAGACGGCTGTGCAAGTCTGAAGTGAAATGCCGGGGCTCAACCCCGGAACTGCTTTGGAAACTGTACAGCTAGAGTGCAGGAGGGGTGAGCGGAATTCCTAGTGTAGCGGTGAAATGCGTAGATATTAGGAGGAACACCGGTGGCGAAGGCGGCTCACTGGACTGTAACTGACGTTGAGGCTCGAAAGCGTGGGGAGCAAACAGG

049							
ASV023	Bacteria	Firmicutes	Clostridia	Lachnospirales	Lachnospiraceae	Lachnospiraceae_NK4A136_group	TACGTAGGGGGCAAGCGTTATCCGGATTTACTGGGTGTAAAGGGAGCGTAGACGGCTGTGCAAGTCTGAAGTGAAATGCCGGGGCCCAACCCCGGAACTGCTTTGGAAACTGTACAGCTAGAGTGCAGGAGGGGTGAGCGGAATTCCTAGTGTAGCGGTGAAATGCGTAGATATTAGGAGGAACACCGGTGGCGAAGGCGGCTCACTGGACTGTAACTGACGTTGAGGCTCGAAAGCGTGGGGAGCAAACAGG
ASV020	Bacteria	Firmicutes	Clostridia	Lachnospirales	Lachnospiraceae	Lachnospiraceae_NK4A136_group	TACGTAGGGGGCAAGCGTTATCCGGATTTACTGGGTGTAAAGGGAGCGTAGACGGCAGCGCAAGTCTGGAGTGAAATCCCATGGCTTAACCATGGAACTGCTTTGGAAACTGTGCAGCTAGAGTGCAGGAGAGGTAAGCGGAATTCCTAGTGTAGCGGTGAAATGCGTAGATATTAGGAGGAACACCAGTGGCGAAGGCGGCTTACTGGACTGTAACTGACGTTGAGGCTCGAAAGCGTGGGGAGCAAACAGG
ASV016	Bacteria	Firmicutes	Clostridia	Lachnospirales	Lachnospiraceae	Lachnospiraceae_NK4A136_group	TACGTAGGGGGCAAGCGTTATCCGGATTTACTGGGTGTAAAGGGAGCGTAGACGGCAGCGCAAGTCTGGAGTGAAATCCCATGGCTTAACCATGGAACTGCTTTGGAAACTGTGCAGCTGGAGTGCAGGAGAGGTAAGCGGAATTCCTAGTGTAGCGGTGAAATGCGTAGATATTAGGAGGAACACCAGTGGCGAAGGCGGCTTACTGGACTGTAACTGACGTTGAGGCTCGAAAGCGTGGGGAGCAAACAGG
ASV122	Bacteria	Firmicutes	Clostridia	Lachnospirales	Lachnospiraceae	Lachnospiraceae_NK4A136_group	TACGTAGGGGGCAAGCGTTATCCGGATTTACTGGGTGTAAAGGGAGCGTAGACGGCAGCGCAAGTCTGGAGTGAAATGCCGGGGCCCAACCCCGGAACTGCTTTGGAAACTGTGCAGCTCGAGTGCAGGAGAGGTAAGCGGAATTCCTAGTGTAGCGGTGAAATGCGTAGATATTAGGAGGAACACCAGTGGCGAAGGCGGCTTACTGGACTGTAACTGACGTTGAGGCTCGAAAGCGTGGGGAGCAAACAGG
ASV349	Bacteria	Firmicutes	Clostridia	Lachnospirales	Lachnospiraceae	Lachnospiraceae_NK4A136_group	TACGTAGGGGGCAAGCGTTATCCGGATTTACTGGGTGTAAAGGGAGCGTAGACGGCAGCGCAAGTCTGGAGTGAAATGCCGGGGCCCAACCCCGGAACTGCTTTGGAAACTGTGCAGCTCGAGTGCGGGAGAGGTAAGCGGAATTCCTAGTGTAGCGGTGAAATGCGTAGATATTAGGAGGAACACCAGTGGCGAAGGCGGCTTACTGGACTGTAACTGACGTTGAGGCTCGAAAGCGTGGGGAGCAAACAGG
ASV285	Bacteria	Firmicutes	Clostridia	Lachnospirales	Lachnospiraceae	Lachnospiraceae_NK4A136_group	TACGTAGGGGGCAAGCGTTATCCGGATTTACTGGGTGTAAAGGGAGCGTAGACGGCAGCGCAAGTCTGGAGTGAAATGCCGGGGCCCAACCCCGGAACTGCTTTGGAAACTGTGCGGCTCGAGTGCGGGAGAGGTAGGCGGAATTCCTAGTGTAGCGGTGAAATGCGTAGATATTAGGAGGAACACCAGTGGCGAAGGCGGCTTACTGGACCGTAACTGACGTTGAGGCTCGAAAGCGTGGGGAGCAAACAGG
ASV265	Bacteria	Firmicutes	Clostridia	Lachnospirales	Lachnospiraceae	Lachnospiraceae_NK4A136_group	TACGTAGGGGGCAAGCGTTATCCGGATTTACTGGGTGTAAAGGGAGCGTAGACGGCAGCGCAAGTCTGGAGTGAAATGCCGGGGCTCAACCCCGGAACTGCTTTGGAAACTGTGCAGCTCGAGTGCAGGAGAGGTAAGCGGAATTCCTAGTGTAGCGGTGAAATGCGTAGATATTAGGAGGAACACCAGTGGCGAAGGCGGCTTACTGGACTGTAACTGACGTTGAGGCTCGAAAGCGTGGGGAGCAAACAGG
ASV032	Bacteria	Firmicutes	Clostridia	Lachnospirales	Lachnospiraceae	Lachnospiraceae_NK4A136_group	TACGTAGGGGGCAAGCGTTATCCGGATTTACTGGGTGTAAAGGGAGCGTAGACGGCCAGGCAAGTCTGGAGTGAAAGCCCGGGGCTCAACCCCGGGACTGCTTTGGAAACTGTAGGGCTGGAGTGCAGGAGGGGTAAGCGGAATTCCTAGTGTAGCGGTGAAATGCGTAGATATTAGGAGGAACACCGGTGGCGAAGGCGGCTTACTGGACTGCAACTGACGTTGAGGCTCGAAGGCGTGGGGAGCAAACAGG

ASV0400	Bacteria	Firmicutes	Clostridia	Lachnospirales	Lachnospiraceae	Lachnospiraceae_NK4A136_group	TACGTAGGGGGCAAGCGTTATCCGGATTTACTGGGTGTAAAGGGAGCGCAGACGGCTGTGCAAGTCTGGAGTGAAAGGCGGGGGCCCAACCCCCGGACTGCTCTGGAAACTGTAAAGCTGGAGTGCAGGAGAGGTAAGCGGAATTCCTAGTGTAGCGGTGAAATGCGTAGATATTAGGAGGAACACCAGTGGCGAAGGCGGCTTACTGGACTGCAACTGACGTTGAGGCTCGAAAGCGTGGGGAGCAAACAGG
ASV426	Bacteria	Firmicutes	Clostridia	Lachnospirales	Lachnospiraceae	Lachnospiraceae_NK4A136_group	TACGTAGGGGGCAAGCGTTATCCGGAATTACTGGGTGTAAAGGGAGCGTAGACGGTGATGTAAGTCTGGAGTGAAAGGCGGGGGCCCAACCCCCGGACTGCTCTGGAAACTATGTGACTGGAGTGCAGGAGAGGTGAGCGGAATTCCTAGTGTAGCGGTGAAATGCGTAGATATTAGGAGGAACACCAGTGGCGAAGGCGGCTCACTGGACTGTAACTGACGTTGAGGCTCGAAAGCGTGGGGAGCAAACAGG
ASV166	Bacteria	Firmicutes	Clostridia	Lachnospirales	Lachnospiraceae	Lachnospiraceae_NK4A136_group	TACGTAGGGGGCAAGCGTTATCCGGATTTACTGGGTGTAAAGGGAGCGTAGACGGTTGCGCAAGTCTGGAGTGAAAGGCGGGGGCCCAACCCCCGGACTGCTCTGGAAACTGTGTAACTGGAGTGCAGGAGAGGTAAGTGGAATTCCTAGTGTAGCGGTGAAATGCGTAGATATTAGGAGGAACACCAGTGGCGAAGGCGGCTTACTGGACTGTAACTGACGTTGAGGCTCGAAAGCGTGGGGAGCAAACAGG
ASV171	Bacteria	Firmicutes	Clostridia	Lachnospirales	Lachnospiraceae	Lachnospiraceae_NK4A136_group	TACGTAGGGGGCAAGCGTTATCCGGATTTACTGGGTGTAAAGGGAGCGTAGACGGTTGCGCAAGTCTGGAGTGAAAGGCGGGGGCTCAACCCCCGGACTGCTCTGGAAACTGTGTAACTGGAGTGCAGGAGAGGTAAGTGGAATTCCTAGTGTAGCGGTGAAATGCGTAGATATTAGGAGGAACACCAGTGGCGAAGGCGGCTTACTGGACTGTAACTGACGTTGAGGCTCGAAAGCGTGGGGAGCAAACAGG
ASV389	Bacteria	Firmicutes	Clostridia	Lachnospirales	Lachnospiraceae	Lachnospiraceae_NK4A136_group	TACGTAGGGGGCAAGCGTTATCCGGATTTACTGGGTGTAAAGGGAGCGTAGACGGTTGCGCAAGTCTGGAGTGAAAGGCGGGGGCTCACCCCCCGGACTGCTCTGGAAACTGTGTAACTGGAGTGCAGGAGAGGTAAGTGGAATTCCTAGTGTAGCGGTGAAATGCGTAGATATTAGGAGGAACACCAGTGGCGAAGGCGGCTTACTGGACTGTAACTGACGTTGAGGCTCGAAAGCGTGGGGAGCAAACAGG
ASV103	Bacteria	Firmicutes	Clostridia	Lachnospirales	Lachnospiraceae	Lachnospiraceae_NK4A136_group	TACGTAGGGGGCAAGCGTTATCCGGATTTACTGGGTGTAAAGGGAGCGTAGACGGTTGTGCAAGTCTGGAGTGAAAGGCGGGGGCCCAACCCCCGGACTGCTTTGGAAACTGTATAACTGGAGTGCAGGAGAGGTAAGTGGAATTCCTAGTGTAGCGGTGAAATGCGTAGATATTAGGAGGAACACCAGTGGCGAAGGCGGCTTACTGGACTGTAACTGACGTTGAGGCTCGAAAGCGTGGGGAGCAAACAGG
ASV180	Bacteria	Firmicutes	Clostridia	Lachnospirales	Lachnospiraceae	Lachnospiraceae_NK4A136_group	TACGTAGGGGGCAAGCGTTATCCGGATTTACTGGGTGTAAAGGGAGCGTAGACGGTTACGCAAGTCTGAAGTGAAAGGCGGGGGCCCAACCCCCGGACTGCTTTGGAAACTGTGAAACTGGAGTGCAGGAGAGGTAAGTGGAATTCCTAGTGTAGCGGTGAAATGCGTAGATATTAGGAGGAACACCAGTGGCGAAGGCGGCTTACTGGACTGTAACTGACGTTGAGGCTCGAAAGCGTGGGGAGCAAACAGG
ASV228	Bacteria	Firmicutes	Clostridia	Lachnospirales	Lachnospiraceae	Lachnospiraceae_NK4A136_group	TACGTAGGGGGCAAGCGTTATCCGGATTTACTGGGTGTAAAGGGAGCGTAGACGGTTACGCAAGTCTGGAGTGAAAGGCGGGGGCCCAACCCCCGGACTGCTTTGGAAACTGTGAAACTGGAGTGCAGGAGAGGTAAGTGGAATTCCTAGTGTAGCGGTGAAATGCGTAGATATTAGGAGGAACACCAGTGGCGAAGGCGGCTTACTGGACTGTAACTGACGTTGAGGCTCGAAAGCGTGGGGAGCAAACAGG
ASV	Bacteria	Firmicutes	Clostridia	Lachnospirales	Lachnospiraceae	Lachnospiraceae_NK4A136_group	TACGTAGGGGGCAAGCGTTATCCGGATTTACTGGGTGTAAAGGGAGCGTAGACGGTTACGCAAGTCTGGAGTGAAAGGCGGGGGCCCAACCCCCGGACTGCTTTGGAAACTGTATAACTGGAGTGCAGGAGAGGTAAGTGGAATTCCTAGTGTAGCGGTGAAATGCGTAGATATTAGGAGGAACACCAGTGGCGAAGGCGGCTTACTGGACTGTAACTGACGTTGAGGCTCGAAAGCGTGGGGAGCAAACAGG

135							
ASV029	Bacteria	Firmicutes	Clostridia	Lachnospirales	Lachnospiraceae	Lachnospiraceae_NK4A136_group	TACGTAGGGGGCAAGCGTTATCCGGATTTACTGGGTGTAAAGGGAGCGTAGACGGCGATGCAAGTCTGAAGTGAAAGGCGGGGGCCCAACCCCCGGACTGCTTTGGAAACTGTATGGCTGGAGTGCAGGAGAGGTAAGTGGAATTCCTAGTGTAGCGGTGAAATGCGTAGATATTAGGAGGAACACCAGTGGCGAAGGCGGCTTACTGGACTGTAACTGACGTTGAGGCTCGAAAGCGTGGGGAGCAAACAGG
ASV169	Bacteria	Firmicutes	Clostridia	Lachnospirales	Lachnospiraceae	Lachnospiraceae_NK4A136_group	TACGTAGGGGGCAAGCGTTATCCGGAATCACTGGGTGTAAAGGGAGCGTAGACGGCTGTGCAAGCCTGAAGTGAAAGGCGGGGGCCCAACCCCTGGACTGCTTTGGGAACTGTACGGCTGGAGTGCAGGAGAGGTAAGTGGAATTCCTAGTGTAGCGGTGAAATGCGTAGATATTAGGAGGAACACCAGTGGCGAAGGCGGCTTACTGGACTGTAACTGACGTTGAGGCTCGAAAGCGTGGGGAGCAAACAGG
ASV154	Bacteria	Firmicutes	Clostridia	Lachnospirales	Lachnospiraceae	Lachnospiraceae_NK4A136_group	TACGTAGGGGGCAAGCGTTATCCGGAATCACTGGGTGTAAAGGGAGCGTAGACGGCTGAGCAAGCCTGAAGTGAAAGGCGGGGGCCCAACCCCTGGACTGCTTTGGGAACTGTACGGCTGGAGTGCAGGAGAGGTAAGTGGAATTCCTAGTGTAGCGGTGAAATGCGTAGATATTAGGAGGAACACCAGTGGCGAAGGCGGCTTACTGGACTGTAACTGACGTTGAGGCTCGAAAGCGTGGGGAGCAAACAGG
ASV195	Bacteria	Firmicutes	Clostridia	Lachnospirales	Lachnospiraceae	Lachnospiraceae_NK4A136_group	TACGTAGGGGGCAAGCGTTATCCGGAATCACTGGGTGTAAAGGGAGCGTAGACGGCTGTGCAAGCCTGAAGTGAAAGGCGGGGGCCCAACCCCCGGACTGCTTTGGGAACTGTACGGCTGGAGTGCAGGAGAGGTAAGTGGAATTCCTAGTGTAGCGGTGAAATGCGTAGATATTAGGAGGAACACCAGTGGCGAAGGCGGCTTACTGGACTGTAACTGACGTTGAGGCTCGAAAGCGTGGGGAGCAAACAGG
ASV094	Bacteria	Firmicutes	Clostridia	Lachnospirales	Lachnospiraceae	Lachnospiraceae_NK4A136_group	TACGTAGGGGGCAAGCGTTATCCGGAATCACTGGGTGTAAAGGGAGCGTAGACGGCTGAGCAAGCCTGAAGTGAAAGGCGGGGGCCCAACCCCCGGACTGCTTTGGGAACTGTACGGCTGGAGTGCAGGAGAGGTAAGTGGAATTCCTAGTGTAGCGGTGAAATGCGTAGATATTAGGAGGAACACCAGTGGCGAAGGCGGCTTACTGGACTGTAACTGACGTTGAGGCTCGAAAGCGTGGGGAGCAAACAGG
ASV017	Bacteria	Firmicutes	Clostridia	Lachnospirales	Lachnospiraceae	Lachnospiraceae_NK4A136_group	TACGTAGGGGGCAAGCGTTATCCGGAATTACTGGGTGTAAAGGGAGCGTAGACGGCTGTGCAAGTCTGGAGTGAAAGGCGGGGGCCCAACCCCCGGACTGCTCTGGAAACTGTATGGCTGGAGTGCAGGAGAGGTAAGCGGAATTCCTAGTGTAGCGGTGAAATGCGTAGATATTAGGAGGAACACCAGTGGCGAAGGCGGCTTACTGGACTGTAACTGACGTTGAGGCTCGAAAGCGTGGGGAGCAAACAGG
ASV131	Bacteria	Firmicutes	Clostridia	Lachnospirales	Lachnospiraceae	Lachnospiraceae_NK4A136_group	TACGTAGGGGGCAAGCGTTATCCGGATTTACTGGGTGTAAAGGGAGCGTAGACGGCGGGGCAAGTCTGGAGTGAAAGGCCGGGGCCCAACCCCGGGACCGCTCTGGAAACTGCCCGGCTGGAGTGCGGGAGAGGTAAGCGGAATTCCTAGTGTAGCGGTGAAATGCGTAGATATTAGGAGGAACACCAGCGGCGAAGGCGGCTTACTGGACCGTGACTGACGTTGAGGCTCGAAAGCGTGGGGAGCAAACAGG
ASV261	Bacteria	Firmicutes	Clostridia	Lachnospirales	Lachnospiraceae	Lachnospiraceae_NK4A136_group	TACGTAGGGGGCAAGCGTTATCCGGATTCACTGGGTGTAAAGGGAGCGTAGACGGCGAGACAAGTCTGGAGTGAAAGGCCGGGGCCCAACCCCGGGACTGCTCTGGAAACTGCCTGGCTGGAGTACAGGAGAGGTAAGTGGAATTCCTAGTGTAGCGGTGAAATGCGTAGATATTAGGAGGAACACCAGTGGCGAAGGCGGCTTACTGGACTGTAACTGACGTTGAGGCTCGAAAGCGTGGGGAGCAAACAGG

ASV1411	Bacteria	Firmicutes	Clostridia	Lachnospirales	Lachnospiraceae	Lachnospiraceae_NK4A136_group	TACGTAGGGGGCAAGCGTTATCCGGATTTACTGGGTGTAAAGGGAGCGTAGACGGCAGGGCAAGTCTGGAGTGAAAGGCGGGGGCTCAACCCCCGGACTGCTCTGGAAACTGCCAGGCTGGAGTGCAGGAGAGGTAAGTGGAATTCCTAGTGTAGCGGTGAAATGCGTAGATATTAGGAGGAACACCAGTGGCGAAGGCGGCTTACTGGACTGTAACTGACGTTGAGGCTCGAAAGCGTGGGGAGCAAACAGG
ASV030	Bacteria	Firmicutes	Clostridia	Lachnospirales	Lachnospiraceae	Lachnospiraceae_NK4A136_group	TACGTAGGGGGCAAGCGTTATCCGGATTTACTGGGTGTAAAGGGAGCGTAGACGGCAGGGCAAGTCTGGAGTGAAAGGCAGGGGCCCAACCCCTGGACTGCTCTGGAAACTGTCCGGCTGGAGTGCAGGAGAGGTAAGTGGAATTCCTAGTGTAGCGGTGAAATGCGTAGATATTAGGAGGAACACCAGTGGCGAAGGCGGCTTACTGGACTGTAACTGACGTTGAGGCTCGAAAGCGTGGGGAGCAAACAGG
ASV183	Bacteria	Firmicutes	Clostridia	Lachnospirales	Lachnospiraceae	Lachnospiraceae_Genus	TACGTAGGGGGCGAGCGTTATCCGGAATTACTGGGTGTAAAGGGAGCGTAGACGGCGAGGTAAGCCTGAAGTGGAAGCCCGCGGCCCAACCGCGGAACTGCTTTGGGAACTGTTTTGCTGGAGTATGGGAGGGGTAAGCGGAATTCCTGGTGTAGCGGTGAAATGCGTAGATATCAGGAGGAACACCGGTGGCGAAGGCGGCTTACTGGACCATAACTGACGTTGAGGCTCGAAAGCGTGGGGAGCGAACAGG
ASV267	Bacteria	Firmicutes	Clostridia	Lachnospirales	Lachnospiraceae	Lachnospiraceae_Genus	TACGTAGGGGGCAAGCGTTATCCGGAATTACTGGGTGTAAAGGGAGCGTAGACGGCAATGCAAGTCCGGAGTGGAATGCGGCAGCTCAACTGCCGAACTGCTCTGGAAACTGTATGGCTTGAGTGCAGGAGGGGTAAGCGGAATTCCTGGTGTAGCGGTGAAATGCGTAGATATCAGGAGGAACACCGGTGGCGAAGGCGGCTTACTGGACTGTAACTGACGTTGAGGCTCGAAAGCGTGGGGAGCAAACAGG
ASV170	Bacteria	Firmicutes	Clostridia	Lachnospirales	Lachnospiraceae	Lachnospiraceae_Genus	TACGTAGGGGGCAAGCGTTATCCGGATTTACTGGGTGTAAAGGGAGCGTAGACGGCAGCGCAAGCCTGGAGTGAAAGGATGGGGCCCAACCCCATGACTGCTCTGGGAACTGTGCGGCTAGAGTGCCGGAGGGGTAAGCGGAATTCCTAGTGTAGCGGTGAAATGCGTAGATATTAGGAGGAACACCAGTGGCGAAGGCGGCTTACTGGACGGTAACTGACGTTGAGGCTCGAAAGCGTGGGGAGCAAACAGG
ASV089	Bacteria	Firmicutes	Clostridia	Lachnospirales	Lachnospiraceae	Lachnospiraceae_Genus	TACGTAGGGGGCGAGCGTTATCCGGATTTACTGGGTGTAAAGGGAGCGTAGACGGCCATGCAAGCTCGGTGTGAAAGGTGCGGGCACAACCCGCAGACTGCACTGAGAACTGTATGGCTGGAGTGCCGGAGGGGCAGGCGGAATTCCTGGTGTAGCGGTGAAATGCGTAGATATCAGGAGGAACACCGGTGGCGAAGGCGGCCTGCTGGACGGCGACTGACGTTGAGGCTCGAAAGCGTGGGGAGCGAACAGG
ASV050	Bacteria	Firmicutes	Clostridia	Lachnospirales	Lachnospiraceae	Lachnospiraceae_Genus	TACGTAGGGGGCAAGCGTTATCCGGATTTACTGGGTGTAAAGGGGGCGCAGACGGCCATGGCAAGCCCGGTGTGAAAGGCAGGGGCATAACCCCTGGACTGCACTGGGAACTGTCAGGCTGGAGTGCCGGAGGGGTAAGCGGAATTCCTGGTGTAGCGGTGAAATGCGTAGATATCAGGAGGAACACCGGCGGCGAAGGCGGCTTACTGGACGGCAACTGACGTTGAGGCCCGAAAGCGTGGGGAGCGAACAGG
ASV177	Bacteria	Firmicutes	Clostridia	Lachnospirales	Lachnospiraceae	Lachnospiraceae_Genus	TACGTAGGGGGCGAGCGTTATCCGGATTTACTGGGTGTAAAGGGGGCGCAGACGGCCATGGCAAGCCCGGTGTGAAAGGTGCGGGCACAACCCGCAGACTGCACTGGTAACTGCCTGGCTGGAGTGCCGGAGGGGTAAGCGGAATTCCTGGTGTAGCGGTGAAATGCGTAGATATCAGGAGGAACACCGGTGGCGAAGGCGGCTTACTGGACGGCAACTGACGTTGAGGCCCGAAAGCGTGGGGAGCGAACAGG
ASV	Bacteria	Firmicutes	Clostridia	Lachnospirales	Lachnospiraceae	Lachnospiraceae_Genus	TACGTAGGGGGCGAGCGTTATCCGGATTTACTGGGTGTAAAGGGGGCGCAGACGGCCATGGCAAGCCCGGTGTGAAAGGTGCGGGCACAACCCGCAGACTGCACTGGTAACTGTCTGGCTGGAGTGCCGGAGGGGTAAGCGGAATTCCTGGTGTAGCGGTGAAATGCGTAGATATCAGGAGGAACACCGGTGGCGAAGGCGGCTTACTGGACGGCAACTGACGTTGAGGCCCGAAAGCGTGGGGAGCGAACAGG

Table S2. PQ metadata.

	MouseID	Sex	Genotype	Treatment	SeqID	Barcode	Timepoint	FC.train_baseline_motion	FC.train_frz_tone1	FC.train_frz_tone2	FC.context_totalfrzing	GS.Score	OF1.TotDist	OF2.TotDist	OF3.TotDist	YM.Pct.Spon.Alt
m01	m01	M	A53T-L444P	P	T3-1	CTATATTA TCCG	t3	121.75	26.78	75.22	93.36	2.1	-	1117.93	532.314	57.1429
m02	m02	M	A53T	S	T3-2	CAAACTG CGTTG	t3	139.75	13.78	64.56	73.98	1.7	-	202.998	215.412	80
m03	m03	M	A53T-L444P	S	T3-3	CTGACAC GAAT A	t3	140.5	10.67	46	82.44	1.3	-	349.655	399.71	50
m04	m04	M	A53T-L444P	P	T3-4	AGCAGTG CGGTG	t3	180.47	0	33.67	66.13	1	-	1108.7	933.338	55
m05	m05	M	A53T	P	T3-5	GTTGGAC GAAGG	t3	67.28	38.44	44.33	80.41	1	852.929	960.736	563.017	50
m06	m06	M	A53T	S	T3-6	CAGGAAC CAGGA	t3	163.99	0	43.67	88.58	1.7	1504.35	1109.91	739.297	61.5385
m07	m07	M	A53T	P	T3-7	CAAAGGA GCCCG	t3	199.31	0	10.78	65.59	0.6	1156.42	980.74	812.412	56.5217
m08	m08	M	A53T-L444P	S	T3-8	GAATCTG ACAAC	t3	240.31	0	43.44	65.28	1	1602.87	1089.14	782.335	47.8261
m09	m09	M	A53T-L444P	P	T3-9	CTCATCA TGTTC	t3	69.92	40.22	92.22	91.2	0.9	1241.98	1151.33	789.642	50
m10	m10	M	A53T	S	T3-10	TTATCCA GTCCT	t3	189.71	0	45.78	86.8	1	1733.36	1351.12	1489.86	65
m11	m11	M	A53T-L444P	S	T3-11	GAGTCTT GGTAA	t3	189.62	0	64.11	62.99	1	1067.79	677.85	941.056	54.5455
m12	m12	M	A53T-L444P	P	T3-12	CATTTAC ATCAC	t3	155.47	32.33	49.67	96.07	1.1	1013.65	813.405	608.691	72.2222
m13	m13	M	A53T	S	T3-13	TTCTTAAC GCCT	t3	114.08	18.22	89.67	57.1	0.9	2270.81	2133.11	1034.78	46.6667
m14	m14	M	A53T	P	T3-14	AGTAGTT TCCTT	t3	185.89	3.67	42	60.77	1.2	905.01	630.722	589.561	35.7143
m15	m15	M	A53T-L444P	S	T3-15	GACCCGT TTCGC	t3	174.97	23.56	33.44	79.31	1	938.844	1542.26	1238.26	75
m16	m16	M	A53T-L444P	P	T3-16	ATTGCCTT GATT	t3	43.08	41.33	59.89	85.52	1.1	331.979	284.807	285.339	50
m17	m17	M	A53T-L444P	S	T3-17	TTAGGAT TCTAT	t3	183.22	4.78	27.56	69.74	1	2838.36	1959.58	1482.3	50
m18	m18	M	A53T	P	T3-18	GTTTATCT TAAG	t3	105.87	0	0	12.82	1	1844.26	1626.56	1302.19	41.6667

m19	m19	M	A53T	P	T3-19	CAGTCTA GTACG	t3	87.16	70.11	93.56	67.43	0.8	1443.08	1039.73	763.291	36.3636
m20	m20	M	A53T-L444P	P	T3-20	GTGGGAC TGCGC	t3	174.71	0	11	71.89	0.9	1806.96	1674.51	1503.08	58.8235
m21	m21	M	A53T-L444P	S	T3-21	CTCCCGA GCTCC	t3	101.12	41.89	33.78	92.38	0.8	1789.06	1732.73	902.119	59.0909
m22	m22	F	A53T-L444P	P	T3-22	CAGATCC CAACC	t3	79.44	85.67	67.33	-	0.9	1039.29	608.882	340.988	69.2308
m23	m23	F	A53T	P	T3-23	TGACTGC GTTAG	t3	148.06	13.89	65.11	-	0.9	955.228	899.387	232.513	50
m24	m24	F	A53T	S	T3-24	GAGCCCA AAGAG	t3	137.45	0	36.22	-	1	1540.7	996.256	1133.99	64.5161
m25	m25	F	A53T-L444P	S	T3-25	CAACGAA CCATC	t3	131.04	31.33	42.78	-	0.9	1002.37	842.106	722.568	56.25
m26	m26	F	A53T-L444P	P	T3-26	GTTCGCC GCATC	t3	NA	NA	NA		NA		NA	NA	NA
m27	m27	F	A53T	P	T3-27	GTTCGAG TGAAT	t3	244.95	0	6.67	43.81	0.7	2647.24	2022.93	1655.36	51.7241
m28	m28	F	A53T	S	T3-28	CGGCCTA AGTTC	t3	163.57	0	19.44	25.3	0.9	1442.62	1094.92	424.456	58.8235
m29	m29	F	A53T-L444P	S	T3-29	TAGACTT CAGAG	t3	270.83	0	63.44	70.76	0.8	1676.79	1012.16	878.96	39.1304
m30	m30	F	A53T-L444P	S	T3-30	GATAGCA CTCGT	t3	76.57	12.89	93.44	98.66	0.7	711.639	454.562	272.101	14.2857
m31	m31	F	A53T	P	T3-31	ACGTTAA TATTC	t3	186.25	0	21.33	79.8	1.1	815.788	579.348	377.979	66.6667
m32	m32	F	A53T	S	T3-32	CCATGTG GCTCC	t3	178.32	0	54.78	48.44	0.7	1883.72	1835.54	1900.51	75
m33	m33	F	A53T-L444P	P	T3-33	ACACGTT TGGGT	t3	224.41	0	9.44	64.79	0.7	2064.45	1927	1213.36	66.6667
m34	m34	F	A53T	P	T3-34	AGACTAT TTCAT	t3	112.39	13.11	57.56	78.23	1	1027.68	643.202	630.387	52.381
m35	m35	F	A53T	S	T3-35	AGTCCGA GTTGT	t3	194.07	0	52.22	59.49	0.9	1490.91	1165.87	1066.95	58.8235
m36	m36	F	A53T	P	T3-36	GCTGAGC CTTTG	t3	251.28	0	55.78	64.69	1.2	1071.27	958.29	914.974	52.1739

Table S3. DSS taxon.

	Kingdom	Phylum	Class	Order	Family	Genus
raber-2020-dss_ASV001	Bacteria	Bacteroidota	Bacteroidia	Bacteroidales	Muribaculaceae	NA
raber-2020-dss_ASV002	Bacteria	Bacteroidota	Bacteroidia	Bacteroidales	Muribaculaceae	NA
raber-2020-dss_ASV003	Bacteria	Firmicutes	Bacilli	Erysipelotrichales	Erysipelotrichaceae	Turicibacter
raber-2020-dss_ASV004	Bacteria	Firmicutes	Bacilli	Lactobacillales	Lactobacillaceae	Lactobacillus
raber-2020-dss_ASV005	Bacteria	Firmicutes	Clostridia	Lachnospirales	Lachnospiraceae	NA
raber-2020-dss_ASV006	Bacteria	Bacteroidota	Bacteroidia	Bacteroidales	Muribaculaceae	NA
raber-2020-dss_ASV007	Bacteria	Firmicutes	Clostridia	Lachnospirales	Lachnospiraceae	Lachnospiraceae_NK4A136_group

raber-2020-dss_ASV008	Bacteria	Firmicutes	Clostridia	Lachnospirales	Lachnospiraceae	A2
raber-2020-dss_ASV009	Bacteria	Firmicutes	Clostridia	Lachnospirales	Lachnospiraceae	Lachnospiraceae_NK4A136_group
raber-2020-dss_ASV010	Bacteria	Bacteroidota	Bacteroidia	Bacteroidales	Muribaculaceae	NA
raber-2020-dss_ASV011	Bacteria	Bacteroidota	Bacteroidia	Bacteroidales	Muribaculaceae	NA
raber-2020-dss_ASV012	Bacteria	Firmicutes	Clostridia	Lachnospirales	Lachnospiraceae	NA
raber-2020-dss_ASV013	Bacteria	Firmicutes	Clostridia	Lachnospirales	Lachnospiraceae	Lachnospiraceae_FCS020_group
raber-2020-dss_ASV014	Bacteria	Bacteroidota	Bacteroidia	Bacteroidales	Muribaculaceae	NA
raber-2020-dss_ASV015	Bacteria	Firmicutes	Clostridia	Lachnospirales	Lachnospiraceae	Lachnospiraceae_NK4A136_group

raber-2020-dss_ASV016	Bacteria	Firmicutes	Clostridia	Lachnospirales	Lachnospiraceae	Lachnospiraceae_NK4A136_group
raber-2020-dss_ASV017	Bacteria	Firmicutes	Bacilli	Lactobacillales	Lactobacillaceae	Lactobacillus
raber-2020-dss_ASV018	Bacteria	Firmicutes	Clostridia	Lachnospirales	Lachnospiraceae	Lachnospiraceae_NK4A136_group
raber-2020-dss_ASV019	Bacteria	Bacteroidota	Bacteroidia	Bacteroidales	Muribaculaceae	NA
raber-2020-dss_ASV020	Bacteria	Bacteroidota	Bacteroidia	Bacteroidales	Muribaculaceae	NA
raber-2020-dss_ASV021	Bacteria	Firmicutes	Clostridia	Lachnospirales	Lachnospiraceae	NA
raber-2020-dss_ASV022	Bacteria	Firmicutes	Clostridia	Lachnospirales	Lachnospiraceae	Lachnospiraceae_NK4A136_group
raber-2020-dss_ASV023	Bacteria	Firmicutes	Clostridia	Lachnospirales	Lachnospiraceae	Roseburia

raber-2020-dss_ASV024	Bacteria	Firmicutes	Clostridia	Lachnospirales	Lachnospiraceae	Roseburia
raber-2020-dss_ASV025	Bacteria	Firmicutes	Clostridia	Lachnospirales	Lachnospiraceae	Lachnospiraceae_NK4A136_group
raber-2020-dss_ASV026	Bacteria	Firmicutes	Clostridia	Lachnospirales	Lachnospiraceae	Lachnospiraceae_NK4A136_group
raber-2020-dss_ASV027	Bacteria	Firmicutes	Clostridia	Oscillospirales	Ruminococcaceae	NA
raber-2020-dss_ASV028	Bacteria	Firmicutes	Clostridia	Lachnospirales	Lachnospiraceae	Roseburia
raber-2020-dss_ASV029	Bacteria	Bacteroidota	Bacteroidia	Bacteroidales	Muribaculaceae	NA
raber-2020-dss_ASV030	Bacteria	Firmicutes	Clostridia	Lachnospirales	Lachnospiraceae	NA
raber-2020-dss_ASV031	Bacteria	Firmicutes	Clostridia	Lachnospirales	Lachnospiraceae	Roseburia

raber-2020-dss_ASV032	Bacteria	Bacteroidota	Bacteroidia	Bacteroidales	Muribaculaceae	NA
raber-2020-dss_ASV033	Bacteria	Bacteroidota	Bacteroidia	Bacteroidales	Muribaculaceae	NA
raber-2020-dss_ASV034	Bacteria	Bacteroidota	Bacteroidia	Bacteroidales	Muribaculaceae	NA
raber-2020-dss_ASV035	Bacteria	Firmicutes	Clostridia	Lachnospirales	Lachnospiraceae	NA
raber-2020-dss_ASV036	Bacteria	Firmicutes	Bacilli	Erysipelotrichales	Erysipelotrichaceae	Faecalibaculum
raber-2020-dss_ASV037	Bacteria	Firmicutes	Clostridia	Lachnospirales	Lachnospiraceae	Lachnoclostridium
raber-2020-dss_ASV038	Bacteria	Firmicutes	Bacilli	Staphylococcales	Staphylococcaceae	Staphylococcus
raber-2020-dss_ASV039	Bacteria	Firmicutes	Clostridia	Lachnospirales	Lachnospiraceae	Roseburia

raber-2020-dss_ASV040	Bacteria	Bacteroidota	Bacteroidia	Bacteroidales	Muribaculaceae	NA
raber-2020-dss_ASV041	Bacteria	Firmicutes	Clostridia	Lachnospirales	Lachnospiraceae	Lachnospiraceae_NK4A136_group
raber-2020-dss_ASV042	Bacteria	Bacteroidota	Bacteroidia	Bacteroidales	Marinifilaceae	Odoribacter
raber-2020-dss_ASV043	Bacteria	Bacteroidota	Bacteroidia	Bacteroidales	Rikenellaceae	Alistipes
raber-2020-dss_ASV044	Bacteria	Firmicutes	Clostridia	Clostridiales	Clostridiaceae	Clostridium_sensu_stricto_1
raber-2020-dss_ASV045	Bacteria	Bacteroidota	Bacteroidia	Bacteroidales	Rikenellaceae	Alistipes
raber-2020-dss_ASV046	Bacteria	Firmicutes	Clostridia	Lachnospirales	Lachnospiraceae	A2
raber-2020-dss_ASV047	Bacteria	Bacteroidota	Bacteroidia	Bacteroidales	Rikenellaceae	Alistipes

raber-2020-dss_ASV048	Bacteria	Firmicutes	Clostridia	Lachnospirales	Lachnospiraceae	Marvinbryantia
raber-2020-dss_ASV049	Bacteria	Firmicutes	Clostridia	Lachnospirales	Lachnospiraceae	NA
raber-2020-dss_ASV050	Bacteria	Firmicutes	Clostridia	Lachnospirales	Lachnospiraceae	Lachnospiraceae_NK4B4_group
raber-2020-dss_ASV051	Bacteria	Firmicutes	Clostridia	Oscillospirales	Butyricicoccaceae	Butyricicoccus
raber-2020-dss_ASV052	Bacteria	Firmicutes	Clostridia	Lachnospirales	Lachnospiraceae	NA
raber-2020-dss_ASV053	Bacteria	Bacteroidota	Bacteroidia	Bacteroidales	Muribaculaceae	NA
raber-2020-dss_ASV054	Bacteria	Firmicutes	Clostridia	Lachnospirales	Lachnospiraceae	Lachnospiraceae_UCG-001
raber-2020-dss_ASV055	Bacteria	Bacteroidota	Bacteroidia	Bacteroidales	Muribaculaceae	NA

raber-2020-dss_ASV056	Bacteria	Firmicutes	Clostridia	Oscillospirales	Ruminococcaceae	Incertae_Sedis
raber-2020-dss_ASV057	Bacteria	Firmicutes	Clostridia	Lachnospirales	Lachnospiraceae	NA
raber-2020-dss_ASV058	Bacteria	Firmicutes	Clostridia	Clostridia_vadinBB60_group	NA	NA
raber-2020-dss_ASV059	Bacteria	Bacteroidota	Bacteroidia	Bacteroidales	Bacteroidaceae	Bacteroides
raber-2020-dss_ASV060	Bacteria	Firmicutes	Clostridia	Oscillospirales	Oscillospiraceae	Oscillibacter
raber-2020-dss_ASV061	Bacteria	Firmicutes	Bacilli	Erysipelotrichales	Erysipelotrichaceae	Dubosiella
raber-2020-dss_ASV062	Bacteria	Firmicutes	Clostridia	Lachnospirales	Lachnospiraceae	Lachnospiraceae_NK4A136_group
raber-2020-dss_ASV063	Bacteria	Bacteroidota	Bacteroidia	Bacteroidales	Muribaculaceae	NA

raber-2020-dss_ASV065	Bacteria	Firmicutes	Clostridia	Oscillospirales	Ruminococcaceae	NA
raber-2020-dss_ASV066	Bacteria	Firmicutes	Clostridia	Oscillospirales	NA	NA
raber-2020-dss_ASV067	Bacteria	Firmicutes	Clostridia	Lachnospirales	Lachnospiraceae	Lachnospiraceae_NK4A136_group
raber-2020-dss_ASV068	Bacteria	Firmicutes	Clostridia	Oscillospirales	Oscillospiraceae	NA
raber-2020-dss_ASV069	Bacteria	Firmicutes	Clostridia	Lachnospirales	Lachnospiraceae	NA
raber-2020-dss_ASV070	Bacteria	Firmicutes	Clostridia	Lachnospirales	Lachnospiraceae	Roseburia
raber-2020-dss_ASV071	Bacteria	Firmicutes	Clostridia	Lachnospirales	Lachnospiraceae	NA
raber-2020-dss_ASV072	Bacteria	Bacteroidota	Bacteroidia	Bacteroidales	Rikenellaceae	Alistipes

raber-2020-dss_ASV073	Bacteria	Firmicutes	Clostridia	Oscillospirales	Oscillospiraceae	Oscillibacter
raber-2020-dss_ASV074	Bacteria	Firmicutes	Clostridia	Lachnospirales	Lachnospiraceae	Acetatifactor
raber-2020-dss_ASV075	Bacteria	Firmicutes	Clostridia	Lachnospirales	Lachnospiraceae	NA
raber-2020-dss_ASV076	Bacteria	Firmicutes	Clostridia	Lachnospirales	Lachnospiraceae	Acetatifactor
raber-2020-dss_ASV077	Bacteria	Firmicutes	Clostridia	Lachnospirales	Lachnospiraceae	Roseburia
raber-2020-dss_ASV078	Bacteria	Firmicutes	Clostridia	Lachnospirales	Lachnospiraceae	NA
raber-2020-dss_ASV079	Bacteria	Firmicutes	Bacilli	RF39	NA	NA
raber-2020-dss_ASV080	Bacteria	Bacteroidota	Bacteroidia	Bacteroidales	Muribaculaceae	NA

raber-2020-dss_ASV081	Bacteria	Firmicutes	Clostridia	Oscillospirales	Oscillospiraceae	NA
raber-2020-dss_ASV082	Bacteria	Firmicutes	Clostridia	Lachnospirales	Lachnospiraceae	NA
raber-2020-dss_ASV083	Bacteria	Firmicutes	Clostridia	Lachnospirales	Lachnospiraceae	Lachnospiraceae_NK4A136_group
raber-2020-dss_ASV084	Bacteria	Firmicutes	Clostridia	Lachnospirales	Lachnospiraceae	NA
raber-2020-dss_ASV085	Bacteria	Firmicutes	Clostridia	Lachnospirales	Lachnospiraceae	NA
raber-2020-dss_ASV086	Bacteria	Bacteroidota	Bacteroidia	Bacteroidales	Muribaculaceae	NA
raber-2020-dss_ASV087	Bacteria	Firmicutes	Clostridia	Lachnospirales	Lachnospiraceae	Lachnospiraceae_NK4A136_group
raber-2020-dss_ASV088	Bacteria	Firmicutes	Clostridia	Oscillospirales	Oscillospiraceae	Colidextribacter

raber-2020-dss_ASV089	Bacteria	Firmicutes	Clostridia	Lachnospirales	Lachnospiraceae	NA
raber-2020-dss_ASV090	Bacteria	Bacteroidota	Bacteroidia	Bacteroidales	Prevotellaceae	Prevotellaceae_UCG-001
raber-2020-dss_ASV091	Bacteria	Firmicutes	Clostridia	Clostridia_vadinBB60_group	NA	NA
raber-2020-dss_ASV092	Bacteria	Bacteroidota	Bacteroidia	Bacteroidales	Muribaculaceae	NA
raber-2020-dss_ASV093	Bacteria	Firmicutes	Clostridia	Lachnospirales	Lachnospiraceae	GCA-900066575
raber-2020-dss_ASV094	Bacteria	Firmicutes	Clostridia	Oscillospirales	Ruminococcaceae	Incertae_Sedis
raber-2020-dss_ASV095	Bacteria	Firmicutes	Clostridia	Lachnospirales	Lachnospiraceae	ASF356
raber-2020-dss_ASV096	Bacteria	Firmicutes	Clostridia	Lachnospirales	Lachnospiraceae	NA

raber-2020-dss_ASV097	Bacteria	Firmicutes	Clostridia	Lachnospirales	Lachnospiraceae	Lachnospiraceae_NK4A136_group
raber-2020-dss_ASV098	Bacteria	Bacteroidota	Bacteroidia	Bacteroidales	Muribaculaceae	Muribaculum
raber-2020-dss_ASV099	Bacteria	Firmicutes	Clostridia	Oscillospirales	Oscillospiraceae	NA
raber-2020-dss_ASV100	Bacteria	Firmicutes	Clostridia	Oscillospirales	Oscillospiraceae	NA
raber-2020-dss_ASV101	Bacteria	Firmicutes	Clostridia	Lachnospirales	Lachnospiraceae	NA
raber-2020-dss_ASV102	Bacteria	Firmicutes	Clostridia	Lachnospirales	Lachnospiraceae	Roseburia
raber-2020-dss_ASV103	Bacteria	Firmicutes	Clostridia	Lachnospirales	Lachnospiraceae	NA
raber-2020-dss_ASV104	Bacteria	Firmicutes	Clostridia	Lachnospirales	Lachnospiraceae	Acetatifactor

raber-2020-dss_ASV105	Bacteria	Bacteroidota	Bacteroidia	Bacteroidales	Muribaculaceae	NA
raber-2020-dss_ASV106	Bacteria	Firmicutes	Clostridia	Lachnospirales	Lachnospiraceae	Roseburia
raber-2020-dss_ASV107	Bacteria	Firmicutes	Clostridia	Lachnospirales	Lachnospiraceae	NA
raber-2020-dss_ASV108	Bacteria	Firmicutes	Clostridia	Lachnospirales	Lachnospiraceae	NA
raber-2020-dss_ASV109	Bacteria	Firmicutes	Clostridia	Oscillospirales	Butyricicoccaceae	Butyricicoccus
raber-2020-dss_ASV110	Bacteria	Firmicutes	Clostridia	Oscillospirales	Oscillospiraceae	NA
raber-2020-dss_ASV111	Bacteria	Firmicutes	Clostridia	Oscillospirales	Ruminococcaceae	Incertae_Sedis
raber-2020-dss_ASV112	Bacteria	Firmicutes	Clostridia	Lachnospirales	Lachnospiraceae	NA

raber-2020-dss_ASV113	Bacteria	Firmicutes	Clostridia	Lachnospirales	Lachnospiraceae	Lachnoclostridium
raber-2020-dss_ASV114	Bacteria	Firmicutes	Clostridia	Clostridiales	Clostridiaceae	Clostridium_sensu_stricto_1
raber-2020-dss_ASV115	Bacteria	Bacteroidota	Bacteroidia	Bacteroidales	Muribaculaceae	NA
raber-2020-dss_ASV116	Bacteria	Firmicutes	Clostridia	Oscillospirales	Ruminococcaceae	Incertae_Sedis
raber-2020-dss_ASV117	Bacteria	Firmicutes	Clostridia	Oscillospirales	Oscillospiraceae	Colidextribacter
raber-2020-dss_ASV118	Bacteria	Firmicutes	Clostridia	Lachnospirales	Lachnospiraceae	Lachnoclostridium
raber-2020-dss_ASV119	Bacteria	Firmicutes	Clostridia	Lachnospirales	Lachnospiraceae	Lachnospiraceae_NK4A136_group
raber-2020-dss_ASV120	Bacteria	Firmicutes	Clostridia	Lachnospirales	Lachnospiraceae	ASF356

raber-2020-dss_ASV121	Bacteria	Firmicutes	Clostridia	Lachnospirales	Lachnospiraceae	Marvinbryantia
raber-2020-dss_ASV122	Bacteria	Bacteroidota	Bacteroidia	Bacteroidales	Muribaculaceae	NA
raber-2020-dss_ASV123	Bacteria	Firmicutes	Clostridia	Lachnospirales	Lachnospiraceae	NA
raber-2020-dss_ASV124	Bacteria	Firmicutes	Clostridia	Lachnospirales	Lachnospiraceae	NA
raber-2020-dss_ASV125	Bacteria	Firmicutes	Clostridia	Oscillospirales	Butyricicoccaceae	UCG-009
raber-2020-dss_ASV126	Bacteria	Firmicutes	Clostridia	Oscillospirales	Oscillospiraceae	Oscillibacter
raber-2020-dss_ASV127	Bacteria	Firmicutes	Clostridia	Lachnospirales	Lachnospiraceae	NA
raber-2020-dss_ASV128	Bacteria	Firmicutes	Clostridia	Lachnospirales	Lachnospiraceae	NA

raber-2020-dss_ASV129	Bacteria	Firmicutes	Bacilli	Erysipelotrichales	Erysipelotrichaceae	Turicibacter
raber-2020-dss_ASV130	Bacteria	Firmicutes	Clostridia	Lachnospirales	Lachnospiraceae	Lachnospiraceae_NK4A136_group
raber-2020-dss_ASV131	Bacteria	Firmicutes	Clostridia	Lachnospirales	Lachnospiraceae	Acetatifactor
raber-2020-dss_ASV132	Bacteria	Firmicutes	Clostridia	Oscillospirales	Ruminococcaceae	Anaerotruncus
raber-2020-dss_ASV133	Bacteria	Firmicutes	Clostridia	Lachnospirales	Lachnospiraceae	NA
raber-2020-dss_ASV134	Bacteria	Firmicutes	Clostridia	Lachnospirales	Lachnospiraceae	NA
raber-2020-dss_ASV135	Bacteria	Firmicutes	Clostridia	Lachnospirales	Lachnospiraceae	NA
raber-2020-dss_ASV136	Bacteria	Firmicutes	Clostridia	Oscillospirales	Oscillospiraceae	NA

raber-2020-dss_ASV137	Bacteria	Firmicutes	Clostridia	Lachnospirales	Lachnospiraceae	Lachnospiraceae_FCS020_group
raber-2020-dss_ASV138	Bacteria	Firmicutes	Clostridia	Lachnospirales	Lachnospiraceae	Lachnospiraceae_NK4A136_group
raber-2020-dss_ASV139	Bacteria	Bacteroidota	Bacteroidia	Bacteroidales	Muribaculaceae	NA
raber-2020-dss_ASV140	Bacteria	Firmicutes	Clostridia	Lachnospirales	Lachnospiraceae	NA
raber-2020-dss_ASV141	Bacteria	Bacteroidota	Bacteroidia	Bacteroidales	Muribaculaceae	NA
raber-2020-dss_ASV142	Bacteria	Firmicutes	Clostridia	Lachnospirales	Lachnospiraceae	NA
raber-2020-dss_ASV143	Bacteria	Firmicutes	Clostridia	Clostridia_vadinBB60_group	NA	NA
raber-2020-dss_ASV144	Bacteria	Bacteroidota	Bacteroidia	Bacteroidales	Prevotellaceae	Alloprevotella

raber-2020-dss_ASV145	Bacteria	Firmicutes	Clostridia	Lachnospirales	Lachnospiraceae	Lachnospiraceae_UCG-001
raber-2020-dss_ASV146	Bacteria	Firmicutes	Clostridia	Lachnospirales	Lachnospiraceae	Lachnospiraceae_UCG-001
raber-2020-dss_ASV147	Bacteria	Firmicutes	Clostridia	Lachnospirales	Lachnospiraceae	GCA-900066575
raber-2020-dss_ASV148	Bacteria	Firmicutes	Clostridia	Oscillospirales	Oscillospiraceae	Intestinimonas
raber-2020-dss_ASV149	Bacteria	Firmicutes	Clostridia	Oscillospirales	Oscillospiraceae	Colidextribacter
raber-2020-dss_ASV150	Bacteria	Bacteroidota	Bacteroidia	Bacteroidales	Muribaculaceae	NA
raber-2020-dss_ASV151	Bacteria	Bacteroidota	Bacteroidia	Bacteroidales	Bacteroidaceae	Bacteroides
raber-2020-dss_ASV152	Bacteria	Firmicutes	Clostridia	Lachnospirales	Lachnospiraceae	Eisenbergiella

raber-2020-dss_ASV153	Bacteria	Firmicutes	Clostridia	Clostridia_UCG-014	NA	NA
raber-2020-dss_ASV154	Bacteria	Firmicutes	Clostridia	Lachnospirales	Lachnospiraceae	Lachnospiraceae_UCG-006
raber-2020-dss_ASV155	Bacteria	Firmicutes	Clostridia	Lachnospirales	Lachnospiraceae	Roseburia
raber-2020-dss_ASV156	Bacteria	Firmicutes	Clostridia	Lachnospirales	Lachnospiraceae	NA
raber-2020-dss_ASV157	Bacteria	Firmicutes	Clostridia	Lachnospirales	Lachnospiraceae	NA
raber-2020-dss_ASV158	Bacteria	Bacteroidota	Bacteroidia	Bacteroidales	Muribaculaceae	NA
raber-2020-dss_ASV159	Bacteria	Firmicutes	Clostridia	Clostridia_UCG-014	NA	NA
raber-2020-dss_ASV160	Bacteria	Firmicutes	Clostridia	Oscillospirales	Butyricicoccaceae	Butyricicoccus

raber-2020-dss_ASV161	Bacteria	Firmicutes	Clostridia	Oscillospirales	Oscillospiraceae	NA
raber-2020-dss_ASV162	Bacteria	Firmicutes	Clostridia	Oscillospirales	Oscillospiraceae	Colidextribacter
raber-2020-dss_ASV163	Bacteria	Firmicutes	Clostridia	Lachnospirales	Lachnospiraceae	NA
raber-2020-dss_ASV164	Bacteria	Firmicutes	Clostridia	Lachnospirales	Lachnospiraceae	Lachnospiraceae_UCG-004
raber-2020-dss_ASV165	Bacteria	Bacteroidota	Bacteroidia	Bacteroidales	Bacteroidaceae	Bacteroides
raber-2020-dss_ASV166	Bacteria	Firmicutes	Clostridia	Lachnospirales	Lachnospiraceae	Lachnospiraceae_NK4A136_group
raber-2020-dss_ASV167	Bacteria	Firmicutes	Clostridia	Oscillospirales	Ruminococcaceae	Anaerotruncus
raber-2020-dss_ASV168	Bacteria	Firmicutes	Clostridia	Lachnospirales	Lachnospiraceae	Roseburia

raber-2020-dss_ASV169	Bacteria	Firmicutes	Bacilli	RF39	NA	NA
raber-2020-dss_ASV170	Bacteria	Firmicutes	Clostridia	Oscillospirales	Oscillospiraceae	Oscillibacter
raber-2020-dss_ASV171	Bacteria	Firmicutes	Clostridia	Clostridia_vadinBB60_group	NA	NA
raber-2020-dss_ASV172	Bacteria	Firmicutes	Clostridia	Lachnospirales	Lachnospiraceae	NA
raber-2020-dss_ASV173	Bacteria	Firmicutes	Clostridia	Lachnospirales	Lachnospiraceae	NA
raber-2020-dss_ASV174	Bacteria	Firmicutes	Clostridia	Lachnospirales	Lachnospiraceae	Lachnospiraceae_UCG-006
raber-2020-dss_ASV175	Bacteria	Firmicutes	Clostridia	Oscillospirales	Oscillospiraceae	NA
raber-2020-dss_ASV176	Bacteria	Firmicutes	Clostridia	Oscillospirales	Oscillospiraceae	NA

raber-2020-dss_ASV177	Bacteria	Firmicutes	Clostridia	Oscillospirales	Oscillospiraceae	Oscillibacter
raber-2020-dss_ASV178	Bacteria	Firmicutes	Clostridia	Clostridiales	Clostridiaceae	Clostridium_sensu_stricto_1
raber-2020-dss_ASV179	Bacteria	Firmicutes	Clostridia	Oscillospirales	Oscillospiraceae	Oscillibacter
raber-2020-dss_ASV180	Bacteria	Firmicutes	Clostridia	Lachnospirales	Lachnospiraceae	Lachnospiraceae_NK4A136_group
raber-2020-dss_ASV181	Bacteria	Bacteroidota	Bacteroidia	Bacteroidales	Muribaculaceae	NA
raber-2020-dss_ASV182	Bacteria	Firmicutes	Clostridia	Clostridia_UCG-014	NA	NA
raber-2020-dss_ASV183	Bacteria	Firmicutes	Clostridia	Oscillospirales	Oscillospiraceae	NA
raber-2020-dss_ASV184	Bacteria	Firmicutes	Clostridia	Lachnospirales	Lachnospiraceae	Lachnoclostridium

raber-2020-dss_ASV185	Bacteria	Bacteroidota	Bacteroidia	Bacteroidales	Muribaculaceae	NA
raber-2020-dss_ASV186	Bacteria	Firmicutes	Clostridia	Lachnospirales	Lachnospiraceae	GCA-900066575
raber-2020-dss_ASV187	Bacteria	Firmicutes	Clostridia	Lachnospirales	Lachnospiraceae	NA
raber-2020-dss_ASV188	Bacteria	Firmicutes	Bacilli	Erysipelotrichales	Erysipelatoclostridiaceae	NA
raber-2020-dss_ASV189	Bacteria	Firmicutes	Clostridia	Lachnospirales	Lachnospiraceae	Anaerostipes
raber-2020-dss_ASV190	Bacteria	Firmicutes	Clostridia	Lachnospirales	Lachnospiraceae	Lachnospiraceae_NK4A136_group
raber-2020-dss_ASV191	Bacteria	Firmicutes	Clostridia	Lachnospirales	Lachnospiraceae	Lachnospiraceae_NK4A136_group
raber-2020-dss_ASV192	Bacteria	Firmicutes	Clostridia	Lachnospirales	Lachnospiraceae	NA

raber-2020-dss_ASV193	Bacteria	Firmicutes	Clostridia	Lachnospirales	Lachnospiraceae	Dorea
raber-2020-dss_ASV194	Bacteria	Firmicutes	Clostridia	Lachnospirales	Lachnospiraceae	Acetatifactor
raber-2020-dss_ASV195	Bacteria	Firmicutes	Clostridia	Oscillospirales	Oscillospiraceae	NA
raber-2020-dss_ASV196	Bacteria	Firmicutes	Bacilli	Erysipelotrichales	Erysipelotrichaceae	NA
raber-2020-dss_ASV197	Bacteria	Firmicutes	Clostridia	Lachnospirales	Lachnospiraceae	Marvinbryantia
raber-2020-dss_ASV198	Bacteria	Firmicutes	Clostridia	Lachnospirales	Lachnospiraceae	A2
raber-2020-dss_ASV199	Bacteria	Firmicutes	Clostridia	Oscillospirales	Ruminococcaceae	NA
raber-2020-dss_ASV200	Bacteria	Firmicutes	Clostridia	Lachnospirales	Lachnospiraceae	Lachnospiraceae_UCG-006

raber-2020-dss_ASV201	Bacteria	Firmicutes	Bacilli	RF39	NA	NA
raber-2020-dss_ASV202	Bacteria	Bacteroidota	Bacteroidia	Bacteroidales	Rikenellaceae	Rikenella
raber-2020-dss_ASV203	Bacteria	Firmicutes	Clostridia	Lachnospirales	Lachnospiraceae	NA
raber-2020-dss_ASV204	Bacteria	Proteobacteria	Gammaproteobacteria	Burkholderiales	Sutterellaceae	Parasutterella
raber-2020-dss_ASV205	Bacteria	Firmicutes	Clostridia	Lachnospirales	Lachnospiraceae	NA
raber-2020-dss_ASV206	Bacteria	Firmicutes	Clostridia	Lachnospirales	Lachnospiraceae	NA
raber-2020-dss_ASV207	Bacteria	Firmicutes	Clostridia	Lachnospirales	Lachnospiraceae	NA
raber-2020-dss_ASV208	Bacteria	Firmicutes	Clostridia	Oscillospirales	Oscillospiraceae	Oscillibacter

raber-2020-dss_ASV209	Bacteria	Firmicutes	Clostridia	Lachnospirales	Lachnospiraceae	NA
raber-2020-dss_ASV210	Bacteria	Firmicutes	Clostridia	Lachnospirales	Lachnospiraceae	Lachnospiraceae_NK4A136_group
raber-2020-dss_ASV211	Bacteria	Firmicutes	Clostridia	Oscillospirales	Butyricicoccaceae	Butyricicoccus
raber-2020-dss_ASV213	Bacteria	Desulfobacterota	Desulfovibrionia	Desulfovibrionales	Desulfovibrionaceae	Bilophila
raber-2020-dss_ASV214	Bacteria	Firmicutes	Clostridia	Lachnospirales	Lachnospiraceae	NA
raber-2020-dss_ASV215	Bacteria	Bacteroidota	Bacteroidia	Bacteroidales	Muribaculaceae	NA
raber-2020-dss_ASV216	Bacteria	Firmicutes	Clostridia	Oscillospirales	Oscillospiraceae	NA
raber-2020-dss_ASV217	Bacteria	Firmicutes	Clostridia	Clostridia_vadinBB60_group	NA	NA

raber-2020-dss_ASV218	Bacteria	Firmicutes	Bacilli	Staphylococcales	Staphylococcaceae	Staphylococcus
raber-2020-dss_ASV219	Bacteria	Firmicutes	Clostridia	Lachnospirales	Lachnospiraceae	NA
raber-2020-dss_ASV220	Bacteria	Firmicutes	Bacilli	Acholeplasmatales	Acholeplasmataceae	Anaeroplasma
raber-2020-dss_ASV221	Bacteria	Firmicutes	Clostridia	Lachnospirales	Lachnospiraceae	Roseburia
raber-2020-dss_ASV222	Bacteria	Firmicutes	Clostridia	Clostridiales	Clostridiaceae	Clostridium_sensu_stricto_1
raber-2020-dss_ASV223	Bacteria	Firmicutes	Clostridia	Lachnospirales	Lachnospiraceae	GCA-900066575
raber-2020-dss_ASV224	Bacteria	Firmicutes	Clostridia	Lachnospirales	Lachnospiraceae	NA
raber-2020-dss_ASV225	Bacteria	Firmicutes	Clostridia	Lachnospirales	Lachnospiraceae	Acetatifactor

raber-2020-dss_ASV226	Bacteria	Firmicutes	Clostridia	Oscillospirales	Ruminococcaceae	NA
raber-2020-dss_ASV227	Bacteria	Bacteroidota	Bacteroidia	Bacteroidales	Muribaculaceae	NA
raber-2020-dss_ASV228	Bacteria	Firmicutes	Clostridia	Peptostreptococcales-Tissierellales	Anaerovoracaceae	NA
raber-2020-dss_ASV229	Bacteria	Firmicutes	Clostridia	Clostridia_vadinBB60_group	NA	NA
raber-2020-dss_ASV230	Bacteria	Firmicutes	Clostridia	Oscillospirales	Oscillospiraceae	UCG-003
raber-2020-dss_ASV231	Bacteria	Firmicutes	Clostridia	Lachnospirales	Lachnospiraceae	Lachnoclostridium
raber-2020-dss_ASV232	Bacteria	Firmicutes	Clostridia	Lachnospirales	Lachnospiraceae	NA
raber-2020-dss_ASV233	Bacteria	Firmicutes	Clostridia	Lachnospirales	Lachnospiraceae	Lachnospiraceae_UCG-001

raber-2020-dss_ASV234	Bacteria	Firmicutes	Clostridia	Lachnospirales	Lachnospiraceae	NA
raber-2020-dss_ASV235	Bacteria	Firmicutes	Clostridia	Lachnospirales	Lachnospiraceae	Lachnospiraceae_NK4A136_group
raber-2020-dss_ASV236	Bacteria	Cyanobacteria	Vampirivibrionia	Gastranaerophilales	NA	NA
raber-2020-dss_ASV237	Bacteria	Firmicutes	Clostridia	Lachnospirales	Lachnospiraceae	Lachnospiraceae_NK4A136_group
raber-2020-dss_ASV238	Bacteria	Firmicutes	Clostridia	Clostridia_UCG-014	NA	NA
raber-2020-dss_ASV239	Bacteria	Firmicutes	Clostridia	Lachnospirales	Lachnospiraceae	NA
raber-2020-dss_ASV240	Bacteria	Bacteroidota	Bacteroidia	Bacteroidales	Muribaculaceae	NA
raber-2020-dss_ASV241	Bacteria	Bacteroidota	Bacteroidia	Bacteroidales	Muribaculaceae	NA

raber-2020-dss_ASV242	Bacteria	Actinobacteriota	Coriobacteriia	Coriobacteriales	Eggerthellaceae	Adlercreutzia
raber-2020-dss_ASV243	Bacteria	Firmicutes	Clostridia	Clostridia_UCG-014	NA	NA
raber-2020-dss_ASV244	Bacteria	Firmicutes	Clostridia	Lachnospirales	Lachnospiraceae	Lachnoclostridium
raber-2020-dss_ASV245	Bacteria	Firmicutes	Bacilli	Erysipelotrichales	Erysipelotrichaceae	Faecalibaculum
raber-2020-dss_ASV246	Bacteria	Cyanobacteria	Vampirivibrionia	Gastranaerophilales	NA	NA
raber-2020-dss_ASV247	Bacteria	Firmicutes	Clostridia	Lachnospirales	Lachnospiraceae	Roseburia
raber-2020-dss_ASV248	Bacteria	Bacteroidota	Bacteroidia	Bacteroidales	Rikenellaceae	Rikenella
raber-2020-dss_ASV249	Bacteria	Firmicutes	Clostridia	Oscillospirales	Oscillospiraceae	Colidextribacter

raber-2020-dss_ASV250	Bacteria	Firmicutes	Clostridia	Lachnospirales	Lachnospiraceae	Lachnospiraceae_NK4A136_group
raber-2020-dss_ASV251	Bacteria	Firmicutes	Clostridia	Oscillospirales	Oscillospiraceae	Colidextribacter
raber-2020-dss_ASV252	Bacteria	Actinobacteriota	Coriobacteriia	Coriobacteriales	Eggerthellaceae	Enterorhabdus
raber-2020-dss_ASV253	Bacteria	Bacteroidota	Bacteroidia	Bacteroidales	Muribaculaceae	NA
raber-2020-dss_ASV254	Bacteria	Firmicutes	Clostridia	Lachnospirales	Lachnospiraceae	Lachnospiraceae_NK4A136_group
raber-2020-dss_ASV255	Bacteria	Bacteroidota	Bacteroidia	Bacteroidales	Muribaculaceae	NA
raber-2020-dss_ASV256	Bacteria	Firmicutes	Clostridia	Peptostreptococcales-Tissierellales	Anaerovoracaceae	NA
raber-2020-dss_ASV257	Bacteria	Firmicutes	Clostridia	Lachnospirales	Lachnospiraceae	Lachnospiraceae_NK4A136_group

raber-2020-dss_ASV258	Bacteria	Firmicutes	Bacilli	Lactobacillales	Lactobacillaceae	Lactobacillus
raber-2020-dss_ASV259	Bacteria	Bacteroidota	Bacteroidia	Bacteroidales	Muribaculaceae	NA
raber-2020-dss_ASV260	Bacteria	Firmicutes	Clostridia	Clostridia_UCG-014	NA	NA
raber-2020-dss_ASV261	Bacteria	Firmicutes	Clostridia	Oscillospirales	Ruminococcaceae	Paludicola
raber-2020-dss_ASV263	Bacteria	Firmicutes	Clostridia	Oscillospirales	Oscillospiraceae	Intestinimonas
raber-2020-dss_ASV264	Bacteria	Firmicutes	Clostridia	Clostridia_vadinBB60_group	NA	NA
raber-2020-dss_ASV265	Bacteria	Firmicutes	Clostridia	Clostridia_UCG-014	NA	NA
raber-2020-dss_ASV266	Bacteria	Bacteroidota	Bacteroidia	Bacteroidales	Muribaculaceae	NA

raber-2020-dss_ASV267	Bacteria	Firmicutes	Clostridia	Oscillospirales	Oscillospiraceae	Oscillibacter
raber-2020-dss_ASV268	Bacteria	Firmicutes	Clostridia	Lachnospirales	Lachnospiraceae	UC5-1-2E3
raber-2020-dss_ASV269	Bacteria	Bacteroidota	Bacteroidia	Bacteroidales	Muribaculaceae	NA
raber-2020-dss_ASV270	Bacteria	Proteobacteria	Gammaproteobacteria	Burkholderiales	Sutterellaceae	Parasutterella
raber-2020-dss_ASV271	Bacteria	Firmicutes	Clostridia	Oscillospirales	Oscillospiraceae	Oscillibacter
raber-2020-dss_ASV272	Bacteria	Firmicutes	Clostridia	Lachnospirales	Lachnospiraceae	Lachnospiraceae_UCG-010
raber-2020-dss_ASV273	Bacteria	Firmicutes	Clostridia	Lachnospirales	Lachnospiraceae	Acetatifactor
raber-2020-dss_ASV274	Bacteria	Bacteroidota	Bacteroidia	Bacteroidales	Muribaculaceae	NA

raber-2020-dss_ASV275	Bacteria	Bacteroidota	Bacteroidia	Bacteroidales	Muribaculaceae	NA
raber-2020-dss_ASV276	Bacteria	Firmicutes	Bacilli	Staphylococcales	Staphylococcaceae	Jeotgalicoccus
raber-2020-dss_ASV277	Bacteria	Firmicutes	Bacilli	Erysipelotrichales	Erysipelotrichaceae	Dubosiella
raber-2020-dss_ASV278	Bacteria	Firmicutes	Clostridia	Lachnospirales	Lachnospiraceae	NA
raber-2020-dss_ASV280	Bacteria	Firmicutes	Bacilli	Lactobacillales	Lactobacillaceae	Lactobacillus
raber-2020-dss_ASV281	Bacteria	Actinobacteriota	Coriobacteriia	Coriobacteriales	Eggerthellaceae	DNF00809
raber-2020-dss_ASV282	Bacteria	Firmicutes	Clostridia	Clostridia_vadinBB60_group	NA	NA
raber-2020-dss_ASV283	Bacteria	Firmicutes	Clostridia	Lachnospirales	Lachnospiraceae	Lachnospiraceae_NK4A136_group

raber-2020-dss_ASV284	Bacteria	Firmicutes	Clostridia	Oscillospirales	Ruminococcaceae	Anaerotruncus
raber-2020-dss_ASV285	Bacteria	Firmicutes	Clostridia	Lachnospirales	Lachnospiraceae	Marvinbryantia
raber-2020-dss_ASV286	Bacteria	Firmicutes	Clostridia	Oscillospirales	Oscillospiraceae	NK4A214_group
raber-2020-dss_ASV287	Bacteria	Firmicutes	Clostridia	Peptococcales	Peptococcaceae	NA
raber-2020-dss_ASV289	Bacteria	Firmicutes	Clostridia	Lachnospirales	Lachnospiraceae	NA
raber-2020-dss_ASV290	Bacteria	Firmicutes	Clostridia	Peptostreptococcales-Tissierellales	Anaerovoracaceae	Family_XIII_AD3011_group
raber-2020-dss_ASV291	Bacteria	Firmicutes	Bacilli	RF39	NA	NA
raber-2020-dss_ASV292	Bacteria	Firmicutes	Bacilli	Lactobacillales	Lactobacillaceae	Lactobacillus

raber-2020-dss_ASV293	Bacteria	Firmicutes	Bacilli	Erysipelotrichales	Erysipelotrichaceae	NA
raber-2020-dss_ASV294	Bacteria	Firmicutes	Clostridia	Lachnospirales	Lachnospiraceae	Lachnoclostridium
raber-2020-dss_ASV295	Bacteria	Firmicutes	Clostridia	Oscillospirales	Oscillospiraceae	Colidextribacter
raber-2020-dss_ASV297	Bacteria	Firmicutes	Clostridia	Lachnospirales	Lachnospiraceae	NA
raber-2020-dss_ASV298	Bacteria	Firmicutes	Bacilli	RF39	NA	NA
raber-2020-dss_ASV299	Bacteria	Firmicutes	Clostridia	Lachnospirales	Lachnospiraceae	GCA-900066575
raber-2020-dss_ASV300	Bacteria	Firmicutes	Clostridia	Lachnospirales	Lachnospiraceae	Lachnoclostridium
raber-2020-dss_ASV301	Bacteria	Firmicutes	Clostridia	Lachnospirales	Lachnospiraceae	Marvinbryantia

raber-2020-dss_ASV302	Bacteria	Firmicutes	Bacilli	Erysipelotrichales	Erysipelotrichaceae	NA
raber-2020-dss_ASV303	Bacteria	Firmicutes	Clostridia	Lachnospirales	Lachnospiraceae	NA
raber-2020-dss_ASV304	Bacteria	Firmicutes	Clostridia	Lachnospirales	Lachnospiraceae	Roseburia
raber-2020-dss_ASV305	Bacteria	Firmicutes	Clostridia	Clostridia_vadinBB60_group	NA	NA
raber-2020-dss_ASV306	Bacteria	Bacteroidota	Bacteroidia	Bacteroidales	Rikenellaceae	Alistipes
raber-2020-dss_ASV307	Bacteria	Bacteroidota	Bacteroidia	Bacteroidales	Marinifilaceae	Odoribacter
raber-2020-dss_ASV308	Bacteria	Firmicutes	Clostridia	Lachnospirales	Lachnospiraceae	Lachnospiraceae_NK4A136_group
raber-2020-dss_ASV309	Bacteria	Bacteroidota	Bacteroidia	Bacteroidales	Muribaculaceae	NA

raber-2020-dss_ASV310	Bacteria	Bacteroidota	Bacteroidia	Bacteroidales	Marinifilaceae	Odoribacter
raber-2020-dss_ASV311	Bacteria	Firmicutes	Clostridia	Oscillospirales	Ruminococcaceae	Incertae_Sedis
raber-2020-dss_ASV312	Bacteria	Firmicutes	Clostridia	Clostridia_vadinBB60_group	NA	NA
raber-2020-dss_ASV313	Bacteria	Firmicutes	Clostridia	NA	NA	NA
raber-2020-dss_ASV315	Bacteria	Bacteroidota	Bacteroidia	Bacteroidales	Bacteroidaceae	Bacteroides
raber-2020-dss_ASV316	Bacteria	Firmicutes	Clostridia	Lachnospirales	Lachnospiraceae	NA
raber-2020-dss_ASV317	Bacteria	Firmicutes	Clostridia	Lachnospirales	Lachnospiraceae	Lachnospiraceae_NK4A136_group
raber-2020-dss_ASV318	Bacteria	Firmicutes	Clostridia	Clostridia_UCG-014	NA	NA

raber-2020-dss_ASV319	Bacteria	Bacteroidota	Bacteroidia	Bacteroidales	Muribaculaceae	NA
raber-2020-dss_ASV320	Bacteria	Bacteroidota	Bacteroidia	Bacteroidales	Muribaculaceae	NA
raber-2020-dss_ASV321	Bacteria	Bacteroidota	Bacteroidia	Bacteroidales	Muribaculaceae	NA
raber-2020-dss_ASV322	Bacteria	Bacteroidota	Bacteroidia	Bacteroidales	Muribaculaceae	NA
raber-2020-dss_ASV323	Bacteria	Firmicutes	Clostridia	Lachnospirales	Lachnospiraceae	Lachnospiraceae_NK4A136_group
raber-2020-dss_ASV324	Bacteria	Firmicutes	Clostridia	Lachnospirales	Lachnospiraceae	Lachnospiraceae_UCG-006
raber-2020-dss_ASV325	Bacteria	Firmicutes	Clostridia	Clostridia_UCG-014	NA	NA
raber-2020-dss_ASV326	Bacteria	Firmicutes	Bacilli	Lactobacillales	Enterococcaceae	Enterococcus

raber-2020-dss_ASV327	Bacteria	Firmicutes	Clostridia	Lachnospirales	Lachnospiraceae	GCA-900066575
raber-2020-dss_ASV328	Bacteria	Firmicutes	Clostridia	Clostridia_vadinBB60_group	NA	NA
raber-2020-dss_ASV329	Bacteria	Firmicutes	Clostridia	Clostridia_UCG-014	NA	NA
raber-2020-dss_ASV330	Bacteria	Firmicutes	Clostridia	Oscillospirales	Oscillospiraceae	NA
raber-2020-dss_ASV331	Bacteria	Firmicutes	Clostridia	Lachnospirales	Lachnospiraceae	Lachnospiraceae_UCG-004
raber-2020-dss_ASV332	Bacteria	Firmicutes	Clostridia	Oscillospirales	Ruminococcaceae	Incertae_Sedis
raber-2020-dss_ASV333	Bacteria	Firmicutes	Clostridia	Oscillospirales	Oscillospiraceae	NA
raber-2020-dss_ASV334	Bacteria	Firmicutes	Clostridia	Clostridia_vadinBB60_group	NA	NA

raber-2020-dss_ASV335	Bacteria	Firmicutes	Clostridia	Lachnospirales	Lachnospiraceae	NA
raber-2020-dss_ASV336	Bacteria	Firmicutes	Clostridia	Oscillospirales	Oscillospiraceae	NA
raber-2020-dss_ASV337	Bacteria	Firmicutes	Clostridia	Clostridia_UCG-014	NA	NA
raber-2020-dss_ASV338	Bacteria	Firmicutes	Clostridia	Lachnospirales	Lachnospiraceae	Lachnospiraceae_NK4A136_group
raber-2020-dss_ASV339	Bacteria	Firmicutes	Clostridia	Lachnospirales	Lachnospiraceae	Lachnospiraceae_UCG-006
raber-2020-dss_ASV340	Bacteria	Firmicutes	Clostridia	Oscillospirales	Oscillospiraceae	Colidextribacter
raber-2020-dss_ASV341	Bacteria	Firmicutes	Clostridia	Oscillospirales	Oscillospiraceae	NA
raber-2020-dss_ASV342	Bacteria	Firmicutes	Clostridia	Lachnospirales	Lachnospiraceae	NA

raber-2020-dss_ASV343	Bacteria	Firmicutes	Clostridia	Oscillospirales	Oscillospiraceae	Oscillibacter
raber-2020-dss_ASV344	Bacteria	Firmicutes	Clostridia	Lachnospirales	Lachnospiraceae	Acetatifactor
raber-2020-dss_ASV345	Bacteria	Bacteroidota	Bacteroidia	Bacteroidales	Muribaculaceae	NA
raber-2020-dss_ASV346	Bacteria	Bacteroidota	Bacteroidia	Bacteroidales	Rikenellaceae	Rikenella
raber-2020-dss_ASV347	Bacteria	Firmicutes	Clostridia	Lachnospirales	Lachnospiraceae	Lachnoclostridium
raber-2020-dss_ASV348	Bacteria	Bacteroidota	Bacteroidia	Bacteroidales	Muribaculaceae	NA
raber-2020-dss_ASV349	Bacteria	Bacteroidota	Bacteroidia	Bacteroidales	Muribaculaceae	NA
raber-2020-dss_ASV350	Bacteria	Firmicutes	Clostridia	Clostridia_UCG-014	NA	NA

raber-2020-dss_ASV351	Bacteria	Firmicutes	Clostridia	Lachnospirales	Lachnospiraceae	Lachnospiraceae_NK4A136_group
raber-2020-dss_ASV352	Bacteria	Firmicutes	Clostridia	Lachnospirales	Lachnospiraceae	A2
raber-2020-dss_ASV353	Bacteria	Firmicutes	Bacilli	Erysipelotrichales	Erysipelotrichaceae	Breznakia
raber-2020-dss_ASV354	Bacteria	Firmicutes	Clostridia	Lachnospirales	Lachnospiraceae	Lachnospiraceae_NK4A136_group
raber-2020-dss_ASV355	Bacteria	Actinobacteriota	Coriobacteriia	Coriobacteriales	Eggerthellaceae	NA
raber-2020-dss_ASV356	Bacteria	Bacteroidota	Bacteroidia	Bacteroidales	Rikenellaceae	Alistipes
raber-2020-dss_ASV357	Bacteria	Firmicutes	Bacilli	Erysipelotrichales	Erysipelotrichaceae	NA
raber-2020-dss_ASV358	Bacteria	Firmicutes	Clostridia	Lachnospirales	Lachnospiraceae	NA

raber-2020-dss_ASV359	Bacteria	Firmicutes	Clostridia	Lachnospirales	Lachnospiraceae	Lachnospiraceae_NK4A136_group
raber-2020-dss_ASV360	Bacteria	Firmicutes	Clostridia	Oscillospirales	Ruminococcaceae	NA
raber-2020-dss_ASV361	Bacteria	Firmicutes	Clostridia	Clostridia_vadinBB60_group	NA	NA
raber-2020-dss_ASV362	Bacteria	Firmicutes	Clostridia	Lachnospirales	Lachnospiraceae	Lachnoclostridium
raber-2020-dss_ASV363	Bacteria	Firmicutes	Clostridia	Lachnospirales	Lachnospiraceae	Lachnoclostridium
raber-2020-dss_ASV364	Bacteria	Firmicutes	Clostridia	Lachnospirales	Lachnospiraceae	Roseburia
raber-2020-dss_ASV365	Bacteria	Firmicutes	Clostridia	Lachnospirales	Lachnospiraceae	Lachnospiraceae_NK4A136_group
raber-2020-dss_ASV366	Bacteria	Firmicutes	Clostridia	Oscillospirales	Oscillospiraceae	NA

raber-2020-dss_ASV367	Bacteria	Firmicutes	Clostridia	Lachnospirales	Lachnospiraceae	NA
raber-2020-dss_ASV368	Bacteria	Firmicutes	Bacilli	RF39	NA	NA
raber-2020-dss_ASV369	Bacteria	Firmicutes	Clostridia	Clostridia_UCG-014	NA	NA
raber-2020-dss_ASV370	Bacteria	Firmicutes	Clostridia	Lachnospirales	Lachnospiraceae	Lachnoclostridium
raber-2020-dss_ASV371	Bacteria	Firmicutes	Clostridia	Clostridia_vadinBB60_group	NA	NA
raber-2020-dss_ASV372	Bacteria	Firmicutes	Bacilli	RF39	NA	NA
raber-2020-dss_ASV373	Bacteria	Firmicutes	Clostridia	Oscillospirales	Ruminococcaceae	Incertae_Sedis
raber-2020-dss_ASV374	Bacteria	Firmicutes	Clostridia	Lachnospirales	Lachnospiraceae	NA

raber-2020-dss_ASV375	Bacteria	Firmicutes	Clostridia	Oscillospirales	Oscillospiraceae	Colidextribacter
raber-2020-dss_ASV376	Bacteria	Firmicutes	Bacilli	Lactobacillales	Lactobacillaceae	Lactobacillus
raber-2020-dss_ASV377	Bacteria	Firmicutes	Clostridia	Oscillospirales	Ruminococcaceae	NA
raber-2020-dss_ASV378	Bacteria	Firmicutes	Clostridia	Lachnospirales	Lachnospiraceae	Lachnospiraceae_NK4A136_group
raber-2020-dss_ASV379	Bacteria	Firmicutes	Clostridia	Oscillospirales	Oscillospiraceae	Colidextribacter
raber-2020-dss_ASV380	Bacteria	Firmicutes	Bacilli	Lactobacillales	Lactobacillaceae	Lactobacillus
raber-2020-dss_ASV381	Bacteria	Firmicutes	Clostridia	Clostridia_UCG-014	NA	NA
raber-2020-dss_ASV382	Bacteria	Firmicutes	Clostridia	Lachnospirales	Lachnospiraceae	Lachnospiraceae_NK4A136_group

raber-2020-dss_ASV383	Bacteria	Firmicutes	Clostridia	Oscillospirales	Oscillospiraceae	Colidextribacter
raber-2020-dss_ASV384	Bacteria	Actinobacteriota	Coriobacteriia	Coriobacteriales	Eggerthellaceae	NA
raber-2020-dss_ASV385	Bacteria	Firmicutes	Clostridia	Clostridia_UCG-014	NA	NA
raber-2020-dss_ASV386	Bacteria	Firmicutes	Clostridia	Lachnospirales	Lachnospiraceae	Lachnoclostridium
raber-2020-dss_ASV387	Bacteria	Firmicutes	Clostridia	Peptostreptococcales-Tissierellales	Anaerovoracaceae	Family_XIII_UCG-001
raber-2020-dss_ASV388	Bacteria	Bacteroidota	Bacteroidia	Bacteroidales	Muribaculaceae	NA
raber-2020-dss_ASV389	Bacteria	Firmicutes	Clostridia	Oscillospirales	Oscillospiraceae	NK4A214_group
raber-2020-dss_ASV390	Bacteria	Firmicutes	Clostridia	Oscillospirales	Ruminococcaceae	NA

raber-2020-dss_ASV391	Bacteria	Firmicutes	Clostridia	Lachnospirales	Lachnospiraceae	NA
raber-2020-dss_ASV393	Bacteria	Firmicutes	Clostridia	Peptococcales	Peptococcaceae	NA
raber-2020-dss_ASV394	Bacteria	Actinobacteriota	Actinobacteria	Bifidobacteriales	Bifidobacteriaceae	Bifidobacterium
raber-2020-dss_ASV395	Bacteria	Firmicutes	Clostridia	Lachnospirales	Lachnospiraceae	NA
raber-2020-dss_ASV396	Bacteria	Firmicutes	Clostridia	Lachnospirales	Lachnospiraceae	NA
raber-2020-dss_ASV397	Bacteria	Firmicutes	Clostridia	Lachnospirales	Lachnospiraceae	Tuzzerella
raber-2020-dss_ASV398	Bacteria	Bacteroidota	Bacteroidia	Bacteroidales	Muribaculaceae	NA
raber-2020-dss_ASV399	Bacteria	Firmicutes	Clostridia	Clostridia_vadinBB60_group	NA	NA

raber-2020-dss_ASV400	Bacteria	Firmicutes	Clostridia	Clostridia_vadinBB60_group	NA	NA
raber-2020-dss_ASV401	Bacteria	Firmicutes	Clostridia	Lachnospirales	Lachnospiraceae	Lachnospiraceae_UCG-001
raber-2020-dss_ASV402	Bacteria	Firmicutes	Clostridia	Lachnospirales	Lachnospiraceae	NA
raber-2020-dss_ASV403	Bacteria	Firmicutes	Clostridia	Oscillospirales	Ruminococcaceae	NA
raber-2020-dss_ASV404	Bacteria	Firmicutes	Clostridia	Lachnospirales	Lachnospiraceae	GCA-900066575
raber-2020-dss_ASV405	Bacteria	Firmicutes	Clostridia	Oscillospirales	Oscillospiraceae	UCG-005
raber-2020-dss_ASV406	Bacteria	Cyanobacteria	Vampirivibrionia	Gastranaerophilales	NA	NA
raber-2020-dss_ASV407	Bacteria	Firmicutes	Clostridia	Lachnospirales	Lachnospiraceae	Acetatifactor

raber-2020-dss_ASV408	Bacteria	Firmicutes	Clostridia	Oscillospirales	Ruminococcaceae	NA
raber-2020-dss_ASV409	Bacteria	Firmicutes	Clostridia	Lachnospirales	Lachnospiraceae	Lachnospiraceae_NK4A136_group
raber-2020-dss_ASV410	Bacteria	Firmicutes	Clostridia	Clostridia_UCG-014	NA	NA
raber-2020-dss_ASV411	Bacteria	Firmicutes	Clostridia	Lachnospirales	Lachnospiraceae	Lachnoclostridium
raber-2020-dss_ASV412	Bacteria	Bacteroidota	Bacteroidia	Bacteroidales	Muribaculaceae	NA
raber-2020-dss_ASV413	Bacteria	Firmicutes	Clostridia	Oscillospirales	Ruminococcaceae	Negativibacillus
raber-2020-dss_ASV414	Bacteria	Proteobacteria	Gammaproteobacteria	Burkholderiales	Sutterellaceae	Parasutterella
raber-2020-dss_ASV416	Bacteria	Firmicutes	Clostridia	Clostridia_UCG-014	NA	NA

raber-2020-dss_ASV417	Bacteria	Firmicutes	Clostridia	Lachnospirales	Lachnospiraceae	NA
raber-2020-dss_ASV418	Bacteria	Actinobacteriota	Coriobacteriia	Coriobacteriales	Eggerthellaceae	Enterorhabdus
raber-2020-dss_ASV419	Bacteria	Firmicutes	Clostridia	Clostridia_UCG-014	NA	NA
raber-2020-dss_ASV420	Bacteria	Firmicutes	NA	NA	NA	NA
raber-2020-dss_ASV421	Bacteria	Firmicutes	Clostridia	Lachnospirales	Lachnospiraceae	Lachnospiraceae_UCG-001
raber-2020-dss_ASV425	Bacteria	Bacteroidota	Bacteroidia	Bacteroidales	Muribaculaceae	NA
raber-2020-dss_ASV426	Bacteria	Firmicutes	Clostridia	Lachnospirales	Lachnospiraceae	Lachnospiraceae_FCS020_group
raber-2020-dss_ASV427	Bacteria	Firmicutes	Clostridia	Clostridia_UCG-014	NA	NA

raber-2020-dss_ASV429	Bacteria	Bacteroidota	Bacteroidia	Bacteroidales	Muribaculaceae	NA
raber-2020-dss_ASV430	Bacteria	Bacteroidota	Bacteroidia	Bacteroidales	Muribaculaceae	NA
raber-2020-dss_ASV431	Bacteria	Firmicutes	Clostridia	Clostridia_UCG-014	NA	NA
raber-2020-dss_ASV432	Bacteria	Bacteroidota	Bacteroidia	Bacteroidales	Muribaculaceae	NA
raber-2020-dss_ASV433	Bacteria	Actinobacteriota	Coriobacteriia	Coriobacteriales	Eggerthellaceae	DNF00809
raber-2020-dss_ASV434	Bacteria	Bacteroidota	Bacteroidia	Bacteroidales	Muribaculaceae	NA
raber-2020-dss_ASV435	Bacteria	Firmicutes	Clostridia	Lachnospirales	Lachnospiraceae	Lachnoclostridium
raber-2020-dss_ASV436	Bacteria	Actinobacteriota	Coriobacteriia	Coriobacteriales	Eggerthellaceae	DNF00809

raber-2020-dss_ASV437	Bacteria	Firmicutes	Clostridia	Peptostreptococcales-Tissierellales	Anaerovoracaceae	Family_XIII_AD3011_group
raber-2020-dss_ASV438	Bacteria	Firmicutes	Clostridia	Clostridia_vadinBB60_group	NA	NA
raber-2020-dss_ASV439	Bacteria	Firmicutes	Clostridia	Lachnospirales	Lachnospiraceae	NA
raber-2020-dss_ASV440	Bacteria	Firmicutes	Clostridia	Lachnospirales	Lachnospiraceae	Acetatifactor
raber-2020-dss_ASV441	Bacteria	Firmicutes	Clostridia	Clostridia_vadinBB60_group	NA	NA
raber-2020-dss_ASV442	Bacteria	Firmicutes	Clostridia	Lachnospirales	Lachnospiraceae	NA
raber-2020-dss_ASV443	Bacteria	Firmicutes	Clostridia	Lachnospirales	Lachnospiraceae	NA
raber-2020-dss_ASV444	Bacteria	Firmicutes	Clostridia	Oscillospirales	Ruminococcaceae	Incertae_Sedis

raber-2020-dss_ASV445	Bacteria	Firmicutes	Clostridia	Oscillospirales	Ruminococcaceae	NA
raber-2020-dss_ASV446	Bacteria	Firmicutes	Clostridia	Lachnospirales	Lachnospiraceae	NA
raber-2020-dss_ASV447	Bacteria	Firmicutes	Clostridia	Lachnospirales	Lachnospiraceae	Lachnospiraceae_NK4A136_group
raber-2020-dss_ASV448	Bacteria	Bacteroidota	Bacteroidia	Bacteroidales	Muribaculaceae	NA
raber-2020-dss_ASV449	Bacteria	Firmicutes	Clostridia	Lachnospirales	Lachnospiraceae	NA
raber-2020-dss_ASV451	Bacteria	Bacteroidota	Bacteroidia	Bacteroidales	Muribaculaceae	NA
raber-2020-dss_ASV452	Bacteria	Firmicutes	Clostridia	Lachnospirales	Lachnospiraceae	NA
raber-2020-dss_ASV453	Bacteria	Bacteroidota	Bacteroidia	Bacteroidales	Muribaculaceae	NA

raber-2020-dss_ASV454	Bacteria	Firmicutes	Clostridia	Peptostreptococcales-Tissierellales	Anaerovoracaceae	Family_XIII_UCG-001
raber-2020-dss_ASV455	Bacteria	Firmicutes	Clostridia	Lachnospirales	Lachnospiraceae	Lachnoclostridium
raber-2020-dss_ASV456	Bacteria	Firmicutes	Clostridia	Lachnospirales	Lachnospiraceae	Acetatifactor
raber-2020-dss_ASV457	Bacteria	Bacteroidota	Bacteroidia	Bacteroidales	Muribaculaceae	NA
raber-2020-dss_ASV458	Bacteria	Firmicutes	Clostridia	Clostridia_vadinBB60_group	NA	NA
raber-2020-dss_ASV459	Bacteria	Firmicutes	Clostridia	Oscillospirales	Ruminococcaceae	NA
raber-2020-dss_ASV460	Bacteria	Firmicutes	Clostridia	Lachnospirales	Lachnospiraceae	Lachnospiraceae_UCG-006
raber-2020-dss_ASV461	Bacteria	Bacteroidota	Bacteroidia	Bacteroidales	Muribaculaceae	NA

raber-2020-dss_ASV462	Bacteria	Firmicutes	Clostridia	Clostridia_UCG-014	NA	NA
raber-2020-dss_ASV463	Bacteria	Firmicutes	Bacilli	RF39	NA	NA
raber-2020-dss_ASV464	Bacteria	Firmicutes	Clostridia	Oscillospirales	Oscillospiraceae	NA
raber-2020-dss_ASV465	Bacteria	Firmicutes	Clostridia	Oscillospirales	Ruminococcaceae	NA
raber-2020-dss_ASV466	Bacteria	Firmicutes	Clostridia	Lachnospirales	Lachnospiraceae	Tuzzerella
raber-2020-dss_ASV467	Bacteria	Desulfobacterota	Desulfovibrionia	Desulfovibrionales	Desulfovibrionaceae	Desulfovibrio
raber-2020-dss_ASV468	Bacteria	Firmicutes	Clostridia	Oscillospirales	Oscillospiraceae	Oscillibacter
raber-2020-dss_ASV469	Bacteria	Actinobacteriota	Coriobacteriia	Coriobacteriales	Eggerthellaceae	NA

raber-2020-dss_ASV470	Bacteria	Firmicutes	Clostridia	Oscillospirales	Ruminococcaceae	Incertae_Sedis
raber-2020-dss_ASV471	Bacteria	Actinobacteriota	Coriobacteriia	Coriobacteriales	Eggerthellaceae	NA
raber-2020-dss_ASV472	Bacteria	Firmicutes	Clostridia	Clostridia_UCG-014	NA	NA
raber-2020-dss_ASV473	Bacteria	Firmicutes	Clostridia	Lachnospirales	Lachnospiraceae	NA
raber-2020-dss_ASV474	Bacteria	Firmicutes	Clostridia	Oscillospirales	Oscillospiraceae	UCG-003
raber-2020-dss_ASV475	Bacteria	Firmicutes	Clostridia	Peptococcales	Peptococcaceae	NA
raber-2020-dss_ASV478	Bacteria	Firmicutes	Clostridia	Clostridia_UCG-014	NA	NA
raber-2020-dss_ASV480	Bacteria	Firmicutes	Clostridia	Oscillospirales	Oscillospiraceae	NA

raber-2020-dss_ASV481	Bacteria	Firmicutes	Clostridia	Oscillospirales	Ruminococcaceae	Harryflintia
raber-2020-dss_ASV482	Bacteria	Firmicutes	Clostridia	Lachnospirales	Lachnospiraceae	NA
raber-2020-dss_ASV483	Bacteria	Firmicutes	Clostridia	Oscillospirales	Oscillospiraceae	Colidextribacter
raber-2020-dss_ASV484	Bacteria	Firmicutes	Clostridia	Oscillospirales	Oscillospiraceae	NA
raber-2020-dss_ASV485	Bacteria	Firmicutes	Clostridia	Lachnospirales	Lachnospiraceae	Lachnospiraceae_FCS020_group
raber-2020-dss_ASV488	Bacteria	Bacteroidota	Bacteroidia	Bacteroidales	Muribaculaceae	NA
raber-2020-dss_ASV489	Bacteria	Firmicutes	Clostridia	Lachnospirales	Lachnospiraceae	Roseburia
raber-2020-dss_ASV490	Bacteria	Firmicutes	Clostridia	Oscillospirales	Oscillospiraceae	Intestinimonas

raber-2020-dss_ASV491	Bacteria	Firmicutes	Clostridia	Oscillospirales	Oscillospiraceae	NA
raber-2020-dss_ASV492	Bacteria	Firmicutes	Clostridia	Lachnospirales	Lachnospiraceae	NA
raber-2020-dss_ASV493	Bacteria	Firmicutes	Clostridia	Lachnospirales	Lachnospiraceae	NA
raber-2020-dss_ASV495	Bacteria	Firmicutes	Clostridia	Clostridia_UCG-014	NA	NA
raber-2020-dss_ASV496	Bacteria	Firmicutes	Clostridia	Lachnospirales	Lachnospiraceae	Lachnospiraceae_UCG-010
raber-2020-dss_ASV497	Bacteria	Firmicutes	Clostridia	Lachnospirales	Lachnospiraceae	NA
raber-2020-dss_ASV498	Bacteria	Firmicutes	Clostridia	Lachnospirales	Lachnospiraceae	Roseburia
raber-2020-dss_ASV500	Bacteria	Firmicutes	Clostridia	Lachnospirales	Lachnospiraceae	Lachnoclostridium

raber-2020-dss_ASV501	Bacteria	Firmicutes	Clostridia	Lachnospirales	Lachnospiraceae	Lachnoclostridium
raber-2020-dss_ASV503	Bacteria	Firmicutes	Clostridia	Oscillospirales	Oscillospiraceae	NA
raber-2020-dss_ASV504	Bacteria	Firmicutes	Clostridia	Lachnospirales	Lachnospiraceae	NA
raber-2020-dss_ASV505	Bacteria	Firmicutes	Clostridia	Lachnospirales	Lachnospiraceae	NA
raber-2020-dss_ASV506	Bacteria	Firmicutes	Clostridia	Peptostreptococcales-Tissierellales	Anaerovoracaceae	NA
raber-2020-dss_ASV507	Bacteria	Firmicutes	Bacilli	Lactobacillales	Streptococcaceae	Streptococcus
raber-2020-dss_ASV509	Bacteria	Firmicutes	Bacilli	Erysipelotrichales	Erysipelotrichaceae	NA
raber-2020-dss_ASV510	Bacteria	Firmicutes	Clostridia	Lachnospirales	Lachnospiraceae	Roseburia

raber-2020-dss_ASV511	Bacteria	Firmicutes	Clostridia	Lachnospirales	Lachnospiraceae	NA
raber-2020-dss_ASV513	Bacteria	Firmicutes	Clostridia	Clostridia_vadinBB60_group	NA	NA
raber-2020-dss_ASV515	Bacteria	Firmicutes	Clostridia	Monoglobales	Monoglobaceae	Monoglobus
raber-2020-dss_ASV516	Bacteria	Firmicutes	Clostridia	Oscillospirales	Oscillospiraceae	Oscillospira
raber-2020-dss_ASV518	Bacteria	Firmicutes	Clostridia	Oscillospirales	Ruminococcaceae	NA
raber-2020-dss_ASV520	Bacteria	Firmicutes	Clostridia	Oscillospirales	Oscillospiraceae	UCG-003
raber-2020-dss_ASV523	Bacteria	Firmicutes	Clostridia	Lachnospirales	Lachnospiraceae	Lachnospiraceae_FCS020_group
raber-2020-dss_ASV524	Bacteria	Actinobacteriota	Coriobacteriia	Coriobacteriales	Coriobacteriales_Incertae_Sedis	NA

raber-2020-dss_ASV525	Bacteria	Firmicutes	Clostridia	Lachnospirales	Lachnospiraceae	NA
raber-2020-dss_ASV527	Bacteria	Bacteroidota	Bacteroidia	Bacteroidales	Muribaculaceae	NA
raber-2020-dss_ASV528	Bacteria	Firmicutes	Clostridia	Lachnospirales	Lachnospiraceae	NA
raber-2020-dss_ASV529	Bacteria	Bacteroidota	Bacteroidia	Bacteroidales	Muribaculaceae	NA
raber-2020-dss_ASV530	Bacteria	Firmicutes	Bacilli	Lactobacillales	Leuconostocaceae	Weissella
raber-2020-dss_ASV531	Bacteria	Actinobacteriota	Coriobacteriia	Coriobacteriales	Atopobiaceae	Olsenella
raber-2020-dss_ASV533	Bacteria	Firmicutes	Clostridia	Oscillospirales	Oscillospiraceae	Oscillibacter
raber-2020-dss_ASV536	Bacteria	Firmicutes	Clostridia	Clostridiales	Clostridiaceae	Candidatus_Arthromitus

raber-2020-dss_ASV537	Bacteria	Firmicutes	Bacilli	RF39	NA	NA
raber-2020-dss_ASV539	Bacteria	Firmicutes	Clostridia	Lachnospirales	Lachnospiraceae	Lachnoclostridium
raber-2020-dss_ASV543	Bacteria	Firmicutes	Clostridia	Lachnospirales	Lachnospiraceae	NA
raber-2020-dss_ASV544	Bacteria	Actinobacteriota	Coriobacteriia	Coriobacteriales	Eggerthellaceae	Enterorhabdus
raber-2020-dss_ASV547	Bacteria	Actinobacteriota	Coriobacteriia	Coriobacteriales	Eggerthellaceae	NA
raber-2020-dss_ASV549	Bacteria	Firmicutes	Clostridia	Lachnospirales	Lachnospiraceae	Acetatifactor
raber-2020-dss_ASV550	Bacteria	Bacteroidota	Bacteroidia	Bacteroidales	Muribaculaceae	NA
raber-2020-dss_ASV554	Bacteria	Firmicutes	Clostridia	Oscillospirales	Ruminococcaceae	NA

raber-2020-dss_ASV561	Bacteria	Firmicutes	Clostridia	Lachnospirales	Lachnospiraceae	NA
raber-2020-dss_ASV562	Bacteria	Firmicutes	Clostridia	Oscillospirales	Oscillospiraceae	Colidextribacter
raber-2020-dss_ASV563	Bacteria	Firmicutes	Clostridia	Oscillospirales	Ruminococcaceae	Incertae_Sedis
raber-2020-dss_ASV564	Bacteria	Firmicutes	Clostridia	Lachnospirales	Lachnospiraceae	Lachnoclostridium
raber-2020-dss_ASV566	Bacteria	Bacteroidota	Bacteroidia	Bacteroidales	Muribaculaceae	NA
raber-2020-dss_ASV569	Bacteria	Bacteroidota	Bacteroidia	Bacteroidales	Muribaculaceae	NA
raber-2020-dss_ASV570	Bacteria	Bacteroidota	Bacteroidia	Bacteroidales	Muribaculaceae	NA
raber-2020-dss_ASV573	Bacteria	Firmicutes	Clostridia	Oscillospirales	Ruminococcaceae	NA

raber-2020-dss_ASV574	Bacteria	Proteobacteria	Gammaproteobacteria	Burkholderiales	Sutterellaceae	Parasutterella
raber-2020-dss_ASV583	Bacteria	Actinobacteriota	Coriobacteriia	Coriobacteriales	Eggerthellaceae	DNF00809
raber-2020-dss_ASV584	Bacteria	Firmicutes	Clostridia	Lachnospirales	Lachnospiraceae	Lachnospiraceae_UCG-004
raber-2020-dss_ASV586	Bacteria	Firmicutes	Clostridia	Lachnospirales	Lachnospiraceae	Tuzzerella
raber-2020-dss_ASV587	Bacteria	Firmicutes	Clostridia	Christensenellales	Christensenellaceae	Christensenellaceae_R-7_group
raber-2020-dss_ASV588	Bacteria	Firmicutes	Clostridia	Clostridia_vadinBB60_group	NA	NA
raber-2020-dss_ASV589	Bacteria	Patescibacteria	Saccharimonadia	Saccharimonadales	Saccharimonadaceae	Candidatus_Saccharimonas
raber-2020-dss_ASV591	Bacteria	Bacteroidota	Bacteroidia	Bacteroidales	Muribaculaceae	NA

raber-2020-dss_ASV592	Bacteria	Firmicutes	Clostridia	Lachnospirales	Lachnospiraceae	NA
raber-2020-dss_ASV593	Bacteria	Firmicutes	Clostridia	Lachnospirales	Lachnospiraceae	Acetatifactor
raber-2020-dss_ASV594	Bacteria	Proteobacteria	Gammaproteobacteria	Pseudomonadales	Pseudomonadaceae	Pseudomonas
raber-2020-dss_ASV595	Bacteria	Firmicutes	Clostridia	Lachnospirales	Lachnospiraceae	NA
raber-2020-dss_ASV597	Bacteria	Actinobacteriota	Coriobacteriia	Coriobacteriales	Eggerthellaceae	Enterorhabdus
raber-2020-dss_ASV601	Bacteria	Firmicutes	Clostridia	NA	NA	NA
raber-2020-dss_ASV602	Bacteria	Firmicutes	Bacilli	Erysipelotrichales	Erysipelatoclostridiaceae	NA
raber-2020-dss_ASV605	Bacteria	Firmicutes	Clostridia	Lachnospirales	Lachnospiraceae	NA

raber-2020-dss_ASV609	Bacteria	Actinobacteriota	Coriobacteriia	Coriobacteriales	Eggerthellaceae	DNF00809
raber-2020-dss_ASV610	Bacteria	Firmicutes	Clostridia	Oscillospirales	Oscillospiraceae	NA
raber-2020-dss_ASV613	Bacteria	Actinobacteriota	Coriobacteriia	Coriobacteriales	Eggerthellaceae	DNF00809
raber-2020-dss_ASV622	Bacteria	Firmicutes	Bacilli	Lactobacillales	Lactobacillaceae	Lactobacillus
raber-2020-dss_ASV625	Bacteria	Firmicutes	Clostridia	Oscillospirales	Ruminococcaceae	NA
raber-2020-dss_ASV626	Bacteria	Firmicutes	Clostridia	Lachnospirales	Lachnospiraceae	NA
raber-2020-dss_ASV627	Bacteria	Firmicutes	Bacilli	Erysipelotrichales	Erysipelotrichaceae	Turicibacter
raber-2020-dss_ASV632	Bacteria	Bacteroidota	Bacteroidia	Bacteroidales	Muribaculaceae	NA

raber-2020-dss_ASV633	Bacteria	Firmicutes	Clostridia	Lachnospirales	Lachnospiraceae	NA
raber-2020-dss_ASV636	Bacteria	Bacteroidota	Bacteroidia	Bacteroidales	Rikenellaceae	Alistipes
raber-2020-dss_ASV637	Bacteria	Firmicutes	Clostridia	Lachnospirales	Lachnospiraceae	NA
raber-2020-dss_ASV638	Bacteria	Firmicutes	Clostridia	Oscillospirales	Oscillospiraceae	NA
raber-2020-dss_ASV640	Bacteria	Firmicutes	Clostridia	Lachnospirales	Lachnospiraceae	NA
raber-2020-dss_ASV641	Bacteria	Bacteroidota	Bacteroidia	Bacteroidales	Muribaculaceae	NA
raber-2020-dss_ASV642	Bacteria	Bacteroidota	Bacteroidia	Bacteroidales	Muribaculaceae	CAG-873
raber-2020-dss_ASV643	Bacteria	Firmicutes	Clostridia	Oscillospirales	Oscillospiraceae	Colidextribacter

raber-2020-dss_ASV644	Bacteria	Proteobacteria	Alphaproteobacteria	Rickettsiales	Mitochondria	NA
raber-2020-dss_ASV646	Bacteria	Firmicutes	Clostridia	Oscillospirales	Ruminococcaceae	Incertae_Sedis
raber-2020-dss_ASV650	Bacteria	Patescibacteria	Saccharimonadia	Saccharimonadales	Saccharimonadaceae	Candidatus_Saccharimonas
raber-2020-dss_ASV651	Bacteria	Firmicutes	Clostridia	Lachnospirales	Lachnospiraceae	NA
raber-2020-dss_ASV652	Bacteria	Bacteroidota	Bacteroidia	Bacteroidales	NA	NA
raber-2020-dss_ASV653	Bacteria	Bacteroidota	Bacteroidia	Bacteroidales	Muribaculaceae	NA
raber-2020-dss_ASV658	Bacteria	Firmicutes	Clostridia	Lachnospirales	Lachnospiraceae	NA
raber-2020-dss_ASV659	Bacteria	Firmicutes	Clostridia	Lachnospirales	Lachnospiraceae	NA

raber-2020-dss_ASV661	Bacteria	Firmicutes	NA	NA	NA	NA
raber-2020-dss_ASV662	Bacteria	Firmicutes	Clostridia	Lachnospirales	Lachnospiraceae	NA
raber-2020-dss_ASV664	Bacteria	Firmicutes	Bacilli	Lactobacillales	Lactobacillaceae	Lactobacillus
raber-2020-dss_ASV665	Bacteria	Firmicutes	Clostridia	Oscillospirales	Oscillospiraceae	NA
raber-2020-dss_ASV666	Bacteria	Firmicutes	Clostridia	Lachnospirales	Lachnospiraceae	ASF356
raber-2020-dss_ASV667	Bacteria	Bacteroidota	Bacteroidia	Bacteroidales	NA	NA
raber-2020-dss_ASV668	Bacteria	Bacteroidota	Bacteroidia	Bacteroidales	NA	NA
raber-2020-dss_ASV671	Bacteria	Firmicutes	Clostridia	Oscillospirales	Oscillospiraceae	NA

raber-2020-dss_ASV672	Bacteria	Firmicutes	Clostridia	Christensenellales	Christensenellaceae	NA
raber-2020-dss_ASV673	Bacteria	Bacteroidota	Bacteroidia	Bacteroidales	NA	NA
raber-2020-dss_ASV674	Bacteria	Bacteroidota	Bacteroidia	Bacteroidales	Muribaculaceae	NA
raber-2020-dss_ASV675	Bacteria	Firmicutes	Clostridia	Peptostreptococcales-Tissierellales	Anaerovoracaceae	NA
raber-2020-dss_ASV676	Bacteria	Firmicutes	Clostridia	Oscillospirales	Ruminococcaceae	Phocea
raber-2020-dss_ASV677	Bacteria	Firmicutes	Clostridia	Lachnospirales	Lachnospiraceae	ASF356
raber-2020-dss_ASV678	Bacteria	Firmicutes	Clostridia	NA	NA	NA
raber-2020-dss_ASV679	Bacteria	Bacteroidota	Bacteroidia	Bacteroidales	NA	NA

raber-2020-dss_ASV680	Bacteria	Bacteroidota	Bacteroidia	Bacteroidales	Marinifilaceae	Odoribacter
raber-2020-dss_ASV681	Bacteria	Firmicutes	Bacilli	Erysipelotrichales	Erysipelotrichaceae	Faecalibaculum
raber-2020-dss_ASV683	Bacteria	Bacteroidota	Bacteroidia	Bacteroidales	Muribaculaceae	NA
raber-2020-dss_ASV684	Bacteria	Firmicutes	Clostridia	Oscillospirales	Butyricicoccaceae	NA
raber-2020-dss_ASV685	Bacteria	Firmicutes	Bacilli	Acholeplasmatales	Acholeplasmataceae	Anaeroplasma
raber-2020-dss_ASV686	Bacteria	Firmicutes	Clostridia	Lachnospirales	Lachnospiraceae	NA
raber-2020-dss_ASV687	Bacteria	Firmicutes	Clostridia	Lachnospirales	Lachnospiraceae	NA
raber-2020-dss_ASV688	Bacteria	Firmicutes	Clostridia	Lachnospirales	Lachnospiraceae	NA

raber-2020-dss_ASV690	Bacteria	Firmicutes	Bacilli	Lactobacillales	Leuconostocaceae	Leuconostoc
raber-2020-dss_ASV694	Bacteria	Firmicutes	Clostridia	Oscillospirales	Oscillospiraceae	Sporobacter
raber-2020-dss_ASV695	Bacteria	Firmicutes	Clostridia	Lachnospirales	Lachnospiraceae	NA
raber-2020-dss_ASV696	Bacteria	Firmicutes	Clostridia	Lachnospirales	Lachnospiraceae	NA
raber-2020-dss_ASV697	Bacteria	Firmicutes	Clostridia	NA	NA	NA
raber-2020-dss_ASV698	Bacteria	Firmicutes	Clostridia	Oscillospirales	Ruminococcaceae	NA
raber-2020-dss_ASV700	Bacteria	Firmicutes	Clostridia	Oscillospirales	Oscillospiraceae	NA
raber-2020-dss_ASV701	Bacteria	Patescibacteria	Saccharimonadia	Saccharimonadales	Saccharimonadaceae	Candidatus_Saccharimonas

raber-2020-dss_ASV702	Bacteria	Firmicutes	Clostridia	Lachnospirales	Lachnospiraceae	NA
raber-2020-dss_ASV703	Bacteria	Firmicutes	Clostridia	Lachnospirales	Lachnospiraceae	NA
raber-2020-dss_ASV704	Bacteria	Bacteroidota	Bacteroidia	Bacteroidales	NA	NA
raber-2020-dss_ASV706	Bacteria	Firmicutes	Clostridia	Oscillospirales	Oscillospiraceae	NA
raber-2020-dss_ASV707	Bacteria	Actinobacteriota	Actinobacteria	Micrococcales	Micrococcaceae	Pseudarthrobacter
raber-2020-dss_ASV710	Bacteria	Firmicutes	Clostridia	Lachnospirales	Lachnospiraceae	NA
raber-2020-dss_ASV714	Bacteria	Firmicutes	Clostridia	Oscillospirales	Oscillospiraceae	Sporobacter
raber-2020-dss_ASV715	Bacteria	Firmicutes	Clostridia	Lachnospirales	Lachnospiraceae	NA

raber-2020-dss_ASV716	Bacteria	Firmicutes	Clostridia	Lachnospirales	Lachnospiraceae	NA
raber-2020-dss_ASV717	Bacteria	Patescibacteria	Saccharimonadia	Saccharimonadales	Saccharimonadaceae	Candidatus_Saccharimonas
raber-2020-dss_ASV718	Bacteria	Firmicutes	Clostridia	Oscillospirales	Oscillospiraceae	Oscillibacter
raber-2020-dss_ASV721	Bacteria	Firmicutes	Clostridia	Lachnospirales	Lachnospiraceae	A2
raber-2020-dss_ASV723	Bacteria	Bacteroidota	Bacteroidia	Bacteroidales	Muribaculaceae	NA
raber-2020-dss_ASV730	Bacteria	Firmicutes	Bacilli	NA	NA	NA
raber-2020-dss_ASV731	Bacteria	Firmicutes	Clostridia	NA	NA	NA
raber-2020-dss_ASV732	Bacteria	Firmicutes	Bacilli	NA	NA	NA

raber-2020-dss_ASV733	Bacteria	Bacteroidota	Bacteroidia	Bacteroidales	Muribaculaceae	Muribaculum
raber-2020-dss_ASV734	Bacteria	Firmicutes	Clostridia	Lachnospirales	Lachnospiraceae	NA
raber-2020-dss_ASV735	Bacteria	Firmicutes	Clostridia	Oscillospirales	Oscillospiraceae	Sporobacter
raber-2020-dss_ASV736	Bacteria	Actinobacteriota	Actinobacteria	Micrococcales	Microbacteriaceae	Curtobacterium
raber-2020-dss_ASV737	Bacteria	Firmicutes	Clostridia	Oscillospirales	Ruminococcaceae	NA
raber-2020-dss_ASV742	Bacteria	Firmicutes	Clostridia	Peptostreptococcales-Tissierellales	Anaerovoracaceae	NA
raber-2020-dss_ASV743	Bacteria	Bacteroidota	Bacteroidia	Bacteroidales	NA	NA
raber-2020-dss_ASV744	Bacteria	Firmicutes	Clostridia	NA	NA	NA

raber-2020-dss_ASV745	Bacteria	Firmicutes	Clostridia	Oscillospirales	Ruminococcaceae	NA
raber-2020-dss_ASV746	Bacteria	Firmicutes	Clostridia	Lachnospirales	Lachnospiraceae	NA
raber-2020-dss_ASV747	Bacteria	Firmicutes	Clostridia	Lachnospirales	Lachnospiraceae	NA
raber-2020-dss_ASV750	Bacteria	Firmicutes	Clostridia	Lachnospirales	Lachnospiraceae	NA
raber-2020-dss_ASV767	Bacteria	Firmicutes	Clostridia	Lachnospirales	Lachnospiraceae	NA
raber-2020-dss_ASV769	Bacteria	Firmicutes	Clostridia	Lachnospirales	Lachnospiraceae	NA
raber-2020-dss_ASV770	Bacteria	Firmicutes	Bacilli	RF39	NA	NA
raber-2020-dss_ASV771	Bacteria	Firmicutes	Clostridia	Lachnospirales	Lachnospiraceae	Lachnospiraceae_NK4A136_group

raber-2020-dss_ASV772	Bacteria	Firmicutes	Clostridia	Oscillospirales	Ruminococcaceae	NA
raber-2020-dss_ASV773	Bacteria	NA	NA	NA	NA	NA
raber-2020-dss_ASV774	Bacteria	Bacteroidota	Bacteroidia	NA	NA	NA
raber-2020-dss_ASV775	Bacteria	Firmicutes	NA	NA	NA	NA
raber-2020-dss_ASV776	Bacteria	Firmicutes	Clostridia	Lachnospirales	Lachnospiraceae	NA
raber-2020-dss_ASV778	Bacteria	Firmicutes	Clostridia	Oscillospirales	Ruminococcaceae	NA
raber-2020-dss_ASV779	Bacteria	Firmicutes	Clostridia	Oscillospirales	Oscillospiraceae	NA
raber-2020-dss_ASV780	Bacteria	Bacteroidota	Bacteroidia	Bacteroidales	Bacteroidaceae	Bacteroides

raber-2020-dss_ASV781	Bacteria	Firmicutes	Clostridia	Oscillospirales	NA	NA
raber-2020-dss_ASV782	Bacteria	Firmicutes	Clostridia	Lachnospirales	Lachnospiraceae	NA
raber-2020-dss_ASV783	Bacteria	Firmicutes	Clostridia	Lachnospirales	Lachnospiraceae	NA
raber-2020-dss_ASV784	Bacteria	Firmicutes	Clostridia	Lachnospirales	Lachnospiraceae	Acetatifactor
raber-2020-dss_ASV785	Bacteria	Firmicutes	Clostridia	Lachnospirales	Lachnospiraceae	NA
raber-2020-dss_ASV786	Bacteria	Firmicutes	Bacilli	Staphylococcales	Staphylococcaceae	NA
raber-2020-dss_ASV787	Bacteria	Firmicutes	Clostridia	Lachnospirales	Lachnospiraceae	NA
raber-2020-dss_ASV788	Bacteria	Firmicutes	Clostridia	NA	NA	NA

raber-2020-dss_ASV790	Bacteria	Firmicutes	Clostridia	Lachnospirales	Lachnospiraceae	NA
raber-2020-dss_ASV791	Bacteria	Firmicutes	Clostridia	Lachnospirales	Lachnospiraceae	NA
raber-2020-dss_ASV792	Bacteria	Bacteroidota	Bacteroidia	Bacteroidales	Muribaculaceae	CAG-873
raber-2020-dss_ASV793	Bacteria	Firmicutes	Clostridia	Oscillospirales	Oscillospiraceae	Pseudoflavonifractor
raber-2020-dss_ASV794	Bacteria	Bacteroidota	Bacteroidia	Bacteroidales	Muribaculaceae	NA
raber-2020-dss_ASV796	Bacteria	Firmicutes	Clostridia	Oscillospirales	NA	NA
raber-2020-dss_ASV824	Bacteria	Firmicutes	Clostridia	Lachnospirales	Lachnospiraceae	NA
raber-2020-dss_ASV825	Bacteria	Firmicutes	Clostridia	Lachnospirales	Lachnospiraceae	NA

raber-2020-dss_ASV826	Bacteria	Bacteroidota	Bacteroidia	Bacteroidales	Muribaculaceae	CAG-873
raber-2020-dss_ASV827	Bacteria	Firmicutes	Clostridia	Oscillospirales	Ruminococcaceae	NA
raber-2020-dss_ASV828	Bacteria	Firmicutes	Clostridia	Lachnospirales	Lachnospiraceae	NA
raber-2020-dss_ASV829	Bacteria	Bacteroidota	Bacteroidia	Bacteroidales	Muribaculaceae	NA
raber-2020-dss_ASV830	Bacteria	Bacteroidota	Bacteroidia	Bacteroidales	Muribaculaceae	NA
raber-2020-dss_ASV831	Bacteria	Bacteroidota	Bacteroidia	Bacteroidales	Bacteroidaceae	Bacteroides
raber-2020-dss_ASV833	Bacteria	Bacteroidota	Bacteroidia	Bacteroidales	Rikenellaceae	NA
raber-2020-dss_ASV834	Bacteria	Bacteroidota	Bacteroidia	Bacteroidales	Muribaculaceae	NA

raber-2020-dss_ASV835	Bacteria	Firmicutes	Clostridia	Lachnospirales	Lachnospiraceae	NA
raber-2020-dss_ASV836	Bacteria	Firmicutes	Clostridia	Oscillospirales	NA	NA
raber-2020-dss_ASV838	Bacteria	Firmicutes	NA	NA	NA	NA
raber-2020-dss_ASV839	Bacteria	Firmicutes	Clostridia	Lachnospirales	Lachnospiraceae	NA
raber-2020-dss_ASV840	Bacteria	Bacteroidota	Bacteroidia	Bacteroidales	NA	NA
raber-2020-dss_ASV841	Bacteria	Firmicutes	NA	NA	NA	NA
raber-2020-dss_ASV842	Bacteria	Firmicutes	Clostridia	Lachnospirales	Lachnospiraceae	NA
raber-2020-dss_ASV843	Bacteria	Bacteroidota	Bacteroidia	Bacteroidales	Muribaculaceae	NA

raber-2020-dss_ASV844	Bacteria	Bacteroidota	Bacteroidia	Bacteroidales	Muribaculaceae	NA
raber-2020-dss_ASV845	Bacteria	Firmicutes	Clostridia	NA	NA	NA
raber-2020-dss_ASV846	Bacteria	Patescibacteria	Saccharimonadia	Saccharimonadales	Saccharimonadaceae	Candidatus_Saccharimonas
raber-2020-dss_ASV847	Bacteria	Firmicutes	Clostridia	Lachnospirales	Lachnospiraceae	NA
raber-2020-dss_ASV850	Bacteria	Bacteroidota	Bacteroidia	Bacteroidales	NA	NA
raber-2020-dss_ASV852	Bacteria	Firmicutes	NA	NA	NA	NA
raber-2020-dss_ASV853	Bacteria	Firmicutes	Clostridia	Lachnospirales	Lachnospiraceae	NA
raber-2020-dss_ASV854	Bacteria	Firmicutes	Clostridia	Oscillospirales	Oscillospiraceae	Pseudoflavonifractor

raber-2020-dss_ASV855	Bacteria	Firmicutes	Clostridia	Lachnospirales	Lachnospiraceae	NA
raber-2020-dss_ASV859	Bacteria	Firmicutes	Clostridia	Oscillospirales	Butyricicoccaceae	UCG-008
raber-2020-dss_ASV905	Bacteria	Firmicutes	Clostridia	Lachnospirales	Lachnospiraceae	NA

Table S4. DSS metadata.

Sample.ID	SID	Sex	Geno	DSS	Timepoint.Week	OF1.TotDistMove.cm	OF1.CentDur	NO1.SOTotDistMove.cm	SO.total.dm.all.3.days	NO2.DO.TotDistMove.cm	NO2.DO.Pct.Obj2	NO2.DO.DI	Y.Maze.Total.Arm.Entries	Y.Maze.Pct.Spont Alt	Delta.rotorod	FallScore	Reach Score	Grip.Strength.3.g	Grip.Wk.3.weight.g	Grip. Ratio	FST.Scoring	ContextFC.ContTotPctFrz	Context.FC.CuedTonePctFrz
594wk4-1	1	M	WT	0%	4	878.29	0	2016.81	460.674	1502.48	32.3272322	-0.3534554	19	52.9411765	10.7	9	0	126	27.6	4.56521739	23.3333333	69.22	41.17
594wk4-2	2	M	Het	0%	4	1471.63	8.40838	2631.61	1069.604	1908.24	64.5885105	0.29177021	28	53.8461539	-14.1	6	0	67.2	25.2	2.66666667	11.6666667	50.57	8.24
594wk4-3	3	M	Het	3%	4	1164.23	12.4791	1669.15	747.047	1448.41	40.0000245	-0.1999995	15	46.1538462	1.7	9	0	112.5	30.6	3.67647059	3.33333333	84.73	13.81
594wk4-4	4	F	WT	3%	4	2070.77	15.9492	2822.02	912.59	2013.67	36.5759712	-0.2684806	36	52.9411765	-16.6	9	0	92	19	4.84210526	0	74.19	1.56
594wk4-5	5	F	WT	0%	4	2105.13	9.00898	2567.65	510.08	2575.4	68.4101133	0.36820227	20	33.3333333	29	6	3	97.2	24.1	4.03319502	6.66666667	48.94	1.44
594wk4-6	6	F	Het	3%	4	1480.37	0	2230.5	516.074	1954.17	19.0476318	-0.6190474	15	23.0769231	0.6	5	0	128.2	22.3	5.74887892	8.33333333	39.88	14.57
594wk4-7	7	F	Het	0%	4	1458.68	17.017	2088.3	206.4	1173.14	72.493564	0.44987128	13	54.5454546	13.3	8	0	83.5	22.6	3.69469027	36.6666667	85.04	73.52
594wk4-8	8	M	WT	3%	4	1233.78	34.1674	1404.63	552.637	1359.87	73.8128399	0.4762568	18	87.5	5.4	8	0	100.5	25.9	3.88030888	36.6666667	54.86	25.2
594wk4-9	9	M	WT	0%	4	1281.82	10.0767	1491.28	458.501	1488.18	NA	NA	20	44.4444444	22	9	0	107.5	25.6	4.19921875	23.3333333	77.24	26.57
594wk4-10	10	M	Het	3%	4	1244.53	18.0847	1674.94	918.556	837.252	NA	NA	14	58.3333333	15.8	5	0	120.5	24.4	4.93852459	11.6666667	81.24	10.7
594wk4-11	11	M	Het	0%	4	1986.11	16.6166	3515.36	720.97	2420.52	81.2976789	0.62595358	22	55	1.8	4	1	117	21.7	5.39170507	15	36.81	33.28
594wk4-12	12	F	WT	3%	4	2124.24	22.6226	2199.28	1334.148	2747.43	58.6583194	0.17316639	25	39.1304348	6.1	7	0	96	23.5	4.08510638	25	70.24	35.91
594wk4-13	13	F	WT	0%	4	1311.7	0	1821.92	386.581	1468.84	83.1695231	0.66339046	15	23.0769231	13.3	5	2	80.7	20.9	3.86124402	16.6666667	72.99	33.61
594wk4-14	14	F	WT	3%	4	1306.84	10.6106	2511.31	675.51	1326.12	37.7358527	-0.2452829	14	25	20.3	5	1	96	23.2	4.13793103	43.3333333	73.1	8.17
594wk4-15	15	F	Het	0%	4	3276.91	28.2949	3714.74	1809.34	3488.76	47.6662018	-0.046676	21	52.631579	6.8	9	2	108	24.2	4.46280992	23.3333333	40.74	9.43
594wk4-16	16	M	Het	0%	4	1704.38	25.759	3321.37	943.376	2647.62	55.9386973	0.11877395	17	73.3333333	17	4	3	150.5	27.8	5.41366907	28.3333333	81.17	20.96
594wk4-17	17	M	Het	3%	4	812.729	10.8775	1311.05	350.516	1353.81	60.0733111	0.20146622	13	54.5454546	5.1	6	0	125.5	28.8	4.35763889	30	73.43	52.28
594wk4-18	18	F	Het	3%	4	1690.28	14.014	3854.12	971.119	4180.94	51.7112047	0.0342241	28	34.6153846	-1.1	7	0	126	24.8	5.08064516	40	75.77	24.3
594wk4-19	19	F	Het	0%	4	1228.12	12.0787	2314.5	304.877	1360.32	61.5176506	0.23035301	14	58.3333333	-4.3	8	0	107.5	25.5	4.21568628	5	64.33	36.83

594wk 4-20	20	F	WT	0%	4	1137.5	7.00698	1794.9	485.61	1641.04	69.2307536	0.38461507	24	40.9090909	8.3	6	1	96	21.2	4.52830189	50	82.76	4.19
594wk 4-21	21	F	Het	3%	4	3016.85	19.4861	7341.13	302.82	6048.21	58.1594325	0.16318865	28	38.4615385	14.6	8	1	87.5	26.6	3.28947368	38.3333333	75.03	17.91
594wk 4-22	22	F	Het	0%	4	753.011	2.002	1020.49	57.179	945.582	NA	NA	9	71.4285714	41.8	8	0	90.5	23.1	3.91774892	13.3333333	72.36	53.22
594wk 4-23	23	M	Het	3%	4	1049.81	6.87352	1794.37	431.151	1414.57	57.6922634	0.15384527	19	35.2941177	-5.1	0	0	71.2	24.9	2.85943775	25	53.96	8.2
594wk 4-24	24	M	Het	0%	4	1912.33	12.0787	4622.27	-79.02	4044.95	62.8016405	0.25603281	29	55.5555556	50.3	8	0	106.2	23.3	4.55793991	20	22.49	1.89
594wk 4-25	25	M	Het	3%	4	1392.18	32.1654	2064.98	656.569	1702.97	68.6411337	0.37282267	24	50	28.1	7	0	83	25.3	3.28063241	61.6666667	69.02	29.44
594wk 4-26-kit	26	M	WT	3%	4	1338.4	4.87152	1871.72	724.87	1820.9	34.0886012	-0.318228	17	40	-12.7	9	1	97.2	23	4.22608696	50	88.04	47.96
594wk 4-27	27	M	WT	0%	4	1590.97	41.1744	2445.91	769.717	1044.56	34.920633	-0.3015873	21	47.3684211	2.3	8	2	114.2	24.1	4.73858921	50	49.38	23.22
594wk 4-28	28	M	Het	0%	4	882.171	45.3786	2069.9	-194.899	2149.78	72.3756325	0.44751265	18	62.5	26.6	10	0	110.2	23.4	4.70940171	0	55.72	43.3
594wk 4-29	29	F	Het	0%	4	1734.62	14.748	1869.07	794.353	1773.11	39.2618413	-0.2147632	29	70.3703704	23.7	10	0	99	21.5	4.60465116	38.3333333	46.12	3.19
594wk 4-30	30	F	Het	3%	4	1547.28	12.1454	1636.1	906.188	1316.04	68.5714775	0.37142955	14	58.3333333	-0.5	6	1	82	21	3.90476191	10	54.27	31.11
